# Corneal Allogeneic Intrastromal Ring Segments for Treating Keratoconus—Systematic Review and Meta-Analysis

**DOI:** 10.3390/medicina62030523

**Published:** 2026-03-12

**Authors:** Eline Elodie Barbara De Clerck, Johann Krüger, Martina Kropp, Horace Massa, Bojan Pajic, Josef Guber, Gabriele Thumann, Ivo Guber

**Affiliations:** 1 Division of Ophthalmology, Department of Clinical Neurosciences, Geneva University Hospitals, 1205 Geneva, Switzerland; martina.kropp@unige.ch (M.K.); horace.massa@hug.ch (H.M.); bojan.pajic@orasis.ch (B.P.); gabriele.thumann@hug.ch (G.T.); ivo.guber@augenchirurgie.ch (I.G.); 2 Experimental Ophthalmology, University of Geneva, 1205 Geneva, Switzerland; 3 Tygervalley Eye Laser Cataract Hospital, Cape Town 7530, South Africa; drkruger@eyelaserclinic.co.za; 4 Department of Physics, Faculty of Sciences, University of Novi Sad, Trg Dositeja Obradovica 4, 21000 Novi Sad, Serbia; 5 Faculty of Medicine of the Military Medical Academy, University of Defense, 11000 Belgrade, Serbia; 6 Eye Clinic ORASIS, Swiss Eye Research Foundation, 5734 Reinach, Switzerland; 7 Department of Ophthalmology, University of Basel, 4031 Basel, Switzerland; josef.guber@augenchirurgie.ch

**Keywords:** keratoconus, corneal allogeneic intrastromal ring segments, implantation

## Abstract

*Background and Objectives*: Corneal allogeneic intrastromal ring segments (CAIRS) are designed to decrease and stabilize the extent of corneal ectasia in keratoconus patients. This systematic review and meta-analysis evaluate the effectiveness of different surgical techniques for CAIRS preparation and the adjunctive use of corneal cross-linking. *Materials and Methods*: Following the PRISMA statement and checklist, a comprehensive search was conducted in Embase, Medline, and the Cochrane Controlled Trials Register, through the use of a systematic search approach in accordance with the Cochrane Collaboration guidelines. *Results*: Eighteen studies, involving 567 eyes of 459 patients, met the inclusion criteria. At one month postoperatively, CAIRS implantation significantly improved uncorrected visual acuity (UCVA) (−0.45 logMAR, 95% CI [−0.59 to −0.31], *p* < 0.001) and best corrected visual acuity (BCVA) (−0.36 logMAR, 95% CI [−0.46 to −0.25], *p* < 0.001). These improvements remained significant after one year (UCVA: −0.39 logMAR, 95% CI [−0.48 to −0.30], *p* < 0.001; BCVA: −0.34 logMAR, 95% CI [−0.50 to −0.18], *p* < 0.001). Similarly, mean simulated keratometry (Kmean) decreased by −4.42 D (95% CI [−5.94 to −2.90], *p* < 0.001) and maximum keratometry (Kmax) by −3.88 D (95% CI [−6.71 to −1.05], *p* < 0.001) at one month, with sustained reductions at one year (−3.59 D, 95% CI [−4.35 to −2.84], *p* < 0.001 and −3.73 D, 95% CI [−4.91 to −2.55], *p* < 0.001). No significant differences in surgical outcome have been observed between the different surgical techniques. *Conclusions*: CAIRS implantation appears to be an effective treatment option for keratoconus, regardless of the technique used for segment preparation or the addition of corneal cross-linking. No approach demonstrated clear clinical superiority over others in the first year after surgery.

## 1. Introduction

Keratoconus is a gradually advancing corneal disease distinguished by asymmetric ectasia [1,2]. This condition manifests through corneal thinning and irregular astigmatism, leading to substantial impairment in visual function. Its prevalence is estimated at approximately 289 per 100,000 individuals, with a notably higher incidence among younger populations [3]. Despite extensive research, the relative contributions of genetic, environmental, mechanical, and inflammatory factors to its pathogenesis remain unclear [4,5,6].

Keratoconus severity is commonly graded from stage 1 to 4 according to the Amsler–Krumeich classification [7]. Conventional management strategies include avoidance of eye rubbing, contact lenses, corneal cross-linking, and the implantation of intracorneal ring segments (ICRS). For advanced keratoconus or when patients are unable to tolerate contact lenses, deep anterior lamellar keratoplasty or penetrating keratoplasty remain the preferred surgical approaches. However, recent surgical innovations, such as corneal allogeneic intrastromal ring segments (CAIRS), have demonstrated promising results in stabilizing corneal ectasia, even in advanced cases [8].

**Figure 1:**
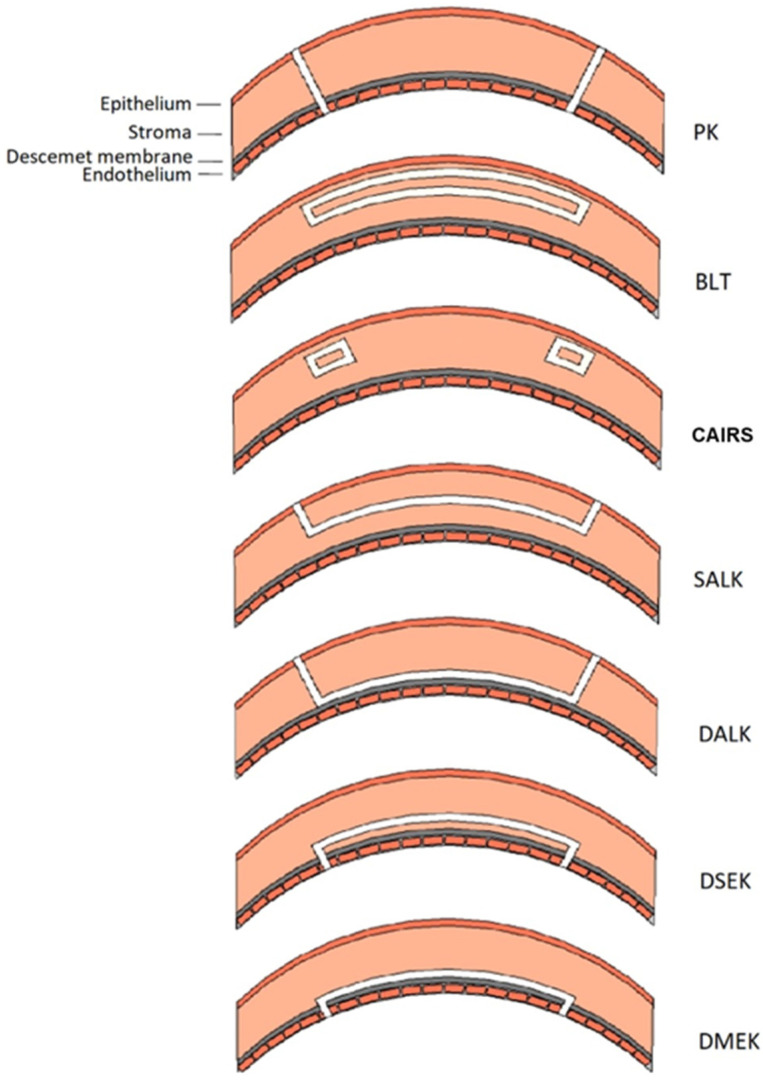
Schematic representation of different types of keratoplasty. The white section represents transplanted tissue. PK = penetrating keratoplasty. BLT = Bowman layer transplantation. CAIRS = corneal allogeneic intrastromal ring segments. SALK = superficial anterior lamellar keratoplasty. DALK = deep anterior lamellar keratoplasty. DSEK = Descemet stripping endothelial keratoplasty. DMEK = Descemet membrane endothelial keratoplasty. Adapted from a previous paper of De Clerck and colleagues [7].

**Figure 2:**
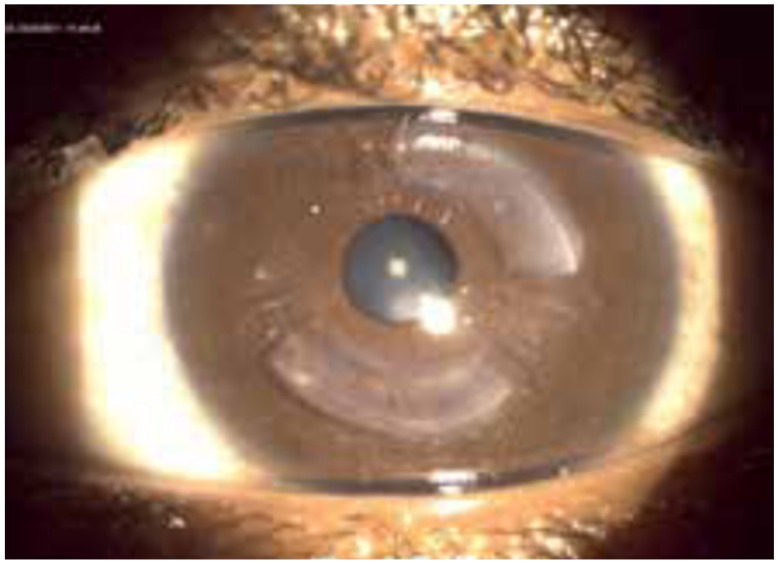
CAIRS segment in keratoconus eye. Reproduced with permission from Krüger [11].

CAIRS offers several advantages over synthetic ICRS. Allogeneic tissue presents superior biocompatibility, allowing a more superficial implantation and improved corneal regularization in accordance with Barraquer’s law [9]. CAIRS are small curvilinear segments, created from donor corneal button tissue, intended for stromal implantation to modify corneal curvature (Figure 1 and Figure 2). Modification of corneal curvature and surface regularization are induced by their arc-shortening effect in combination with their role as spacer elements [10].

Several systematic reviews have recently examined CAIRS outcomes [9,12,13,14], presenting the performance of this novel technique developed by S. Jacob et al. [10]. These reviews point out the heterogeneity of the surgical protocols derived from the original proposal, all showing potential benefits for the surgeon or the patient. However, no performance comparison of the different techniques for CAIRS preparation (trephined blade vs. femtosecond laser, dehydrated vs. non-dehydrated) and the use of adjunctive cross-linking (cross-linking vs. no cross-linking) is proposed. This systematic review aims to address this gap by comparing the performance of different techniques for CAIRS surgery.

## 2. Materials and Methods

### 2.1. Search Strategy and Selection Criteria

Following the PRISMA statement and checklist [15,16], a comprehensive search was conducted in Embase, Medline, and the Cochrane Controlled Trials Register, through the use of a systematic search approach in accordance with the Cochrane Collaboration guidelines (Section S3, p. 231). The search was conducted for studies published up to 8 July 2025, using the terms “keratoconus” and “corneal allogenic intrastromal ring segments”, in English, German, French, or Dutch, in human subjects.

Case–control studies, prospective and retrospective case series, randomized controlled trials, and cohort studies of adults (≥18 years) with keratoconus were included. Included studies were required to report one or more of the predefined outcome measures of CAIRS. Although the inclusion criteria were predefined and documented in a protocol, the protocol was not registered prior to the initiation of the review. The assessed outcome variables included the (1) UCVA (logMAR), (2) BCVA (logMAR), (3) pachymetry thinnest point (µm), (4) pachymetry central point (µm), Kmax (D), (5) mean simulated keratometry Kmean (D)—i.e., the mean refractive power of the two principal meridians, (6) total higher-order aberrations, (7) spherical aberration, (8) vertical coma, (9) horizontal coma, (10) trefoil, and (11) total RMS.

The study selection was done by Eline De Clerck (EDC) and checked by Ivo Guber (IG). The study selection was conducted in two stages. In the first stage, the title, abstract, and keywords were screened, and comments, letters, and reviews were excluded. In the second stage, the full text of eligible papers was assessed. Studies were included if they evaluated one or more of the predefined postoperative outcome measures. Studies were excluded if they involved patients with non-keratoconus forms of ectasia or the presence of ocular comorbidities, other intrastromal tissue transplantations or CAIRS as a secondary surgery, e.g., after ICRS removal.

### 2.2. Data Extraction and Analysis

EDC assessed the studies for inclusion and quality and performed the data extraction. Therefore, an extraction sheet based on the Cochrane Costumers and Communication Review Group’s data extraction template was used [16,17]. The data were cross-checked by IG. Any discrepancies were resolved through discussion between EDC and IG. Studies were not blinded for the journal or any other characteristics related to the journal. The data extracted included: author and year of publication, study type, study design, country, number of patients with keratoconus, subgroup characteristics, in- and exclusion criteria, details of the intervention (i.e., (1) donor tissue preparation [levy, cutout, soaking, dehydration/rehydration], (2) surgical technique [incision, implantation depth, tunnel parameters, cross-linking], and (3) postoperative treatment), the outcome variables studied, and mean values and standard deviation (SD) of the variables. In addition, data on patient age, sex, and stage of keratoconus were extracted.

Methodological quality was evaluated using the Delphi list [18] supplemented with one additional criterion. Table 1 describes the five quality domains assessed: (1) whether patients were enrolled consecutively (continuous reporting), (2) whether inclusion criteria were specified, (3) whether exclusion criteria were specified, (4) whether point estimates and measures of variability were presented for the primary outcome measures (significance and reliability of the results), and (5) whether calculation of statistical power was reported (planning and aforethought in study design). Each item was rated as “Yes” (high quality), “Unclear” (uncertain quality), or “No” (low quality).

Risk of bias was evaluated in accordance with the Cochrane guidelines [35]. Four domains were assessed: (1) whether data collectors were blinded to the identity and clinical results of the patients (performance bias), (2) whether outcome assessors were blinded to the identity and clinical results of the patients (detection bias), (3) whether study reports were free from selective outcome reporting (reporting bias), and (4) whether the study was free from additional sources of bias (selection bias, attrition bias, or other bias). These items were rated as “Yes” (low risk of bias), “Unclear” (uncertain risk of bias), or “No” (high risk of bias).

Pooled analyses were conducted using random-effects models to account for heterogeneity among the included studies with respect to study populations, interventions, and outcome measures [36]. Microsoft^®^ Excel^®^ 2016 MSO (Version 2302 Build 16.0.16130.20186) and Review Manager version 5.4.1 were used to perform statistical analyses.

Pooled analyses were performed (1) in the total study population, (2) according to CAIRS preparation (trephined blade vs. femtosecond laser), (3) CAIRS hydration (dehydrated vs. non-dehydrated), (4) and the use of cross-linking or not. CAIRS preparation by KeraNatural^®^ has been included in the trephine subgroup.

The mean preoperative outcome measures of patients with keratoconus were compared with the corresponding postoperative outcome measures obtained at 1 month, 6 months, and 1 year following surgery to evaluate the performance of CAIRS.

Changes in outcome measures were evaluated summary effect estimates derived from random-effects meta-analyses with 95% CIs [37]. Negative values indicated a decrease in the outcome measure after CAIRS surgery compared with its preoperative value. Heterogeneity between studies was assessed using the χ^2^ (*p* < 0.05 indicating significant heterogeneity) and the I^2^ statistic (values ≥ 30% indicating significant heterogeneity) [38].

Welch’s *t*-test was applied when comparing the mean of two groups, to confirm if the superiority of the group with the highest mean was significant. If the result of the test was not significant, no higher benefit could be concluded.

## 3. Results

### 3.1. Selected Studies

**Figure 3:**
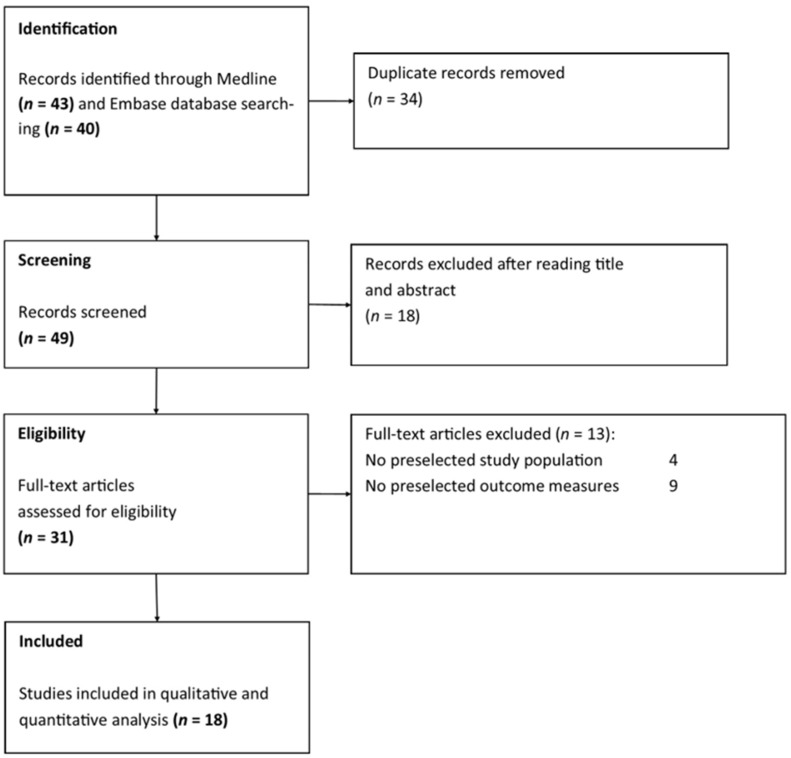
Flow diagram of study selection.

A total of 83 articles were found through database searches. After duplicate records were removed, 49 articles remained for screening. Thirty-one articles assessed CAIRS in patients with keratoconus. Finally, 18 studies met our inclusion criteria (Figure 3). Table 2 and Section S2 summarize the characteristics of the included studies. One study was a prospective cohort study [8], one was a retrospective cohort study [34], seven were prospective case series [10,20,29,30,31,32,33], and nine were retrospective case series [19,21,22,23,24,25,26,27,28].

### 3.2. Quality Assessment

Information regarding the consecutiveness of the sample was adequately reported in two of the 18 studies [19,20]. Inclusion criteria were specified in 17 studies [8,10,19,21,22,23,24,25,26,27,28,29,30,31,32,33,34] and exclusion criteria were specified in 13 studies [8,10,19,21,23,25,26,27,28,30,31,33,34]. Selection criteria varied across included studies. Overall, the samples included patients with keratoconus ranging from stage I to stage IV (Table 2). Eleven studies excluded the presence of corneal scarring [10,19,22,23,25,26,27,28,30,31,34], 13 studies excluded previous corneal or intraocular surgery [10,19,21,22,23,25,26,27,28,30,31,33,34], 11 studies excluded autoimmune or connective tissue diseases [10,21,22,23,25,27,28,30,31,33,34], and 10 studies excluded a history of viral keratitis [10,19,22,23,25,28,30,31,33,34]. All but one of the included studies provided point estimates and SDs for the outcome variables [20]. Calculation of statistical power was only reported in three studies [19,29,34]. Section S2 shows the quality assessment of the included studies (pp. 198–230). Table 1 summarizes the quality assessment items applied in the included studies.

### 3.3. Risk of Bias in Included Studies

Section S2 provides a detailed evaluation of the risk of bias for all included studies. None of the studies described masking of data collectors or outcome assessors. Selective outcome reporting could not be determined in 15 studies [8,10,20,22,23,25,26,27,28,29,30,31,32,33,34]. All studies appeared vulnerable to additional bias, primarily due to small sample sizes and the lack of a control group.

### 3.4. Outcome Analyses and Investigation of Heterogeneity

Baseline characteristics of the study populations are summarized in Table 2. In total, eighteen studies met our inclusion criteria, with a total of 567 eyes from 459 patients. The mean age of the patients was 35 years, with an overall range of 20–67 years, and 248 patients (72%) were male. Based on the Amsler–Krumeich classification, 6 studies included patients with keratoconus stages I–IV [10,19,26,27,30,32], one study included keratoconus stages I–III [31], one study only included keratoconus stage II [21], six studies included keratoconus stages II–IV [8,20,22,23,24,29,34], one study included keratoconus stages III–IV [33], and two studies did not specify the keratoconus stage [25,28] (Table 2). One study has evaluated the relationship between keratoconus severity and clinical outcomes and demonstrated that in advanced-stage keratoconus (Kmax > 75D), CAIRS implantation did not lead to a significant improvement in visual acuity [32]. Table 3 summarizes the surgical techniques used for CAIRS implantation in the included studies. Thirteen studies used a trephined blade [8,10,19,22,24,25,26,28,29,30,31,33,34] and five studies used a femtosecond laser for CAIRS preparation [20,21,23,27,32]. Nine studies performed dehydration [8,20,21,22,23,26,27,32,34] and three studies implanted non-dehydrated segments [10,24,30]. Four studies used cross-linking [10,19,20,30], 13 studies did not use cross-linking [8,21,22,23,24,25,26,27,29,31,32,33,34], and one study compared outcomes between patients who underwent preoperative cross-linking and those who did not [28].

Table 4, Table 5, Table 6 and Table 7 present numerical values for all outcome measures. Section S1 provides the corresponding graphical representations and χ^2^ and I^2^ statistics used to assess heterogeneity.

#### 3.4.1. Total Study Population

In the total study population (Table 4), patients who underwent CAIRS surgery had significantly higher UCVA one month (*p* < 0.001), 6 months (*p* < 0.001), and 1 year postoperatively (*p* < 0.001) and significantly higher BCVA (*p* < 0.001). Kmax was significantly decreased in patients 1 month (*p* < 0.001), 6 months (*p* < 0.001), and 1 year postoperatively (*p* < 0.001) compared to preoperative values. Kmean values were also significantly decreased in patients 1 month (*p* < 0.001), 6 months (*p* < 0.001), and 1 year postoperatively (*p* < 0.001) compared to preoperative values. Total higher-order aberrations were significantly decreased in patients 1 month (*p* < 0.001), 6 months (*p* = 0.02), and 1 year postoperatively (*p* < 0.01) compared to preoperative values. Spherical aberration was only significantly increased 1 month postoperatively (*p* < 0.001), but not 6 months and 1 year postoperatively. Vertical coma showed a significant decrease 1 month (*p* = 0.02) and 6 months (*p* < 0.001) postoperatively, but not 1 year postoperatively. Trefoil was significantly increased 1 month (*p* < 0.001), 6 months (*p* < 0.01), and 1 year postoperatively (*p* = 0.03) compared to preoperative values.

#### 3.4.2. CAIRS Preparation: Trephined Blade vs. Femtosecond Laser

In the trephined blade subgroup (Table 5A), patients who underwent CAIRS surgery had significantly higher UCVA one month (*p* < 0.001), 6 months (*p* < 0.001), and 1 year postoperatively (*p* < 0.001) and significantly higher BCVA (*p* < 0.001). Kmax was significantly decreased in patients 1 month (*p* = 0.04) and 1 year postoperatively (*p* < 0.001) compared to preoperative values. Kmean values were also significantly decreased in patients 1 month (*p* < 0.001), 6 months (*p* < 0.001), and 1 year postoperatively (*p* < 0.001) compared to preoperative values. Total higher-order aberrations were significantly decreased in patients 1 month (*p* = 0.02) and 1 year postoperatively (*p* = 0.03). Spherical aberration was significantly increased 1 month postoperatively (*p* < 0.001) and 6 months postoperatively (*p* < 0.001), but not 1 year postoperatively. Vertical coma was only significantly decreased at 1 month (*p* < 0.001). Trefoil showed a significant increase at 1 month (*p* < 0.001) and 6 months (*p* < 0.001).

In the femtosecond laser subgroup (Table 5B), patients who underwent CAIRS surgery had significantly higher UCVA one month (*p* = 0.03), 6 months (*p* < 0.001), and 1 year postoperatively (*p* < 0.001) and a significantly higher BCVA (*p* < 0.001). Kmax was significantly decreased in patients 1 month (*p* = 0.04), 6 months (*p* < 0.01), and 1 year postoperatively (*p* = 0.04) compared to preoperative values. Kmean values were also significantly decreased in patients 1 month (*p* = 0.01), 6 months (*p* < 0.01), and 1 year postoperatively (*p* < 0.001). Total higher-order aberrations were significantly decreased in patients 6 months (*p* = 0.03) and one year postoperatively (*p* = 0.03). Spherical aberration was only significantly increased one month postoperatively (*p* = 0.04). Vertical coma showed a significant decease 6 months (*p* < 0.001) and 1 year postoperatively (*p* < 0.001). Trefoil showed a significant increase at 1 month (*p* = 0.02) and 6 months (*p* = 0.02). Total RMS showed a significant decrease at 1 month (*p* < 0.01) and 6 months (*p* < 0.01).

Welch’s *t*-test did not show a significant difference between the two groups for BCVA and Kmax (Section S1, Table S1.4.4.1, p. 196).

#### 3.4.3. CAIRS Dehydration vs. No Dehydration

In the non-dehydrated subgroup (Table 6A), patients who underwent CAIRS surgery had significantly higher UCVA 6 months (*p* < 0.01) and 1 year postoperatively (*p* < 0.001) and significantly higher BCVA one year postoperatively (*p* = 0.001). Kmax was significantly decreased in patients 1 year postoperatively (*p* < 0.01). Vertical coma was significantly increased 1 year postoperatively (*p* = 0.01).

In the dehydrated subgroup (Table 6B), patients who underwent CAIRS surgery had a significantly higher UCVA one month (*p* < 0.01), 6 months (*p* = 0.001), and 1 year postoperatively (*p* < 0.001) and significantly higher BCVA (*p* < 0.001). Kmax was significantly decreased in patients one month (*p* < 0.01) and 1 year postoperatively (*p* < 0.001). Kmean values were also significantly decreased in patients 1 month (*p* < 0.001), 6 months (*p* < 0.001), and 1 year postoperatively (*p* < 0.001). Total higher-order aberrations were significantly decreased in patients 1 month (*p* < 0.01), 6 months (*p* = 0.03), and one year postoperatively (*p* < 0.01). Spherical aberration was only significantly increased one month postoperatively (*p* < 0.001). Vertical coma showed a significant decrease one month (*p* = 0.02), 6 months (*p* < 0.001) and 1 year postoperatively (*p* < 0.001). Trefoil showed a significant increase 1 month (*p* < 0.001), 6 months (*p* < 0.01), and 1 year postoperatively (*p* = 0.03).

Welch’s *t*-test showed a significant difference between the two groups for BCVA at 6 months (*p* = 0.04) and 1 year (*p* < 0.001), but not for Kmax (Section S1, Table S1.4.4.2, p. 196).

#### 3.4.4. Corneal Cross-Linking or Not

In the corneal cross-linking subgroup (Table 7A), patients who underwent CAIRS surgery had significantly higher UCVA 1 month (*p* < 0.001), 6 months (*p* < 0.001), and 1 year postoperatively (*p* < 0.001) and significantly higher BCVA one month (*p* < 0.01) and 6 months postoperatively (*p* < 0.01). Kmax was significantly decreased in patients 6 months (*p* < 0.01) and 1 year postoperatively (*p* < 0.001). Kmean values were also significantly decreased in patients 1 month (*p* < 0.001), 6 months (*p* < 0.001), and 1 year postoperatively (*p* < 0.001). Vertical coma showed a significant increase one year postoperatively (*p* = 0.01).

In the non-cross-linking subgroup (Table 7B), patients who underwent CAIRS surgery had significantly higher UCVA 1 month (*p* < 0.001), 6 months (*p* < 0.001), and 1 year postoperatively (*p* < 0.001) and significantly higher BCVA one month (*p* < 0.001), 6 months (*p* < 0.001), and one year postoperatively (*p* < 0.001). Pachymetry thinnest point was only significantly decreased in patients 1 month postoperatively (*p* = 0.04). Kmax was significantly decreased in patients one month (*p* < 0.01), 6 months (*p* = 0.04) and 1 year postoperatively (*p* < 0.001). Kmean values were also significantly decreased in patients 1 month (*p* < 0.001), 6 months (*p* < 0.001), and 1 year postoperatively (*p* < 0.001). Total higher-order aberrations were significantly decreased in patients 1 month (*p* < 0.01), 6 months (*p* = 0.02), and one year postoperatively (*p* < 0.01). Spherical aberration was only significantly increased one month postoperatively (*p* < 0.001). Vertical coma showed a significant decrease one month (*p* = 0.02), 6 months (*p* < 0.001), and 1 year postoperatively (*p* < 0.001). Trefoil showed a significant increase 1 month (*p* < 0.001), 6 months (*p* < 0.01), and 1 year postoperatively (*p* = 0.03).

Welch’s *t*-test did not show a significant difference between the two groups for BCVA and Kmax (Section S1, Table S1.4.4.3, p. 197).

## 4. Discussion

Benefits of CAIRS surgery with respect to ICRS are lower risk of complication [10], higher biocompatibility [9], and possible customization of the implants, even if sophisticated nomograms are needed to optimize preoperative planning [9]. Although the core principles of the CAIRS procedure remain consistent in all studies, there is considerable variation in specific parameters such as segment shape, thickness, arc length, implantation depth, and optical zone diameter [14].

This systematic review compares the use of trephined blade versus femtosecond laser for segment preparation, dehydration of the segments versus no dehydration, and the impact of cross-linking versus no cross-linking on a follow-up period up to 1 year. The parameters selected to assess the performance of CAIRS surgery across the different groups are improvement of BCVA for visual function and reduction in Kmax for topographic correction, which are clinically meaningful outcome variables. To the best of our knowledge, other reviews focus on treatment performance without applying a statistical approach to comparing the surgical options performance. This systematic review did not identify a significant difference in surgical outcome between the different surgical techniques.

Firstly, when preparing the surgery, the ophthalmologist must define the dimensions of CAIRS and the position of the tunnel as described in the nomograms [14]. This includes segment thickness [300 µm–750 µm], segment width [400 µm–1500 µm], arc length [90–270°], segment inner diameter [4 mm–6.5 mm], segment outer diameter [7 mm–8 mm], the use of unique or double segments, implantation depth [225 µm–350 µm], and tunnel width [0.7 mm–1.75 mm] [14]. The position of the segments is based on cone location and severity.

Another pre-operative parameter identified is the preparation technique of the segments. The method proposed by Jacob et al. is using a trephined blade [10], but other authors report the use of a femtosecond laser for cutting the implants [21]. Also, pre-cut segments from eye banks (like KeraNatural^®^) are used in other studies [25] not discussed here. When comparing the two groups with predefined performance criteria, no technique showed a significant superiority to the other. The choice of the method will therefore depend on the ophthalmologist’s preference, the availability of the trephined blade or the setup of the femtosecond laser, as well as economic considerations. All selected articles use a femtosecond laser for tunnel creation, except two [29,39]. The advantages of femtosecond laser are the possibility of segments customization and maximization of the production of segments from each cornea by eye banks [21,32]. Also, orientation of the segments Bowman’s layer perpendicularly to the patient cornea can enhance the flattening effect [21].

The second question aims to detect the influence of dehydration of the implanted segments on surgical performance. This adaptation of the original surgical protocol [10] was introduced by Parker et al. [40]. Dehydrating the implant makes it stiffer and allows an easier insertion in the tunnel. Also, the segment can be shaped on a mold when it loses its water content [41]. After applying the Welch’s t test to the two groups, no significant superiority of one procedure could be identified. Therefore, the practical use of dehydrated segments should help beginners and experienced ophthalmologists to perform faster and easier CAIRS surgery, especially for larger segments [41].

The additional effect of performing UV cross-linking of cornea collagen is an important question identified in this review. The goal of cross-linking is to slow down or prevent keratoconus progression. Some authors even consider that it can improve corneal curvature [13]. Pre-operative cross-linking makes the cornea stiffer and reduces the flattening effect of the surgery [28]. This stiffening effect can be used for the preparation of the segments, as an alternative or complement to dehydration [20]. For already cross-linked corneas or non-progressive cases, postoperative cross-linking is not indicated [30]. No single study reports progression of the keratoconus after surgery within the follow-up period. However, longer follow-up periods of 10 years and more are needed to confirm these preliminary findings. Whether CAIRS slows or prevents keratoconus progression, particularly in younger patients, remains uncertain [9]. Among the studies with post-operative cross-linking, the timing can vary from direct post-operation [10,30] to a 6-month period [25]. It has been reported that changes in visual acuity, refractive status, and corneal topographic outcomes take place primarily during the first month after surgery. Then, the situation remains stable during the first year [19]. According to Asfar et al., reduction in implanted segments thickness over time is to be expected [34]. Yakut et al. confirmed a remodeling during the first month after surgery, with a stabilization afterwards [33]. When putting side by side the studies with and without corneal cross-linking, no superiority of the cross-linked group can be identified for the selected performance indicators.

A key aspect for CAIRS surgery long-term performance evaluation is the cornea remodeling and segments size during follow-up. Imaging techniques like anterior segment optical coherence tomography (AS-OCT) and corneal topography with Scheimpflug imaging proved to be effective [10,33,34].

Several limitations require consideration. Although methods of analysis and inclusion criteria were specified and documented in a protocol, this protocol was not prospectively registered. Included studies were uncontrolled case series, limiting the overall interpretation of the results. The main limitations of the studied articles are the absence of information on the position of the conus, the number and position of the segments, and the limited follow-up period. This review was limited to a postoperative follow-up period of one year. Most of the studies specified in- and exclusion criteria, but the selection criteria were heterogeneous among studies. Only a limited number of studies reported statistical power, and in several studies the sample size was small. Gaps in reporting at all time intervals were due to methodological heterogeneity among the included studies. All surgeries were performed by a limited number of experienced ophthalmologists. Translation of their excellent results to other surgeons is not yet documented.

It is expected that further research will deal with surgery planning to improve the treatment customization and predictivity. Imaging will then play a central role.

## 5. Conclusions

CAIRS implantation appears to be an effective treatment option for keratoconus, regardless of the technique used for segment preparation or the addition of corneal cross-linking. No approach demonstrated clear clinical superiority over others in the first year after surgery. The choice for the segments preparation and stromal tunnel creation will be dictated by the ophthalmologist’s experience, material availability, and economic considerations. Imaging systems and refined predictive models should lead to future improvements in CAIRS surgery.

## Figures and Tables

**Table 1:** Quality criteria and number of publications by source.

Source [18]	Quality Item	No. of Publications Scored “Yes”
Added by authors	Consecutive patients?	2 [19,20]
Delphi list	Were inclusion criteria specified?	17 [8,10,19,21,22,23,24,25,26,27,28,29,30,31,32,33,34]
Delphi list	Were exclusion criteria specified?	13 [8,10,19,21,23,25,26,27,28,30,31,33,34]
Delphi list	Were point estimates and measures of variability presented for primary outcome measures?	15 [10,19,21,22,23,25,26,27,28,29,30,31,32,33,34]
Considered for Delphi list	Was calculation of statistical power reported?	3 [19,29,34]

**Table 2:** Baseline characteristics of the study population.

Study	Number of Eyes/Patients	Age (Years)	Male Sex, *n* (%)	Keratoconus Stage
Asfar 2024 [34]	34/31	34 (15)	25 (72)	II–IV
Bteich 2023 [22]	52/39	31 (13)	25 (63)	II–IV
Bteich 2023 Ther Refr Surg [21]	4/2	66 (1)	-	II
Bteich 2025 [23]	20/15	-	-	II–IV
Colak 2025 [24]	6/5	36 (30–44)	3 (60)	II–IV
Coscarelli 2024 [8]	3/3	26 (4)	0 (0)	II–IV
Haciagaoglu 2022 [25]	44/32	30 (7)	36 (75)	-
Hayashi 2025 [29]	5/5	36 (13)	1 (20)	II–IV
Jacob 2018 [10]	24/20	≤35	-	I–IV
Jacob 2023 [22]	32/29	-	16 (55)	I–IV
Keskin Perk 2025 [31]	62/49	29 (8)	33 (67)	I–III
Kirgiz 2024 [26]	23/23	29 (7)	18 (78)	I–IV
Mazzotta 2024 [20]	2/2	49 (4)	2 (100)	II and IV
Mechleb 2024 [32]	10/10	-	-	I–IV
Mechleb 2025 [27]	79/71	≥18	-	I–IV
Nacaraglu 2023 [19]	65/49	29 (20–52)	38 (78)	I–IV
Yakut 2025 [33]	35/27	31 (8)	17 (63)	III–IV
Yukecul 2024 [28]	67/47	27 (20–52)	34 (72)	-
Total	567/459	35 (20–67)	248 (72)	I–IV

Data are mean (SD).

**Table 3:** Surgical technique used for CAIRS implantation in the included studies: (1) CAIRS preparation (trephined blade vs. femtosecond laser), (2) CAIRS hydration (dehydrated vs. non-dehydrated), (3) and the use of cross-linking or not. Adapted from Friedrich and colleagues [9].

Study	CAIRS Preparation (Trephined Blade/FS Laser)	CAIRS Hydration (Dehydrated/Non-dehydrated)	CXL (CXL/No CXL)
Asfar 2024 [34]	Trephine	Dehydrated	No CXL
Bteich 2023 [22]	Trephine	Dehydrated	No CXL
Bteich 2023 Ther Refr Surg [21]	FS laser	Dehydrated	No CXL
Bteich 2025 [23]	FS laser	Dehydrated	No CXL
Colak 2025 [24]	Trephine	Non-dehydrated	No CXL (1 case with CXL excluded)
Coscarelli 2024 [8]	Trephine	Dehydrated	No CXL
Haciagaoglu 2022 [25]	Trephine	-	No CXL
Hayashi 2025 [29]	Trephine	-	No CXL
Jacob 2018 [10]	Trephine	Non-dehydrated	CXL intraoperatively
Jacob 2023 [22]	Trephine	Non-dehydrated	CXL intraoperatively
Keskin Perk 2025 [31]	Trephine	-	No CXL
Kirgiz 2024 [26]	Trephine	Dehydrated	No CXL
Mazzotta 2024 [20]	FS laser	Dehydrated	CXL preoperatively
Mechleb 2024 [32]	FS laser	Dehydrated	No CXL
Mechleb 2025 [27]	FS laser	Dehydrated	No CXL
Nacaraglu 2023 [19]	Trephine	-	CXL preoperatively
Yakut 2025 [33]	Trephine	-	No CXL
Yukecul 2024 [28]	Trephine	-	2 subgroups, CXL preoperatively and no CXL

FS = FemtoSecond; CXL = cross-linking.

**Table 4:** Clinical outcome measures during follow-up visits compared with pre-operative values in the total study population.

TOTAL	Postoperative Follow-Up		
Clinical Outcome Measures	1 Month *	6 Months *	1 Year *
Uncorrected visual acuity (logMAR)	−0.45 [−0.59 to −0.31] *	−0.47 [−0.60 to −0.34] *	−0.39 [−0.48 to −0.30] *
Best corrected visual acuity (logMAR)	−0.36 [−0.46 to −0.25] *	−0.35 [−0.45 to −0.26] *	−0.34 [−0.50 to −0.18] *
Pachymetry thinnest point (μm)	−4.45 [−9.48 to 0.59]	3.55 [−2.64 to 9.74]	−0.22 [−12.52 to 12.07]
Pachymetry central point (μm)	−13.93 [−32.42 to 4.56]	2.80 [−17.48 to 23.08]	−9.44 [−25.37 to 6.49]
Maximum keratometry (D)	−3.88 [−6.71 to −1.05] *	−3.67 [−6.57 to −0.76] *	−3.73 [−4.91 to −2.55] *
Mean simulated keratometry (D)	−4.42 [−5.94 to −2.90] *	−4.42 [−6.12 to −2.72] *	−3.59 [−4.35 to −2.84] *
Total higher-order aberrations	−0.43 [−0.74 to −0.13] *	−0.51 [−0.92 to −0.09] *	−0.54 [−0.89 to −0.20] *
Spherical aberration	0.46 [0.23 to 0.69] *	0.08 [−0.28 to 0.43]	0.11 [−0.06 to 0.28]
Vertical coma	−0.73 [−1.32 to −0.13] *	−0.85 [−1.10 to −0.60] *	−0.50 [−1.68 to 0.67]
Horizontal coma	−0.14 [−0.47 to 0.19]	-	−0.16 [−0.55 to 0.23]
Trefoil	0.41 [0.29 to 0.52] *	0.61 [0.20 to 1.02] *	0.24 [0.02 to 0.45] *
Total RMS	−3.88 [−9.49 to 1.72]	−1.53 [−5.13 to 2.07]	−0.26 [−0.65 to 0.13]

Data are mean effect size (95% CI). * Significant decrease or increase in postoperative outcome measures (*p* < 0.05). See Section S1 (pp. 1–36) for graphical presentation, *p* values, heterogeneity, and references.

**Table 5:** Clinical outcome measures during follow-up visits compared with pre-operative values in two subgroups: CAIRS preparation using trephined blade (**A**) vs. femtosecond laser (**B**).

**A**. **TREPHINED** **BLADE**	**Postoperative Follow-Up**		
**Clinical Outcome Measures**	1 Month *	6 Months *	1 Year *
Uncorrected visual acuity (logMAR)	−0.50 [−0.64 to −0.37] *	−0.53 [−0.66 to −0.40] *	−0.39 [−0.50 to −0.29] *
Best corrected visual acuity (logMAR)	−0.37 [−0.49 to −0.24] *	−0.39 [−0.51 to −0.28] *	−0.32 [−0.50 to −0.15] *
Pachymetry thinnest point (μm)	−4.68 [−9.83 to 0.46]	2.13 [−4.57 to 8.84]	−0.09 [−13.75 to 13.57]
Pachymetry central point (μm)	−13.93 [−32.42 to 4.56]	−2.76 [−32.46 to 26.94]	−9.44 [−25.37 to 6.493]
Maximum keratometry (D)	−3.56 [−6.88 to −0.25] *	−3.96 [−7.94 to 0.01]	−3.67 [−4.90 to −2.44] *
Mean simulated keratometry (D)	−4.09 [−5.96 to −2.22] *	−5.27 [−7.31 to −3.22] *	−3.54 [−4.33 to −2.75] *
Total higher-order aberrations	−0.46 [−0.84 to −0.08] *	−1.35 [−3.31 to 0.61]	−0.53 [−1.00 to −0.06] *
Spherical aberration	0.42 [0.37 to 0.46] *	0.27 [0.22 to 0.32] *	0.11 [−0.11 to 0.33]
Vertical coma	−1.12 [−1.48 to −0.76] *	-	−0.22 [−1.98 to 1.55]
Horizontal coma	−0.14 [−0.47 to 0.19]	-	-
Trefoil	0.40 [0.22 to 0.57] *	0.79 [0.72 to 0.86] *	0.20 [−0.09 to 0.49]
Total RMS	−0.14 [−0.28 to 0.00]	0.09 [−0.05 to 0.23]	−0.26 [−0.65 to 0.13]
**B**. **FEMTOSECOND** **LASER**	**Postoperative Follow-Up**		
**Clinical Outcome Measures**	1 Month *	6 Months *	1 Year *
Uncorrected visual acuity (logMAR)	−0.23 [−0.44 to −0.03] *	−0.36 [−0.51 to −0.21] *	−0.37 [−0.53 to −0.21] *
Best corrected visual acuity (logMAR)	−0.30 [−0.42 to −0.18] *	−0.29 [−0.42 to −0.16] *	−0.42 [−0.53 to −0.31] *
Pachymetry thinnest point (μm)	1.00 [−23.59 to 25.59]	5.77 [−21.70 to 33.23]	−0.80 [−28.98 to 27.38]
Pachymetry central point (μm)	-	11.00 [−5.09 to 27.09]	-
Maximum keratometry (D)	−4.34 [−8.50 to −0.19] *	−2.66 [−4.55 to −0.78] *	−4.36 [−8.43 to −0.29] *
Mean simulated keratometry (D)	−6.26 [−11.18 to −1.35] *	−2.81 [−4.76 to −0.85] *	−4.07 [−6.52 to −1.62] *
Total higher-order aberrations	−0.39 [−0.88 to 0.10]	−0.47 [−0.89 to −0.05] *	−0.56 [−1.06 to −0.06] *
Spherical aberration	1.23 [0.04 to 2.41] *	−0.04 [−0.65 to 0.57]	0.10 [−0.17 to 0.37]
Vertical coma	−0.42 [−1.36 to 0.51]	−0.85 [−1.10 to −0.60] *	−1.09 [−1.73 to −0.45] *
Horizontal coma	-	-	-
Trefoil	0.36 [0.05 to 0.67] *	0.37 [0.05 to 0.69] *	0.28 [−0.04 to 0.59]
Total RMS	−6.76 [−11.56 to −1.96] *	−3.61 [−6.16 to −1.06] *	-

Data are mean effect size (95% CI). * Significant decrease or increase in postoperative outcome measures (*p* < 0.05). See Section S1 (pp. 37–102) for graphical presentation, *p* values, heterogeneity, and references.

**Table 6:** Clinical outcome measures during follow-up visits compared with pre-operative values in two subgroups: non-dehydrated (**A**) vs. dehydrated (**B**) CAIRS.

**A**. **NON-DEHYDRATED**	**Postoperative Follow-Up**		
**Clinical Outcome Measures**	1 Month *	6 Months *	1 Year *
Uncorrected visual acuity (logMAR)	-	−0.72 [−1.25 to −0.19] *	−0.29 [−0.40 to −0.19] *
Best corrected visual acuity (logMAR)	-	−0.13 [−0.32 to 0.06]	−0.09 [−0.15 to −0.03] *
Pachymetry thinnest point (μm)	-	-	6.06 [−14.11 to 26.23]
Pachymetry central point (μm)	-	35.25 [−25.72 to 96.22]	-
Maximum keratometry (D)	-	−0.67 [−5.49 to 4.15]	−3.95 [−6.47 to −1.43] *
Mean simulated keratometry (D)	-	−3.17 [−7.82 to 1.48]	−3.44 [−4.90 to −1.98] *
Total higher-order aberrations	-	-	-
Spherical aberration	-	-	-
Vertical coma	-	-	0.69 [0.15 to 1.23] *
Horizontal coma	-	-	-
Trefoil	-	-	-
Total RMS	-	-	−0.26 [−0.65 to 0.13]
**B**. **DEHYDRATED**	**Postoperative Follow-Up**		
**Clinical Outcome Measures**	1 Month *	6 Months *	1 Year *
Uncorrected visual acuity (logMAR)	−0.42 [−0.72 to −0.12] *	−0.43 [−0.70 to −0.17] *	−0.33 [−0.44 to −0.22] *
Best corrected visual acuity (logMAR)	−0.30 [−0.36 to −0.23] *	−0.33 [−0.45 to −0.20] *	−0.39 [−0.46 to −0.31] *
Pachymetry thinnest point (μm)	−5.62 [−12.79 to 1.56]	6.05 [−1.93 to 14.03]	−0.80 [−28.98 to 27.38]
Pachymetry central point (μm)	-	11.00 [−5.09 to 27.09]	-
Maximum keratometry (D)	−5.96 [−9.82 to −2.11] *	−5.03 [−10.55 to 0.50] *	−4.84 [−7.41 to −2.27] *
Mean simulated keratometry (D)	−5.53 [−8.06 to −3.00] *	−5.45 [−8.63 to −2.28] *	−3.53 [−4.92 to −2.13] *
Total higher-order aberrations	−0.43 [−0.74 to −0.13] *	−0.47 [−0.89 to −0.05] *	−0.54 [−0.89 to −0.20] *
Spherical aberration	0.46 [0.23 to 0.69] *	0.08 [−0.28 to 0.43]	0.11 [−0.06 to 0.28]
Vertical coma	−0.73 [−1.32 to −0.13] *	−0.85 [−1.10 to −0.60] *	−1.10 [−1.45 to −0.76] *
Horizontal coma	-	-	-
Trefoil	0.41 [0.29 to 0.52] *	0.61 [0.20 to 1.02] *	0.24 [0.02 to 0.45] *
Total RMS	−3.88 [−9.49 to 1.72]	−1.53 [−5.13 to 2.07]	-

Data are mean effect size (95% CI). * Significant decrease or increase in postoperative outcome measures (*p* < 0.05). See Section S1 (pp. 103–144) for graphical presentation, *p* values, heterogeneity, and references.

**Table 7:** Clinical outcome measures during follow-up visits compared with pre-operative values in two subgroups: CAIRS with cross-linking (CXL) (**A**) vs. CAIRS without cross-linking (**B**).

**A**. **CXL**	**Postoperative Follow-Up**		
**Clinical Outcome Measures**	1 Month *	6 Months *	1 Year *
Uncorrected visual acuity (logMAR)	−0.44 [−0.63 to −0.24] *	−0.45 [−0.61 to −0.29] *	−0.37 [−0.55 to −0.19] *
Best corrected visual acuity (logMAR)	−0.45 [−0.76 to −0.14] *	−0.43 [−0.70 to −0.16] *	−0.24 [−0.51 to 0.03]
Pachymetry thinnest point (μm)	12.00 [−8.27 to 32.27]	15.32 [−4.45 to 35.10]	6.06 [−14.11 to 26.23]
Pachymetry central point (μm)	−13.93 [−32.42 to 4.56]	−16.16 [−37.06 to 4.74]	−16.31 [−38.33 to 5.71]
Maximum keratometry (D)	−2.21 [−4.44 to 0.02]	−3.31 [−5.48 to −1.14] *	−3.78 [−5.63 to −1.92] *
Mean simulated keratometry (D)	−3.37 [−5.00 to −1.75] *	−3.40 [−5.00 to −1.80] *	−3.61 [−4.80 to −2.43] *
Total higher-order aberrations	-	-	-
Spherical aberration	-	-	-
Vertical coma	-	-	0.69 [0.15 to 1.23]
Horizontal coma	-	-	-
Trefoil	-	-	-
Total RMS	-	-	−0.26 [−0.65 to 0.13]
**B**. **No CXL**	**Postoperative Follow-Up**		
**Clinical Outcome Measures**	1 Month *	6 Months *	1 Year *
Uncorrected visual acuity (logMAR)	−0.45 [−0.62 to −0.29] *	−0.48 [−0.63 to −0.33] *	−0.40 [−0.50 to −0.30] *
Best corrected visual acuity (logMAR)	−0.32 [−0.37 to −0.26] *	−0.33 [−0.41 to −0.25] *	−0.41 [−0.46 to −0.35] *
Pachymetry thinnest point (μm)	−5.53 [−10.72 to −0.33] *	2.27 [−4.25 to 8.79]	−3.94 [−19.44 to 11.57]
Pachymetry central point (μm)	-	12.42 [−2.79 to 27.64]	−1.91 [−24.97 to 21.15]
Maximum keratometry (D)	−4.36 [−7.62 to −1.10] *	−3.68 [−7.16 to −0.21] *	−3.70 [−5.22 to −2.17] *
Mean simulated keratometry (D)	−4.69 [−6.47 to −2.92] *	−4.66 [−6.64 to −2.69] *	−3.58 [−4.55 to −2.61] *
Total higher-order aberrations	−0.43 [−0.74 to −0.13] *	−0.51 [−0.92 to −0.09] *	−0.54 [−0.89 to −0.20] *
Spherical aberration	0.46 [0.23 to 0.69] *	0.08 [−0.28 to 0.43]	0.11 [−0.06 to 0.28]
Vertical coma	−0.73 [−1.32 to −0.13] *	−0.85 [−1.10 to −0.60] *	−1.10 [−1.45 to −0.76] *
Horizontal coma	-	-	-
Trefoil	0.41 [0.29 to 0.52] *	0.61 [0.20 to 1.02] *	0.24 [0.02 to 0.45] *
Total RMS	−3.88 [−9.49 to 1.72]	−1.53 [−5.13 to 2.07]	-

Data are mean effect size (95% CI). * Significant decrease or increase in postoperative outcome measures (*p* < 0.05). See Section S1 (pp. 145–195) for graphical presentation, *p* values, heterogeneity, and references.

## Data Availability

Data sharing is not applicable to this article as no new data were created or analyzed in this study.

## Supplementary Materials

The following supporting information can be downloaded at: https://www.mdpi.com/article/10.3390/medicina62030523/s1, Section S1: Meta-analysis—TOTAL GROUP, Section S2: Characteristics of included studies; Section S3: Search strategy; Section S4: PRISMA 2020 checklist [42].
