# Supplementary material for: Corneal Allogeneic Intrastromal Ring Segments for Treating Keratoconus—Systematic Review and Meta-Analysis
Source: Medicina (Kaunas). 2026 Mar 12;62(3):523. doi: 10.3390/medicina62030523 (PMC13027829; doi:10.3390/medicina62030523)
Supplement: Supplementary file 1 [file medicina-62-00523-s001.zip › medicina-4104982-supplementary.pdf]

Section S1.1. Meta-analysis – TOTAL GROUP

S.1. 1 month postoperative versus preoperative

Figure S1.1.1.1. Difference in uncorrected visual acuity across 7 studies [1-7]

	1 month post-op			pre-op				Mean Difference
**Study or Subgroup**	**Mean**	**SD**	**Total**	**Mean**	**SD**	**Total**	**Weight**	**IV, Random, 95% CI**
Bteich 2023	0.64	0.23	52	0.97	0.47	52	13.5%	-0.33 [-0.47, -0.19]
Bteich 2024	0.66	0.33	20	0.893	0.33	20	11.7%	-0.23 [-0.44, -0.03]
Haciagaoglu 2022	0.34	0.26	44	0.72	0.63	44	11.8%	-0.38 [-0.58, -0.18]
Keskin Perk 2025	0.4	0.34	62	0.96	0.5	62	13.2%	-0.56 [-0.71, -0.41]
Kirgiz 2024	0.432	0.03	23	1.096	0.05	23	15.6%	-0.66 [-0.69, -0.64]
Nacaroglu 2023	0.39	0.28	49	0.91	0.5	49	12.9%	-0.52 [-0.68, -0.36]
Yukecul 2024 (non-CXL)	0.26	0.22	37	0.77	0.76	37	10.2%	-0.51 [-0.76, -0.26]
Yukecul 2024 (prior CXL)	0.38	0.29	30	0.7	0.55	30	11.1%	-0.32 [-0.54, -0.10]
**Total (95% CI)**			**317**			**317**	**100.0%**	**-0.45 [-0.59, -0.31]**

Heterogeneity: Tau² = 0.03; Chi² = 55.87, df = 7 (P < 0.00001); I² = 87%

Test for overall effect: Z = 6.40 (P < 0.00001)

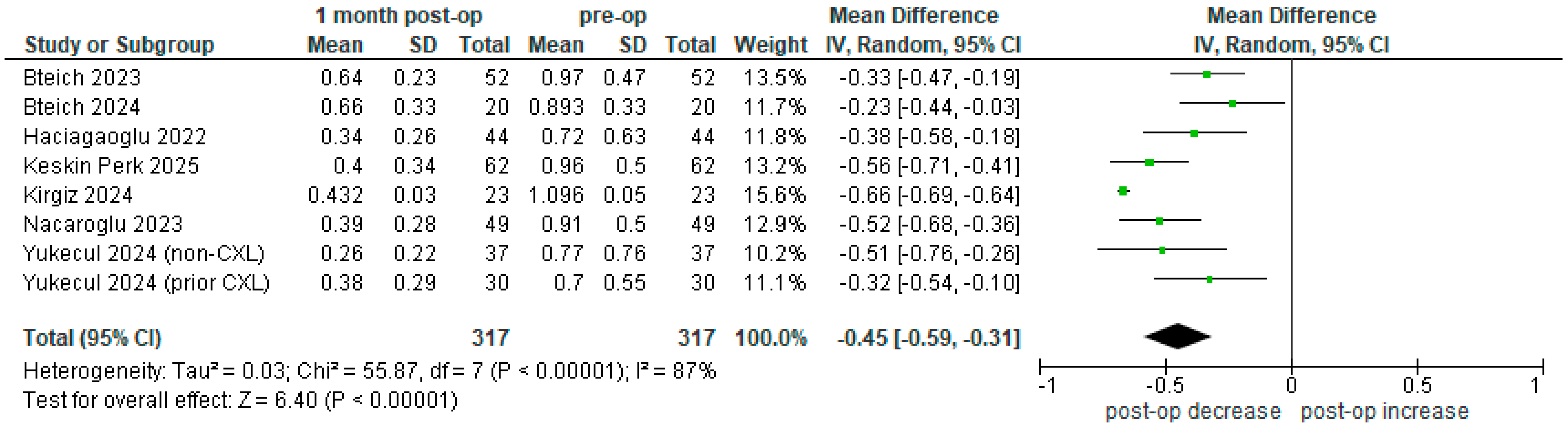

Figure S1.1.1.2. Difference in best corrected visual acuity across 8 studies [1-8]

Study or Subgroup	1 month post-op Mean	SD	Total	pre-op Mean	SD	Total	Weight	Mean Difference IV, Random, 95% CI
Bteich 2023	0.31	0.21	52	0.56	0.19	52	11.8%	-0.25 [-0.33, -0.17]
Bteich 2024	0.32	0.2	20	0.57	0.18	20	10.9%	-0.25 [-0.37, -0.13]
Haciagaoglu 2022	0.24	0.19	44	0.54	0.37	44	10.8%	-0.30 [-0.42, -0.18]
Keskin Perk 2025	0.28	0.22	62	0.72	0.47	62	10.6%	-0.44 [-0.57, -0.31]
Kirgiz 2024	0.237	0.06	23	0.602	0.3	23	10.7%	-0.36 [-0.49, -0.24]
Mechleb 2024 (Kmax inf 75D)	0.03	0.02	6	0.36	0.12	6	11.4%	-0.33 [-0.43, -0.23]
Mechleb 2024 (Kmax sup 75D)	1.03	0.88	4	2.45	1.1	4	0.5%	-1.42 [-2.80, -0.04]
Nacaroglu 2023	0.27	0.06	49	0.87	0.2	49	12.2%	-0.60 [-0.66, -0.54]
Yukecul 2024 (non-CXL)	0.19	0.15	37	0.51	0.29	37	11.2%	-0.32 [-0.43, -0.21]
Yukecul 2024 (prior CXL)	0.27	0.2	30	0.55	0.39	30	9.8%	-0.28 [-0.44, -0.12]
**Total (95% CI)**			**327**			**327**	**100.0%**	**-0.36 [-0.46, -0.25]**

Heterogeneity: Tau² = 0.02; Chi² = 77.49, df = 9 (P < 0.00001); I² = 88%
Test for overall effect: Z = 6.72 (P < 0.00001)

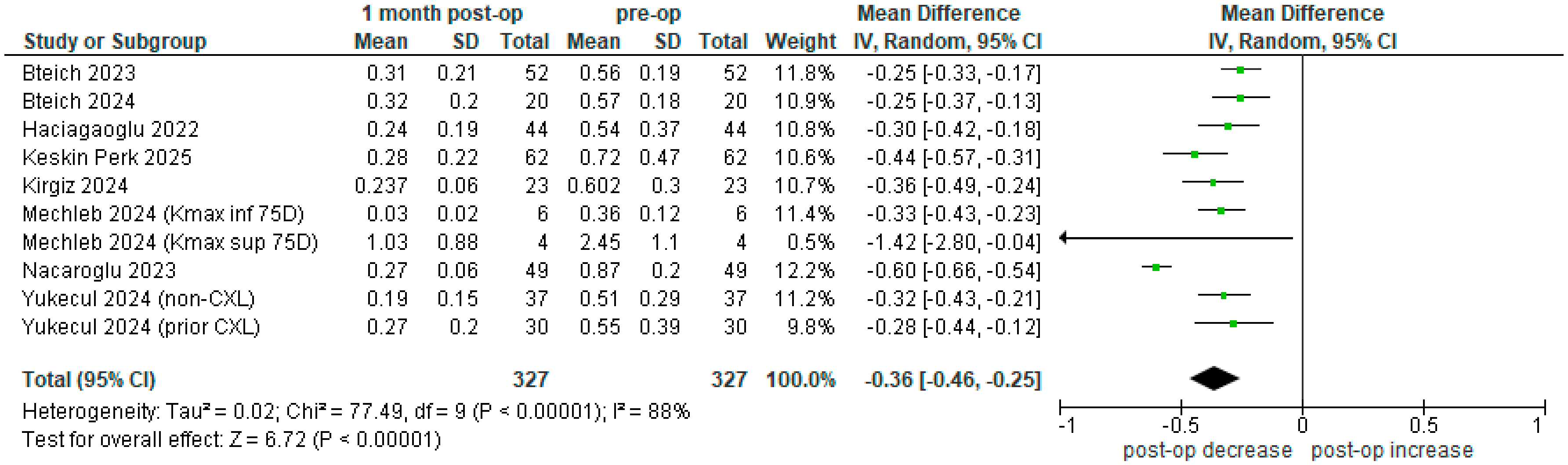

Figure S1.1.1.3. Difference in pachymetry thinnest point across 6 studies [1-5, 7]

	1 month post-op			pre-op				Mean Difference
**Study or Subgroup**	**Mean**	**SD**	**Total**	**Mean**	**SD**	**Total**	**Weight**	**IV, Random, 95% CI**
Bteich 2023	429.7	55.5	52	422.5	55.2	52	5.6%	7.20 [-14.08, 28.48]
Bteich 2024	443.8	41.2	20	442.8	38.1	20	4.2%	1.00 [-23.59, 25.59]
Haciagaoglu 2022	449.2	57.41	44	453.73	45.87	44	5.4%	-4.53 [-26.24, 17.18]
Keskin Perk 2025	446.32	40.26	62	449.68	49.48	62	10.0%	-3.36 [-19.24, 12.52]
Kirgiz 2024	431.33	11.78	23	439.33	10.43	23	61.3%	-8.00 [-14.43, -1.57]
Yukecul 2024 (non-CXL)	452	36	37	454	45	37	7.3%	-2.00 [-20.57, 16.57]
Yukecul 2024 (prior CXL)	449	42	30	437	38	30	6.2%	12.00 [-8.27, 32.27]
**Total (95% CI)**			**268**			**268**	**100.0%**	**-4.45 [-9.48, 0.59]**

Heterogeneity: Tau² = 0.00; Chi² = 5.13, df = 6 (P = 0.53); I² = 0%
Test for overall effect: Z = 1.73 (P = 0.08)

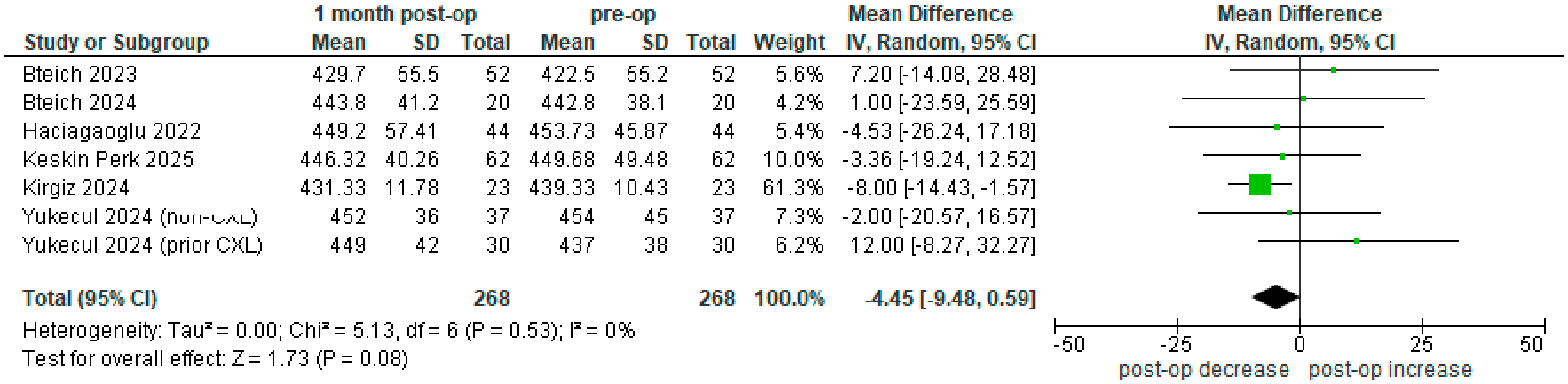

Figure S1.1.1.4. Difference in pachymetry central point across 1 study [6]

	1 month post-op			pre-op				Mean Difference
**Study or Subgroup**	**Mean**	**SD**	**Total**	**Mean**	**SD**	**Total**	**Weight**	**IV, Random, 95% CI**
Nacaroglu 2023	449.69	36.72	49	463.62	54.88	49	100.0%	-13.93 [-32.42, 4.56]
**Total (95% CI)**			**49**			**49**	**100.0%**	**-13.93 [-32.42, 4.56]**
Heterogeneity: Not applicable								
Test for overall effect: Z = 1.48 (P = 0.14)								

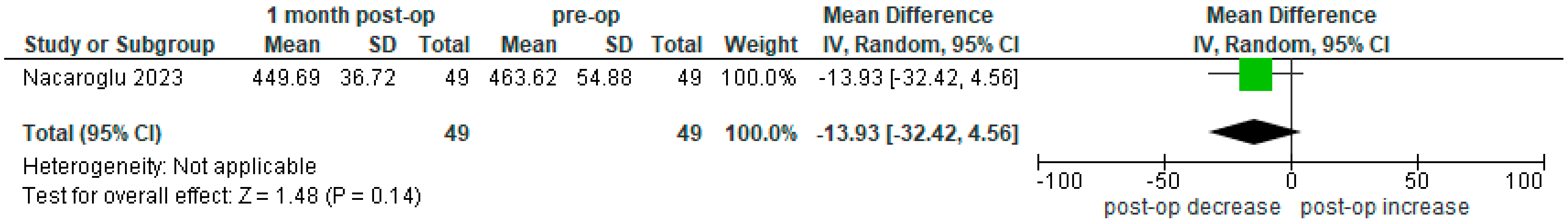

Figure S1.1.1.5. Difference in maximum keratometry across 8 studies [1-8]

	1 month post-op			pre-op				Mean Difference
**Study or Subgroup**	**Mean**	**SD**	**Total**	**Mean**	**SD**	**Total**	**Weight**	**IV, Random, 95% CI**
Bteich 2023	54.17	8.22	52	58.09	7.92	52	11.0%	-3.92 [-7.02, -0.82]
Bteich 2024	53.3	6.22	20	57.21	7.53	20	9.8%	-3.91 [-8.19, 0.37]
Haciagaoglu 2022	55.46	6.97	44	57.56	7.06	44	11.1%	-2.10 [-5.03, 0.83]
Keskin Perk 2025	56.34	7.43	62	58.51	7.55	62	11.4%	-2.17 [-4.81, 0.47]
Kirgiz 2024	48.18	2.03	23	57.65	1.41	23	12.4%	-9.47 [-10.48, -8.46]
Mechleb 2024 (Kmax inf 75D)	51.93	5.79	6	54.43	4.09	6	8.4%	-2.50 [-8.17, 3.17]
Mechleb 2024 (Kmax sup 75D)	67.02	10.95	4	81.5	7.95	4	3.3%	-14.48 [-27.74, -1.22]
Nacaroglu 2023	56.75	7.72	49	59.2	7.66	49	11.0%	-2.45 [-5.50, 0.60]
Yukecul 2024 (non-CXL)	56.17	7.13	37	58.38	7.24	37	10.8%	-2.21 [-5.48, 1.06]
Yukecul 2024 (prior CXL)	55.28	6.26	30	57.22	6.68	30	10.8%	-1.94 [-5.22, 1.34]
**Total (95% CI)**			**327**			**327**	**100.0%**	**-3.88 [-6.71, -1.05]**

Heterogeneity: $Tau^2 = 16.46$; $Chi^2 = 80.42$, $df = 9$ ($P < 0.00001$); $I^2 = 89\%$
Test for overall effect: $Z = 2.69$ ($P = 0.007$)

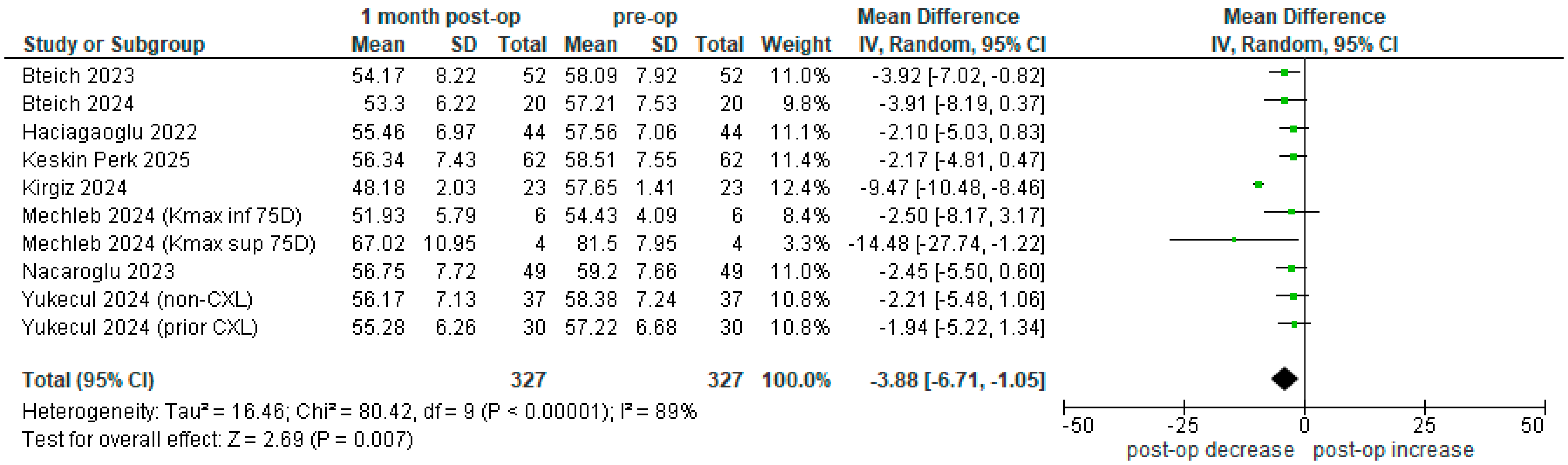

Figure S1.1.1.6. Difference in mean simulated keratometry across 8 studies [1-8]

Study or Subgroup	1 month post-op Mean	SD	Total	pre-op Mean	SD	Total	Weight	Mean Difference IV, Random, 95% CI
Bteich 2023	45.66	5.04	52	48.92	4.49	52	11.4%	-3.26 [-5.09, -1.43]
Bteich 2024	46.28	3.62	20	49.3	4.83	20	9.7%	-3.02 [-5.67, -0.37]
Haciagaoglu 2022	45.72	4.85	44	49.01	5.11	44	10.9%	-3.29 [-5.37, -1.21]
Keskin Perk 2025	46.22	4.98	62	49.17	4.97	62	11.6%	-2.95 [-4.70, -1.20]
Kirgiz 2024	42.91	1.32	23	49.86	1.16	23	13.3%	-6.95 [-7.67, -6.23]
Mechleb 2024 (Kmax inf 75D)	44.925	3.91	6	49.665	3.16	6	7.0%	-4.74 [-8.76, -0.72]
Mechleb 2024 (Kmax sup 75D)	55.325	5.36	4	68.01	2.215	4	4.7%	-12.69 [-18.37, -7.00]
Nacaroglu 2023	46.32	5.35	49	49.34	5.32	49	10.8%	-3.02 [-5.13, -0.91]
Yukecul 2024 (non-CXL)	44.52	4.8	37	49.11	4.66	37	10.7%	-4.59 [-6.75, -2.43]
Yukecul 2024 (prior CXL)	45.58	4.45	30	49.46	5.55	30	9.9%	-3.88 [-6.43, -1.33]
**Total (95% CI)**			**327**			**327**	**100.0%**	**-4.42 [-5.94, -2.90]**

Heterogeneity: Tau² = 4.41; Chi² = 50.90, df = 9 (P < 0.00001); I² = 82%

Test for overall effect: Z = 5.69 (P < 0.00001)

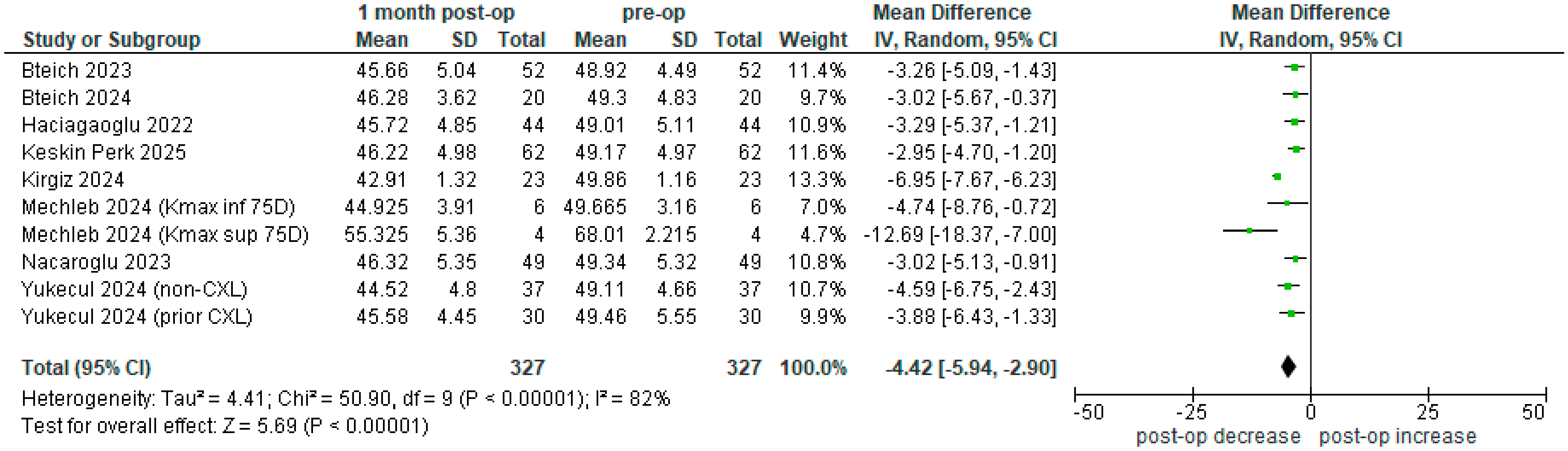

Figure S1.1.1.7. Difference in total higher order aberrations across 2 studies [1, 2]

	1 month post-op			pre-op				Mean Difference
**Study or Subgroup**	**Mean**	**SD**	**Total**	**Mean**	**SD**	**Total**	**Weight**	**IV, Random, 95% CI**
Bteich 2023	1.74	0.85	52	2.2	1.13	52	61.9%	-0.46 [-0.84, -0.08]
Bteich 2024	1.53	0.59	20	1.92	0.95	20	38.1%	-0.39 [-0.88, 0.10]
**Total (95% CI)**			**72**			**72**	**100.0%**	**-0.43 [-0.74, -0.13]**

Heterogeneity: Tau² = 0.00; Chi² = 0.05, df = 1 (P = 0.83); I² = 0%
Test for overall effect: Z = 2.81 (P = 0.005)

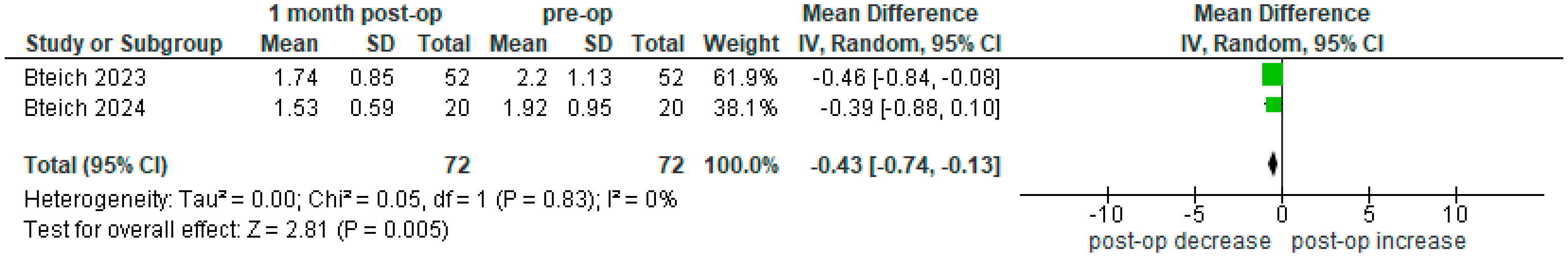

Figure S1.1.1.8. Difference in spherical aberration across 4 studies [1, 2, 5, 8]

	1 month post-op			pre-op				Mean Difference
**Study or Subgroup**	**Mean**	**SD**	**Total**	**Mean**	**SD**	**Total**	**Weight**	**IV, Random, 95% CI**
Bteich 2023	0.51	0.59	52	0.15	0.45	52	28.7%	0.36 [0.16, 0.56]
Bteich 2024	0.31	0.39	20	0.05	0.42	20	25.6%	0.26 [0.01, 0.51]
Kirgiz 2024	0.8	0.1	23	0.38	0.07	23	36.3%	0.42 [0.37, 0.47]
Mechleb 2024 (Kmax inf 75D)	0.13	0.5	6	-0.94	0.75	6	7.9%	1.07 [0.35, 1.79]
Mechleb 2024 (Kmax sup 75D)	-1.35	0.69	4	-4.76	1.73	4	1.5%	3.41 [1.58, 5.24]
**Total (95% CI)**			**105**			**105**	**100.0%**	**0.46 [0.23, 0.69]**

Heterogeneity: $Tau^2 = 0.04$; $Chi^2 = 15.30$, df = 4 (P = 0.004); $I^2 = 74\%$
Test for overall effect: Z = 3.92 (P < 0.0001)

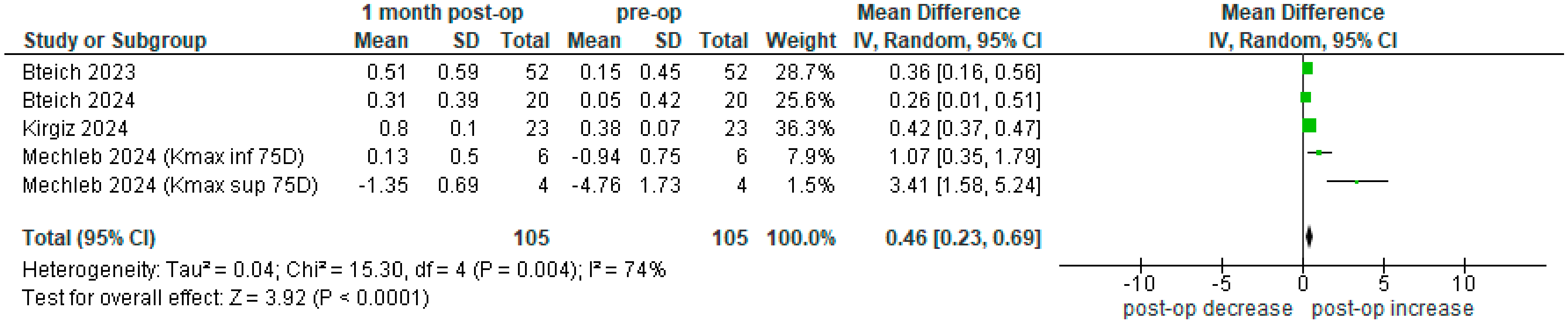

Figure S1.1.1.9. Difference in vertical coma across 3 studies [1, 2, 8]

	1 month post-op			pre-op				Mean Difference
**Study or Subgroup**	**Mean**	**SD**	**Total**	**Mean**	**SD**	**Total**	**Weight**	**IV, Random, 95% CI**
Bteich 2023	0.44	0.76	52	1.56	1.09	52	38.3%	-1.12 [-1.48, -0.76]
Bteich 2024	0.6	0.88	20	1.44	1.09	20	30.2%	-0.84 [-1.45, -0.23]
Mechleb 2024 (Kmax inf 75D)	1.16	1.19	6	2.03	0.52	6	18.9%	-0.87 [-1.91, 0.17]
Mechleb 2024 (Kmax sup 75D)	3.2	1.33	4	2.25	0.59	4	12.6%	0.95 [-0.48, 2.38]
**Total (95% CI)**			**82**			**82**	**100.0%**	**-0.73 [-1.32, -0.13]**

Heterogeneity: $Tau^2 = 0.21$; $Chi^2 = 7.82$, df = 3 (P = 0.05); $I^2 = 62\%$

Test for overall effect: Z = 2.39 (P = 0.02)

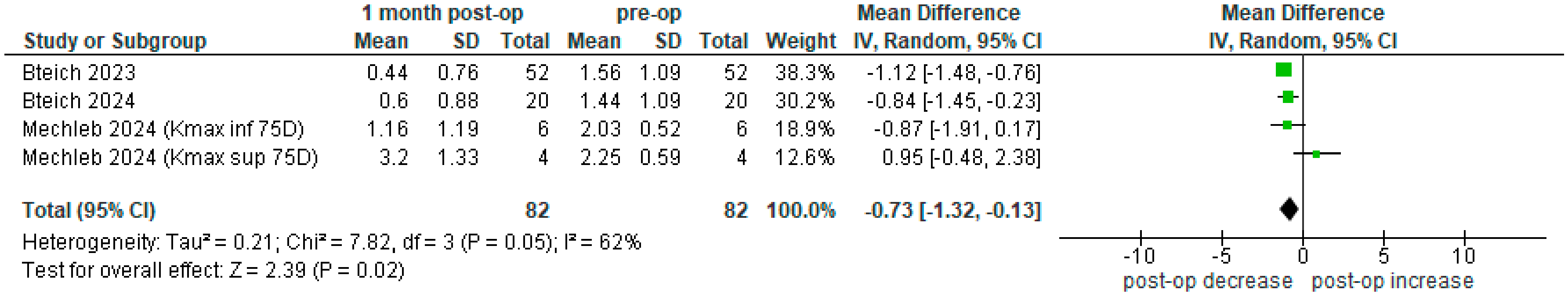

Figure S1.1.1.10. Difference in horizontal coma across 1 study [1]

	1 month post-op			pre-op				Mean Difference	Mean Difference
**Study or Subgroup**	**Mean**	**SD**	**Total**	**Mean**	**SD**	**Total**	**Weight**	**IV, Random, 95% CI**	**IV, Random, 95% CI**
Bteich 2023	-0.03	0.73	52	0.11	0.97	52	100.0%	-0.14 [-0.47, 0.19]	
**Total (95% CI)**			**52**			**52**	**100.0%**	**-0.14 [-0.47, 0.19]**	

Heterogeneity: Not applicable
Test for overall effect: Z = 0.83 (P = 0.41)

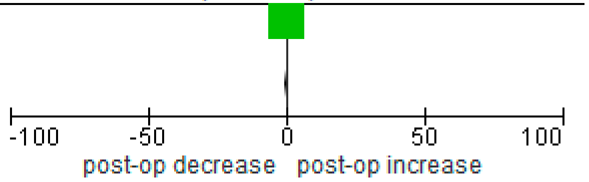

Figure S1.1.1.11. Difference in trefoil across 3 studies [1, 2, 5]

	1 month post-op			pre-op				Mean Difference
**Study or Subgroup**	**Mean**	**SD**	**Total**	**Mean**	**SD**	**Total**	**Weight**	**IV, Random, 95% CI**
Bteich 2023	-0.12	0.64	52	-0.39	0.52	52	20.3%	0.27 [0.05, 0.49]
Bteich 2024	0.088	0.59	20	-0.27	0.4	20	11.7%	0.36 [0.05, 0.67]
Kirgiz 2024	1.12	0.1	23	0.66	0.13	23	68.0%	0.46 [0.39, 0.53]
**Total (95% CI)**			**95**			**95**	**100.0%**	**0.41 [0.29, 0.52]**

Heterogeneity: Tau² = 0.00; Chi² = 2.82, df = 2 (P = 0.24); I² = 29%
Test for overall effect: Z = 6.99 (P < 0.00001)

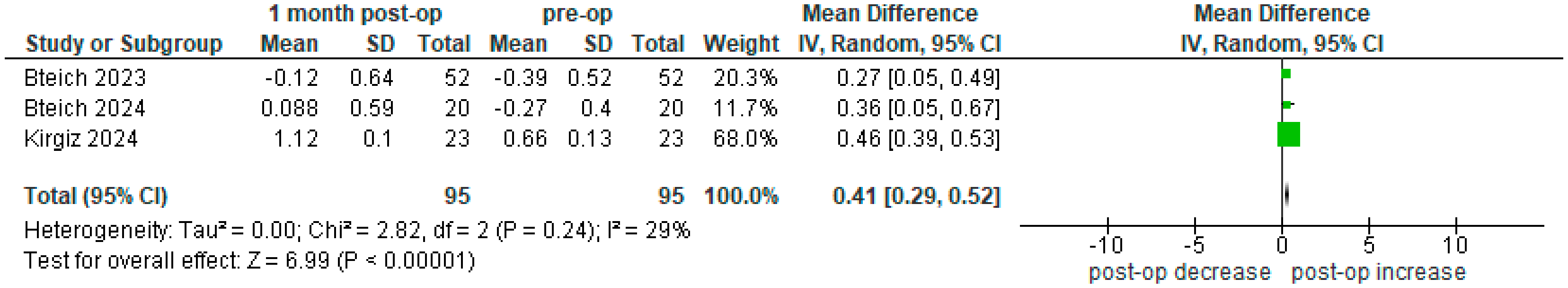

Figure S1.1.1.12. Difference in total RMS across 3 studies [5, 8]

	1 month post-op			pre-op				Mean Difference
**Study or Subgroup**	**Mean**	**SD**	**Total**	**Mean**	**SD**	**Total**	**Weight**	**IV, Random, 95% CI**
Kirgiz 2024	2.21	0.15	23	2.35	0.3	23	46.9%	-0.14 [-0.28, -0.00]
Mechleb 2024 (Kmax inf 75D)	8.45	5.87	6	14.1	4.04	6	31.6%	-5.65 [-11.35, 0.05]
Mechleb 2024 (Kmax sup 75D)	20.33	4.94	4	29.78	7.59	4	21.5%	-9.45 [-18.32, -0.58]
**Total (95% CI)**			**33**			**33**	**100.0%**	**-3.88 [-9.49, 1.72]**

Heterogeneity: Tau² = 17.41; Chi² = 7.81, df = 2 (P = 0.02); I² = 74%

Test for overall effect: Z = 1.36 (P = 0.17)

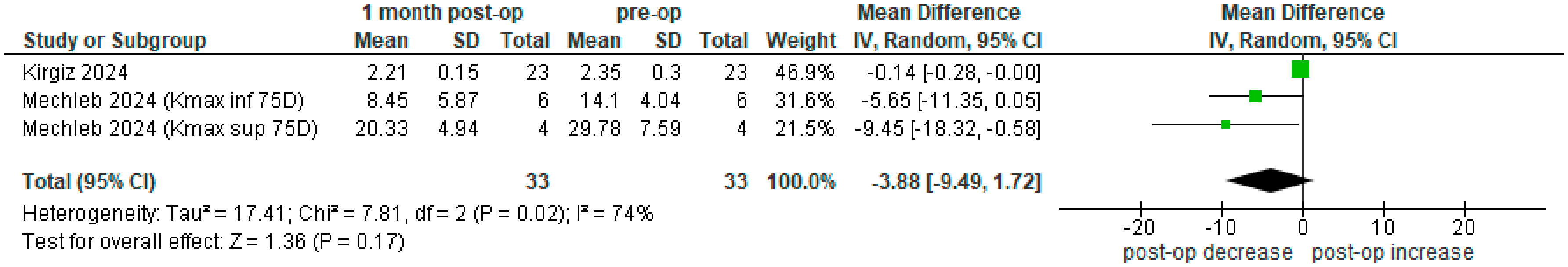

S1.1.2. 6 months postoperative versus preoperative

Figure S1.1.2.1. Difference in uncorrected visual acuity across 11 studies [1-7, 9-12]

	6 months post-op			pre-op				Mean Difference
**Study or Subgroup**	**Mean**	**SD**	**Total**	**Mean**	**SD**	**Total**	**Weight**	**IV, Random, 95% CI**
Mazzotta 2024	0.52	0	2	1	0	2		Not estimable
Hayashi 2025	0.68	0.54	5	1.39	0.28	5	4.1%	-0.71 [-1.24, -0.18]
Colak 2025	0.48	0.21	4	1.2	0.5	4	4.1%	-0.72 [-1.25, -0.19]
Bteich 2023	0.68	0.23	4	0.93	0.31	4	6.3%	-0.25 [-0.63, 0.13]
Bteich 2023 ther refr	0.68	0.23	4	0.93	0.31	4	6.3%	-0.25 [-0.63, 0.13]
Yukecul 2024 (non-CXL)	0.31	0.21	37	0.77	0.76	37	9.1%	-0.46 [-0.71, -0.21]
Yukecul 2024 (prior CXL)	0.36	0.33	30	0.7	0.55	30	9.8%	-0.34 [-0.57, -0.11]
Haciagaoglu 2022	0.35	0.29	44	0.72	0.63	44	10.5%	-0.37 [-0.57, -0.17]
Bteich 2024	0.51	0.18	20	0.893	0.33	20	11.6%	-0.38 [-0.55, -0.22]
Nacaroglu 2023	0.4	0.24	49	0.91	0.5	49	11.9%	-0.51 [-0.67, -0.35]
Keskin Perk 2025	0.44	0.34	62	0.96	0.5	62	12.0%	-0.52 [-0.67, -0.37]
Kirgiz 2024	0.398	0.05	23	1.096	0.05	23	14.3%	-0.70 [-0.73, -0.67]
**Total (95% CI)**			**284**			**284**	**100.0%**	**-0.47 [-0.60, -0.34]**

Heterogeneity: $Tau^2 = 0.03$; $Chi^2 = 51.05$, df = 10 ($P < 0.00001$); $I^2 = 80\%$

Test for overall effect: Z = 7.30 ($P < 0.00001$)

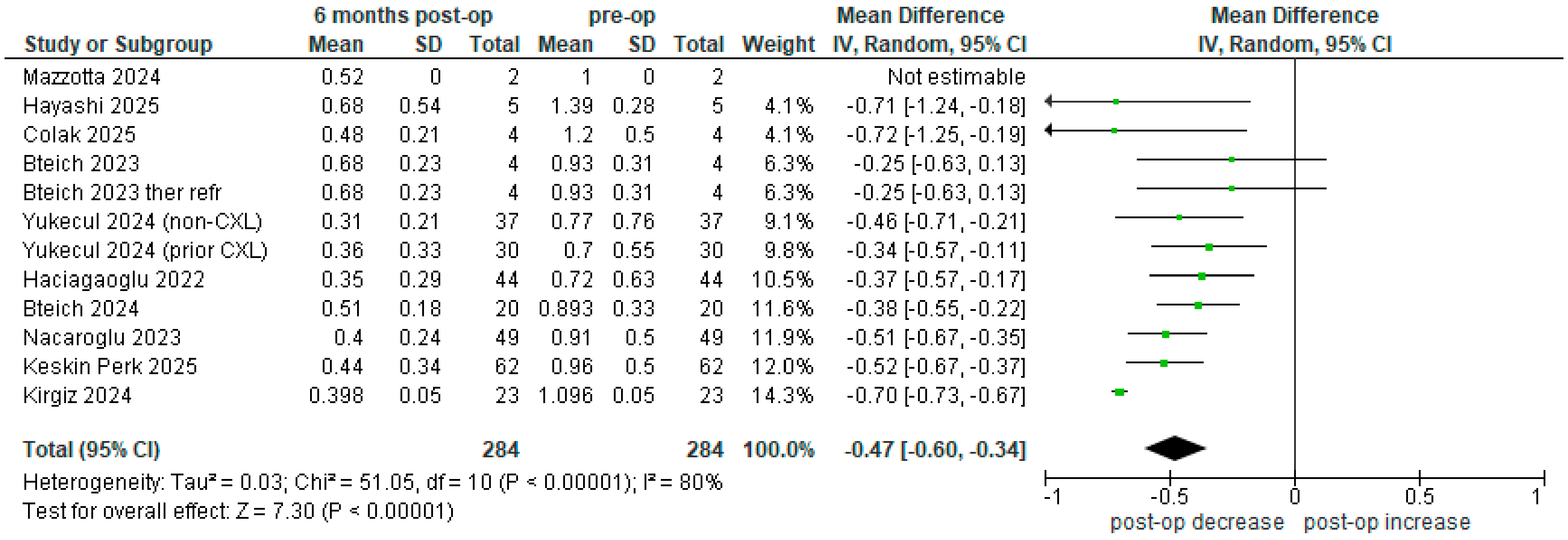

Figure S1.1.2.2. Difference in best corrected visual acuity across 11 studies [2-7, 10-14]

	6 months post-op			pre-op				Mean Difference
**Study or Subgroup**	**Mean**	**SD**	**Total**	**Mean**	**SD**	**Total**	**Weight**	**IV, Random, 95% CI**
Bteich 2023 ther refr	0.01	0.02	4	0.34	0.16	4	8.8%	-0.33 [-0.49, -0.17]
Bteich 2024	0.19	0.21	20	0.57	0.18	20	9.8%	-0.38 [-0.50, -0.26]
Colak 2025	0.4	0.14	4	0.53	0.13	4	8.1%	-0.13 [-0.32, 0.06]
Coscarelli 2024	0.301	0	3	1.06	0.22	3		Not estimable
Haciagaoglu 2022	0.25	0.22	44	0.54	0.37	44	9.7%	-0.29 [-0.42, -0.16]
Hayashi 2025	0.17	0.16	5	0.74	0.44	5	3.7%	-0.57 [-0.98, -0.16]
Keskin Perk 2025	0.25	0.24	62	0.72	0.47	62	9.5%	-0.47 [-0.60, -0.34]
Kirgiz 2024	0.167	0.05	23	0.602	0.3	23	9.7%	-0.43 [-0.56, -0.31]
Mechleb 2025	0.17	0.18	33	0.36	0.25	79	10.7%	-0.19 [-0.27, -0.11]
Nacaroglu 2023	0.31	0.09	49	0.87	0.2	49	11.1%	-0.56 [-0.62, -0.50]
Yukecul 2024 (non-CXL)	0.17	0.18	37	0.51	0.29	37	10.1%	-0.34 [-0.45, -0.23]
Yukecul 2024 (prior CXL)	0.27	0.23	30	0.55	0.39	30	8.7%	-0.28 [-0.44, -0.12]
**Total (95% CI)**			**314**			**360**	**100.0%**	**-0.35 [-0.45, -0.26]**

Heterogeneity: Tau² = 0.02; Chi² = 67.59, df = 10 (P < 0.00001); I² = 85%
Test for overall effect: Z = 7.22 (P < 0.00001)

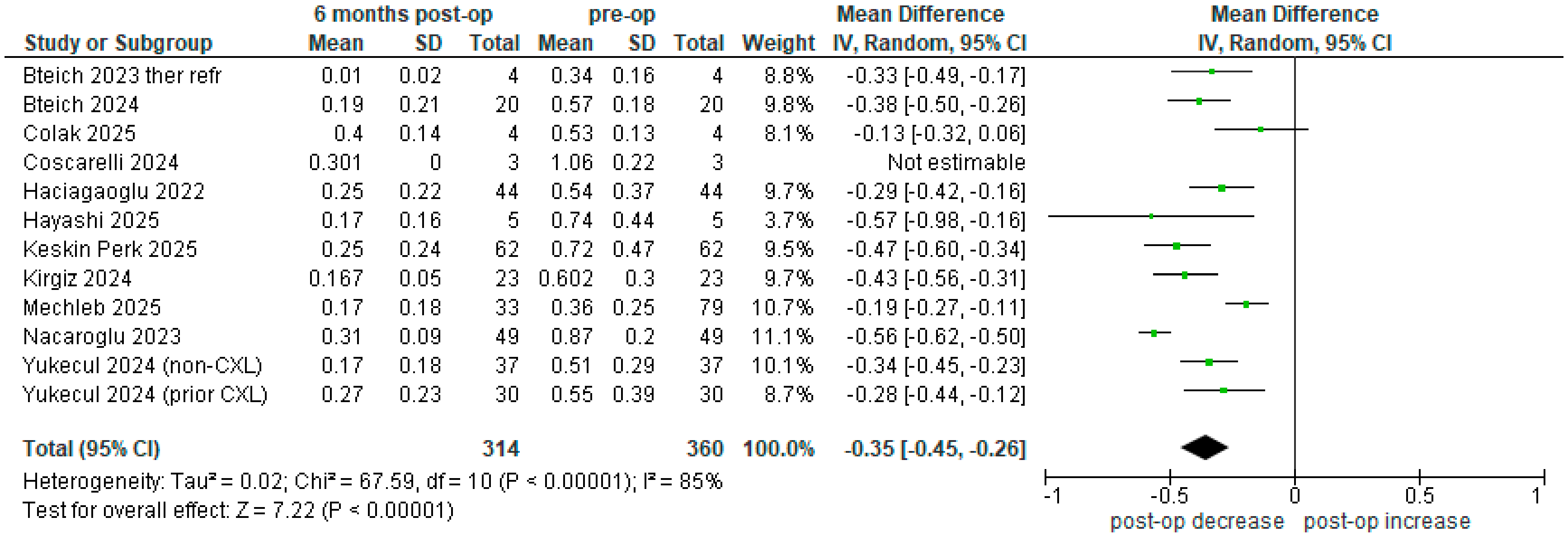

Figure S1.1.2.3. Difference in pachymetry thinnest point in 6 studies [2-5, 7, 9]

	6 months post-op			pre-op				Mean Difference
**Study or Subgroup**	**Mean**	**SD**	**Total**	**Mean**	**SD**	**Total**	**Weight**	**IV, Random, 95% CI**
Bteich 2024	447.5	51.5	20	442.8	38.1	20	4.9%	4.70 [-23.38, 32.78]
Haciagaoglu 2022	449.48	61.06	44	453.73	45.87	44	7.5%	-4.25 [-26.82, 18.32]
Keskin Perk 2025	445.7	47.35	62	449.68	49.48	62	13.2%	-3.98 [-21.03, 13.07]
Kirgiz 2024	445.5	17.5	23	439.33	10.43	23	55.3%	6.17 [-2.16, 14.50]
Mazzotta 2024	432	76.37	2	402.5	57.28	2	0.2%	29.50 [-102.80, 161.80]
Yukecul 2024 (non-CXL)	446	44	37	454	45	37	9.3%	-8.00 [-28.28, 12.28]
Yukecul 2024 (prior CXL)	452	41	30	437	38	30	9.6%	15.00 [-5.00, 35.00]
**Total (95% CI)**			**218**			**218**	**100.0%**	**3.55 [-2.64, 9.74]**

Heterogeneity: Tau² = 0.00; Chi² = 4.25, df = 6 (P = 0.64); I² = 0%
Test for overall effect: Z = 1.12 (P = 0.26)

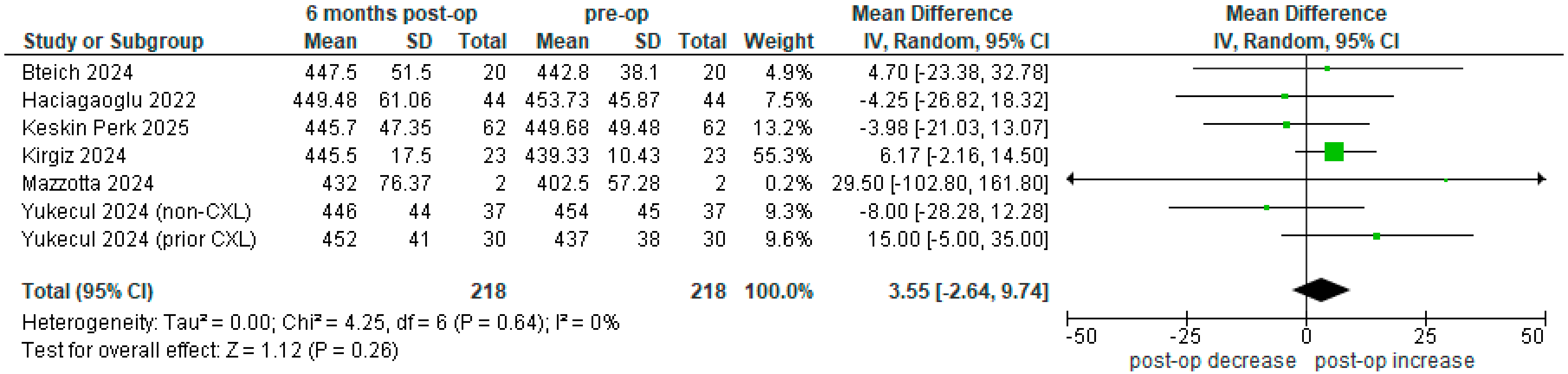

Figure S1.1.2.4. Difference in pachymetry central point in 4 studies [6, 10, 11, 14]

	6 months post-op			pre-op				Mean Difference
**Study or Subgroup**	**Mean**	**SD**	**Total**	**Mean**	**SD**	**Total**	**Weight**	**IV, Random, 95% CI**
Colak 2025	452.5	47.03	4	417.25	40.73	4	9.4%	35.25 [-25.72, 96.22]
Hayashi 2025	514.6	62.63	5	505.6	55.4	5	6.8%	9.00 [-64.29, 82.29]
Mechleb 2025	466	39	33	455	41	79	45.6%	11.00 [-5.09, 27.09]
Nacaroglu 2023	447.46	50.57	49	463.62	54.88	49	38.1%	-16.16 [-37.06, 4.74]
**Total (95% CI)**			**91**			**137**	**100.0%**	**2.80 [-17.48, 23.08]**

Heterogeneity: $Tau^2 = 167.23$; $Chi^2 = 5.27$, $df = 3$ ($P = 0.15$); $I^2 = 43\%$

Test for overall effect: $Z = 0.27$ ($P = 0.79$)

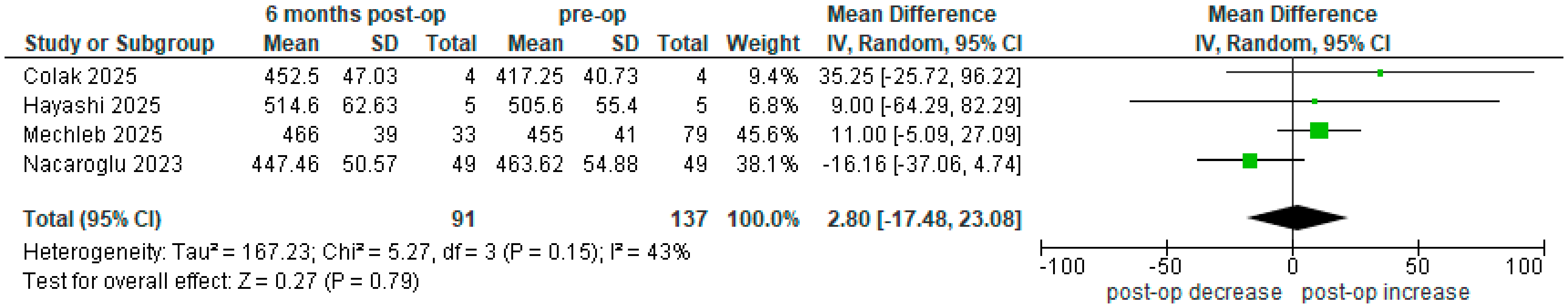

Figure S1.1.2.5. Difference in maximum keratometry across 11 studies [2-7, 9-12, 14]

	6 months post-op			pre-op				Mean Difference
**Study or Subgroup**	**Mean**	**SD**	**Total**	**Mean**	**SD**	**Total**	**Weight**	**IV, Random, 95% CI**
Bteich 2023 ther refr	47.89	3.05	4	50.02	1.99	4	8.9%	-2.13 [-5.70, 1.44]
Bteich 2024	52.38	6.02	20	57.21	7.53	20	8.5%	-4.83 [-9.06, -0.60]
Colak 2025	55.88	3.64	4	56.55	3.31	4	8.0%	-0.67 [-5.49, 4.15]
Haciagaoglu 2022	55.4	6.73	44	57.56	7.06	44	9.4%	-2.16 [-5.04, 0.72]
Hayashi 2025	59.62	6.78	5	63.58	8.26	5	5.0%	-3.96 [-13.33, 5.41]
Keskin Perk 2025	55.27	6.17	62	58.51	7.55	62	9.6%	-3.24 [-5.67, -0.81]
Kirgiz 2024	46.96	1.88	23	57.65	1.41	23	10.2%	-10.69 [-11.65, -9.73]
Mazzotta 2024	50.81	4.53	2	56.21	8.78	2	3.1%	-5.40 [-19.09, 8.29]
Mechleb 2025	55.6	6.4	33	57.6	6.9	79	9.5%	-2.00 [-4.66, 0.66]
Nacaroglu 2023	55.83	6.96	49	59.2	7.66	49	9.4%	-3.37 [-6.27, -0.47]
Yukecul 2024 (non-CXL)	55.66	6.29	37	58.38	7.24	37	9.3%	-2.72 [-5.81, 0.37]
Yukecul 2024 (prior CXL)	54.11	6.65	30	57.22	6.68	30	9.1%	-3.11 [-6.48, 0.26]
**Total (95% CI)**			**313**			**359**	**100.0%**	**-3.67 [-6.57, -0.76]**

Heterogeneity: $Tau^2 = 21.22$; $Chi^2 = 124.07$, df = 11 (P < 0.00001); $I^2 = 91\%$
Test for overall effect: Z = 2.48 (P = 0.01)

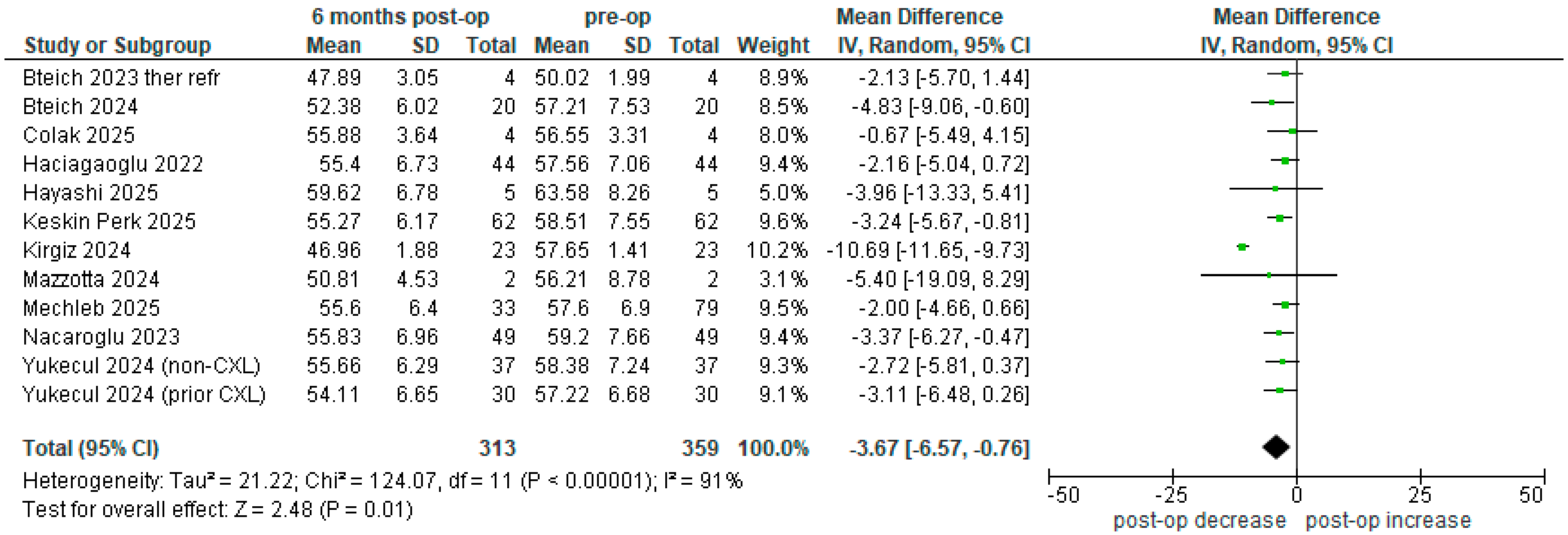

Figure S1.1.2.6. Difference in mean simulated keratometry across 10 studies [2-7, 11-14]

	6 months post-op			pre-op				Mean Difference
**Study or Subgroup**	**Mean**	**SD**	**Total**	**Mean**	**SD**	**Total**	**Weight**	**IV, Random, 95% CI**
Bteich 2023 ther refr	43.18	1.52	4	44.32	0.95	4	9.9%	-1.14 [-2.90, 0.62]
Bteich 2024	45.45	3.57	20	49.3	4.83	20	8.7%	-3.85 [-6.48, -1.22]
Colak 2025	45.88	3.27	4	49.05	3.44	4	6.1%	-3.17 [-7.82, 1.48]
Coscarelli 2024	44.28	0.87	3	55.78	2.84	3	7.7%	-11.50 [-14.86, -8.14]
Haciagaoglu 2022	45.71	4.8	44	49.01	5.11	44	9.5%	-3.30 [-5.37, -1.23]
Keskin Perk 2025	45.75	4.47	62	49.17	4.97	62	10.0%	-3.42 [-5.08, -1.76]
Kirgiz 2024	42.4	1.37	23	49.86	1.16	23	10.8%	-7.46 [-8.19, -6.73]
Mechleb 2025	45.3	5	33	49.2	6.5	79	9.3%	-3.90 [-6.13, -1.67]
Nacaroglu 2023	45.76	5.02	49	49.34	5.32	49	9.5%	-3.58 [-5.63, -1.53]
Yukecul 2024 (non-CXL)	44.45	4.2	37	49.11	4.66	37	9.6%	-4.66 [-6.68, -2.64]
Yukecul 2024 (prior CXL)	46.34	4.46	30	49.46	5.55	30	8.9%	-3.12 [-5.67, -0.57]
**Total (95% CI)**			**309**			**355**	**100.0%**	**-4.42 [-6.12, -2.72]**

Heterogeneity: $Tau^2 = 6.82$; $Chi^2 = 85.47$, $df = 10$ ($P < 0.00001$); $I^2 = 88\%$
Test for overall effect: $Z = 5.09$ ($P < 0.00001$)

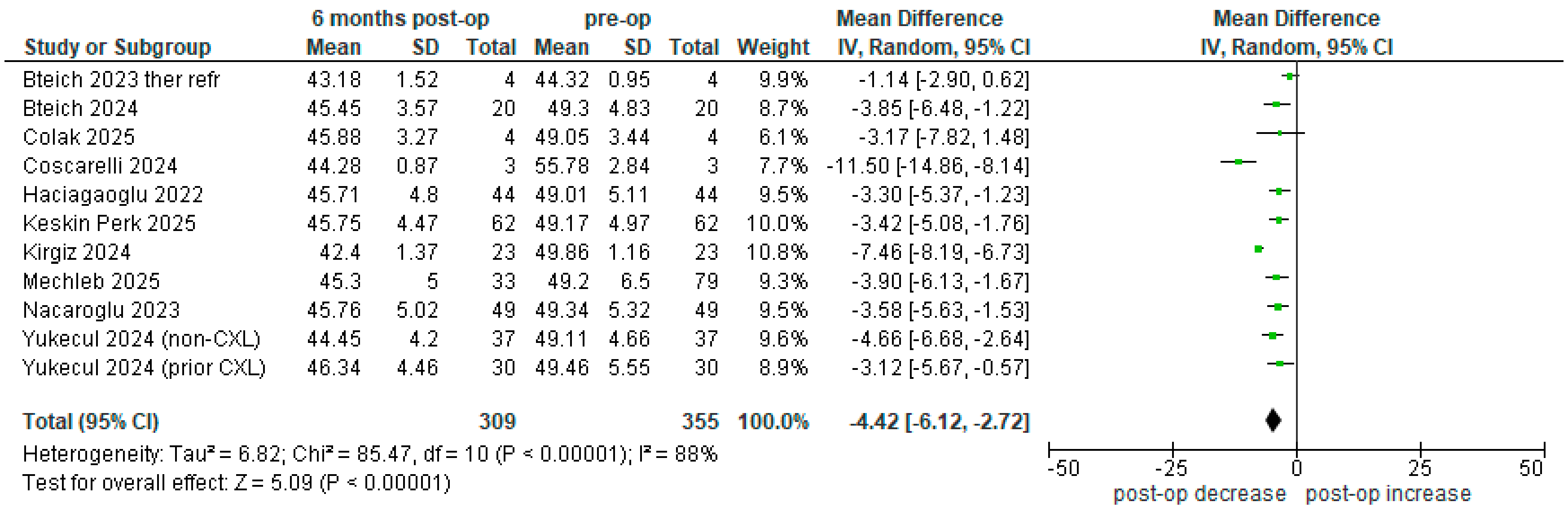

Figure S1.1.2.7. Difference in total higher order aberrations in 3 studies [2, 10, 14]

	6 months post-op			pre-op				Mean Difference
**Study or Subgroup**	**Mean**	**SD**	**Total**	**Mean**	**SD**	**Total**	**Weight**	**IV, Random, 95% CI**
Bteich 2024	1.39	0.62	20	1.92	0.95	20	69.6%	-0.53 [-1.03, -0.03]
Hayashi 2025	4.24	1.61	5	5.59	1.55	5	4.5%	-1.35 [-3.31, 0.61]
Mechleb 2025	3.47	2.08	33	3.78	1.82	79	25.9%	-0.31 [-1.13, 0.51]
**Total (95% CI)**			**58**			**104**	**100.0%**	**-0.51 [-0.92, -0.09]**

Heterogeneity: Tau² = 0.00; Chi² = 0.94, df = 2 (P = 0.62); I² = 0%
Test for overall effect: Z = 2.41 (P = 0.02)

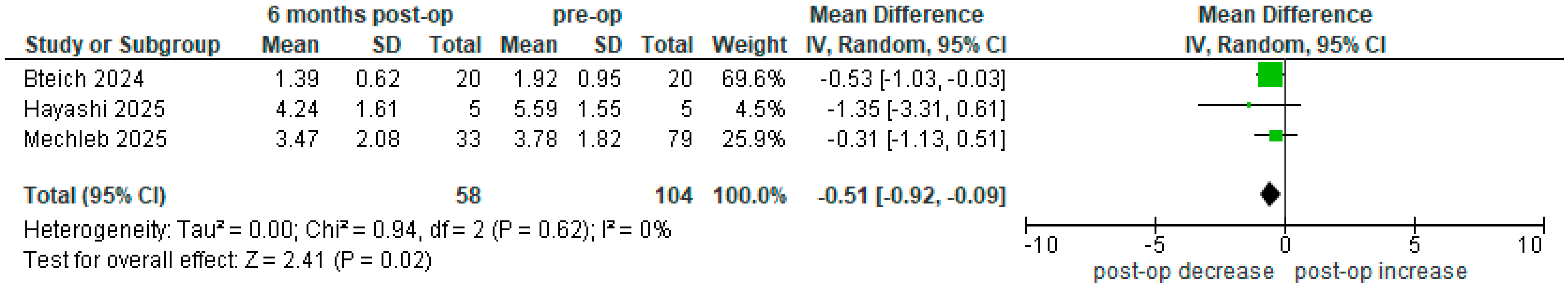

Figure S1.1.2.8. Difference in spherical aberration across 3 studies [2, 5, 12]

	6 months post-op			pre-op				Mean Difference
**Study or Subgroup**	**Mean**	**SD**	**Total**	**Mean**	**SD**	**Total**	**Weight**	**IV, Random, 95% CI**
Bteich 2023 ther refr	-0.09	0.27	4	0.26	0.08	4	31.0%	-0.35 [-0.63, -0.07]
Bteich 2024	0.32	0.46	20	0.05	0.42	20	31.1%	0.27 [-0.00, 0.54]
Kirgiz 2024	0.65	0.1	23	0.38	0.07	23	37.8%	0.27 [0.22, 0.32]
**Total (95% CI)**			**47**			**47**	**100.0%**	**0.08 [-0.28, 0.43]**

Heterogeneity: $Tau^2 = 0.09$; $Chi^2 = 18.80$, df = 2 ($P < 0.0001$); $I^2 = 89\%$
Test for overall effect: Z = 0.43 (P = 0.67)

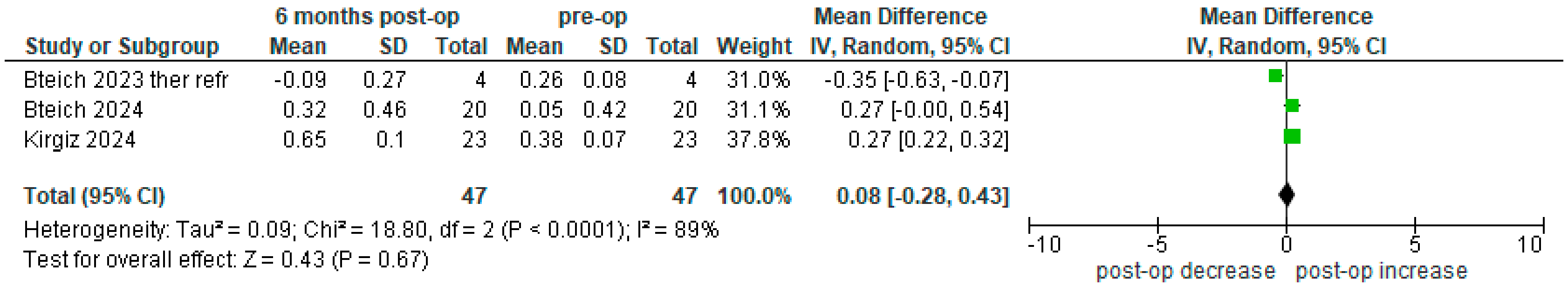

Figure S1.1.2.9. Difference in vertical coma across 2 studies [1, 12]

	6 months post-op			pre-op				Mean Difference
**Study or Subgroup**	**Mean**	**SD**	**Total**	**Mean**	**SD**	**Total**	**Weight**	**IV, Random, 95% CI**
Bteich 2023 ther refr	0.21	0.19	4	1.05	0.21	4	80.6%	-0.84 [-1.12, -0.56]
Bteich 2024	0.53	0.69	20	1.44	1.09	20	19.4%	-0.91 [-1.48, -0.34]
**Total (95% CI)**			**24**			**24**	**100.0%**	**-0.85 [-1.10, -0.60]**

Heterogeneity: Tau² = 0.00; Chi² = 0.05, df = 1 (P = 0.83); I² = 0%
Test for overall effect: Z = 6.72 (P < 0.00001)

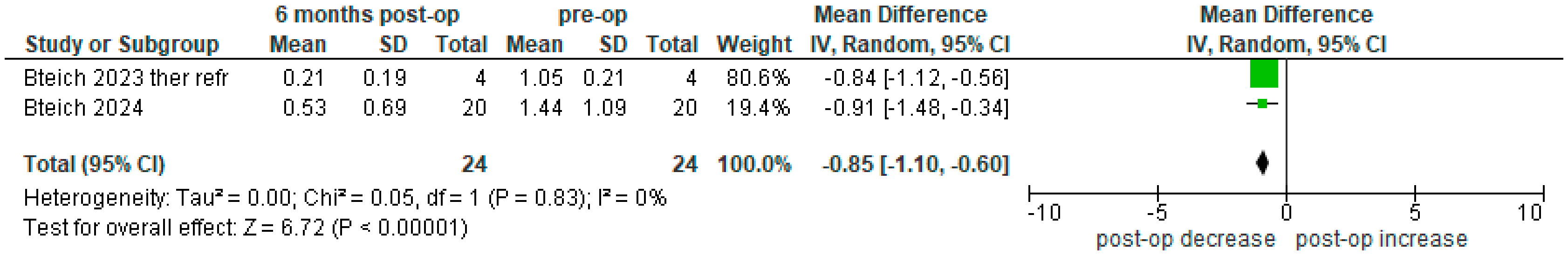

Figure S1.1.2.10. Difference in horizontal coma across 0 studies

Figure S1.1.2.11. Difference in trefoil across 2 studies [2, 5]

	6 months post-op			pre-op				Mean Difference
**Study or Subgroup**	**Mean**	**SD**	**Total**	**Mean**	**SD**	**Total**	**Weight**	**IV, Random, 95% CI**
Bteich 2024	0.1	0.6	20	-0.27	0.4	20	43.0%	0.37 [0.05, 0.69]
Kirgiz 2024	1.45	0.11	23	0.66	0.13	23	57.0%	0.79 [0.72, 0.86]
**Total (95% CI)**			**43**			**43**	**100.0%**	**0.61 [0.20, 1.02]**

Heterogeneity: $Tau^2 = 0.07$; $Chi^2 = 6.47$, df = 1 (P = 0.01); $I^2 = 85\%$
Test for overall effect: Z = 2.93 (P = 0.003)

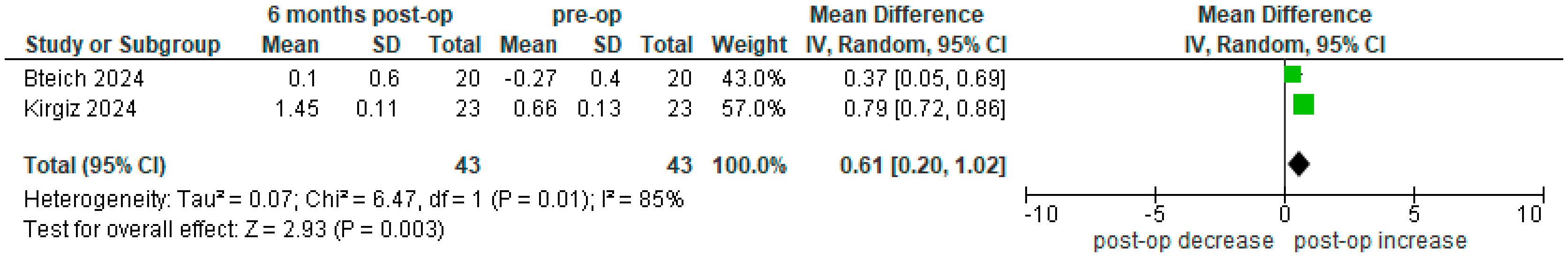

Figure S1.1.2.12. Difference in total RMS in 2 studies [5, 14]

	6 months post-op			pre-op				Mean Difference
**Study or Subgroup**	**Mean**	**SD**	**Total**	**Mean**	**SD**	**Total**	**Weight**	**IV, Random, 95% CI**
Kirgiz 2024	2.44	0.15	23	2.35	0.3	23	56.1%	0.09 [-0.05, 0.23]
Mechleb 2025	10.76	6.24	33	14.37	6.33	79	43.9%	-3.61 [-6.16, -1.06]
**Total (95% CI)**			**56**			**102**	**100.0%**	**-1.53 [-5.13, 2.07]**

Heterogeneity: Tau² = 6.00; Chi² = 8.09, df = 1 (P = 0.004); I² = 88%
Test for overall effect: Z = 0.83 (P = 0.40)

Mean Difference
IV, Random, 95% CI

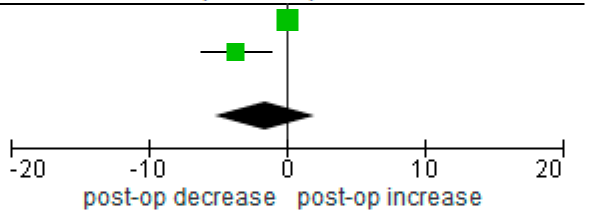

S1.1.3. 1 year postoperative versus preoperative

Figure S1.1.3.1. Difference in uncorrected visual acuity across 7 studies [2, 4, 6, 15-18]

	1 year post-op			pre-op				Mean Difference
**Study or Subgroup**	**Mean**	**SD**	**Total**	**Mean**	**SD**	**Total**	**Weight**	**IV, Random, 95% CI**
Asfar 2024	0.47	0.22	34	0.77	0.38	34	15.7%	-0.30 [-0.45, -0.15]
Bteich 2024	0.52	0.16	20	0.893	0.33	20	14.5%	-0.37 [-0.53, -0.21]
Jacob 2018	0.44	0.31	24	0.66	0.4	24	11.2%	-0.22 [-0.42, -0.02]
Jacob 2023	0.33	0.24	32	0.65	0.24	32	18.8%	-0.32 [-0.44, -0.20]
Keskin Perk 2025	0.44	0.35	50	0.96	0.5	62	14.7%	-0.52 [-0.68, -0.36]
Nacaroglu 2023	0.36	0.25	49	0.91	0.5	49	14.9%	-0.55 [-0.71, -0.39]
Yakut 2025	0.3	0.25	35	0.74	0.61	35	10.2%	-0.44 [-0.66, -0.22]
**Total (95% CI)**			**244**			**256**	**100.0%**	**-0.39 [-0.48, -0.30]**

Heterogeneity: $Tau^2 = 0.01$; $Chi^2 = 12.34$, df = 6 (P = 0.05); $I^2 = 51\%$
Test for overall effect: Z = 8.74 (P < 0.00001)

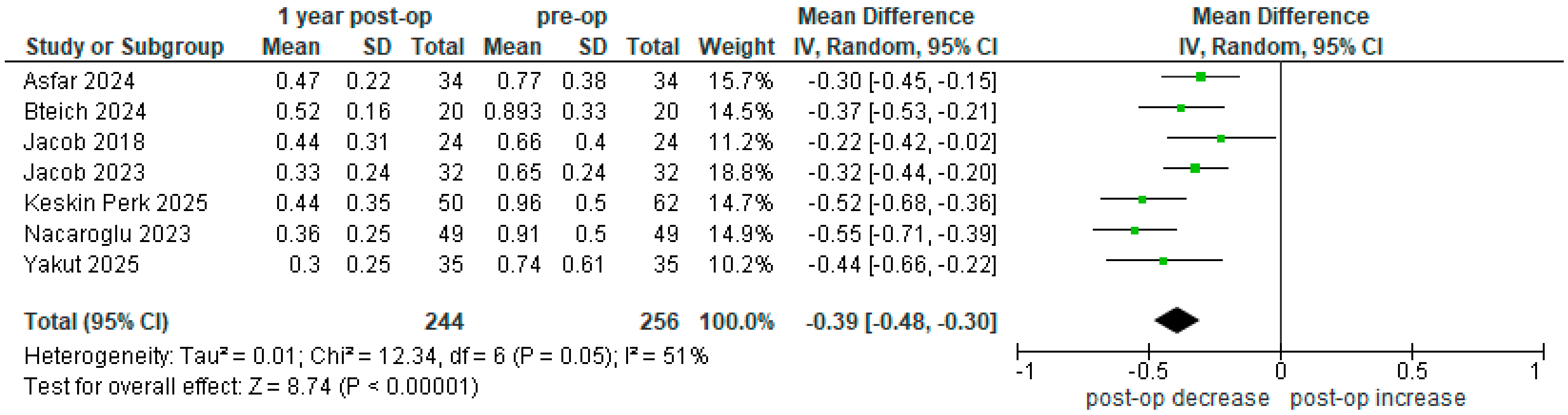

Figure S1.1.3.2. Difference in best corrected visual acuity across 7 studies [2, 4, 6, 15-18]

	1 year post-op			pre-op				Mean Difference
**Study or Subgroup**	**Mean**	**SD**	**Total**	**Mean**	**SD**	**Total**	**Weight**	**IV, Random, 95% CI**
Asfar 2024	0.16	0.18	34	0.52	0.23	34	14.3%	-0.36 [-0.46, -0.26]
Bteich 2024	0.15	0.18	20	0.57	0.18	20	14.0%	-0.42 [-0.53, -0.31]
Jacob 2018	0.1	0.11	24	0.23	0.16	24	14.5%	-0.13 [-0.21, -0.05]
Jacob 2023	0.05	0.06	32	0.12	0.11	32	14.9%	-0.07 [-0.11, -0.03]
Keskin Perk 2025	0.23	0.21	50	0.72	0.47	62	13.7%	-0.49 [-0.62, -0.36]
Nacaroglu 2023	0.36	0.15	49	0.87	0.2	49	14.6%	-0.51 [-0.58, -0.44]
Yakut 2025	0.15	0.15	35	0.54	0.32	35	13.9%	-0.39 [-0.51, -0.27]
**Total (95% CI)**			**244**			**256**	**100.0%**	**-0.34 [-0.50, -0.18]**

Heterogeneity: Tau² = 0.04; Chi² = 159.86, df = 6 (P < 0.00001); I² = 96%
Test for overall effect: Z = 4.11 (P < 0.0001)

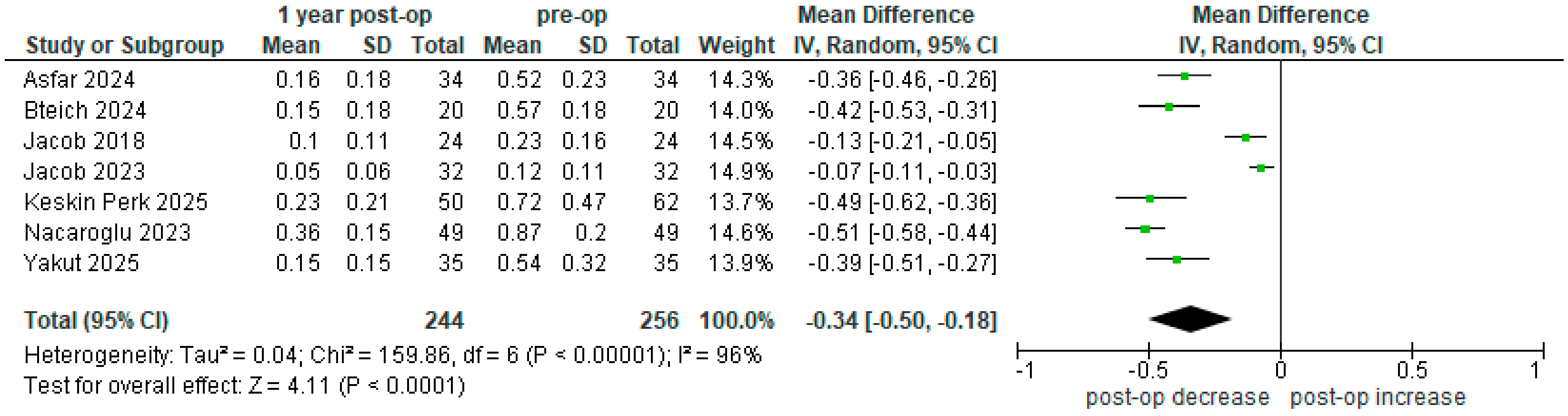

Figure S1.1.3.3. Difference in pachymetry thinnest point across 3 studies [2, 4, 17]

	1 year post-op			pre-op				Mean Difference
**Study or Subgroup**	**Mean**	**SD**	**Total**	**Mean**	**SD**	**Total**	**Weight**	**IV, Random, 95% CI**
Bteich 2024	442	51.8	20	442.8	38.1	20	19.0%	-0.80 [-28.98, 27.38]
Jacob 2023	455.94	45.74	32	449.88	35.99	32	37.2%	6.06 [-14.11, 26.23]
Keskin Perk 2025	444.38	50.15	50	449.68	49.48	62	43.8%	-5.30 [-23.87, 13.27]
**Total (95% CI)**			**102**			**114**	**100.0%**	**-0.22 [-12.52, 12.07]**

Heterogeneity: Tau² = 0.00; Chi² = 0.66, df = 2 (P = 0.72); I² = 0%
Test for overall effect: Z = 0.04 (P = 0.97)

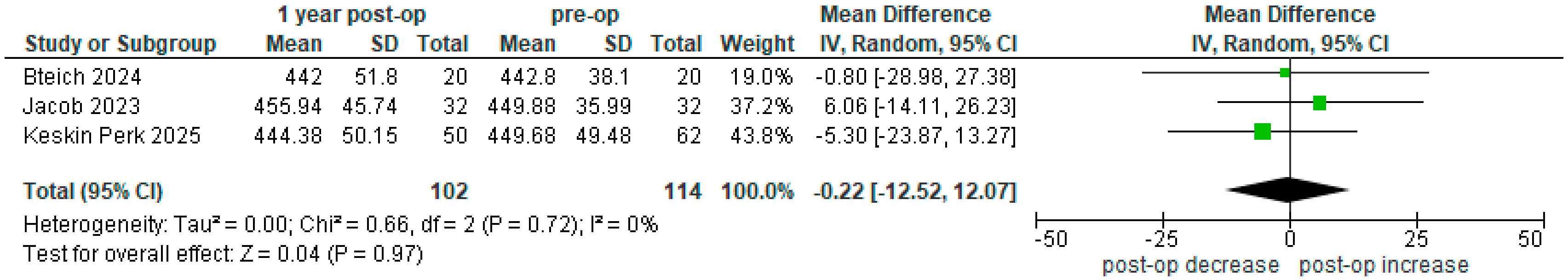

Figure S1.1.3.4. Difference in pachymetry central point in 2 studies [6, 18]

	1 year post-op			pre-op				Mean Difference
**Study or Subgroup**	**Mean**	**SD**	**Total**	**Mean**	**SD**	**Total**	**Weight**	**IV, Random, 95% CI**
Nacaroglu 2023	447.31	56.35	49	463.62	54.88	49	52.3%	-16.31 [-38.33, 5.71]
Yakut 2025	452	50.75	35	453.91	47.65	35	47.7%	-1.91 [-24.97, 21.15]
**Total (95% CI)**			**84**			**84**	**100.0%**	**-9.44 [-25.37, 6.49]**

Heterogeneity: Tau² = 0.00; Chi² = 0.78, df = 1 (P = 0.38); I² = 0%
Test for overall effect: Z = 1.16 (P = 0.25)

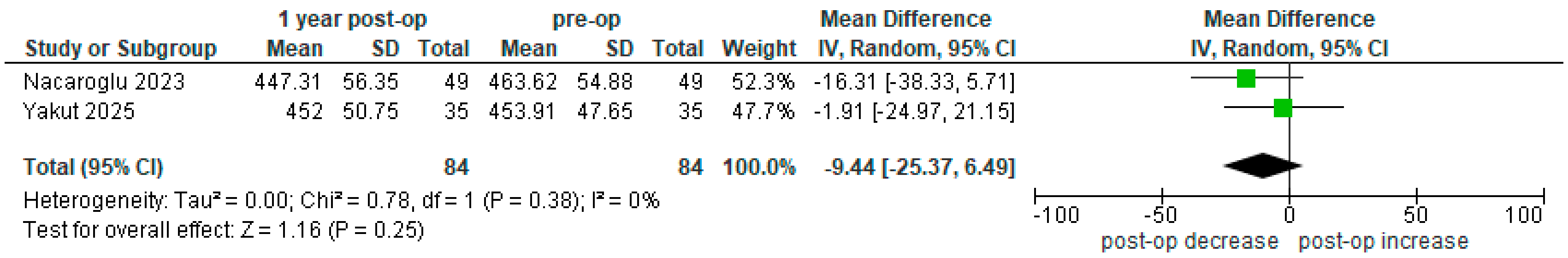

Figure S1.1.3.5. Difference in maximum keratometry across 6 studies [2, 4, 6, 15, 17, 18]

	1 year post-op			pre-op				Mean Difference
**Study or Subgroup**	**Mean**	**SD**	**Total**	**Mean**	**SD**	**Total**	**Weight**	**IV, Random, 95% CI**
Asfar 2024	50.69	6.38	34	55.85	7.53	34	12.6%	-5.16 [-8.48, -1.84]
Bteich 2024	52.85	5.44	20	57.21	7.53	20	8.4%	-4.36 [-8.43, -0.29]
Jacob 2023	54.95	4.71	32	58.9	5.54	32	21.9%	-3.95 [-6.47, -1.43]
Keskin Perk 2025	55.17	5.87	50	58.51	7.55	62	22.5%	-3.34 [-5.83, -0.85]
Nacaroglu 2023	55.63	6.14	49	59.2	7.66	49	18.4%	-3.57 [-6.32, -0.82]
Yakut 2025	54.29	5.61	35	57	6.81	35	16.3%	-2.71 [-5.63, 0.21]
**Total (95% CI)**			**220**			**232**	**100.0%**	**-3.73 [-4.91, -2.55]**

Heterogeneity: $Tau^2 = 0.00$; $Chi^2 = 1.41$, df = 5 (P = 0.92); $I^2 = 0\%$
Test for overall effect: Z = 6.20 (P < 0.00001)

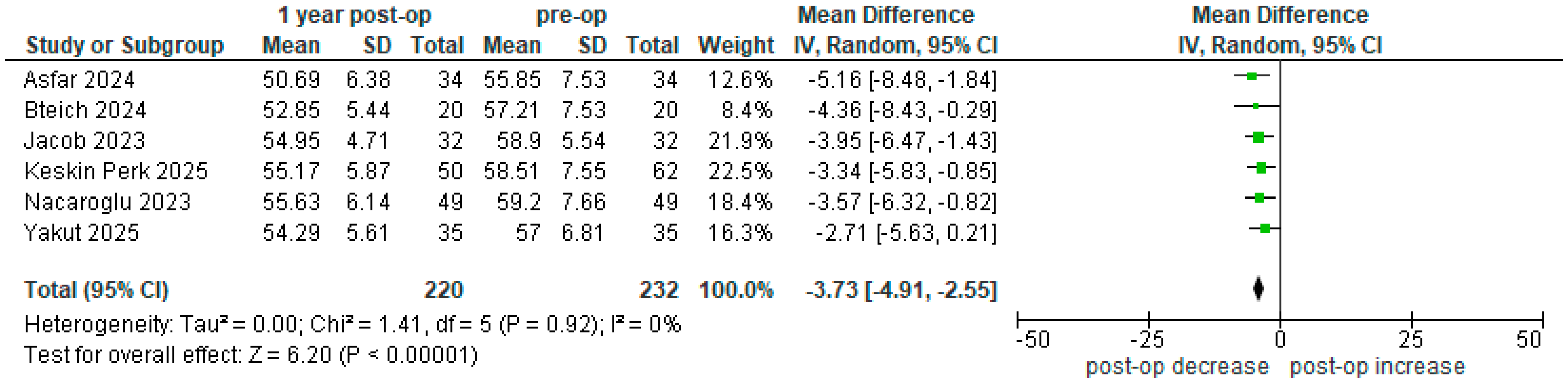

Figure S1.1.3.6. Difference in mean simulated keratometry across 6 studies [2, 4, 6, 15, 17, 18]

	1 year post-op			pre-op				Mean Difference	Mean Difference
**Study or Subgroup**	**Mean**	**SD**	**Total**	**Mean**	**SD**	**Total**	**Weight**	**IV, Random, 95% CI**	**IV, Random, 95% CI**
Asfar 2024	44.25	3.29	34	47.52	3.82	34	19.7%	-3.27 [-4.96, -1.58]	
Bteich 2024	45.23	2.83	20	49.3	4.83	20	9.4%	-4.07 [-6.52, -1.62]	
Jacob 2023	45.63	2.91	32	49.07	3.06	32	26.4%	-3.44 [-4.90, -1.98]	
Keskin Perk 2025	45.63	4.44	50	49.17	4.97	62	18.6%	-3.54 [-5.28, -1.80]	
Nacaroglu 2023	45.39	4.92	49	49.34	5.32	49	13.7%	-3.95 [-5.98, -1.92]	
Yakut 2025	44.5	4.42	35	48.26	4.78	35	12.2%	-3.76 [-5.92, -1.60]	
**Total (95% CI)**			**220**			**232**	**100.0%**	**-3.59 [-4.35, -2.84]**	

Heterogeneity: $Tau^2 = 0.00$; $Chi^2 = 0.47$, df = 5 (P = 0.99); $I^2 = 0\%$
Test for overall effect: Z = 9.36 (P < 0.00001)

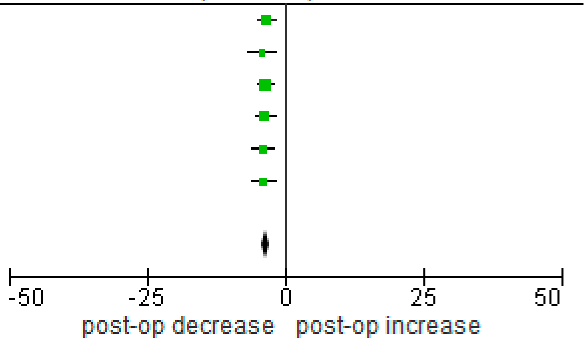

Figure S1.1.3.7. Difference in total higher order aberrations in 2 studies [2, 15]

	1 year post-op			pre-op				Mean Difference
**Study or Subgroup**	**Mean**	**SD**	**Total**	**Mean**	**SD**	**Total**	**Weight**	**IV, Random, 95% CI**
Asfar 2024	1.54	0.93	34	2.07	1.05	34	53.4%	-0.53 [-1.00, -0.06]
Bteich 2024	1.36	0.65	20	1.92	0.95	20	46.6%	-0.56 [-1.06, -0.06]
**Total (95% CI)**			**54**			**54**	**100.0%**	**-0.54 [-0.89, -0.20]**

Heterogeneity: Tau² = 0.00; Chi² = 0.01, df = 1 (P = 0.93); I² = 0%
Test for overall effect: Z = 3.10 (P = 0.002)

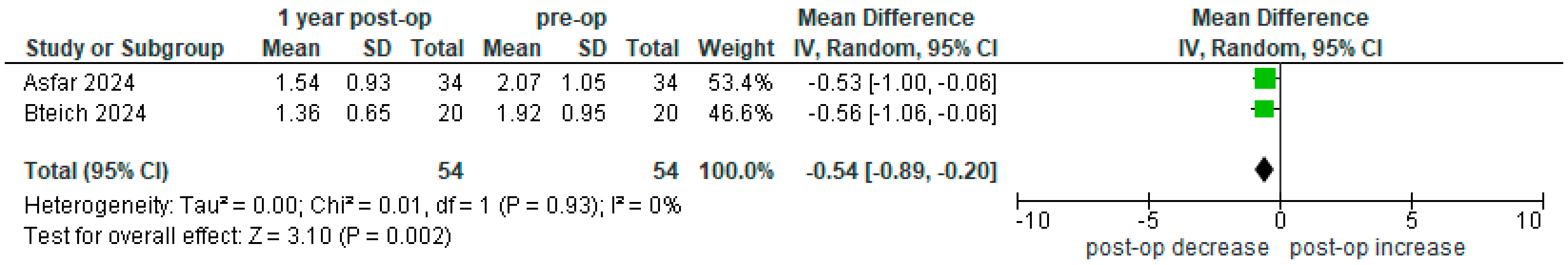

Figure S1.1.3.8. Difference in spherical aberration in 2 studies [2, 15]

	1 year post-op			pre-op				Mean Difference
**Study or Subgroup**	**Mean**	**SD**	**Total**	**Mean**	**SD**	**Total**	**Weight**	**IV, Random, 95% CI**
Asfar 2024	0.18	0.5	34	0.07	0.42	34	60.7%	0.11 [-0.11, 0.33]
Bteich 2024	0.15	0.46	20	0.05	0.42	20	39.3%	0.10 [-0.17, 0.37]
**Total (95% CI)**			**54**			**54**	**100.0%**	**0.11 [-0.06, 0.28]**

Heterogeneity: Tau² = 0.00; Chi² = 0.00, df = 1 (P = 0.96); I² = 0%
Test for overall effect: Z = 1.22 (P = 0.22)

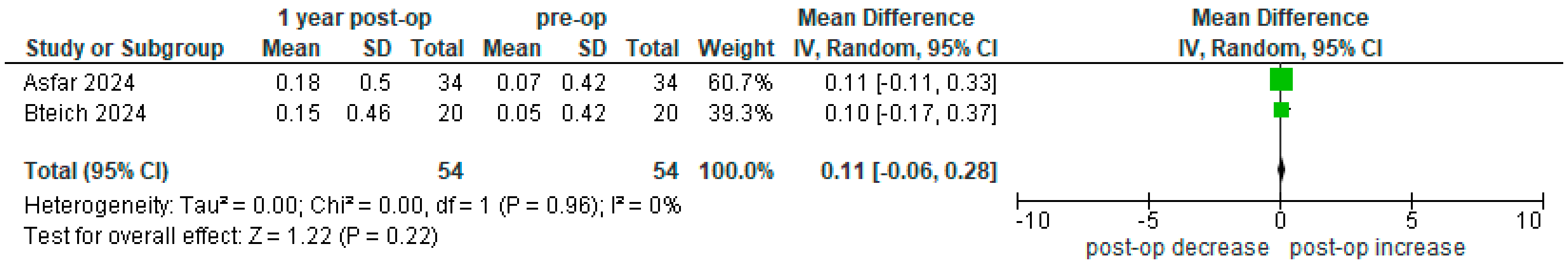

Figure S1.1.3.9. Difference in vertical coma across 3 studies [2, 15, 17]

	1 year post-op			pre-op				Mean Difference
**Study or Subgroup**	**Mean**	**SD**	**Total**	**Mean**	**SD**	**Total**	**Weight**	**IV, Random, 95% CI**
Asfar 2024	0.38	0.65	34	1.49	1.02	34	34.3%	-1.11 [-1.52, -0.70]
Bteich 2024	0.35	0.97	20	1.44	1.09	20	32.4%	-1.09 [-1.73, -0.45]
Jacob 2023	-1.4	1.21	32	-2.09	0.96	32	33.3%	0.69 [0.15, 1.23]
**Total (95% CI)**			**86**			**86**	**100.0%**	**-0.50 [-1.68, 0.67]**

Heterogeneity: Tau² = 1.00; Chi² = 30.61, df = 2 (P < 0.00001); I² = 93%
Test for overall effect: Z = 0.84 (P = 0.40)

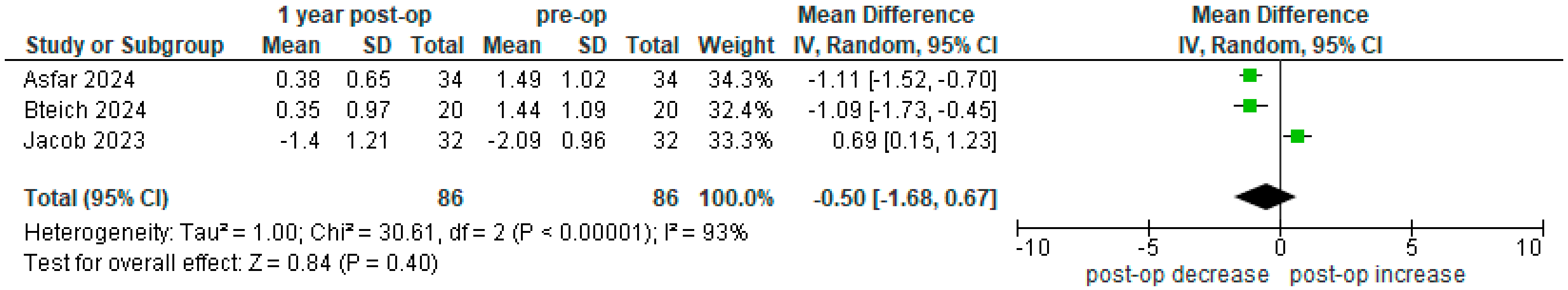

Figure S1.1.3.10. A. Difference in horizontal coma across 1 study [15]

Study or Subgroup	1 year post-op Mean	SD	Total	pre-op Mean	SD	Total	Weight	Mean Difference IV, Random, 95% CI
Asfar 2024	-0.03	0.77	34	0.13	0.86	34	100.0%	-0.16 [-0.55, 0.23]
**Total (95% CI)**			**34**			**34**	**100.0%**	**-0.16 [-0.55, 0.23]**

Heterogeneity: Not applicable
Test for overall effect: Z = 0.81 (P = 0.42)

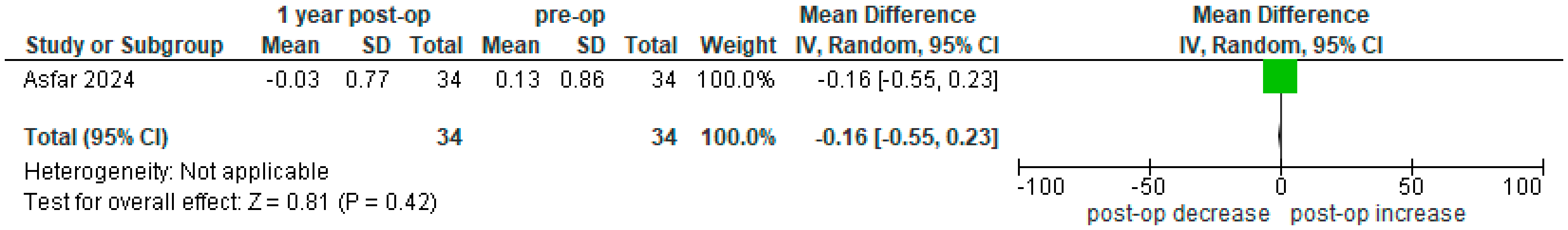

Figure S1.1.3.11. Difference in trefoil in 2 studies [2, 15]

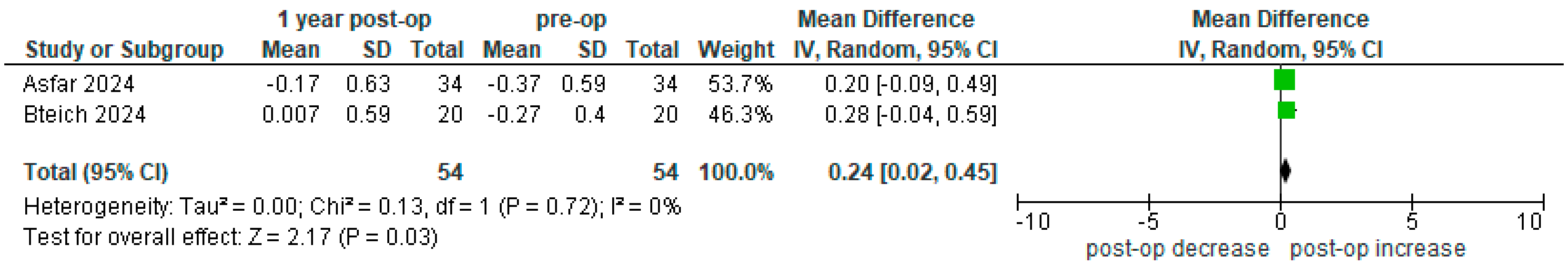

Figure S1.1.3.12. Difference in total RMS in 1 study [17]

	1 year post-op			pre-op				Mean Difference
**Study or Subgroup**	**Mean**	**SD**	**Total**	**Mean**	**SD**	**Total**	**Weight**	**IV, Random, 95% CI**
Jacob 2023	2.59	0.8	32	2.85	0.78	32	100.0%	-0.26 [-0.65, 0.13]
**Total (95% CI)**			**32**			**32**	**100.0%**	**-0.26 [-0.65, 0.13]**

Heterogeneity: Not applicable
Test for overall effect: Z = 1.32 (P = 0.19)

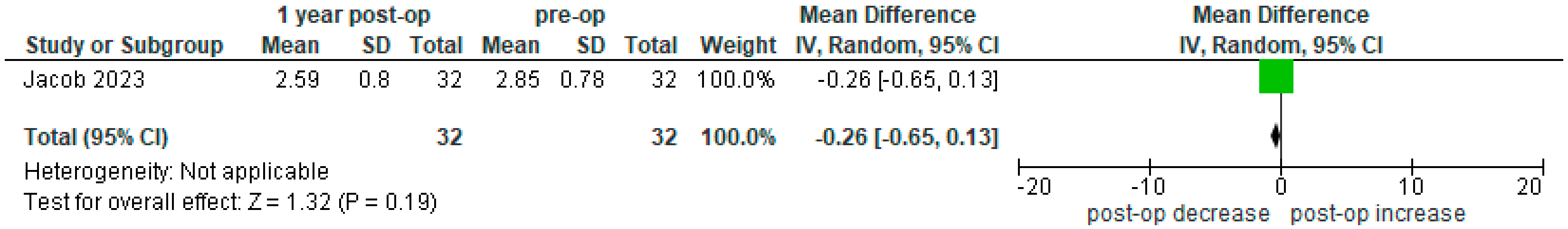

Section S1.2– SUBGROUP ANALYSIS CAIRS PREPARATION – TREPHINE VS KERANATURAL VS FEMTOSECOND LASER

S1.2.1. 1 month postoperative versus preoperative

S1.2.1.1. Difference in uncorrected visual acuity

Figure S1.2.1.1A. Difference in uncorrected visual acuity across 7 studies [1-7] – TREPHINE

	1 month post-op			pre-op				Mean Difference
**Study or Subgroup**	**Mean**	**SD**	**Total**	**Mean**	**SD**	**Total**	**Weight**	**IV, Random, 95% CI**
Bteich 2023	0.64	0.23	52	0.97	0.47	52	17.6%	-0.33 [-0.47, -0.19]
Bteich 2024	0.66	0.33	20	0.893	0.33	20	0.0%	-0.23 [-0.44, -0.03]
Haciagaoglu 2022	0.34	0.26	44	0.72	0.63	44	14.7%	-0.38 [-0.58, -0.18]
Keskin Perk 2025	0.4	0.34	62	0.96	0.5	62	17.2%	-0.56 [-0.71, -0.41]
Kirgiz 2024	0.432	0.03	23	1.096	0.05	23	21.6%	-0.66 [-0.69, -0.64]
Nacaroglu 2023	0.39	0.28	49	0.91	0.5	49	16.7%	-0.52 [-0.68, -0.36]
Yukecul 2024 (non-CXL)	0.26	0.22	37	0.77	0.76	37	12.3%	-0.51 [-0.76, -0.26]
Yukecul 2024 (prior CXL)	0.38	0.29	30	0.7	0.55	30	0.0%	-0.32 [-0.54, -0.10]
**Total (95% CI)**			**267**			**267**	**100.0%**	**-0.50 [-0.64, -0.37]**

Heterogeneity: $Tau^2 = 0.02$; $Chi^2 = 32.57$, $df = 5$ ($P < 0.00001$); $I^2 = 85\%$
Test for overall effect: $Z = 7.27$ ($P < 0.00001$)

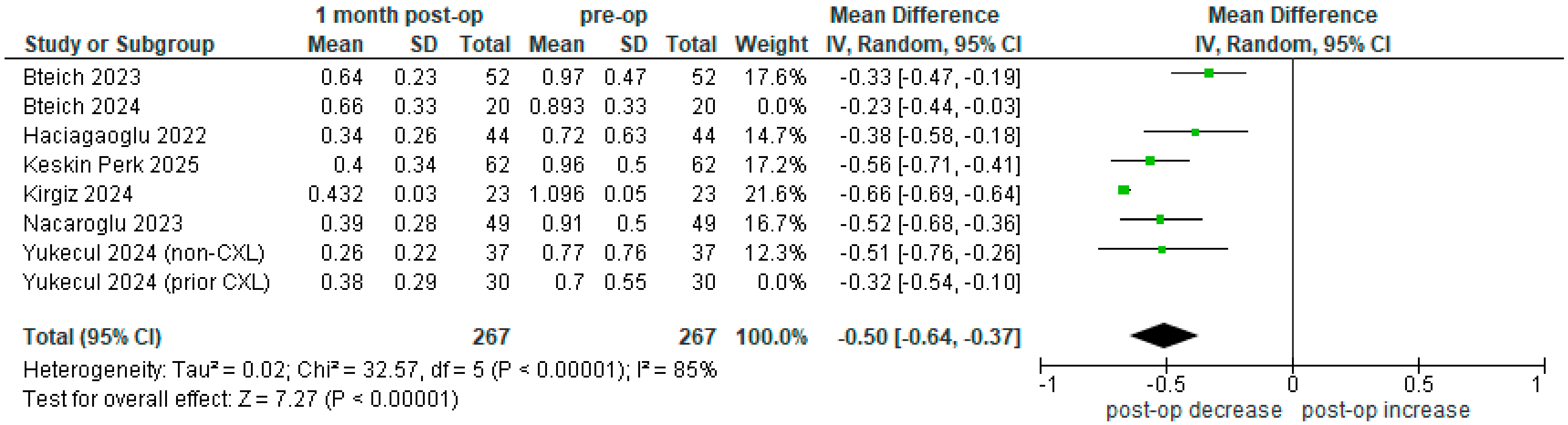

Figure S1.2.1.1B. Difference in uncorrected visual acuity across 1 study [2] – FEMTOSECOND LASER

	1 month post-op			pre-op				Mean Difference
**Study or Subgroup**	**Mean**	**SD**	**Total**	**Mean**	**SD**	**Total**	**Weight**	**IV, Random, 95% CI**
Bteich 2023	0.64	0.23	52	0.97	0.47	52	0.0%	-0.33 [-0.47, -0.19]
Bteich 2024	0.66	0.33	20	0.893	0.33	20	100.0%	-0.23 [-0.44, -0.03]
Haciagaoglu 2022	0.34	0.26	44	0.72	0.63	44	0.0%	-0.38 [-0.58, -0.18]
Keskin Perk 2025	0.4	0.34	62	0.96	0.5	62	0.0%	-0.56 [-0.71, -0.41]
Kirgiz 2024	0.432	0.03	23	1.096	0.05	23	0.0%	-0.66 [-0.69, -0.64]
Nacaroglu 2023	0.39	0.28	49	0.91	0.5	49	0.0%	-0.52 [-0.68, -0.36]
Yukecul 2024 (non-CXL)	0.26	0.22	37	0.77	0.76	37	0.0%	-0.51 [-0.76, -0.26]
Yukecul 2024 (prior CXL)	0.38	0.29	30	0.7	0.55	30	0.0%	-0.32 [-0.54, -0.10]
**Total (95% CI)**			**20**			**20**	**100.0%**	**-0.23 [-0.44, -0.03]**

Heterogeneity: Not applicable
Test for overall effect: Z = 2.23 (P = 0.03)

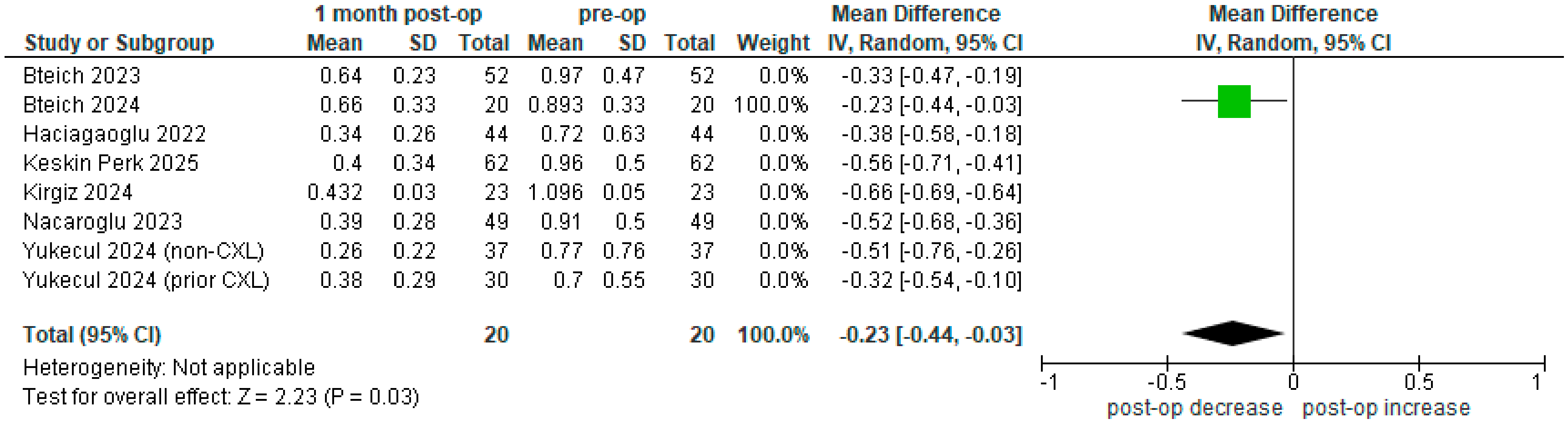

S1.2.1.2. Difference in best corrected visual acuity

Figure S1.2.1.2A. Difference in best corrected visual acuity across 6 studies [1, 3-7] – TREPHINE

	1 month post-op			pre-op				Mean Difference
**Study or Subgroup**	**Mean**	**SD**	**Total**	**Mean**	**SD**	**Total**	**Weight**	**IV, Random, 95% CI**
Bteich 2023	0.31	0.21	52	0.56	0.19	52	15.2%	-0.25 [-0.33, -0.17]
Bteich 2024	0.32	0.2	20	0.57	0.18	20	0.0%	-0.25 [-0.37, -0.13]
Haciagaoglu 2022	0.24	0.19	44	0.54	0.37	44	14.0%	-0.30 [-0.42, -0.18]
Keskin Perk 2025	0.28	0.22	62	0.72	0.47	62	13.8%	-0.44 [-0.57, -0.31]
Kirgiz 2024	0.237	0.06	23	0.602	0.3	23	13.9%	-0.36 [-0.49, -0.24]
Mechleb 2024 (Kmax inf 75D)	0.03	0.02	6	0.36	0.12	6	0.0%	-0.33 [-0.43, -0.23]
Mechleb 2024 (Kmax sup 75D)	1.03	0.88	4	2.45	1.1	4	0.0%	-1.42 [-2.80, -0.04]
Nacaroglu 2023	0.27	0.06	49	0.87	0.2	49	15.6%	-0.60 [-0.66, -0.54]
Yukecul 2024 (non-CXL)	0.19	0.15	37	0.51	0.29	37	14.5%	-0.32 [-0.43, -0.21]
Yukecul 2024 (prior CXL)	0.27	0.2	30	0.55	0.39	30	12.9%	-0.28 [-0.44, -0.12]
**Total (95% CI)**			**297**			**297**	**100.0%**	**-0.37 [-0.49, -0.24]**

Heterogeneity: $Tau^2 = 0.03$; $Chi^2 = 66.18$, $df = 6$ ($P < 0.00001$); $I^2 = 91\%$
Test for overall effect: $Z = 5.71$ ($P < 0.00001$)

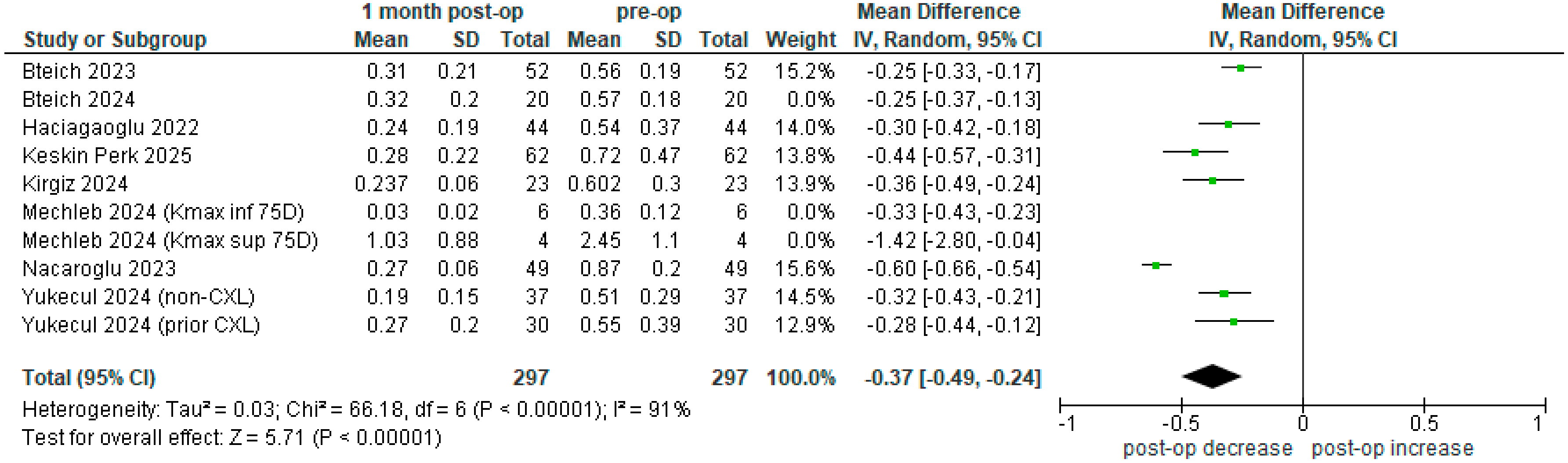

Figure S1.2.1.2B. Difference in best corrected visual acuity across 3 studies [2, 8] – FEMTOSECOND LASER

	1 month post-op			pre-op				Mean Difference
**Study or Subgroup**	**Mean**	**SD**	**Total**	**Mean**	**SD**	**Total**	**Weight**	**IV, Random, 95% CI**
Bteich 2023	0.31	0.21	52	0.56	0.19	52		Not estimable
Bteich 2024	0.32	0.2	20	0.57	0.18	20	46.0%	-0.25 [-0.37, -0.13]
Haciagaoglu 2022	0.24	0.19	44	0.54	0.37	44		Not estimable
Keskin Perk 2025	0.28	0.22	62	0.72	0.47	62		Not estimable
Kirgiz 2024	0.237	0.06	23	0.602	0.3	23		Not estimable
Mechleb 2024 (Kmax inf 75D)	0.03	0.02	6	0.36	0.12	6	53.3%	-0.33 [-0.43, -0.23]
Mechleb 2024 (Kmax sup 75D)	1.03	0.88	4	2.45	1.1	4	0.8%	-1.42 [-2.80, -0.04]
Nacaroglu 2023	0.27	0.06	49	0.87	0.2	49		Not estimable
Yukecul 2024 (non-CXL)	0.19	0.15	37	0.51	0.29	37		Not estimable
Yukecul 2024 (prior CXL)	0.27	0.2	30	0.55	0.39	30		Not estimable
**Total (95% CI)**			**30**			**30**	**100.0%**	**-0.30 [-0.42, -0.18]**

Heterogeneity: $Tau^2 = 0.00$; $Chi^2 = 3.58$, $df = 2$ ($P = 0.17$); $I^2 = 44\%$

Test for overall effect: $Z = 4.86$ ($P < 0.00001$)

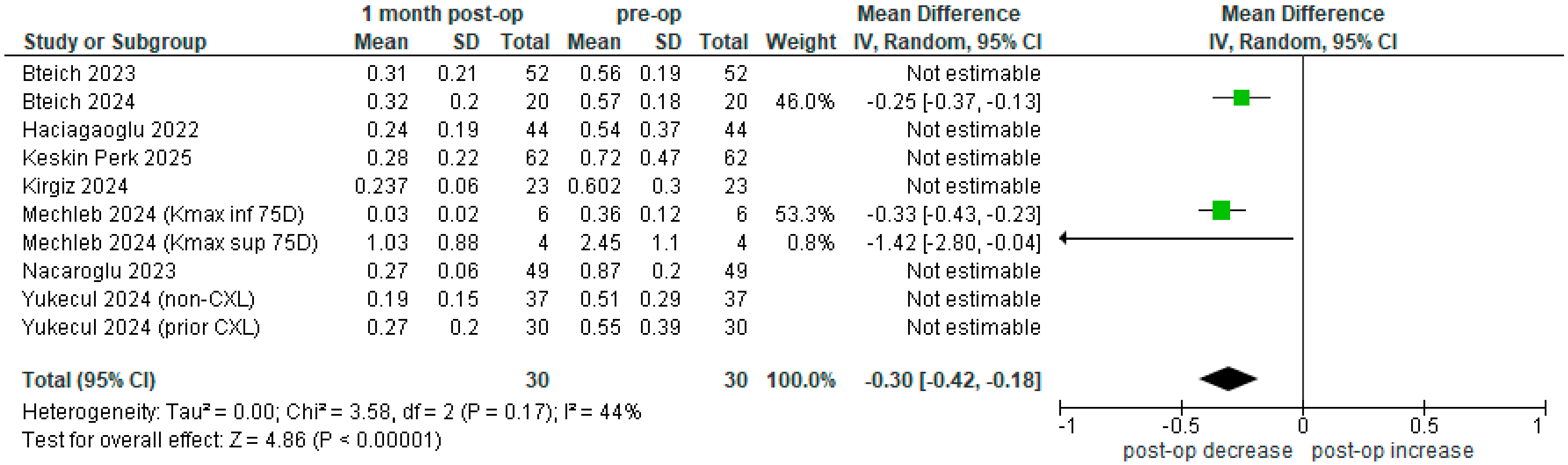

S1.2.1.3. Difference in pachymetry thinnest point

Figure S1.2.1.3A. Difference in pachymetry thinnest point across 5 studies [1, 3-5, 7] – TREPHINE

	1 month post-op			pre-op				Mean Difference
**Study or Subgroup**	**Mean**	**SD**	**Total**	**Mean**	**SD**	**Total**	**Weight**	**IV, Random, 95% CI**
Bteich 2023	429.7	55.5	52	422.5	55.2	52	5.8%	7.20 [-14.08, 28.48]
Bteich 2024	443.8	41.2	20	442.8	38.1	20	0.0%	1.00 [-23.59, 25.59]
Haciagaoglu 2022	449.2	57.41	44	453.73	45.87	44	5.6%	-4.53 [-26.24, 17.18]
Keskin Perk 2025	446.32	40.26	62	449.68	49.48	62	10.5%	-3.36 [-19.24, 12.52]
Kirgiz 2024	431.33	11.78	23	439.33	10.43	23	64.0%	-8.00 [-14.43, -1.57]
Yukecul 2024 (non-CXL)	452	36	37	454	45	37	7.7%	-2.00 [-20.57, 16.57]
Yukecul 2024 (prior CXL)	449	42	30	437	38	30	6.4%	12.00 [-8.27, 32.27]
**Total (95% CI)**			**248**			**248**	**100.0%**	**-4.68 [-9.83, 0.46]**

Heterogeneity: Tau² = 0.00; Chi² = 4.93, df = 5 (P = 0.42); I² = 0%
Test for overall effect: Z = 1.78 (P = 0.07)

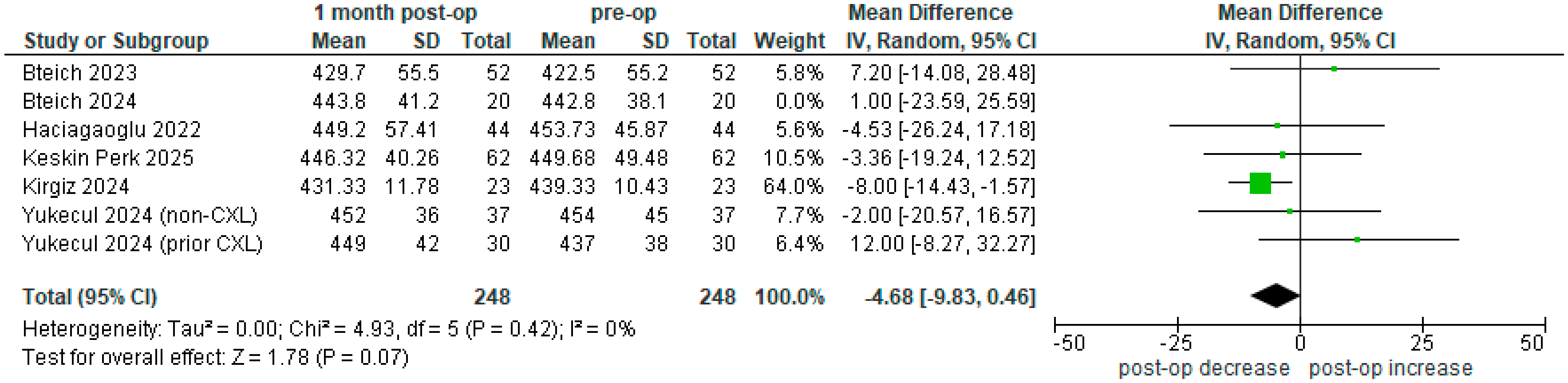

Figure S1.2.1.3B. Difference in pachymetry thinnest point across 1 study [2] – FEMTOSECOND LASER

	**1 month post-op**			**pre-op**				**Mean Difference**
**Study or Subgroup**	**Mean**	**SD**	**Total**	**Mean**	**SD**	**Total**	**Weight**	**IV, Random, 95% CI**
Bteich 2023	429.7	55.5	52	422.5	55.2	52	0.0%	7.20 [-14.08, 28.48]
Bteich 2024	443.8	41.2	20	442.8	38.1	20	100.0%	1.00 [-23.59, 25.59]
Haciagaoglu 2022	449.2	57.41	44	453.73	45.87	44	0.0%	-4.53 [-26.24, 17.18]
Keskin Perk 2025	446.32	40.26	62	449.68	49.48	62	0.0%	-3.36 [-19.24, 12.52]
Kirgiz 2024	431.33	11.78	23	439.33	10.43	23	0.0%	-8.00 [-14.43, -1.57]
Yukecul 2024 (non-CXL)	452	36	37	454	45	37	0.0%	-2.00 [-20.57, 16.57]
Yukecul 2024 (prior CXL)	449	42	30	437	38	30	0.0%	12.00 [-8.27, 32.27]
**Total (95% CI)**			**20**			**20**	**100.0%**	**1.00 [-23.59, 25.59]**

Heterogeneity: Not applicable
Test for overall effect: Z = 0.08 (P = 0.94)

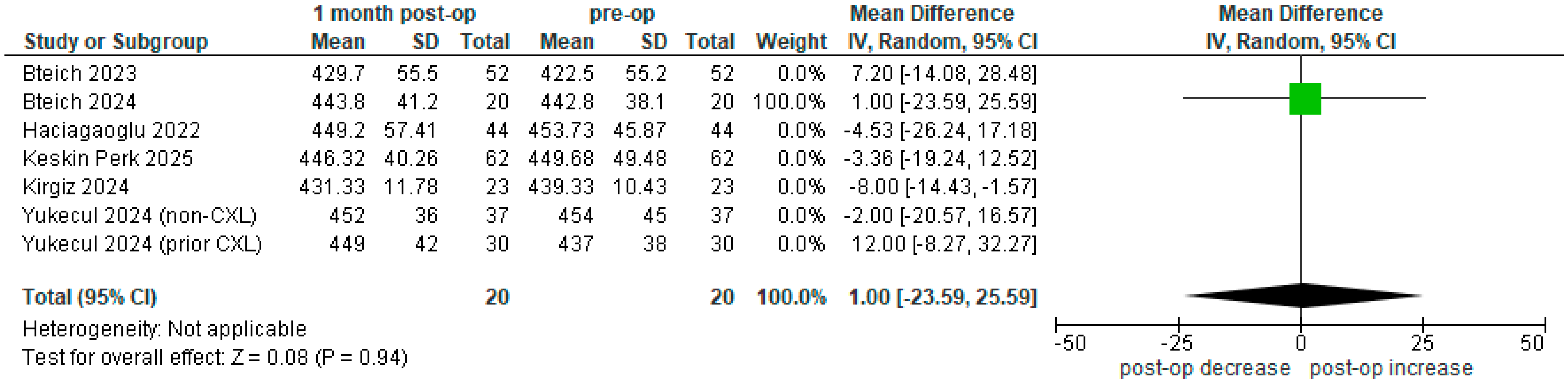

S1.2.1.4. Difference in pachymetry central point

Figure S1.2.1.4A. Difference in pachymetry central point across 1 study – TREPHINE

	1 month post-op			pre-op				Mean Difference
**Study or Subgroup**	**Mean**	**SD**	**Total**	**Mean**	**SD**	**Total**	**Weight**	**IV, Random, 95% CI**
Nacaroglu 2023	449.69	36.72	49	463.62	54.88	49	100.0%	-13.93 [-32.42, 4.56]
**Total (95% CI)**			**49**			**49**	**100.0%**	**-13.93 [-32.42, 4.56]**
Heterogeneity: Not applicable								
Test for overall effect: Z = 1.48 (P = 0.14)								

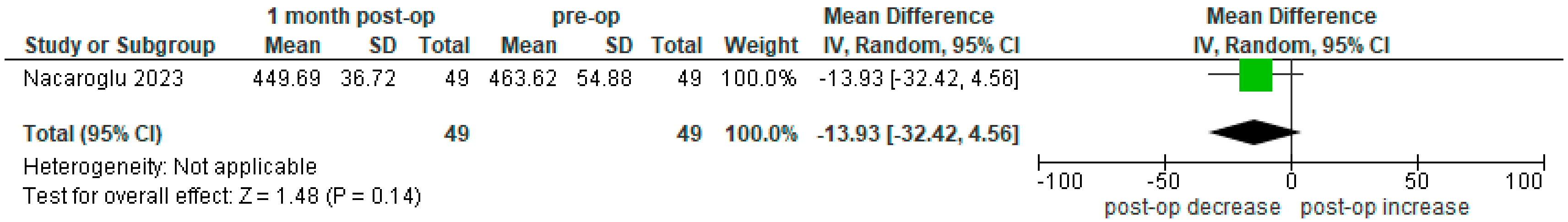

S1.2.1.5. Difference in maximum keratometry

Figure S1.2.1.5A. Difference in maximum keratometry across 6 studies [1, 3-7] – TREPHINE

	1 month post-op			pre-op				Mean Difference
**Study or Subgroup**	**Mean**	**SD**	**Total**	**Mean**	**SD**	**Total**	**Weight**	**IV, Random, 95% CI**
Bteich 2023	54.17	8.22	52	58.09	7.92	52	14.0%	-3.92 [-7.02, -0.82]
Bteich 2024	53.3	6.22	20	57.21	7.53	20	0.0%	-3.91 [-8.19, 0.37]
Haciagaoglu 2022	55.46	6.97	44	57.56	7.06	44	14.2%	-2.10 [-5.03, 0.83]
Keskin Perk 2025	56.34	7.43	62	58.51	7.55	62	14.5%	-2.17 [-4.81, 0.47]
Kirgiz 2024	48.18	2.03	23	57.65	1.41	23	15.7%	-9.47 [-10.48, -8.46]
Mechleb 2024 (Kmax inf 75D)	51.93	5.79	6	54.43	4.09	6	0.0%	-2.50 [-8.17, 3.17]
Mechleb 2024 (Kmax sup 75D)	67.02	10.95	4	81.5	7.95	4	0.0%	-14.48 [-27.74, -1.22]
Nacaroglu 2023	56.75	7.72	49	59.2	7.66	49	14.1%	-2.45 [-5.50, 0.60]
Yukecul 2024 (non-CXL)	56.17	7.13	37	58.38	7.24	37	13.8%	-2.21 [-5.48, 1.06]
Yukecul 2024 (prior CXL)	55.28	6.26	30	57.22	6.68	30	13.8%	-1.94 [-5.22, 1.34]
**Total (95% CI)**			**297**			**297**	**100.0%**	**-3.56 [-6.88, -0.25]**

Heterogeneity: $Tau^2 = 17.98$; $Chi^2 = 75.57$, $df = 6$ ($P < 0.00001$); $I^2 = 92\%$
Test for overall effect: $Z = 2.11$ ($P = 0.04$)

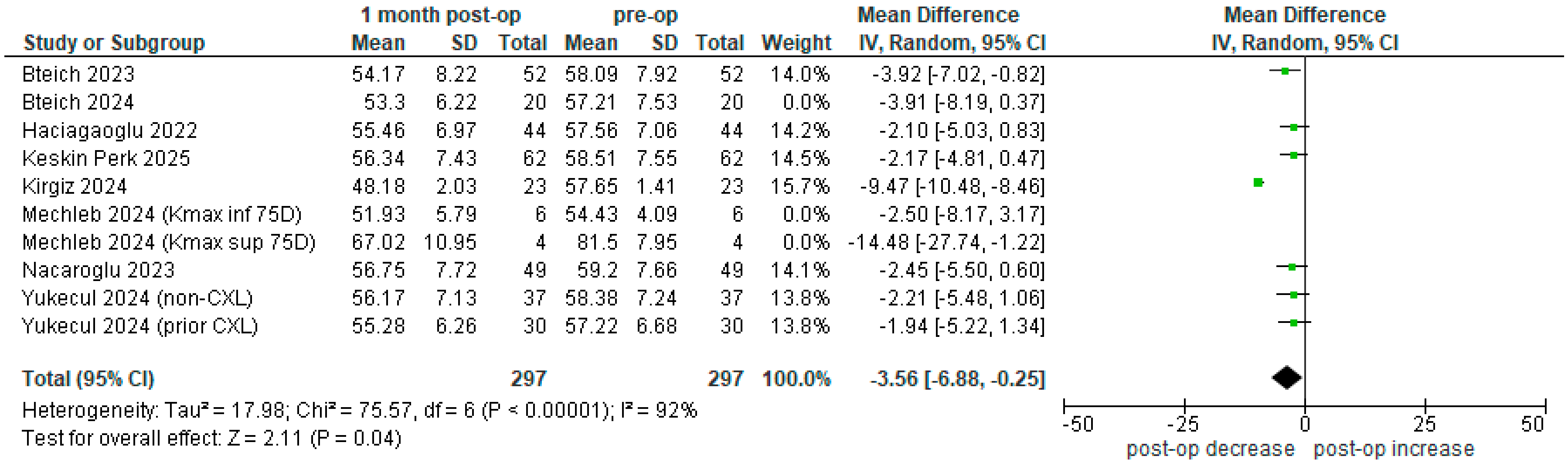

Figure S1.2.1.5B. Difference in maximum keratometry across 2 studies [2, 8]– FEMTOSECOND LASER

	1 month post-op			pre-op				Mean Difference
**Study or Subgroup**	**Mean**	**SD**	**Total**	**Mean**	**SD**	**Total**	**Weight**	**IV, Random, 95% CI**
Bteich 2023	54.17	8.22	52	58.09	7.92	52	0.0%	-3.92 [-7.02, -0.82]
Bteich 2024	53.3	6.22	20	57.21	7.53	20	53.5%	-3.91 [-8.19, 0.37]
Haciagaoglu 2022	55.46	6.97	44	57.56	7.06	44	0.0%	-2.10 [-5.03, 0.83]
Keskin Perk 2025	56.34	7.43	62	58.51	7.55	62	0.0%	-2.17 [-4.81, 0.47]
Kirgiz 2024	48.18	2.03	23	57.65	1.41	23	0.0%	-9.47 [-10.48, -8.46]
Mechleb 2024 (Kmax inf 75D)	51.93	5.79	6	54.43	4.09	6	37.4%	-2.50 [-8.17, 3.17]
Mechleb 2024 (Kmax sup 75D)	67.02	10.95	4	81.5	7.95	4	9.1%	-14.48 [-27.74, -1.22]
Nacaroglu 2023	56.75	7.72	49	59.2	7.66	49	0.0%	-2.45 [-5.50, 0.60]
Yukecul 2024 (non-CXL)	56.17	7.13	37	58.38	7.24	37	0.0%	-2.21 [-5.48, 1.06]
Yukecul 2024 (prior CXL)	55.28	6.26	30	57.22	6.68	30	0.0%	-1.94 [-5.22, 1.34]
**Total (95% CI)**			**30**			**30**	**100.0%**	**-4.34 [-8.50, -0.19]**

Heterogeneity: $Tau^2 = 3.63$; $Chi^2 = 2.67$, df = 2 (P = 0.26); $I^2 = 25\%$

Test for overall effect: Z = 2.05 (P = 0.04)

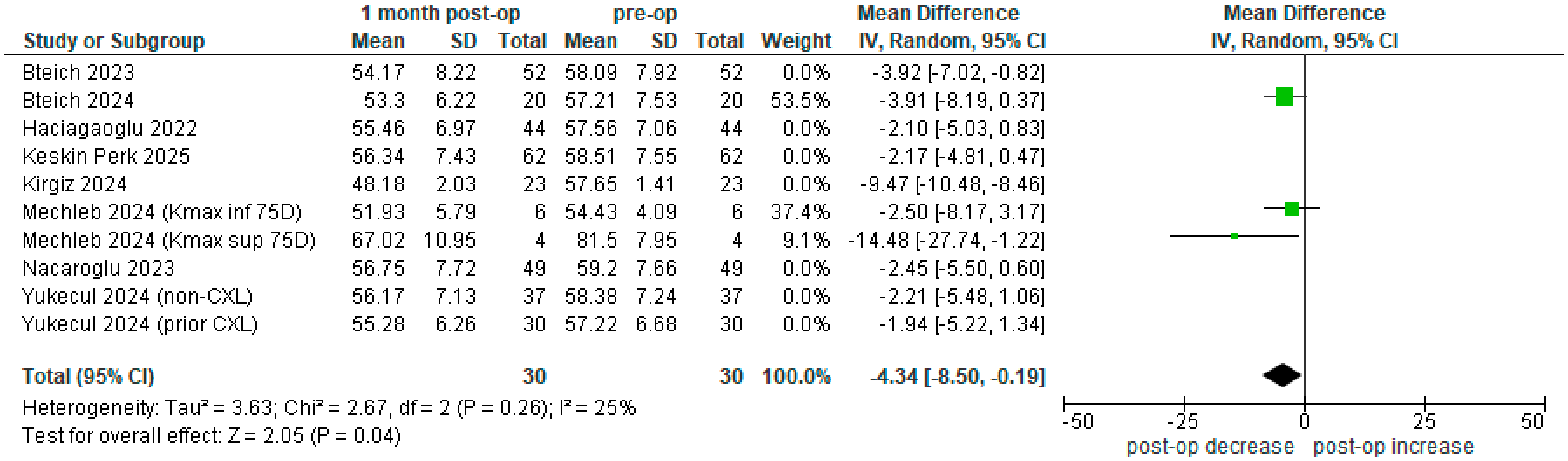

S1.2.1.6. Difference in mean simulated keratometry

Figure S1.2.1.6A. Difference in mean simulated keratometry across 6 studies [1, 3-7]- TREPHINE

	1 month post-op			pre-op				Mean Difference
**Study or Subgroup**	**Mean**	**SD**	**Total**	**Mean**	**SD**	**Total**	**Weight**	**IV, Random, 95% CI**
Bteich 2023	45.66	5.04	52	48.92	4.49	52	16.6%	-3.26 [-5.09, -1.43]
Bteich 2024	46.28	3.62	20	49.3	4.83	20		Not estimable
Haciagaoglu 2022	45.72	4.85	44	49.01	5.11	44	15.9%	-3.29 [-5.37, -1.21]
Keskin Perk 2025	46.22	4.98	62	49.17	4.97	62	16.8%	-2.95 [-4.70, -1.20]
Kirgiz 2024	42.91	1.32	23	49.86	1.16	23	19.2%	-6.95 [-7.67, -6.23]
Mechleb 2024 (Kmax inf 75D)	44.925	3.91	6	49.665	3.16	6		Not estimable
Mechleb 2024 (Kmax sup 75D)	55.325	5.36	4	68.01	2.215	4		Not estimable
Nacaroglu 2023	46.32	5.35	49	49.34	5.32	49	15.8%	-3.02 [-5.13, -0.91]
Yukecul 2024 (non-CXL)	44.52	4.8	37	49.11	4.66	37	15.7%	-4.59 [-6.75, -2.43]
Yukecul 2024 (prior CXL)	45.58	4.45	30	49.46	5.55	30		Not estimable
**Total (95% CI)**			**267**			**267**	**100.0%**	**-4.09 [-5.96, -2.22]**

Heterogeneity: Tau² = 4.62; Chi² = 39.84, df = 5 (P < 0.00001); I² = 87%
Test for overall effect: Z = 4.28 (P < 0.0001)

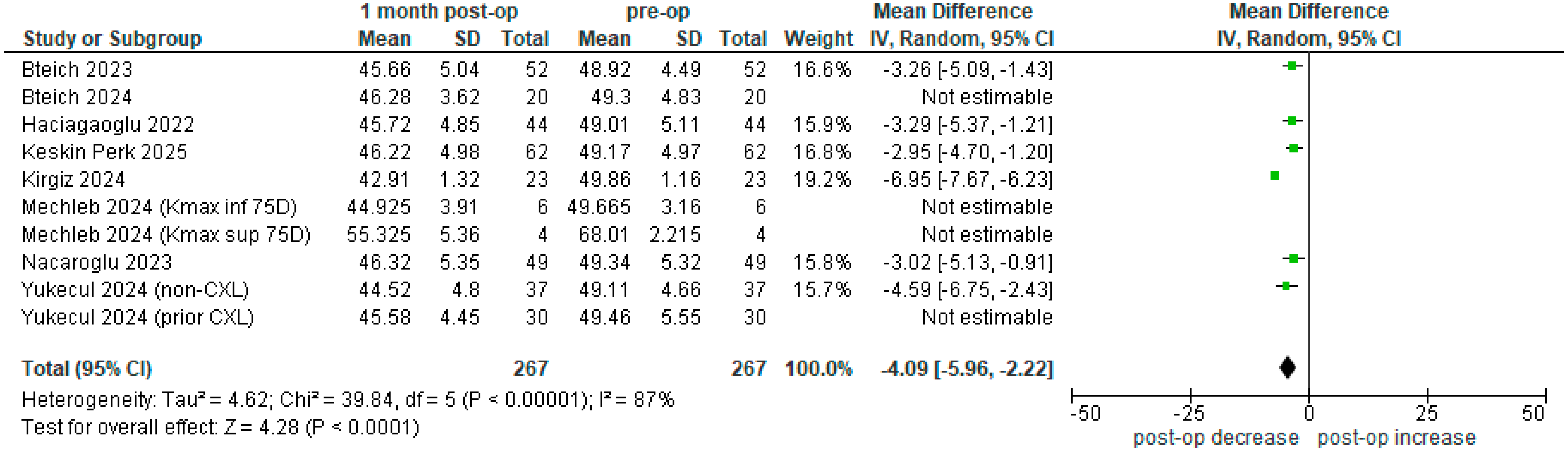

Figure S1.2.1.6B. Difference in mean simulated keratometry across 2 studies [2, 8]– FEMTOSECOND LASER

	1 month post-op			pre-op				Mean Difference
**Study or Subgroup**	**Mean**	**SD**	**Total**	**Mean**	**SD**	**Total**	**Weight**	**IV, Random, 95% CI**
Bteich 2023	45.66	5.04	52	48.92	4.49	52	0.0%	-3.26 [-5.09, -1.43]
Bteich 2024	46.28	3.62	20	49.3	4.83	20	38.7%	-3.02 [-5.67, -0.37]
Haciagaoglu 2022	45.72	4.85	44	49.01	5.11	44	0.0%	-3.29 [-5.37, -1.21]
Keskin Perk 2025	46.22	4.98	62	49.17	4.97	62	0.0%	-2.95 [-4.70, -1.20]
Kirgiz 2024	42.91	1.32	23	49.86	1.16	23	0.0%	-6.95 [-7.67, -6.23]
Mechleb 2024 (Kmax inf 75D)	44.925	3.91	6	49.665	3.16	6	33.7%	-4.74 [-8.76, -0.72]
Mechleb 2024 (Kmax sup 75D)	55.325	5.36	4	68.01	2.215	4	27.5%	-12.69 [-18.37, -7.00]
Nacaroglu 2023	46.32	5.35	49	49.34	5.32	49	0.0%	-3.02 [-5.13, -0.91]
Yukecul 2024 (non-CXL)	44.52	4.8	37	49.11	4.66	37	0.0%	-4.59 [-6.75, -2.43]
Yukecul 2024 (prior CXL)	45.58	4.45	30	49.46	5.55	30	0.0%	-3.88 [-6.43, -1.33]
**Total (95% CI)**			**30**			**30**	**100.0%**	**-6.26 [-11.18, -1.35]**

Heterogeneity: $Tau^2 = 14.42$; $Chi^2 = 9.13$, df = 2 (P = 0.01); $I^2 = 78\%$
Test for overall effect: Z = 2.50 (P = 0.01)

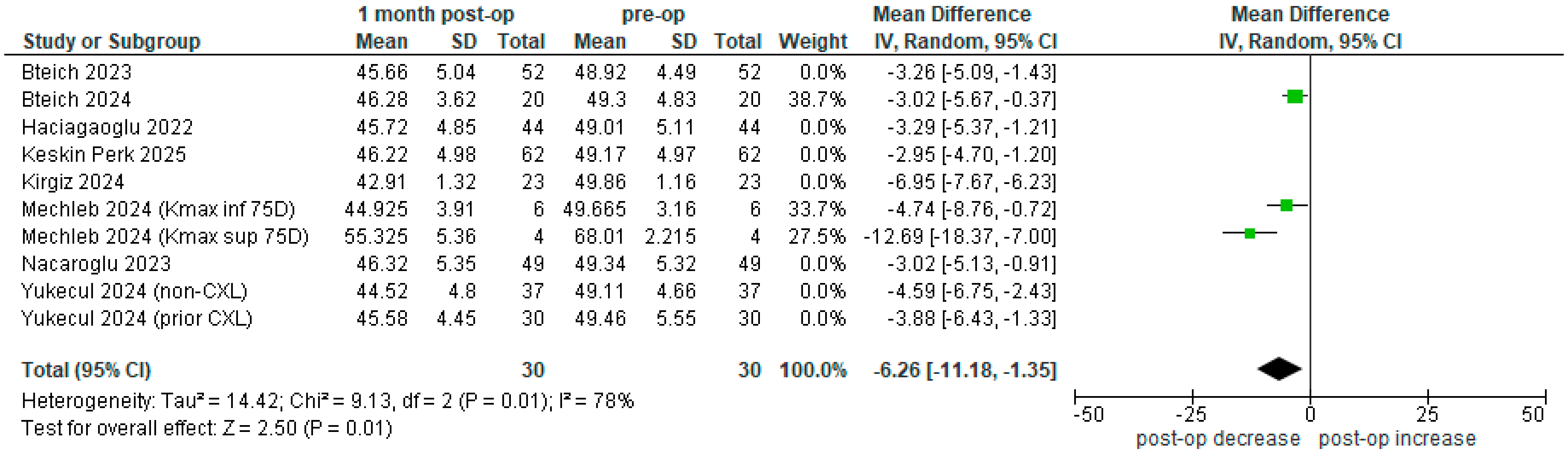

S1.2.1.7. Difference in total higher order aberrations

Figure S1.2.1.7A. Difference in total higher order aberrations across 2 studies [1, 2] - TREPHINE

	1 month post-op			pre-op				Mean Difference
**Study or Subgroup**	**Mean**	**SD**	**Total**	**Mean**	**SD**	**Total**	**Weight**	**IV, Random, 95% CI**
Bteich 2023	1.74	0.85	52	2.2	1.13	52	100.0%	-0.46 [-0.84, -0.08]
Bteich 2024	1.53	0.59	20	1.92	0.95	20	0.0%	-0.39 [-0.88, 0.10]
**Total (95% CI)**			**52**			**52**	**100.0%**	**-0.46 [-0.84, -0.08]**

Heterogeneity: Not applicable
Test for overall effect: Z = 2.35 (P = 0.02)

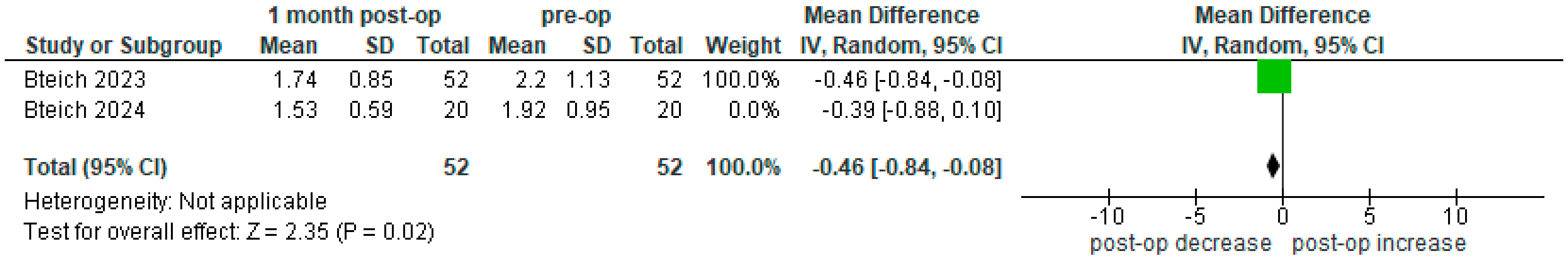

Figure S1.2.1.7B. Difference in total higher order aberrations across 1 study [2] – FEMTOSECOND LASER

	1 month post-op			pre-op				Mean Difference
**Study or Subgroup**	**Mean**	**SD**	**Total**	**Mean**	**SD**	**Total**	**Weight**	**IV, Random, 95% CI**
Bteich 2023	1.74	0.85	52	2.2	1.13	52	0.0%	-0.46 [-0.84, -0.08]
Bteich 2024	1.53	0.59	20	1.92	0.95	20	100.0%	-0.39 [-0.88, 0.10]
**Total (95% CI)**			**20**			**20**	**100.0%**	**-0.39 [-0.88, 0.10]**

Heterogeneity: Not applicable
Test for overall effect: Z = 1.56 (P = 0.12)

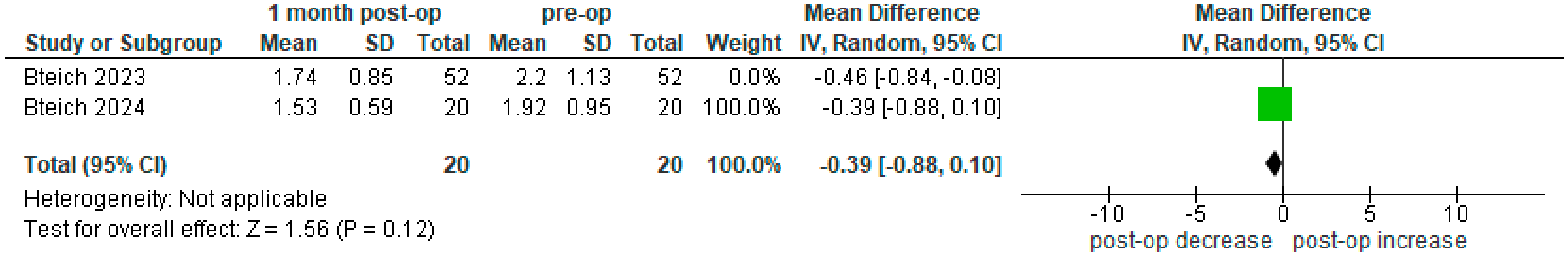

S1.2.1.8. Difference in spherical aberration

Figure S1.2.1.8A. Difference in spherical aberration across 2 studies [1, 5] - TREPHINE

	1 month post-op			pre-op				Mean Difference
**Study or Subgroup**	**Mean**	**SD**	**Total**	**Mean**	**SD**	**Total**	**Weight**	**IV, Random, 95% CI**
Bteich 2023	0.51	0.59	52	0.15	0.45	52	5.8%	0.36 [0.16, 0.56]
Bteich 2024	0.31	0.39	20	0.05	0.42	20	0.0%	0.26 [0.01, 0.51]
Kirgiz 2024	0.8	0.1	23	0.38	0.07	23	94.2%	0.42 [0.37, 0.47]
Mechleb 2024 (Kmax inf 75D)	0.13	0.5	6	-0.94	0.75	6	0.0%	1.07 [0.35, 1.79]
Mechleb 2024 (Kmax sup 75D)	-1.35	0.69	4	-4.76	1.73	4	0.0%	3.41 [1.58, 5.24]
**Total (95% CI)**			**75**			**75**	**100.0%**	**0.42 [0.37, 0.46]**

Heterogeneity: $Tau^2 = 0.00$; $Chi^2 = 0.32$, df = 1 (P = 0.57); $I^2 = 0\%$
Test for overall effect: Z = 16.86 (P < 0.00001)

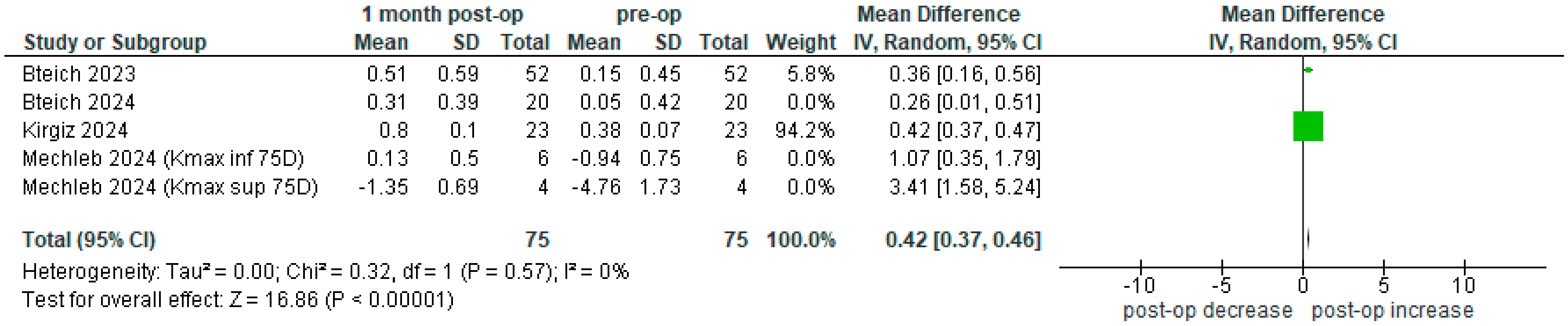

Figure S1.2.1.8B. Difference in spherical aberration across 2 studies [2, 8] – FEMTOSECOND LASER

	1 month post-op			pre-op				Mean Difference	Mean Difference
**Study or Subgroup**	**Mean**	**SD**	**Total**	**Mean**	**SD**	**Total**	**Weight**	**IV, Random, 95% CI**	**IV, Random, 95% CI**
Bteich 2023	0.51	0.59	52	0.15	0.45	52	0.0%	0.36 [0.16, 0.56]	
Bteich 2024	0.31	0.39	20	0.05	0.42	20	41.9%	0.26 [0.01, 0.51]	
Kirgiz 2024	0.8	0.1	23	0.38	0.07	23	0.0%	0.42 [0.37, 0.47]	
Mechleb 2024 (Kmax inf 75D)	0.13	0.5	6	-0.94	0.75	6	36.9%	1.07 [0.35, 1.79]	
Mechleb 2024 (Kmax sup 75D)	-1.35	0.69	4	-4.76	1.73	4	21.2%	3.41 [1.58, 5.24]	
**Total (95% CI)**			**30**			**30**	**100.0%**	**1.23 [0.04, 2.41]**	

Heterogeneity: $Tau^2 = 0.85$; $Chi^2 = 14.96$, df = 2 (P = 0.0006); $I^2 = 87\%$
Test for overall effect: Z = 2.03 (P = 0.04)

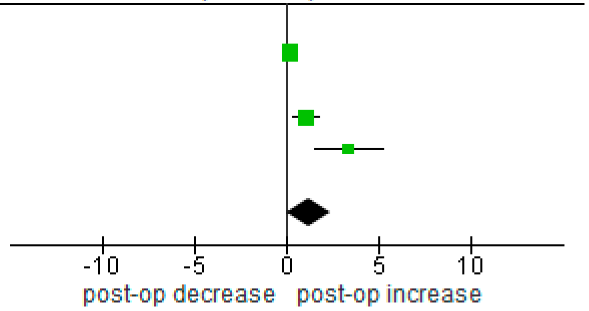

S1.2.1.9. Difference in vertical coma

Figure S1.2.1.9A. Difference in vertical coma in one study [1] - TREPHINE

	1 month post-op			pre-op				Mean Difference
**Study or Subgroup**	**Mean**	**SD**	**Total**	**Mean**	**SD**	**Total**	**Weight**	**IV, Random, 95% CI**
Bteich 2023	0.44	0.76	52	1.56	1.09	52	100.0%	-1.12 [-1.48, -0.76]
Bteich 2024	0.6	0.88	20	1.44	1.09	20	0.0%	-0.84 [-1.45, -0.23]
Mechleb 2024 (Kmax inf 75D)	1.16	1.19	6	2.03	0.52	6	0.0%	-0.87 [-1.91, 0.17]
Mechleb 2024 (Kmax sup 75D)	3.2	1.33	4	2.25	0.59	4	0.0%	0.95 [-0.48, 2.38]
**Total (95% CI)**			**52**			**52**	**100.0%**	**-1.12 [-1.48, -0.76]**

Heterogeneity: Not applicable
Test for overall effect: Z = 6.08 (P < 0.00001)

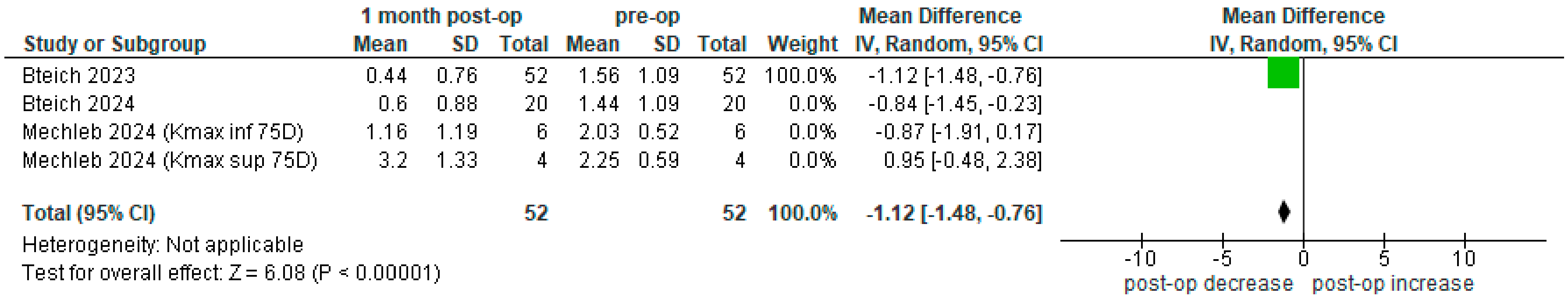

Figure S1.2.1.9B. Difference in vertical coma across 2 studies [2, 19]– FEMTOSECOND LASER

	1 month post-op			pre-op				Mean Difference
**Study or Subgroup**	**Mean**	**SD**	**Total**	**Mean**	**SD**	**Total**	**Weight**	**IV, Random, 95% CI**
Bteich 2023	0.44	0.76	52	1.56	1.09	52	0.0%	-1.12 [-1.48, -0.76]
Bteich 2024	0.6	0.88	20	1.44	1.09	20	43.7%	-0.84 [-1.45, -0.23]
Mechleb 2024 (Kmax inf 75D)	1.16	1.19	6	2.03	0.52	6	32.4%	-0.87 [-1.91, 0.17]
Mechleb 2024 (Kmax sup 75D)	3.2	1.33	4	2.25	0.59	4	23.9%	0.95 [-0.48, 2.38]
**Total (95% CI)**			**30**			**30**	**100.0%**	**-0.42 [-1.36, 0.51]**

Heterogeneity: Tau² = 0.42; Chi² = 5.37, df = 2 (P = 0.07); I² = 63%
Test for overall effect: Z = 0.88 (P = 0.38)

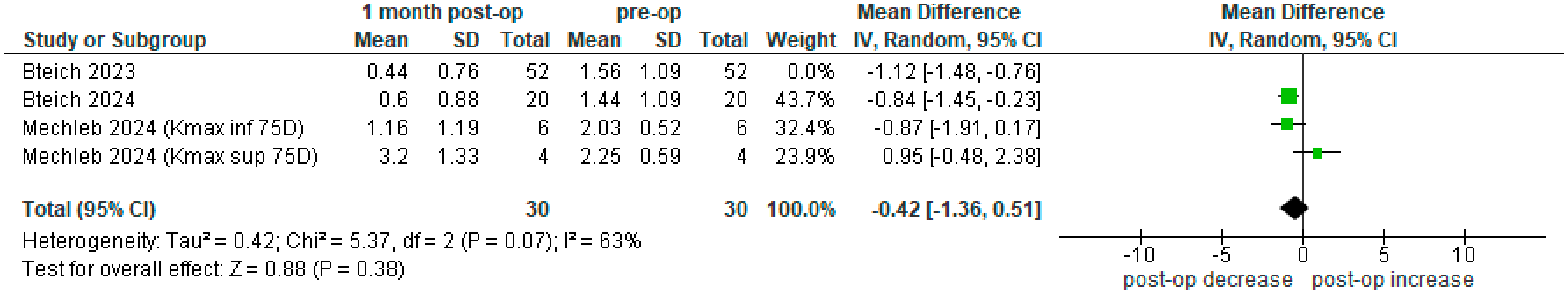

S1.2.1.10. Difference in horizontal coma

Figure S1.2.1.10. A. Difference in horizontal coma across 1 study [1] - TREPHINE

	1 month post-op			pre-op				Mean Difference
**Study or Subgroup**	**Mean**	**SD**	**Total**	**Mean**	**SD**	**Total**	**Weight**	**IV, Random, 95% CI**
Bteich 2023	-0.03	0.73	52	0.11	0.97	52	100.0%	-0.14 [-0.47, 0.19]
**Total (95% CI)**			**52**			**52**	**100.0%**	**-0.14 [-0.47, 0.19]**
Heterogeneity: Not applicable								
Test for overall effect: Z = 0.83 (P = 0.41)								

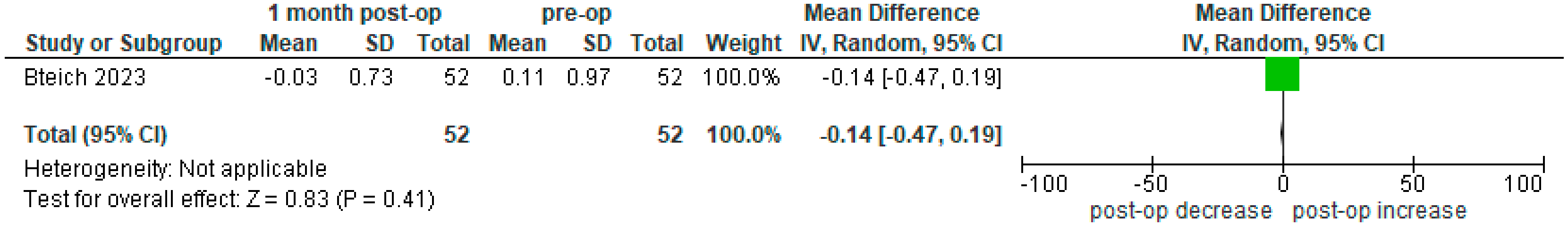

B.1.10. B. Difference in horizontal coma across 0 studies – FEMTOSECOND LASER

S.1.2.1.10. Difference in trefoil

Figure S1.2.1.10A. Difference in trefoil across 2 studies [1, 5] – TREPHINE

	1 month post-op			pre-op				Mean Difference
**Study or Subgroup**	**Mean**	**SD**	**Total**	**Mean**	**SD**	**Total**	**Weight**	**IV, Random, 95% CI**
Bteich 2023	-0.12	0.64	52	-0.39	0.52	52	33.5%	0.27 [0.05, 0.49]
Bteich 2024	0.088	0.59	20	-0.27	0.4	20	0.0%	0.36 [0.05, 0.67]
Kirgiz 2024	1.12	0.1	23	0.66	0.13	23	66.5%	0.46 [0.39, 0.53]
**Total (95% CI)**			**75**			**75**	**100.0%**	**0.40 [0.22, 0.57]**

Heterogeneity: Tau² = 0.01; Chi² = 2.53, df = 1 (P = 0.11); I² = 61%
Test for overall effect: Z = 4.42 (P < 0.00001)

Mean Difference IV, Random, 95% CI

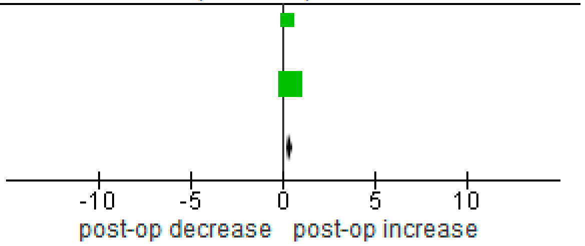

Figure S1.2.1.10B. Difference in trefoil across 1 study [2] – FEMTOSECOND LASER

	1 month post-op			pre-op				Mean Difference
**Study or Subgroup**	**Mean**	**SD**	**Total**	**Mean**	**SD**	**Total**	**Weight**	**IV, Random, 95% CI**
Bteich 2023	-0.12	0.64	52	-0.39	0.52	52	0.0%	0.27 [0.05, 0.49]
Bteich 2024	0.088	0.59	20	-0.27	0.4	20	100.0%	0.36 [0.05, 0.67]
Kirgiz 2024	1.12	0.1	23	0.66	0.13	23	0.0%	0.46 [0.39, 0.53]
**Total (95% CI)**			**20**			**20**	**100.0%**	**0.36 [0.05, 0.67]**

Heterogeneity: Not applicable
Test for overall effect: Z = 2.25 (P = 0.02)

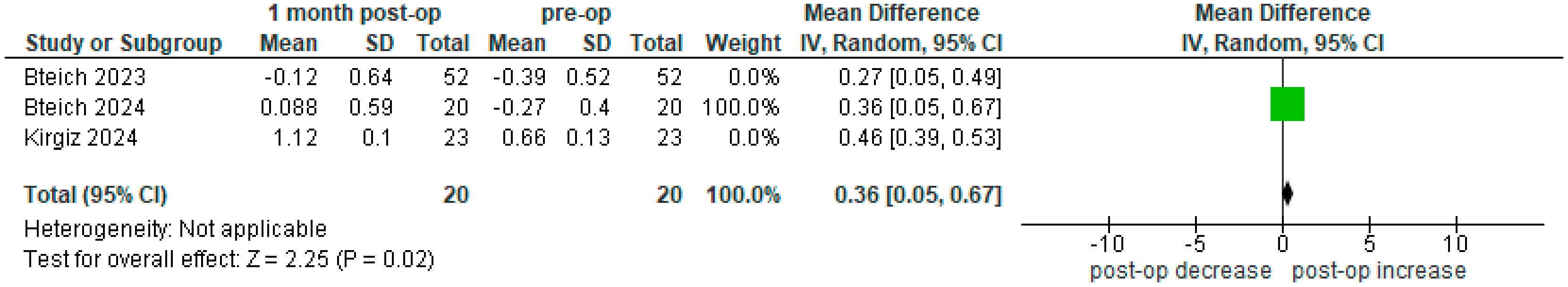

S1.2.1.11. Difference in total RMS

Figure S1.2.1.11A. Difference in total RMS across 1 study [5] – TREPHINE

	1 month post-op			pre-op				Mean Difference
**Study or Subgroup**	**Mean**	**SD**	**Total**	**Mean**	**SD**	**Total**	**Weight**	**IV, Random, 95% CI**
Kirgiz 2024	2.21	0.15	23	2.35	0.3	23	100.0%	-0.14 [-0.28, -0.00]
Mechleb 2024 (Kmax inf 75D)	8.45	5.87	6	14.1	4.04	6	0.0%	-5.65 [-11.35, 0.05]
Mechleb 2024 (Kmax sup 75D)	20.33	4.94	4	29.78	7.59	4	0.0%	-9.45 [-18.32, -0.58]
**Total (95% CI)**			**23**			**23**	**100.0%**	**-0.14 [-0.28, -0.00]**
Heterogeneity: Not applicable								
Test for overall effect: Z = 2.00 (P = 0.05)								

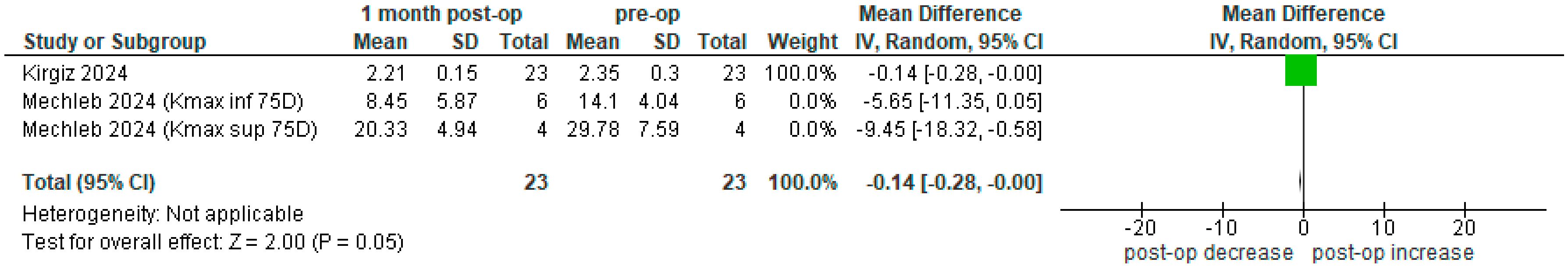

Figure S1.2.1.11B. Difference in total RMS across 1 study [8] – FEMTOSECOND LASER

	1 month post-op			pre-op				Mean Difference
**Study or Subgroup**	**Mean**	**SD**	**Total**	**Mean**	**SD**	**Total**	**Weight**	**IV, Random, 95% CI**
Kirgiz 2024	2.21	0.15	23	2.35	0.3	23	0.0%	-0.14 [-0.28, -0.00]
Mechleb 2024 (Kmax inf 75D)	8.45	5.87	6	14.1	4.04	6	70.8%	-5.65 [-11.35, 0.05]
Mechleb 2024 (Kmax sup 75D)	20.33	4.94	4	29.78	7.59	4	29.2%	-9.45 [-18.32, -0.58]
**Total (95% CI)**			**10**			**10**	**100.0%**	**-6.76 [-11.56, -1.96]**

Heterogeneity: Tau² = 0.00; Chi² = 0.50, df = 1 (P = 0.48); I² = 0%
Test for overall effect: Z = 2.76 (P = 0.006)

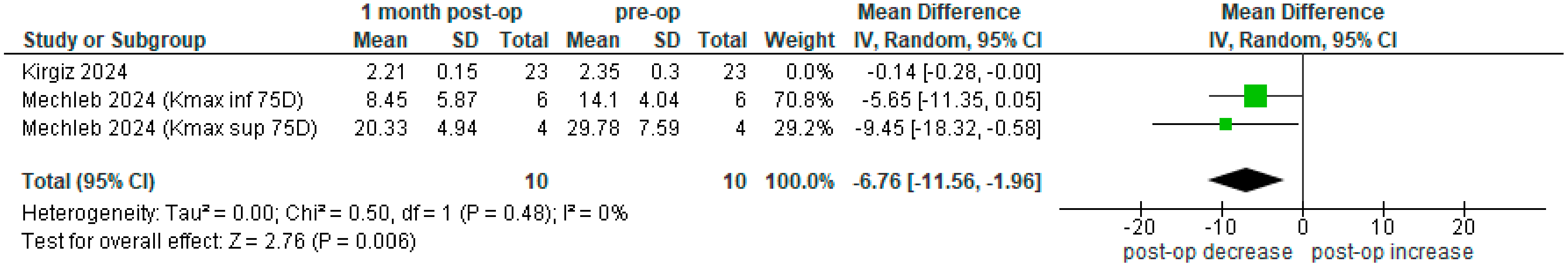

S1.2.2. 6 months postoperative versus preoperative

S1.2.2.1. Difference in uncorrected visual acuity

Figure S1.2.2.1A. Difference in uncorrected visual acuity across 8 studies [1, 3-7, 10, 11]- TREPHINE

	6 months post-op			pre-op				Mean Difference
Study or Subgroup	Mean	SD	Total	Mean	SD	Total	Weight	IV, Random, 95% CI
Bteich 2024	0.51	0.18	20	0.893	0.33	20		Not estimable
Mazzotta 2024	0.52	0	2	1	0	2		Not estimable
Bteich 2023 ther refr	0.68	0.23	4	0.93	0.31	4		Not estimable
Yukecul 2024 (prior CXL)	0.36	0.33	30	0.7	0.55	30		Not estimable
Hayashi 2025	0.68	0.54	5	1.39	0.28	5	4.7%	-0.71 [-1.24, -0.18]
Colak 2025	0.48	0.21	4	1.2	0.5	4	4.8%	-0.72 [-1.25, -0.19]
Bteich 2023	0.68	0.23	4	0.93	0.31	4	7.8%	-0.25 [-0.63, 0.13]
Yukecul 2024 (non-CXL)	0.31	0.21	37	0.77	0.76	37	12.1%	-0.46 [-0.71, -0.21]
Haciagaoglu 2022	0.35	0.29	44	0.72	0.63	44	14.4%	-0.37 [-0.57, -0.17]
Nacaroglu 2023	0.4	0.24	49	0.91	0.5	49	17.0%	-0.51 [-0.67, -0.35]
Keskin Perk 2025	0.44	0.34	62	0.96	0.5	62	17.2%	-0.52 [-0.67, -0.37]
Kirgiz 2024	0.398	0.05	23	1.096	0.05	23	22.0%	-0.70 [-0.73, -0.67]
**Total (95% CI)**			**228**			**228**	**100.0%**	**-0.53 [-0.66, -0.40]**

Heterogeneity: Tau² = 0.02; Chi² = 27.00, df = 7 (P = 0.0003); I² = 74%
Test for overall effect: Z = 7.88 (P < 0.00001)

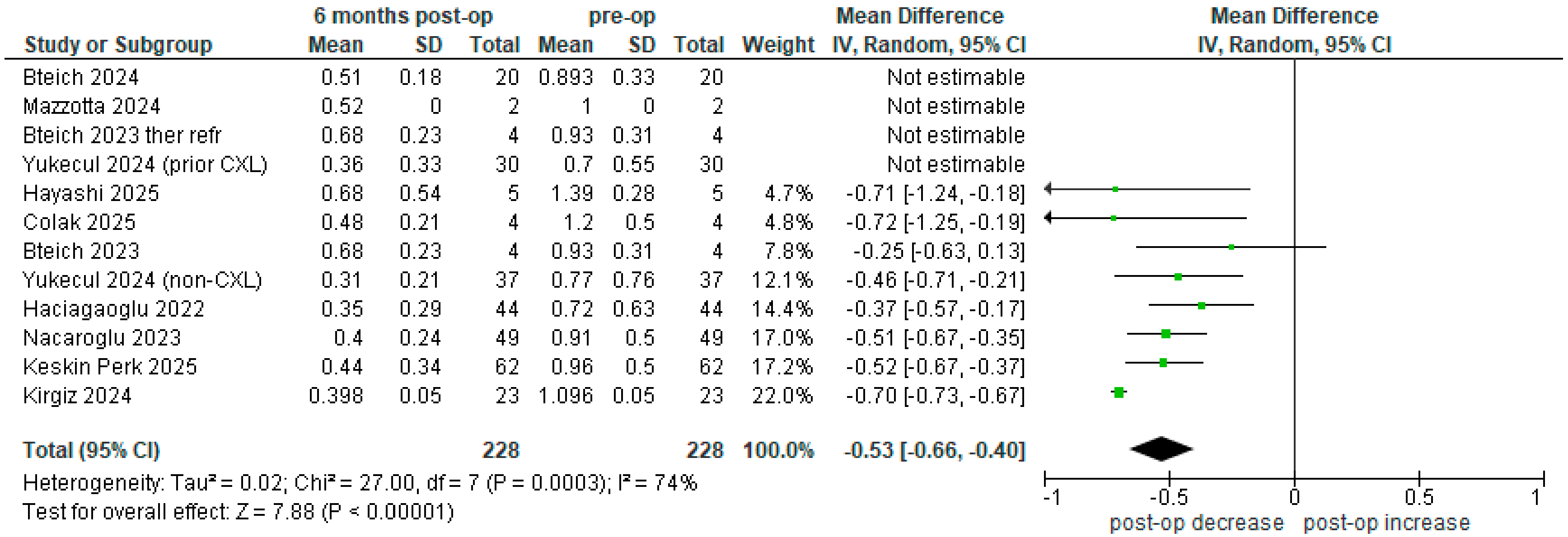

Figure S1.2.2.1B. Difference in uncorrected visual acuity across 2 studies [2, 12]– FEMTOSECOND LASER

	6 months post-op			pre-op				Mean Difference
**Study or Subgroup**	**Mean**	**SD**	**Total**	**Mean**	**SD**	**Total**	**Weight**	**IV, Random, 95% CI**
Bteich 2023	0.68	0.23	4	0.93	0.31	4	0.0%	-0.25 [-0.63, 0.13]
Bteich 2023 ther refr	0.68	0.23	4	0.93	0.31	4	15.9%	-0.25 [-0.63, 0.13]
Bteich 2024	0.51	0.18	20	0.893	0.33	20	84.1%	-0.38 [-0.55, -0.22]
Colak 2025	0.48	0.21	4	1.2	0.5	4	0.0%	-0.72 [-1.25, -0.19]
Haciagaoglu 2022	0.35	0.29	44	0.72	0.63	44	0.0%	-0.37 [-0.57, -0.17]
Hayashi 2025	0.68	0.54	5	1.39	0.28	5	0.0%	-0.71 [-1.24, -0.18]
Keskin Perk 2025	0.44	0.34	62	0.96	0.5	62	0.0%	-0.52 [-0.67, -0.37]
Kirgiz 2024	0.398	0.05	23	1.096	0.05	23	0.0%	-0.70 [-0.73, -0.67]
Mazzotta 2024	0.52	0	2	1	0	2		Not estimable
Nacaroglu 2023	0.4	0.24	49	0.91	0.5	49	0.0%	-0.51 [-0.67, -0.35]
Yukecul 2024 (non-CXL)	0.31	0.21	37	0.77	0.76	37	0.0%	-0.46 [-0.71, -0.21]
Yukecul 2024 (prior CXL)	0.36	0.33	30	0.7	0.55	30	0.0%	-0.34 [-0.57, -0.11]
**Total (95% CI)**			**24**			**24**	**100.0%**	**-0.36 [-0.51, -0.21]**

Heterogeneity: $Tau^2 = 0.00$; $Chi^2 = 0.40$, df = 1 (P = 0.53); $I^2 = 0\%$
Test for overall effect: Z = 4.69 (P < 0.00001)

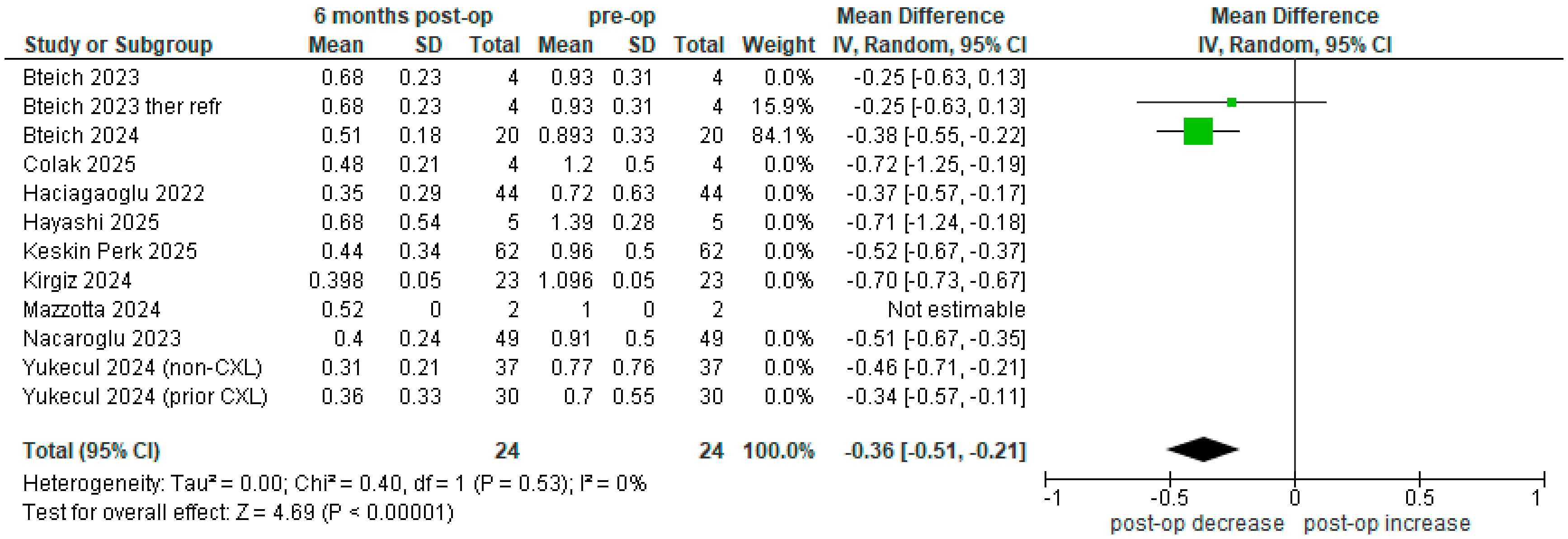

S1.2.2.2. Difference in best corrected visual acuity

Figure S1.2.2.2A. Difference in best corrected visual acuity across 7 studies [3-7, 10, 11]– TREPHINE

	6 months post-op			pre-op				Mean Difference
**Study or Subgroup**	**Mean**	**SD**	**Total**	**Mean**	**SD**	**Total**	**Weight**	**IV, Random, 95% CI**
Bteich 2023 ther refr	0.01	0.02	4	0.34	0.16	4		Not estimable
Bteich 2024	0.19	0.21	20	0.57	0.18	20		Not estimable
Colak 2025	0.4	0.14	4	0.53	0.13	4	12.8%	-0.13 [-0.32, 0.06]
Coscarelli 2024	0.301	0	3	1.06	0.22	3		Not estimable
Haciagaoglu 2022	0.25	0.22	44	0.54	0.37	44	15.6%	-0.29 [-0.42, -0.16]
Hayashi 2025	0.17	0.16	5	0.74	0.44	5	5.6%	-0.57 [-0.98, -0.16]
Keskin Perk 2025	0.25	0.24	62	0.72	0.47	62	15.4%	-0.47 [-0.60, -0.34]
Kirgiz 2024	0.167	0.05	23	0.602	0.3	23	15.8%	-0.43 [-0.56, -0.31]
Mechleb 2025	0.17	0.18	33	0.36	0.25	79		Not estimable
Nacaroglu 2023	0.31	0.09	49	0.87	0.2	49	18.3%	-0.56 [-0.62, -0.50]
Yukecul 2024 (non-CXL)	0.17	0.18	37	0.51	0.29	37	16.4%	-0.34 [-0.45, -0.23]
Yukecul 2024 (prior CXL)	0.27	0.23	30	0.55	0.39	30		Not estimable
**Total (95% CI)**			**227**			**227**	**100.0%**	**-0.39 [-0.51, -0.28]**

Heterogeneity: $Tau^2 = 0.02$; $Chi^2 = 33.92$, df = 6 ($P < 0.00001$); $I^2 = 82\%$
Test for overall effect: Z = 6.71 ($P < 0.00001$)

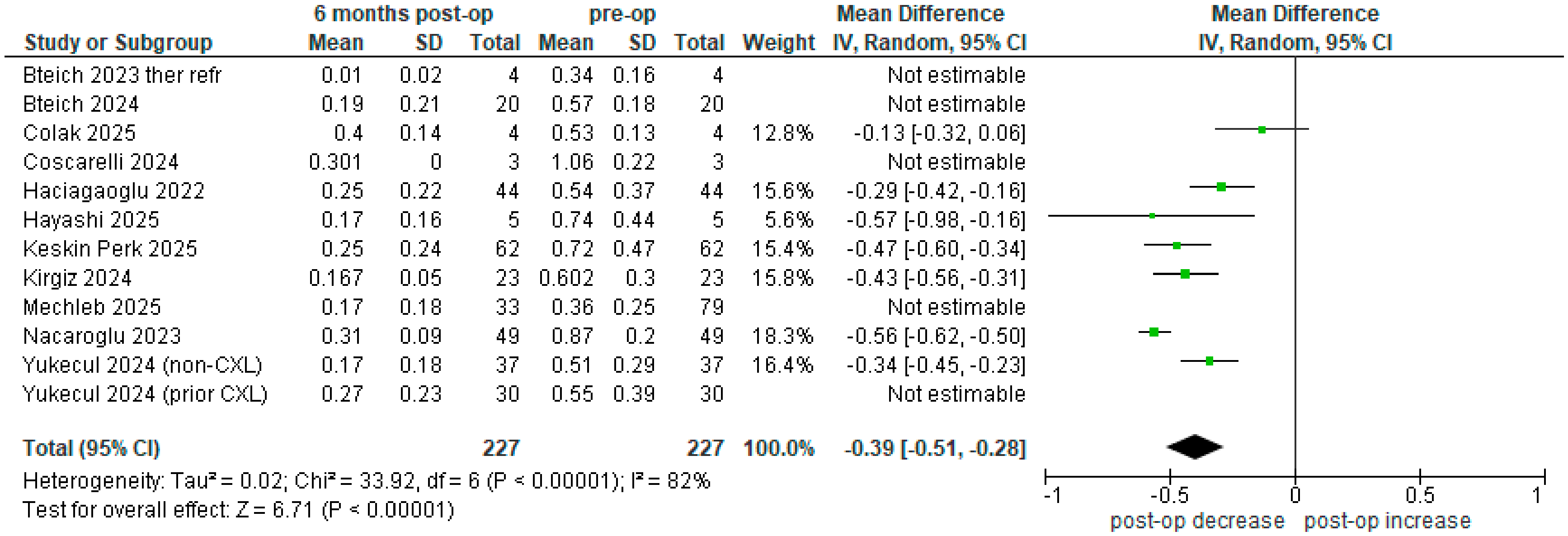

Figure S1.2.2.2B Difference in best corrected visual acuity across 3 studies [2, 12, 14]–FEMTOSECOND LASER

	6 months post-op			pre-op				Mean Difference
**Study or Subgroup**	**Mean**	**SD**	**Total**	**Mean**	**SD**	**Total**	**Weight**	**IV, Random, 95% CI**
Bteich 2023 ther refr	0.01	0.02	4	0.34	0.16	4	27.6%	-0.33 [-0.49, -0.17]
Bteich 2024	0.19	0.21	20	0.57	0.18	20	33.2%	-0.38 [-0.50, -0.26]
Colak 2025	0.4	0.14	4	0.53	0.13	4	0.0%	-0.13 [-0.32, 0.06]
Coscarelli 2024	0.301	0	3	1.06	0.22	3		Not estimable
Haciagaoglu 2022	0.25	0.22	44	0.54	0.37	44	0.0%	-0.29 [-0.42, -0.16]
Hayashi 2025	0.17	0.16	5	0.74	0.44	5	0.0%	-0.57 [-0.98, -0.16]
Keskin Perk 2025	0.25	0.24	62	0.72	0.47	62	0.0%	-0.47 [-0.60, -0.34]
Kirgiz 2024	0.167	0.05	23	0.602	0.3	23	0.0%	-0.43 [-0.56, -0.31]
Mechleb 2025	0.17	0.18	33	0.36	0.25	79	39.2%	-0.19 [-0.27, -0.11]
Nacaroglu 2023	0.31	0.09	49	0.87	0.2	49	0.0%	-0.56 [-0.62, -0.50]
Yukecul 2024 (non-CXL)	0.17	0.18	37	0.51	0.29	37	0.0%	-0.34 [-0.45, -0.23]
Yukecul 2024 (prior CXL)	0.27	0.23	30	0.55	0.39	30	0.0%	-0.28 [-0.44, -0.12]
**Total (95% CI)**			**57**			**103**	**100.0%**	**-0.29 [-0.42, -0.16]**

Heterogeneity: Tau² = 0.01; Chi² = 7.28, df = 2 (P = 0.03); I² = 73%
Test for overall effect: Z = 4.40 (P < 0.0001)

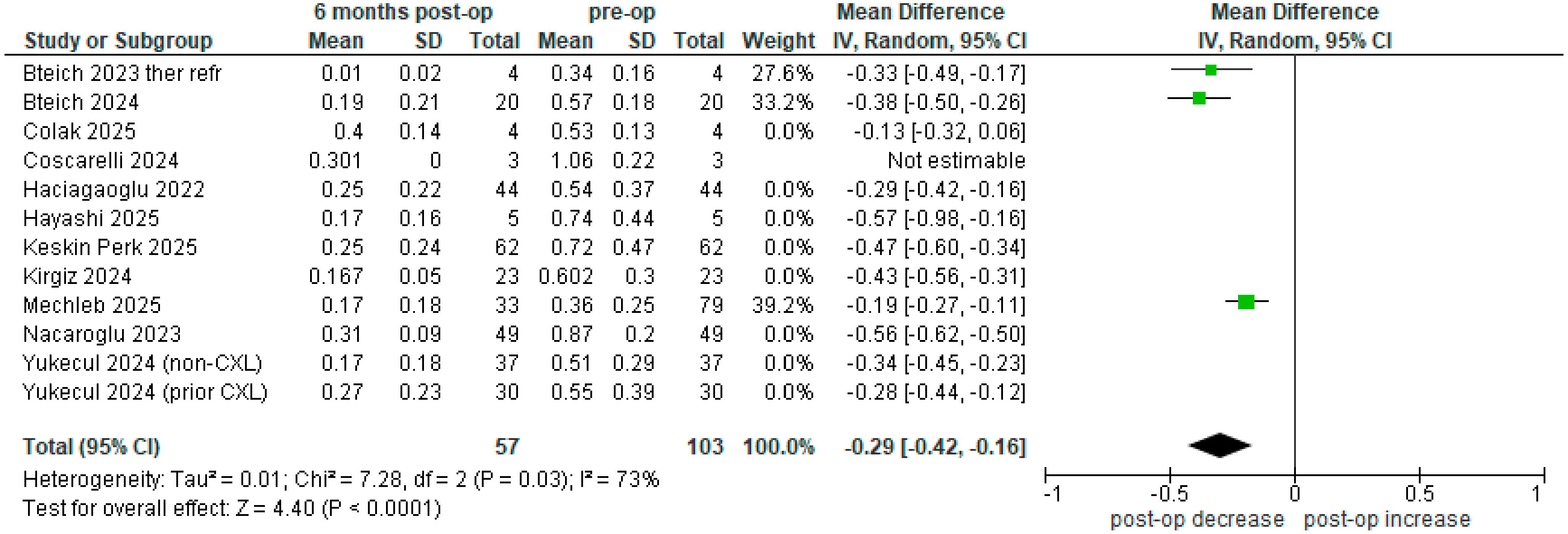

S1.2.2.3. Difference in pachymetry thinnest point

Figure S1.2.2.3A. Difference in pachymetry thinnest point across 4 studies [3-5, 7]- TREPHINE

	6 months post-op			pre-op				Mean Difference
**Study or Subgroup**	**Mean**	**SD**	**Total**	**Mean**	**SD**	**Total**	**Weight**	**IV, Random, 95% CI**
Bteich 2024	447.5	51.5	20	442.8	38.1	20		Not estimable
Haciagaoglu 2022	449.48	61.06	44	453.73	45.87	44	8.8%	-4.25 [-26.82, 18.32]
Keskin Perk 2025	445.7	47.35	62	449.68	49.48	62	15.5%	-3.98 [-21.03, 13.07]
Kirgiz 2024	445.5	17.5	23	439.33	10.43	23	64.8%	6.17 [-2.16, 14.50]
Mazzotta 2024	432	76.37	2	402.5	57.28	2		Not estimable
Yukecul 2024 (non-CXL)	446	44	37	454	45	37	10.9%	-8.00 [-28.28, 12.28]
Yukecul 2024 (prior CXL)	452	41	30	437	38	30		Not estimable
**Total (95% CI)**			**166**			**166**	**100.0%**	**2.13 [-4.57, 8.84]**

Heterogeneity: Tau² = 0.00; Chi² = 2.66, df = 3 (P = 0.45); I² = 0%
Test for overall effect: Z = 0.62 (P = 0.53)

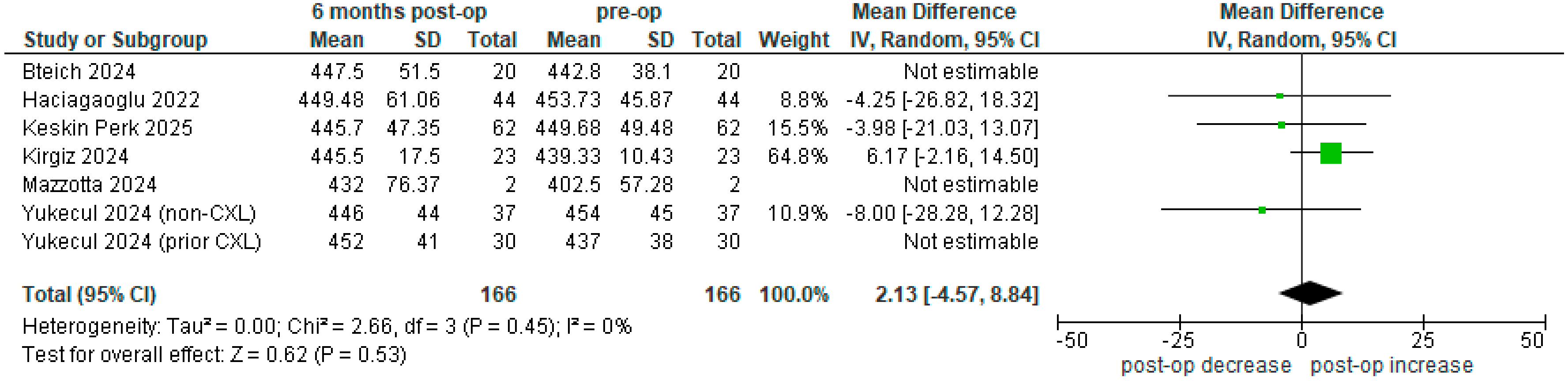

Figure S1.2.2.3B. Difference in pachymetry thinnest point across 2 studies [2, 9]– FEMTOSECOND LASER

	6 months post-op			pre-op				Mean Difference
**Study or Subgroup**	**Mean**	**SD**	**Total**	**Mean**	**SD**	**Total**	**Weight**	**IV, Random, 95% CI**
Bteich 2024	447.5	51.5	20	442.8	38.1	20	95.7%	4.70 [-23.38, 32.78]
Haciagaoglu 2022	449.48	61.06	44	453.73	45.87	44	0.0%	-4.25 [-26.82, 18.32]
Keskin Perk 2025	445.7	47.35	62	449.68	49.48	62	0.0%	-3.98 [-21.03, 13.07]
Kirgiz 2024	445.5	17.5	23	439.33	10.43	23	0.0%	6.17 [-2.16, 14.50]
Mazzotta 2024	432	76.37	2	402.5	57.28	2	4.3%	29.50 [-102.80, 161.80]
Yukecul 2024 (non-CXL)	446	44	37	454	45	37	0.0%	-8.00 [-28.28, 12.28]
Yukecul 2024 (prior CXL)	452	41	30	437	38	30		Not estimable
**Total (95% CI)**			**22**			**22**	**100.0%**	**5.77 [-21.70, 33.23]**

Heterogeneity: $Tau^2 = 0.00$; $Chi^2 = 0.13$, df = 1 (P = 0.72); $I^2 = 0\%$
Test for overall effect: Z = 0.41 (P = 0.68)

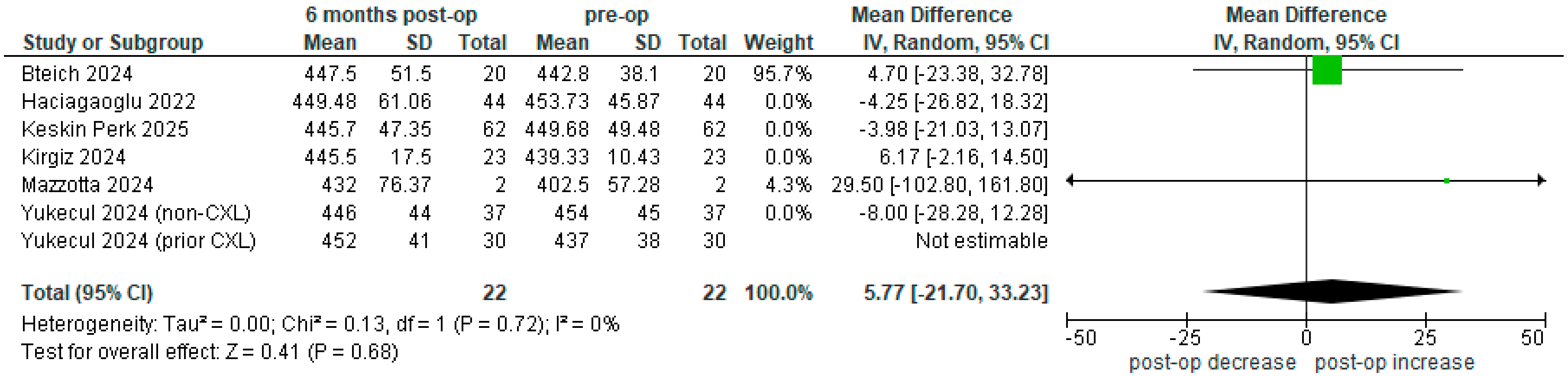

S1.2.2.4. Difference in pachymetry central point

Figure S1.2.2.4A. Difference in pachymetry central point across 3 studies [6, 10, 11]– TREPHINE

	6 months post-op			pre-op				Mean Difference
**Study or Subgroup**	**Mean**	**SD**	**Total**	**Mean**	**SD**	**Total**	**Weight**	**IV, Random, 95% CI**
Colak 2025	452.5	47.03	4	417.25	40.73	4	19.2%	35.25 [-25.72, 96.22]
Hayashi 2025	514.6	62.63	5	505.6	55.4	5	14.1%	9.00 [-64.29, 82.29]
Mechleb 2025	466	39	33	455	41	79		Not estimable
Nacaroglu 2023	447.46	50.57	49	463.62	54.88	49	66.7%	-16.16 [-37.06, 4.74]
**Total (95% CI)**			**58**			**58**	**100.0%**	**-2.76 [-32.46, 26.94]**

Heterogeneity: Tau² = 230.38; Chi² = 2.70, df = 2 (P = 0.26); I² = 26%
Test for overall effect: Z = 0.18 (P = 0.86)

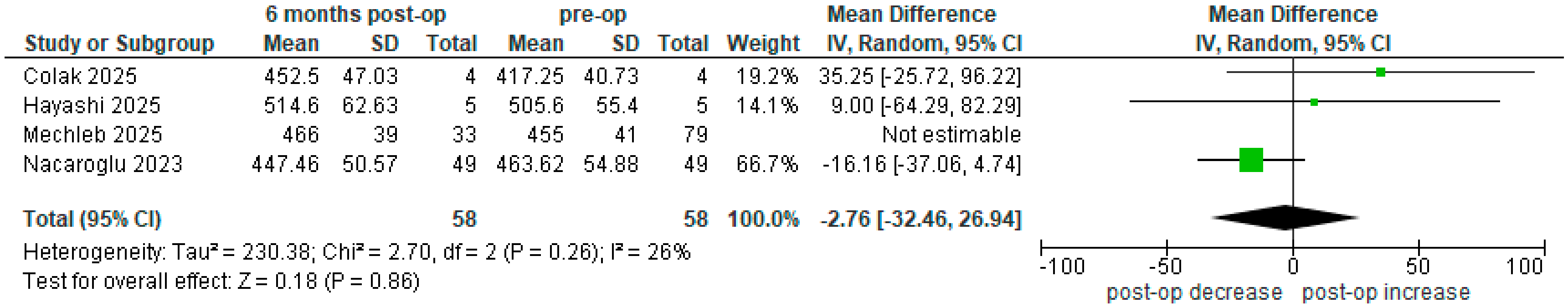

Figure S1.2.2.4B Difference in pachymetry central point across 1 study [14] – FEMTOSECOND LASER

	6 months post-op			pre-op				Mean Difference
**Study or Subgroup**	**Mean**	**SD**	**Total**	**Mean**	**SD**	**Total**	**Weight**	**IV, Random, 95% CI**
Colak 2025	452.5	47.03	4	417.25	40.73	4	0.0%	35.25 [-25.72, 96.22]
Hayashi 2025	514.6	62.63	5	505.6	55.4	5	0.0%	9.00 [-64.29, 82.29]
Mechleb 2025	466	39	33	455	41	79	100.0%	11.00 [-5.09, 27.09]
Nacaroglu 2023	447.46	50.57	49	463.62	54.88	49	0.0%	-16.16 [-37.06, 4.74]
**Total (95% CI)**			**33**			**79**	**100.0%**	**11.00 [-5.09, 27.09]**

Heterogeneity: Not applicable
Test for overall effect: Z = 1.34 (P = 0.18)

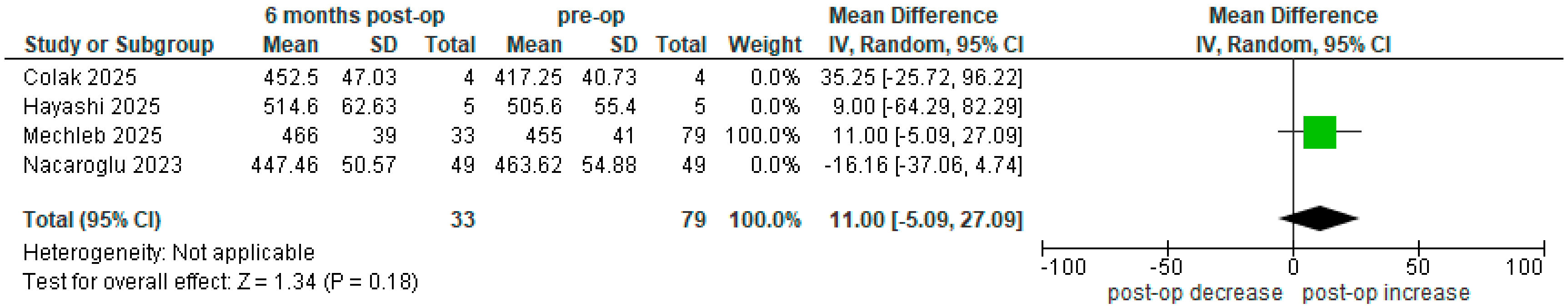

S1.2.2.5. Difference in maximum keratometry

Figure S1.2.2.5A. Difference in maximum keratometry across 7 studies [3-7, 10, 11]– TREPHINE

	6 months post-op			pre-op				Mean Difference
**Study or Subgroup**	**Mean**	**SD**	**Total**	**Mean**	**SD**	**Total**	**Weight**	**IV, Random, 95% CI**
Bteich 2023 ther refr	47.89	3.05	4	50.02	1.99	4		Not estimable
Bteich 2024	52.38	6.02	20	57.21	7.53	20		Not estimable
Colak 2025	55.88	3.64	4	56.55	3.31	4	13.4%	-0.67 [-5.49, 4.15]
Haciagaoglu 2022	55.4	6.73	44	57.56	7.06	44	15.3%	-2.16 [-5.04, 0.72]
Hayashi 2025	59.62	6.78	5	63.58	8.26	5	8.7%	-3.96 [-13.33, 5.41]
Keskin Perk 2025	55.27	6.17	62	58.51	7.55	62	15.7%	-3.24 [-5.67, -0.81]
Kirgiz 2024	46.96	1.88	23	57.65	1.41	23	16.5%	-10.69 [-11.65, -9.73]
Mazzotta 2024	50.81	4.53	2	56.21	8.78	2		Not estimable
Mechleb 2025	55.6	6.4	33	57.6	6.9	79		Not estimable
Nacaroglu 2023	55.83	6.96	49	59.2	7.66	49	15.3%	-3.37 [-6.27, -0.47]
Yukecul 2024 (non-CXL)	55.66	6.29	37	58.38	7.24	37	15.1%	-2.72 [-5.81, 0.37]
Yukecul 2024 (prior CXL)	54.11	6.65	30	57.22	6.68	30		Not estimable
**Total (95% CI)**			**224**			**224**	**100.0%**	**-3.96 [-7.94, 0.01]**

Heterogeneity: $Tau^2 = 24.71$; $Chi^2 = 91.29$, $df = 6$ ($P < 0.00001$); $I^2 = 93\%$
Test for overall effect: $Z = 1.95$ ($P = 0.05$)

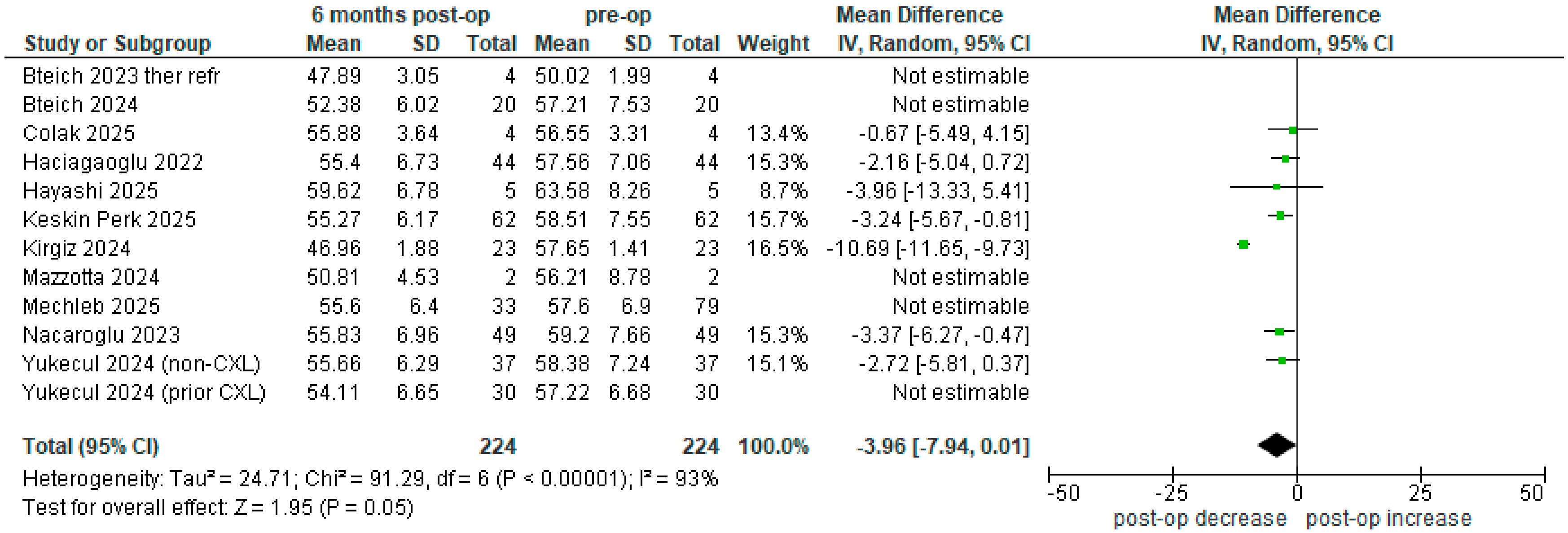

Figure S1.2.2.5B. Difference in maximum keratometry across 4 studies [2, 9, 12, 14] – FEMTOSECOND LASER

	6 months post-op			pre-op				Mean Difference
**Study or Subgroup**	**Mean**	**SD**	**Total**	**Mean**	**SD**	**Total**	**Weight**	**IV, Random, 95% CI**
Bteich 2023 ther refr	47.89	3.05	4	50.02	1.99	4	27.9%	-2.13 [-5.70, 1.44]
Bteich 2024	52.38	6.02	20	57.21	7.53	20	19.9%	-4.83 [-9.06, -0.60]
Colak 2025	55.88	3.64	4	56.55	3.31	4	0.0%	-0.67 [-5.49, 4.15]
Haciagaoglu 2022	55.4	6.73	44	57.56	7.06	44	0.0%	-2.16 [-5.04, 0.72]
Hayashi 2025	59.62	6.78	5	63.58	8.26	5	0.0%	-3.96 [-13.33, 5.41]
Keskin Perk 2025	55.27	6.17	62	58.51	7.55	62	0.0%	-3.24 [-5.67, -0.81]
Kirgiz 2024	46.96	1.88	23	57.65	1.41	23	0.0%	-10.69 [-11.65, -9.73]
Mazzotta 2024	50.81	4.53	2	56.21	8.78	2	1.9%	-5.40 [-19.09, 8.29]
Mechleb 2025	55.6	6.4	33	57.6	6.9	79	50.2%	-2.00 [-4.66, 0.66]
Nacaroglu 2023	55.83	6.96	49	59.2	7.66	49	0.0%	-3.37 [-6.27, -0.47]
Yukecul 2024 (non-CXL)	55.66	6.29	37	58.38	7.24	37	0.0%	-2.72 [-5.81, 0.37]
Yukecul 2024 (prior CXL)	54.11	6.65	30	57.22	6.68	30		Not estimable
**Total (95% CI)**			**59**			**105**	**100.0%**	**-2.66 [-4.55, -0.78]**

Heterogeneity: Tau² = 0.00; Chi² = 1.49, df = 3 (P = 0.69); I² = 0%

Test for overall effect: Z = 2.77 (P = 0.006)

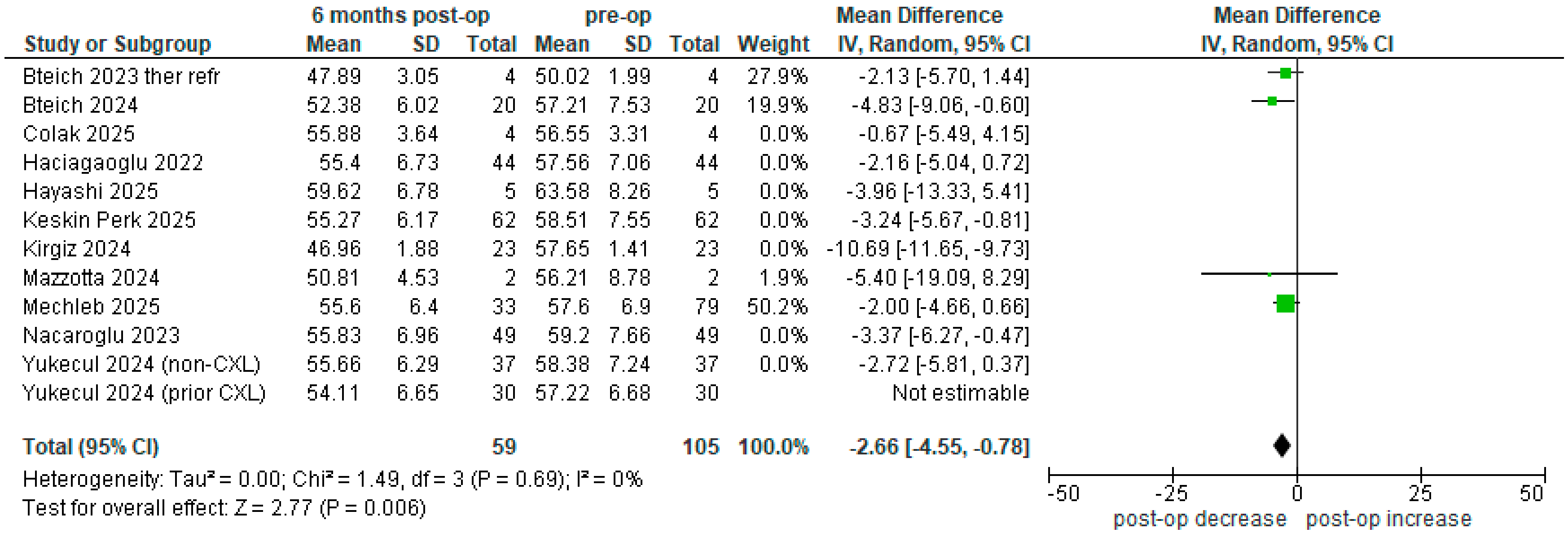

S1.2.2.6. Difference in mean simulated keratometry

Figure S1.2.2.6A Difference in mean simulated keratometry across 7 studies [3-7, 11, 13]– TREPHINE

	6 months post-op			pre-op				Mean Difference
**Study or Subgroup**	**Mean**	**SD**	**Total**	**Mean**	**SD**	**Total**	**Weight**	**IV, Random, 95% CI**
Bteich 2023 ther refr	43.18	1.52	4	44.32	0.95	4		Not estimable
Bteich 2024	45.45	3.57	20	49.3	4.83	20		Not estimable
Colak 2025	45.88	3.27	4	49.05	3.44	4	9.3%	-3.17 [-7.82, 1.48]
Coscarelli 2024	44.28	0.87	3	55.78	2.84	3	12.0%	-11.50 [-14.86, -8.14]
Haciagaoglu 2022	45.71	4.8	44	49.01	5.11	44	15.1%	-3.30 [-5.37, -1.23]
Keskin Perk 2025	45.75	4.47	62	49.17	4.97	62	15.9%	-3.42 [-5.08, -1.76]
Kirgiz 2024	42.4	1.37	23	49.86	1.16	23	17.4%	-7.46 [-8.19, -6.73]
Mechleb 2025	45.3	5	33	49.2	6.5	79		Not estimable
Nacaroglu 2023	45.76	5.02	49	49.34	5.32	49	15.1%	-3.58 [-5.63, -1.53]
Yukecul 2024 (non-CXL)	44.45	4.2	37	49.11	4.66	37	15.2%	-4.66 [-6.68, -2.64]
Yukecul 2024 (prior CXL)	46.34	4.46	30	49.46	5.55	30		Not estimable
**Total (95% CI)**			**222**			**222**	**100.0%**	**-5.27 [-7.31, -3.22]**

Heterogeneity: Tau² = 6.11; Chi² = 49.28, df = 6 (P < 0.00001); I² = 88%
Test for overall effect: Z = 5.05 (P < 0.00001)

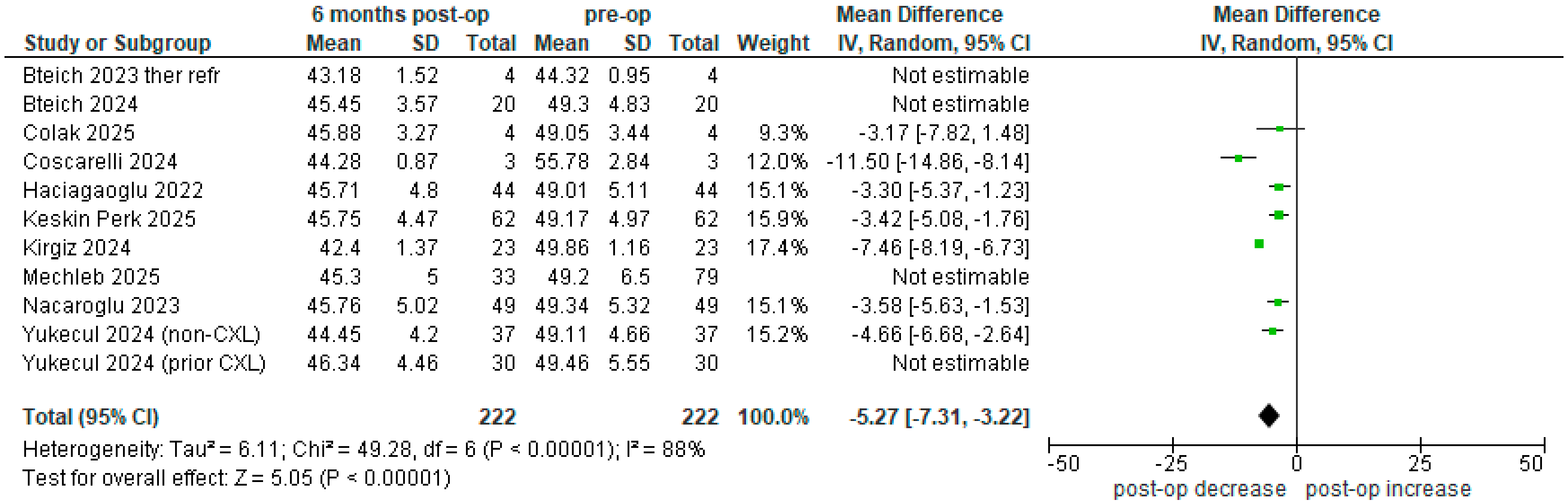

Figure S1.2.2.6B Difference in mean simulated keratometry across 3 studies [2, 12, 14] – FEMTOSECOND LASER

Study or Subgroup	6 months post-op Mean	SD	Total	pre-op Mean	SD	Total	Weight	Mean Difference IV, Random, 95% CI
Bteich 2023 ther refr	43.18	1.52	4	44.32	0.95	4	39.1%	-1.14 [-2.90, 0.62]
Bteich 2024	45.45	3.57	20	49.3	4.83	20	28.1%	-3.85 [-6.48, -1.22]
Colak 2025	45.88	3.27	4	49.05	3.44	4	0.0%	-3.17 [-7.82, 1.48]
Coscarelli 2024	44.28	0.87	3	55.78	2.84	3	0.0%	-11.50 [-14.86, -8.14]
Haciagaoglu 2022	45.71	4.8	44	49.01	5.11	44	0.0%	-3.30 [-5.37, -1.23]
Keskin Perk 2025	45.75	4.47	62	49.17	4.97	62	0.0%	-3.42 [-5.08, -1.76]
Kirgiz 2024	42.4	1.37	23	49.86	1.16	23	0.0%	-7.46 [-8.19, -6.73]
Mechleb 2025	45.3	5	33	49.2	6.5	79	32.8%	-3.90 [-6.13, -1.67]
Nacaroglu 2023	45.76	5.02	49	49.34	5.32	49	0.0%	-3.58 [-5.63, -1.53]
Yukecul 2024 (non-CXL)	44.45	4.2	37	49.11	4.66	37	0.0%	-4.66 [-6.68, -2.64]
Yukecul 2024 (prior CXL)	46.34	4.46	30	49.46	5.55	30	0.0%	-3.12 [-5.67, -0.57]
**Total (95% CI)**			**57**			**103**	**100.0%**	**-2.81 [-4.76, -0.85]**

Heterogeneity: Tau² = 1.74; Chi² = 4.82, df = 2 (P = 0.09); I² = 59%
Test for overall effect: Z = 2.81 (P = 0.005)

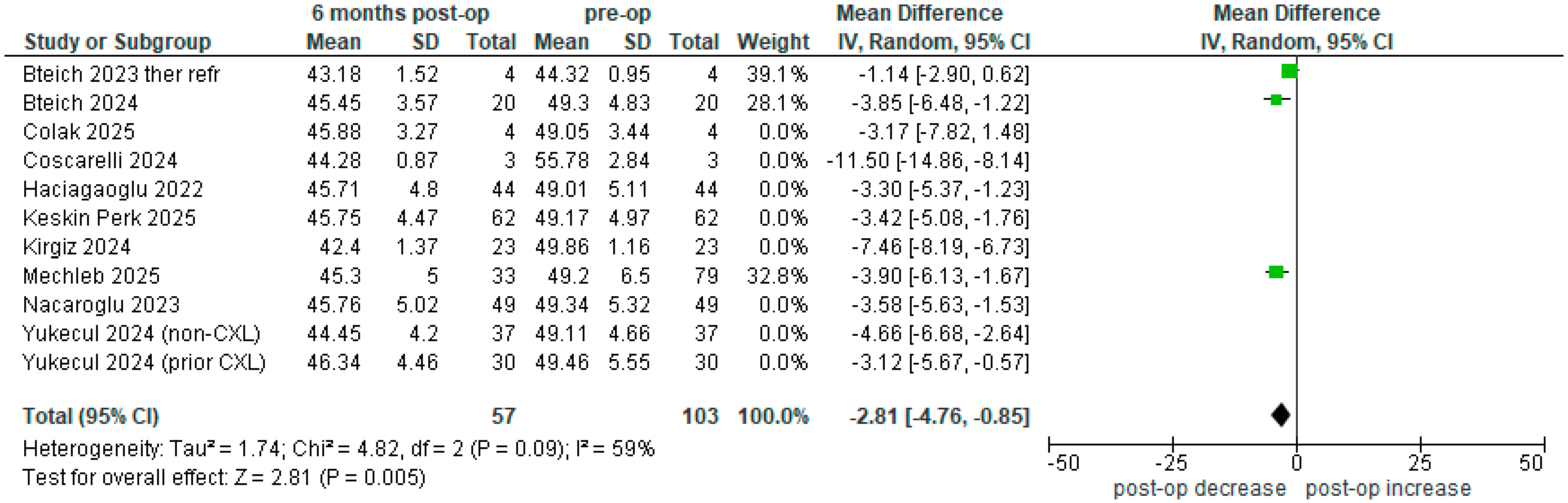

S1.2.2.7. Difference in total higher order aberrations

Figure S1.2.2.7A. Difference in total higher order aberrations across one study [10] – TREPHINE

Study or Subgroup	6 months post-op Mean	SD	Total	pre-op Mean	SD	Total	Weight	Mean Difference IV, Random, 95% CI
Bteich 2024	1.39	0.62	20	1.92	0.95	20		Not estimable
Hayashi 2025	4.24	1.61	5	5.59	1.55	5	100.0%	-1.35 [-3.31, 0.61]
Mechleb 2025	3.47	2.08	33	3.78	1.82	79	0.0%	-0.31 [-1.13, 0.51]
**Total (95% CI)**			**5**			**5**	**100.0%**	**-1.35 [-3.31, 0.61]**
Heterogeneity: Not applicable								
Test for overall effect: Z = 1.35 (P = 0.18)								

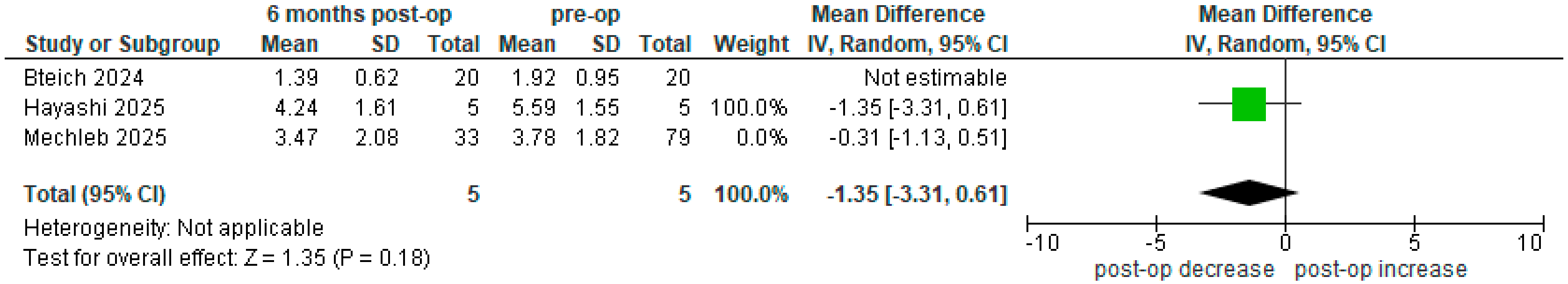

Figure S1.2.2.7B. Difference in total higher order aberrations across 2 studies [2, 14]– FEMTOSECOND LASER

	6 months post-op			pre-op				Mean Difference
**Study or Subgroup**	**Mean**	**SD**	**Total**	**Mean**	**SD**	**Total**	**Weight**	**IV, Random, 95% CI**
Bteich 2024	1.39	0.62	20	1.92	0.95	20	72.9%	-0.53 [-1.03, -0.03]
Hayashi 2025	4.24	1.61	5	5.59	1.55	5	0.0%	-1.35 [-3.31, 0.61]
Mechleb 2025	3.47	2.08	33	3.78	1.82	79	27.1%	-0.31 [-1.13, 0.51]
**Total (95% CI)**			**53**			**99**	**100.0%**	**-0.47 [-0.89, -0.05]**

Heterogeneity: Tau² = 0.00; Chi² = 0.20, df = 1 (P = 0.65); I² = 0%
Test for overall effect: Z = 2.17 (P = 0.03)

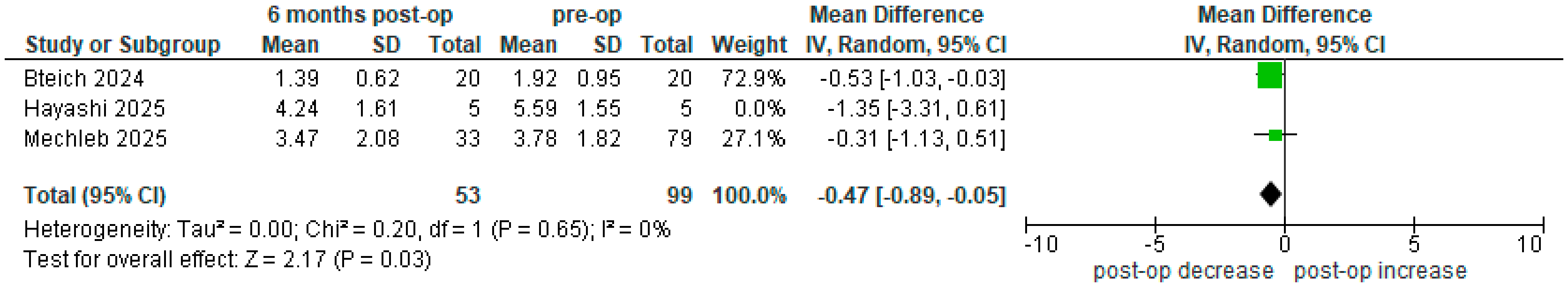

S1.2.2.8. Difference in spherical aberrations

Figure S1.2.2.8A. Difference in spherical aberrations across 1 study [5] - TREPHINE

	6 months post-op			pre-op				Mean Difference
**Study or Subgroup**	**Mean**	**SD**	**Total**	**Mean**	**SD**	**Total**	**Weight**	**IV, Random, 95% CI**
Bteich 2023 ther refr	-0.09	0.27	4	0.26	0.08	4	0.0%	-0.35 [-0.63, -0.07]
Bteich 2024	0.32	0.46	20	0.05	0.42	20	0.0%	0.27 [-0.00, 0.54]
Kirgiz 2024	0.65	0.1	23	0.38	0.07	23	100.0%	0.27 [0.22, 0.32]
**Total (95% CI)**			**23**			**23**	**100.0%**	**0.27 [0.22, 0.32]**

Heterogeneity: Not applicable
Test for overall effect: Z = 10.61 (P < 0.00001)

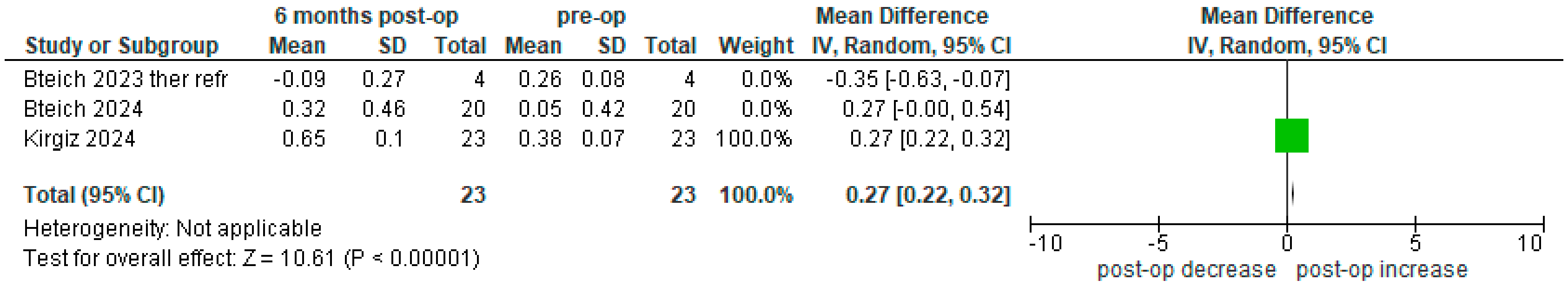

Figure S1.2.2.8B. Difference in spherical aberrations across 2 studies [2, 12]– FEMTOSECOND LASER

	6 months post-op			pre-op				Mean Difference
**Study or Subgroup**	**Mean**	**SD**	**Total**	**Mean**	**SD**	**Total**	**Weight**	**IV, Random, 95% CI**
Bteich 2023 ther refr	-0.09	0.27	4	0.26	0.08	4	49.9%	-0.35 [-0.63, -0.07]
Bteich 2024	0.32	0.46	20	0.05	0.42	20	50.1%	0.27 [-0.00, 0.54]
Kirgiz 2024	0.65	0.1	23	0.38	0.07	23	0.0%	0.27 [0.22, 0.32]
**Total (95% CI)**			**24**			**24**	**100.0%**	**-0.04 [-0.65, 0.57]**

Heterogeneity: Tau² = 0.17; Chi² = 9.80, df = 1 (P = 0.002); I² = 90%
Test for overall effect: Z = 0.13 (P = 0.90)

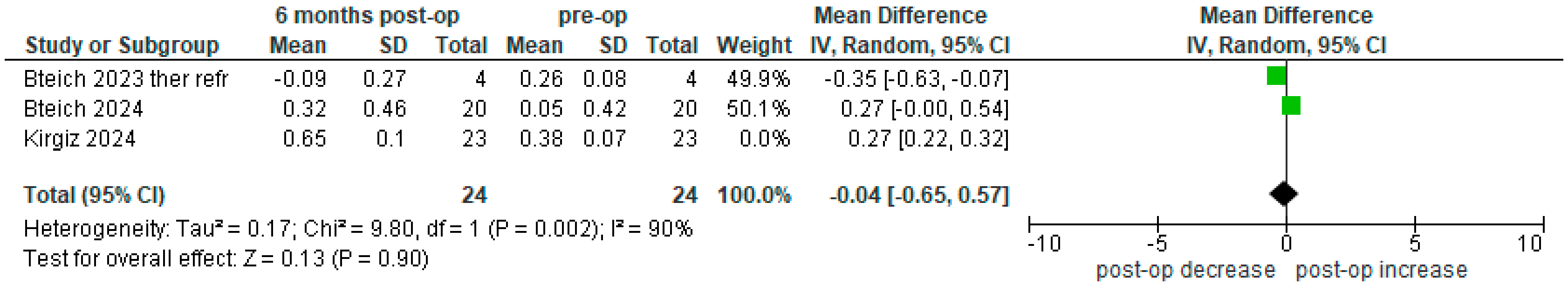

S1.2.2.9 Difference in vertical coma

S1.2.2.9A Difference in vertical coma across 0 studies – TREPHINE

Figure S1.2.2.9B Difference in vertical coma across 2 studies [2, 12] – FEMTOSECOND LASER

	6 months post-op			pre-op				Mean Difference
**Study or Subgroup**	**Mean**	**SD**	**Total**	**Mean**	**SD**	**Total**	**Weight**	**IV, Random, 95% CI**
Bteich 2023 ther refr	0.21	0.19	4	1.05	0.21	4	80.6%	-0.84 [-1.12, -0.56]
Bteich 2024	0.53	0.69	20	1.44	1.09	20	19.4%	-0.91 [-1.48, -0.34]
**Total (95% CI)**			**24**			**24**	**100.0%**	**-0.85 [-1.10, -0.60]**

Heterogeneity: $Tau^2 = 0.00$; $Chi^2 = 0.05$, df = 1 (P = 0.83); $I^2 = 0\%$
Test for overall effect: Z = 6.72 (P < 0.00001)

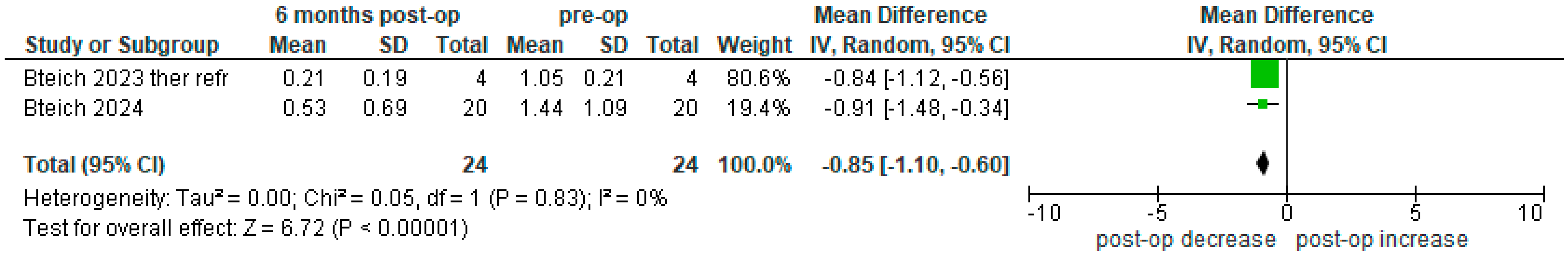

S1.2.2.10 Difference in horizontal coma across 0 studies

S1.2.2.10A Difference in horizontal coma across 0 studies – TREPHINE

S1.2.2.10B Difference in horizontal coma across 0 studies – FEMTOSECOND LASER

S1.2.2.11. Difference in trefoil

Figure S1.2.2.11A. Difference in trefoil across 1 study [5]– TREPHINE

	6 months post-op			pre-op				Mean Difference
**Study or Subgroup**	**Mean**	**SD**	**Total**	**Mean**	**SD**	**Total**	**Weight**	**IV, Random, 95% CI**
Bteich 2024	0.1	0.6	20	-0.27	0.4	20	0.0%	0.37 [0.05, 0.69]
Kirgiz 2024	1.45	0.11	23	0.66	0.13	23	100.0%	0.79 [0.72, 0.86]
**Total (95% CI)**			**23**			**23**	**100.0%**	**0.79 [0.72, 0.86]**

Heterogeneity: Not applicable
Test for overall effect: Z = 22.25 (P < 0.00001)

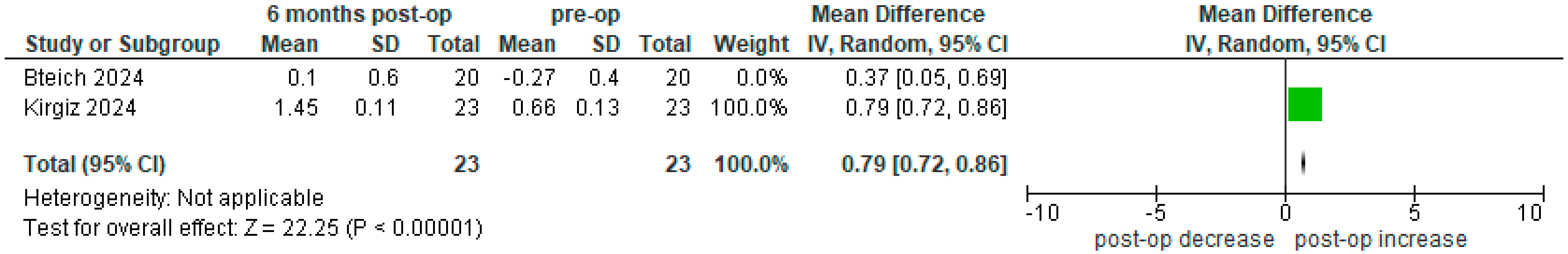

Figure S1.2.2.11B. Difference in trefoil across 1 study [2] – FEMTOSECOND LASER

	6 months post-op			pre-op				Mean Difference
**Study or Subgroup**	**Mean**	**SD**	**Total**	**Mean**	**SD**	**Total**	**Weight**	**IV, Random, 95% CI**
Bteich 2024	0.1	0.6	20	-0.27	0.4	20	100.0%	0.37 [0.05, 0.69]
Kirgiz 2024	1.45	0.11	23	0.66	0.13	23	0.0%	0.79 [0.72, 0.86]
**Total (95% CI)**			**20**			**20**	**100.0%**	**0.37 [0.05, 0.69]**

Heterogeneity: Not applicable
Test for overall effect: Z = 2.29 (P = 0.02)

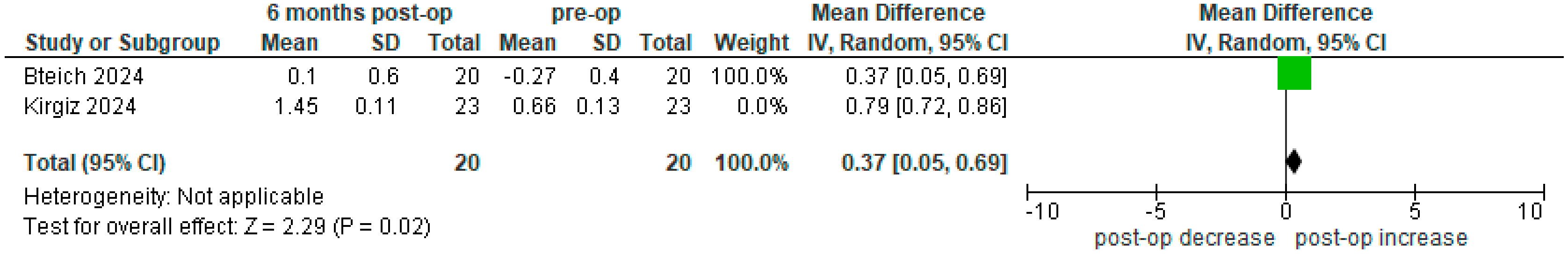

S1.2.2.12. Difference in total RMS

Figure S1.2.2.12A. Difference in total RMS in one study [5] - TREPHINE

	6 months post-op			pre-op				Mean Difference	Mean Difference
**Study or Subgroup**	**Mean**	**SD**	**Total**	**Mean**	**SD**	**Total**	**Weight**	**IV, Random, 95% CI**	**IV, Random, 95% CI**
Kirgiz 2024	2.44	0.15	23	2.35	0.3	23	100.0%	0.09 [-0.05, 0.23]	
Mechleb 2025	10.76	6.24	33	14.37	6.33	79	0.0%	-3.61 [-6.16, -1.06]	
**Total (95% CI)**			**23**			**23**	**100.0%**	**0.09 [-0.05, 0.23]**	

Heterogeneity: Not applicable
Test for overall effect: Z = 1.29 (P = 0.20)

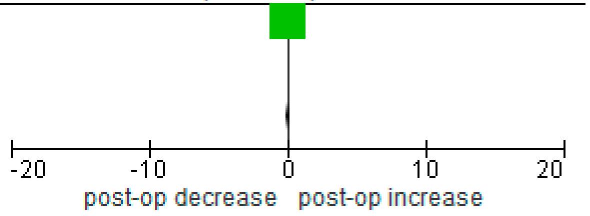

Figure S1.2.2.12B. Difference in total RMS in one study [14] – FEMTOSECOND LASER

	6 months post-op			pre-op				Mean Difference
**Study or Subgroup**	**Mean**	**SD**	**Total**	**Mean**	**SD**	**Total**	**Weight**	**IV, Random, 95% CI**
Kirgiz 2024	2.44	0.15	23	2.35	0.3	23	0.0%	0.09 [-0.05, 0.23]
Mechleb 2025	10.76	6.24	33	14.37	6.33	79	100.0%	-3.61 [-6.16, -1.06]
**Total (95% CI)**			**33**			**79**	**100.0%**	**-3.61 [-6.16, -1.06]**

Heterogeneity: Not applicable
Test for overall effect: Z = 2.78 (P = 0.005)

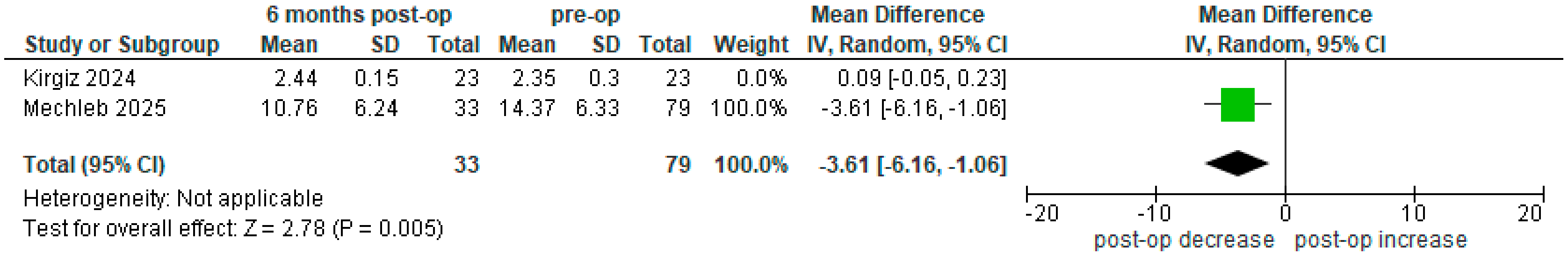

S1.2.3. 1 year postoperative versus preoperative

S1.2.3.1. Difference in uncorrected visual acuity

Figure S1.2.3.1. A. Difference in uncorrected visual acuity across 6 studies [4, 6, 15-18] – TREPHINE

	1 year post-op			pre-op				Mean Difference
**Study or Subgroup**	**Mean**	**SD**	**Total**	**Mean**	**SD**	**Total**	**Weight**	**IV, Random, 95% CI**
Asfar 2024	0.47	0.22	34	0.77	0.38	34	18.1%	-0.30 [-0.45, -0.15]
Bteich 2024	0.52	0.16	20	0.893	0.33	20	0.0%	-0.37 [-0.53, -0.21]
Jacob 2018	0.44	0.31	24	0.66	0.4	24	13.7%	-0.22 [-0.42, -0.02]
Jacob 2023	0.33	0.24	32	0.65	0.24	32	21.0%	-0.32 [-0.44, -0.20]
Keskin Perk 2025	0.44	0.35	50	0.96	0.5	62	17.2%	-0.52 [-0.68, -0.36]
Nacaroglu 2023	0.36	0.25	49	0.91	0.5	49	17.3%	-0.55 [-0.71, -0.39]
Yakut 2025	0.3	0.25	35	0.74	0.61	35	12.6%	-0.44 [-0.66, -0.22]
**Total (95% CI)**			**224**			**236**	**100.0%**	**-0.39 [-0.50, -0.29]**

Heterogeneity: $Tau^2 = 0.01$; $Chi^2 = 12.32$, df = 5 (P = 0.03); $I^2 = 59\%$
Test for overall effect: Z = 7.43 (P < 0.00001)

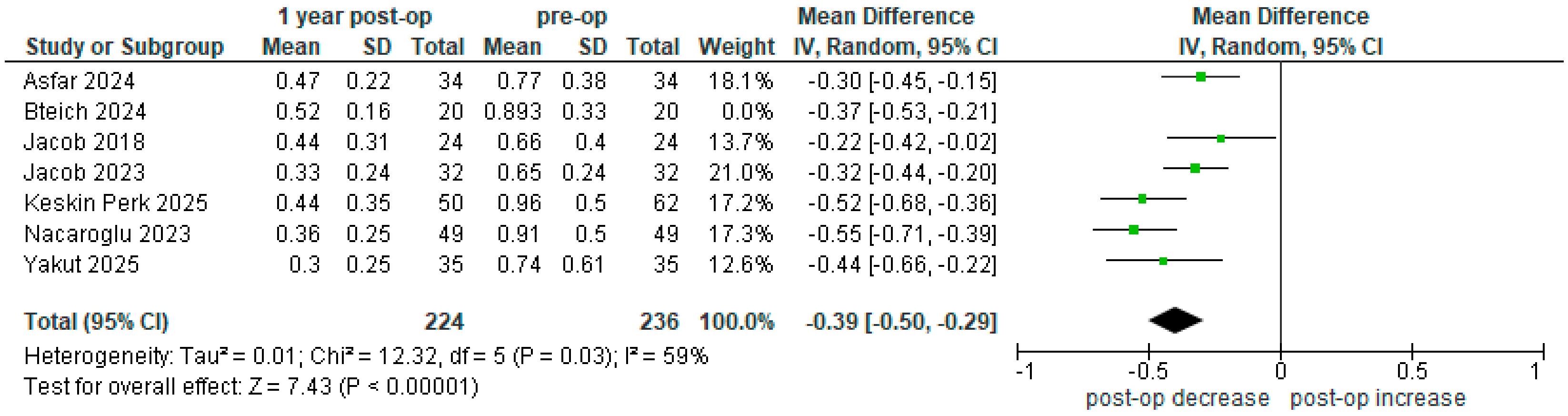

Figure S1.2.3.1B. Difference in uncorrected visual acuity in one study [2] – FEMTOSECOND LASER

	1 year post-op			pre-op				Mean Difference
**Study or Subgroup**	**Mean**	**SD**	**Total**	**Mean**	**SD**	**Total**	**Weight**	**IV, Random, 95% CI**
Asfar 2024	0.47	0.22	34	0.77	0.38	34	0.0%	-0.30 [-0.45, -0.15]
Bteich 2024	0.52	0.16	20	0.893	0.33	20	100.0%	-0.37 [-0.53, -0.21]
Jacob 2023	0.47	0.24	32	0.22	0.11	32	0.0%	0.25 [0.16, 0.34]
Keskin Perk 2025	0.44	0.35	50	0.96	0.5	62	0.0%	-0.52 [-0.68, -0.36]
Nacaroglu 2023	0.36	0.25	49	0.91	0.5	49	0.0%	-0.55 [-0.71, -0.39]
Yakut 2025	0.3	0.25	35	0.74	0.61	35	0.0%	-0.44 [-0.66, -0.22]
**Total (95% CI)**			**20**			**20**	**100.0%**	**-0.37 [-0.53, -0.21]**

Heterogeneity: Not applicable
Test for overall effect: Z = 4.55 (P < 0.00001)

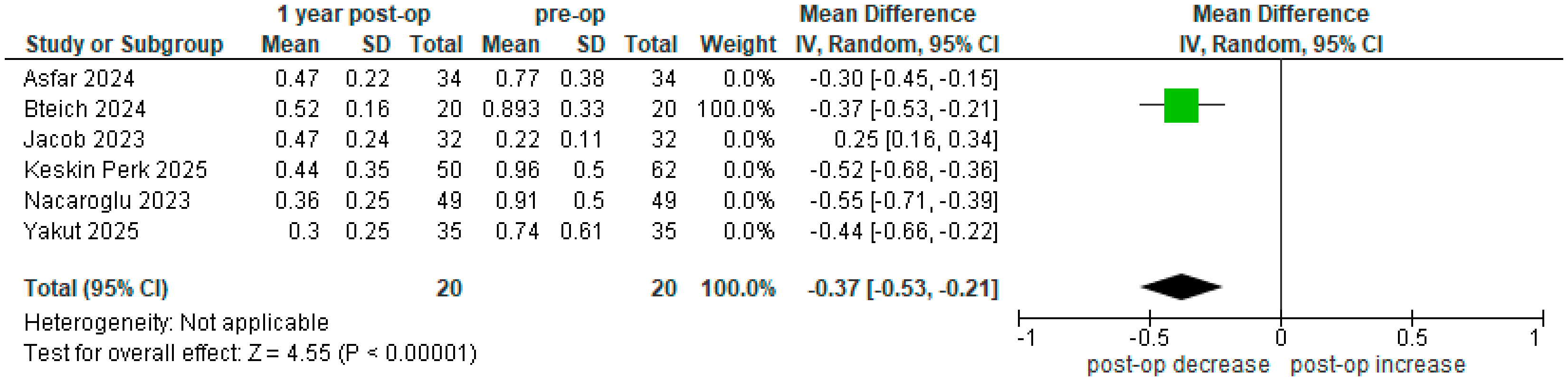

S1.2.3.2. Difference in best corrected visual acuity

Figure S1.2.3.2A. Difference in best corrected visual acuity across 6 studies [4, 6, 15-18]- TREPHINE

	1 year post-op			pre-op				Mean Difference
**Study or Subgroup**	**Mean**	**SD**	**Total**	**Mean**	**SD**	**Total**	**Weight**	**IV, Random, 95% CI**
Asfar 2024	0.16	0.18	34	0.52	0.23	34	16.6%	-0.36 [-0.46, -0.26]
Bteich 2024	0.15	0.18	20	0.57	0.18	20	0.0%	-0.42 [-0.53, -0.31]
Jacob 2018	0.1	0.11	24	0.23	0.16	24	16.9%	-0.13 [-0.21, -0.05]
Jacob 2023	0.05	0.06	32	0.12	0.11	32	17.3%	-0.07 [-0.11, -0.03]
Keskin Perk 2025	0.23	0.21	50	0.72	0.47	62	16.0%	-0.49 [-0.62, -0.36]
Nacaroglu 2023	0.36	0.15	49	0.87	0.2	49	17.0%	-0.51 [-0.58, -0.44]
Yakut 2025	0.15	0.15	35	0.54	0.32	35	16.2%	-0.39 [-0.51, -0.27]
**Total (95% CI)**			**224**			**236**	**100.0%**	**-0.32 [-0.50, -0.15]**

Heterogeneity: $Tau^2 = 0.05$; $Chi^2 = 149.08$, df = 5 ($P < 0.00001$); $I^2 = 97\%$
Test for overall effect: $Z = 3.58$ ($P = 0.0003$)

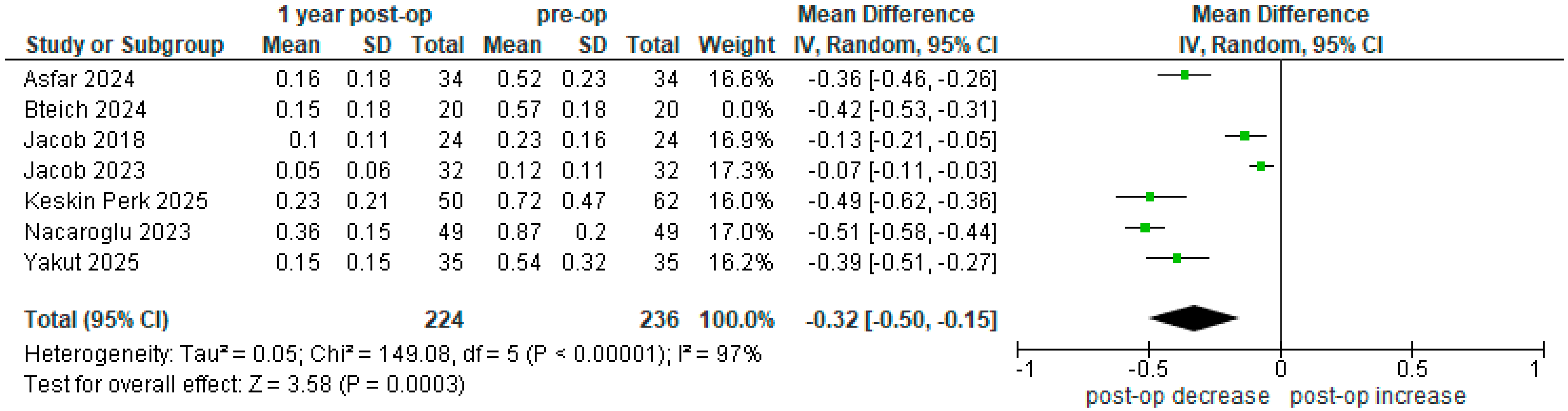

Figure S1.2.3.2B. Difference in best corrected visual acuity across 1 study [2] – FEMTOSECOND LASER

	1 year post-op			pre-op				Mean Difference
**Study or Subgroup**	**Mean**	**SD**	**Total**	**Mean**	**SD**	**Total**	**Weight**	**IV, Random, 95% CI**
Asfar 2024	0.16	0.18	34	0.52	0.23	34	0.0%	-0.36 [-0.46, -0.26]
Bteich 2024	0.15	0.18	20	0.57	0.18	20	100.0%	-0.42 [-0.53, -0.31]
Jacob 2023	0.89	0.17	32	0.76	0.19	32	0.0%	0.13 [0.04, 0.22]
Keskin Perk 2025	0.23	0.21	50	0.72	0.47	62	0.0%	-0.49 [-0.62, -0.36]
Nacaroglu 2023	0.36	0.15	49	0.87	0.2	49	0.0%	-0.51 [-0.58, -0.44]
Yakut 2025	0.15	0.15	35	0.54	0.32	35	0.0%	-0.39 [-0.51, -0.27]
**Total (95% CI)**			**20**			**20**	**100.0%**	**-0.42 [-0.53, -0.31]**

Heterogeneity: Not applicable
Test for overall effect: Z = 7.38 (P < 0.00001)

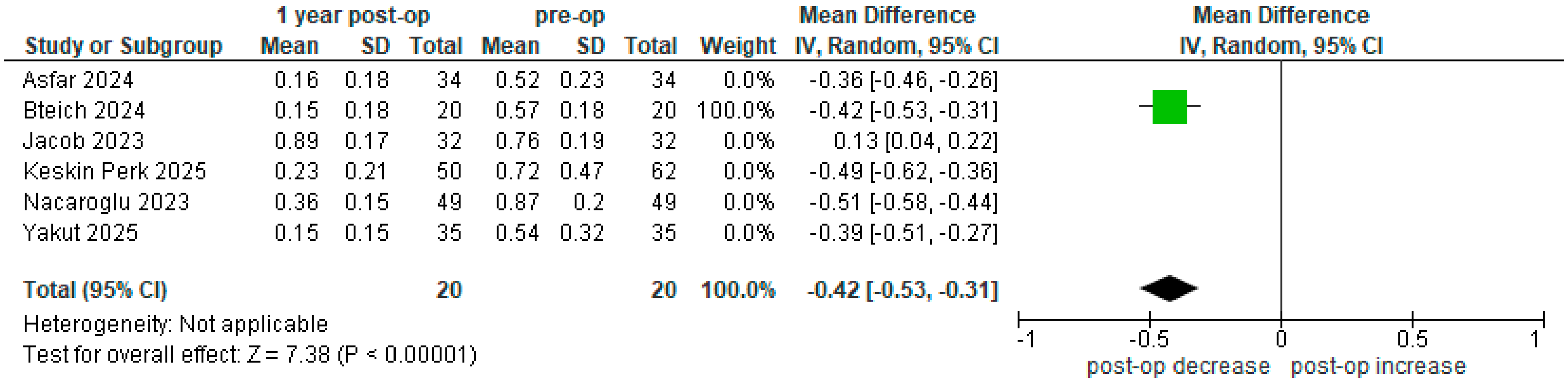

S1.2.3.3. Difference in pachymetry thinnest

Figure S1.2.3.3.A Difference in pachymetry thinnest across 2 studies [4, 17] – TREPHINE

	1 year post-op			pre-op				Mean Difference
**Study or Subgroup**	**Mean**	**SD**	**Total**	**Mean**	**SD**	**Total**	**Weight**	**IV, Random, 95% CI**
Bteich 2024	442	51.8	20	442.8	38.1	20		Not estimable
Jacob 2023	455.94	45.74	32	449.88	35.99	32	45.9%	6.06 [-14.11, 26.23]
Keskin Perk 2025	444.38	50.15	50	449.68	49.48	62	54.1%	-5.30 [-23.87, 13.27]
**Total (95% CI)**			**82**			**94**	**100.0%**	**-0.09 [-13.75, 13.57]**

Heterogeneity: Tau² = 0.00; Chi² = 0.66, df = 1 (P = 0.42); I² = 0%
Test for overall effect: Z = 0.01 (P = 0.99)

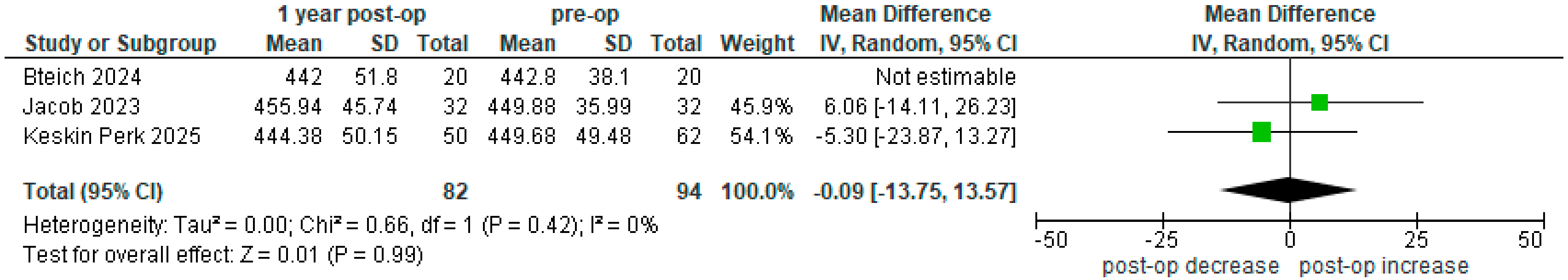

Figure S1.2.3.3.B Difference in pachymetry thinnest in one study [2] – FEMTOSECOND LASER

	1 year post-op			pre-op				Mean Difference
**Study or Subgroup**	**Mean**	**SD**	**Total**	**Mean**	**SD**	**Total**	**Weight**	**IV, Random, 95% CI**
Bteich 2024	442	51.8	20	442.8	38.1	20	100.0%	-0.80 [-28.98, 27.38]
Jacob 2023	455.94	45.74	32	449.88	35.99	32	0.0%	6.06 [-14.11, 26.23]
Keskin Perk 2025	444.38	50.15	50	449.68	49.48	62	0.0%	-5.30 [-23.87, 13.27]
**Total (95% CI)**			**20**			**20**	**100.0%**	**-0.80 [-28.98, 27.38]**

Heterogeneity: Not applicable
Test for overall effect: Z = 0.06 (P = 0.96)

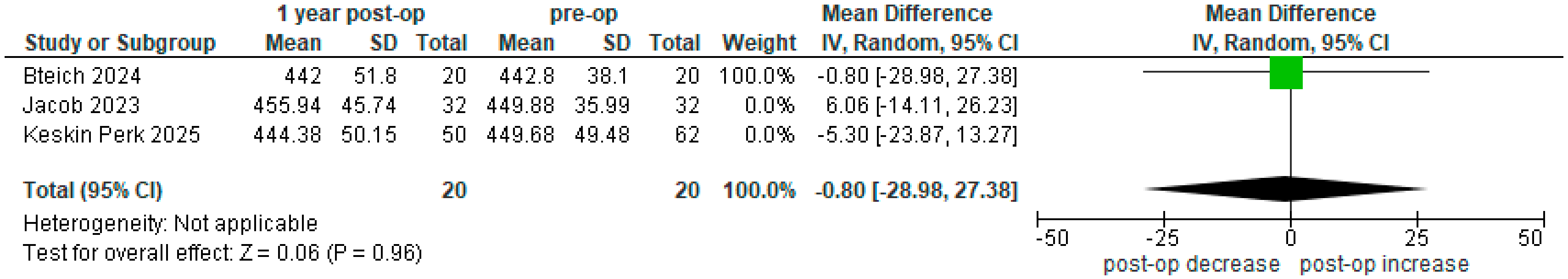

S1.2.3.4. Difference in pachymetry central point

Figure S1.2.3.4A. Difference in pachymetry central point across 2 studies [6, 18]– TREPHINE

	1 year post-op			pre-op				Mean Difference
**Study or Subgroup**	**Mean**	**SD**	**Total**	**Mean**	**SD**	**Total**	**Weight**	**IV, Random, 95% CI**
Nacaroglu 2023	447.31	56.35	49	463.62	54.88	49	52.3%	-16.31 [-38.33, 5.71]
Yakut 2025	452	50.75	35	453.91	47.65	35	47.7%	-1.91 [-24.97, 21.15]
**Total (95% CI)**			**84**			**84**	**100.0%**	**-9.44 [-25.37, 6.49]**

Heterogeneity: Tau² = 0.00; Chi² = 0.78, df = 1 (P = 0.38); I² = 0%
Test for overall effect: Z = 1.16 (P = 0.25)

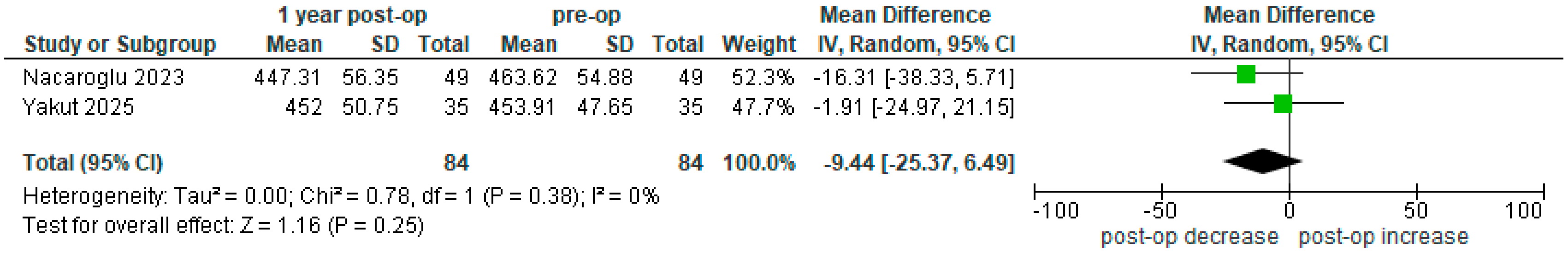

B.3.4B. Difference in pachymetry central point across 0 studies – FEMTOSECOND LASER

S1.2.3.5. Difference in maximum keratometry

Figure S1.2.3.5A. Difference in maximum keratometry across 5 studies [4, 6, 15, 17, 18]– TREPHINE

	1 year post-op			pre-op				Mean Difference
**Study or Subgroup**	**Mean**	**SD**	**Total**	**Mean**	**SD**	**Total**	**Weight**	**IV, Random, 95% CI**
Asfar 2024	50.69	6.38	34	55.85	7.53	34	13.8%	-5.16 [-8.48, -1.84]
Bteich 2024	52.85	5.44	20	57.21	7.53	20	0.0%	-4.36 [-8.43, -0.29]
Jacob 2023	54.95	4.71	32	58.9	5.54	32	23.9%	-3.95 [-6.47, -1.43]
Keskin Perk 2025	55.17	5.87	50	58.51	7.55	62	24.5%	-3.34 [-5.83, -0.85]
Nacaroglu 2023	55.63	6.14	49	59.2	7.66	49	20.1%	-3.57 [-6.32, -0.82]
Yakut 2025	54.29	5.61	35	57	6.81	35	17.7%	-2.71 [-5.63, 0.21]
**Total (95% CI)**			**200**			**212**	**100.0%**	**-3.67 [-4.90, -2.44]**

Heterogeneity: Tau² = 0.00; Chi² = 1.31, df = 4 (P = 0.86); I² = 0%
Test for overall effect: Z = 5.84 (P < 0.00001)

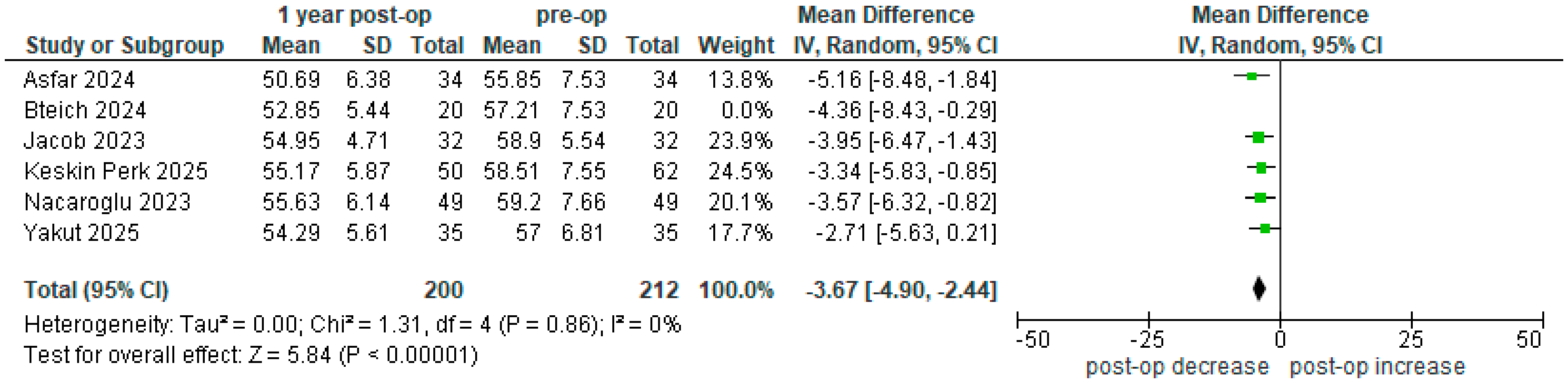

Figure S1.2.3.5B. Difference in maximum keratometry in 1 study [2] – FEMTOSECOND LASER

	1 year post-op			pre-op				Mean Difference
**Study or Subgroup**	**Mean**	**SD**	**Total**	**Mean**	**SD**	**Total**	**Weight**	**IV, Random, 95% CI**
Asfar 2024	50.69	6.38	34	55.85	7.53	34	0.0%	-5.16 [-8.48, -1.84]
Bteich 2024	52.85	5.44	20	57.21	7.53	20	100.0%	-4.36 [-8.43, -0.29]
Jacob 2023	54.95	4.71	32	58.9	5.54	32	0.0%	-3.95 [-6.47, -1.43]
Keskin Perk 2025	55.17	5.87	50	58.51	7.55	62	0.0%	-3.34 [-5.83, -0.85]
Nacaroglu 2023	55.63	6.14	49	59.2	7.66	49	0.0%	-3.57 [-6.32, -0.82]
Yakut 2025	54.29	5.61	35	57	6.81	35	0.0%	-2.71 [-5.63, 0.21]
**Total (95% CI)**			**20**			**20**	**100.0%**	**-4.36 [-8.43, -0.29]**

Heterogeneity: Not applicable
Test for overall effect: Z = 2.10 (P = 0.04)

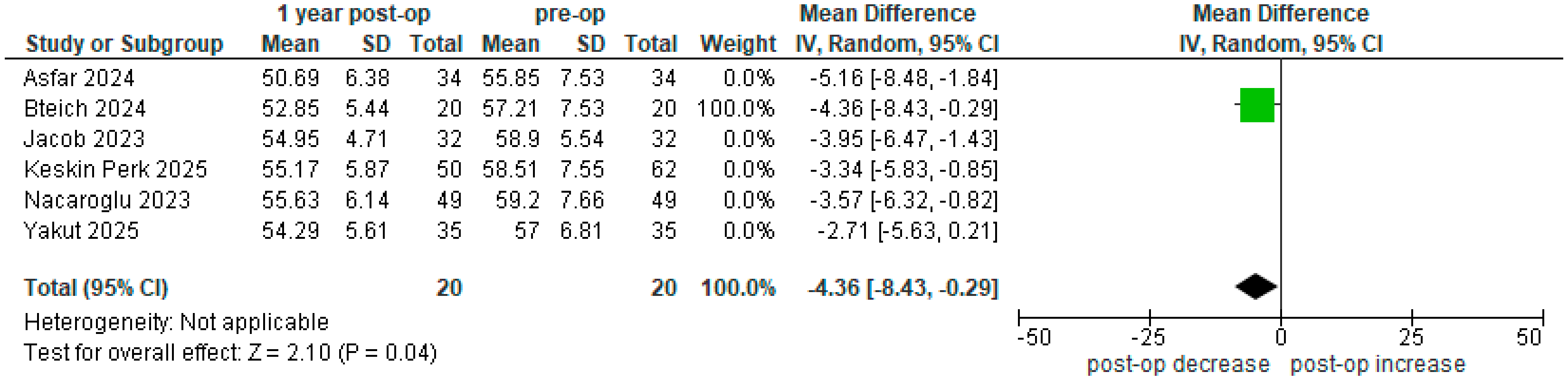

S1.2.3.6. Difference in mean simulated keratometry

Figure S1.2.3.6A. Difference in mean simulated keratometry across 5 studies [4, 6, 15, 17, 18]– TREPHINE

	1 year post-op			pre-op				Mean Difference
Study or Subgroup	Mean	SD	Total	Mean	SD	Total	Weight	IV, Random, 95% CI
Asfar 2024	44.25	3.29	34	47.52	3.82	34	21.7%	-3.27 [-4.96, -1.58]
Bteich 2024	45.23	2.83	20	49.3	4.83	20		Not estimable
Jacob 2023	45.63	2.91	32	49.07	3.06	32	29.2%	-3.44 [-4.90, -1.98]
Keskin Perk 2025	45.63	4.44	50	49.17	4.97	62	20.5%	-3.54 [-5.28, -1.80]
Nacaroglu 2023	45.39	4.92	49	49.34	5.32	49	15.2%	-3.95 [-5.98, -1.92]
Yakut 2025	44.5	4.42	35	48.26	4.78	35	13.4%	-3.76 [-5.92, -1.60]
**Total (95% CI)**			**200**			**212**	**100.0%**	**-3.54 [-4.33, -2.75]**

Heterogeneity: $Tau^2 = 0.00$; $Chi^2 = 0.31$, $df = 4$ ($P = 0.99$); $I^2 = 0\%$
Test for overall effect: $Z = 8.79$ ($P < 0.00001$)

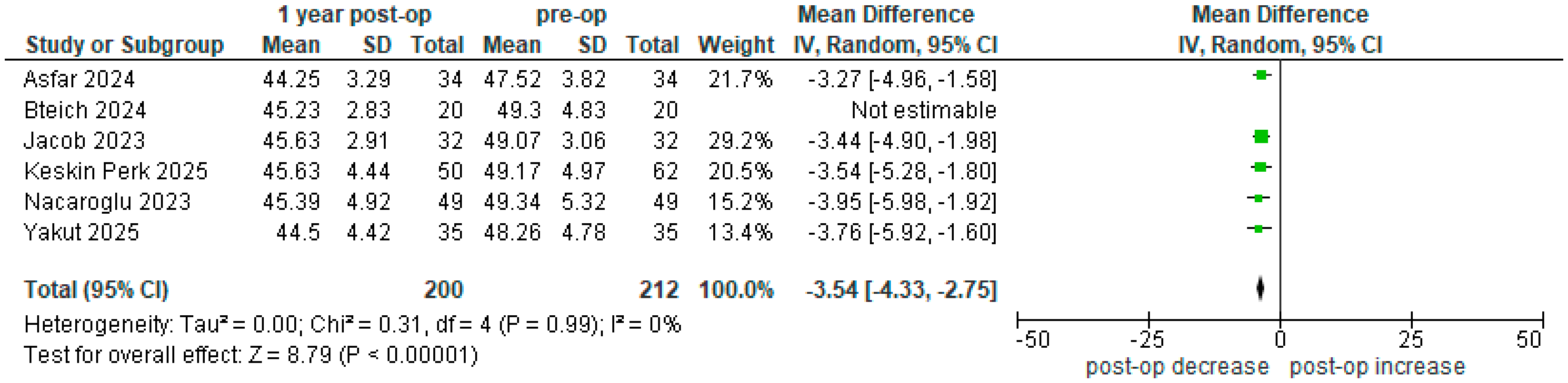

Figure S1.2.3.6B. Difference in mean simulated keratometry in 1 study [2] – FEMTOSECOND LASER

	1 year post-op			pre-op				Mean Difference
Study or Subgroup	Mean	SD	Total	Mean	SD	Total	Weight	IV, Random, 95% CI
Asfar 2024	44.25	3.29	34	47.52	3.82	34	0.0%	-3.27 [-4.96, -1.58]
Bteich 2024	45.23	2.83	20	49.3	4.83	20	100.0%	-4.07 [-6.52, -1.62]
Jacob 2023	45.63	2.91	32	49.07	3.06	32	0.0%	-3.44 [-4.90, -1.98]
Keskin Perk 2025	45.63	4.44	50	49.17	4.97	62	0.0%	-3.54 [-5.28, -1.80]
Nacaroglu 2023	45.39	4.92	49	49.34	5.32	49	0.0%	-3.95 [-5.98, -1.92]
Yakut 2025	44.5	4.42	35	48.26	4.78	35	0.0%	-3.76 [-5.92, -1.60]
**Total (95% CI)**			**20**			**20**	**100.0%**	**-4.07 [-6.52, -1.62]**

Heterogeneity: Not applicable
Test for overall effect: Z = 3.25 (P = 0.001)

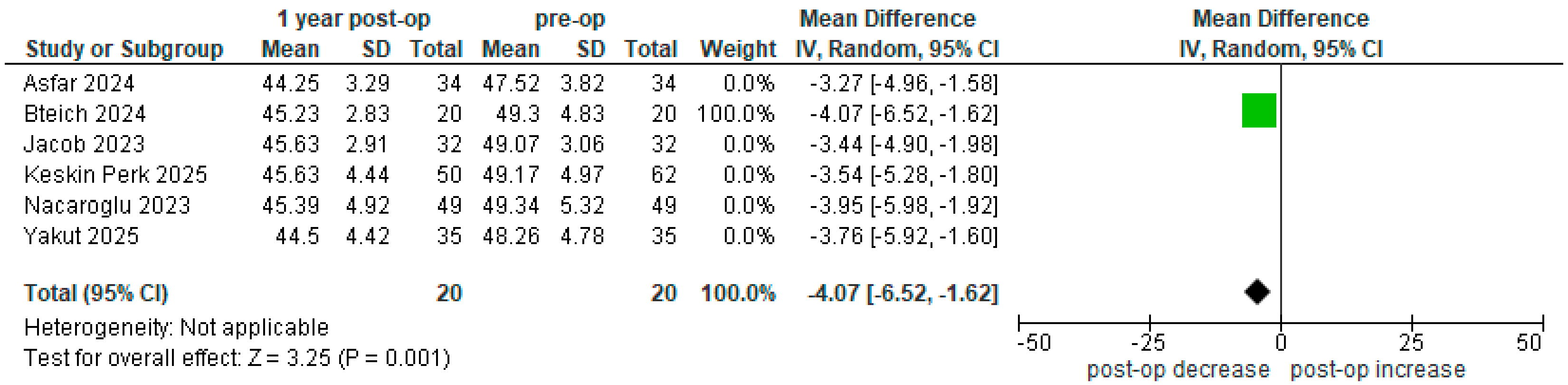

S1.2.3.7 Difference in total higher order aberrations

Figure S2.3.7A Difference in total higher order aberrations in 1 study [15]– TREPHINE

	1 year post-op			pre-op				Mean Difference
**Study or Subgroup**	**Mean**	**SD**	**Total**	**Mean**	**SD**	**Total**	**Weight**	**IV, Random, 95% CI**
Asfar 2024	1.54	0.93	34	2.07	1.05	34	100.0%	-0.53 [-1.00, -0.06]
Bteich 2024	1.36	0.65	20	1.92	0.95	20	0.0%	-0.56 [-1.06, -0.06]
**Total (95% CI)**			**34**			**34**	**100.0%**	**-0.53 [-1.00, -0.06]**
Heterogeneity: Not applicable								
Test for overall effect: Z = 2.20 (P = 0.03)								

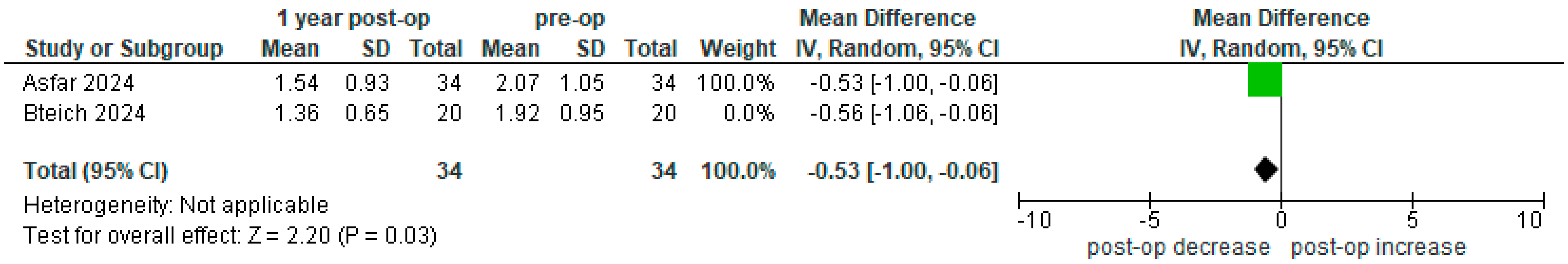

Figure S1.2.3.7B Difference in total higher order aberrations across 1 study [2] – FEMTOSECOND LASER

	1 year post-op			pre-op				Mean Difference
**Study or Subgroup**	**Mean**	**SD**	**Total**	**Mean**	**SD**	**Total**	**Weight**	**IV, Random, 95% CI**
Asfar 2024	1.54	0.93	34	2.07	1.05	34	0.0%	-0.53 [-1.00, -0.06]
Bteich 2024	1.36	0.65	20	1.92	0.95	20	100.0%	-0.56 [-1.06, -0.06]
**Total (95% CI)**			**20**			**20**	**100.0%**	**-0.56 [-1.06, -0.06]**

Heterogeneity: Not applicable
Test for overall effect: Z = 2.18 (P = 0.03)

Mean Difference
IV, Random, 95% CI

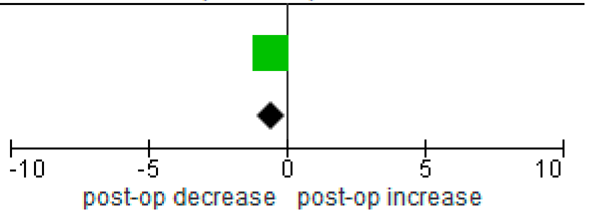

S1.2.3.8 Difference in spherical aberration

Figure S1.2.3.8A Difference in spherical aberration in 1 study [15]– TREPHINE

	1 year post-op			pre-op				Mean Difference
**Study or Subgroup**	**Mean**	**SD**	**Total**	**Mean**	**SD**	**Total**	**Weight**	**IV, Random, 95% CI**
Asfar 2024	0.18	0.5	34	0.07	0.42	34	100.0%	0.11 [-0.11, 0.33]
Bteich 2024	0.15	0.46	20	0.05	0.42	20	0.0%	0.10 [-0.17, 0.37]
**Total (95% CI)**			**34**			**34**	**100.0%**	**0.11 [-0.11, 0.33]**

Heterogeneity: Not applicable
Test for overall effect: Z = 0.98 (P = 0.33)

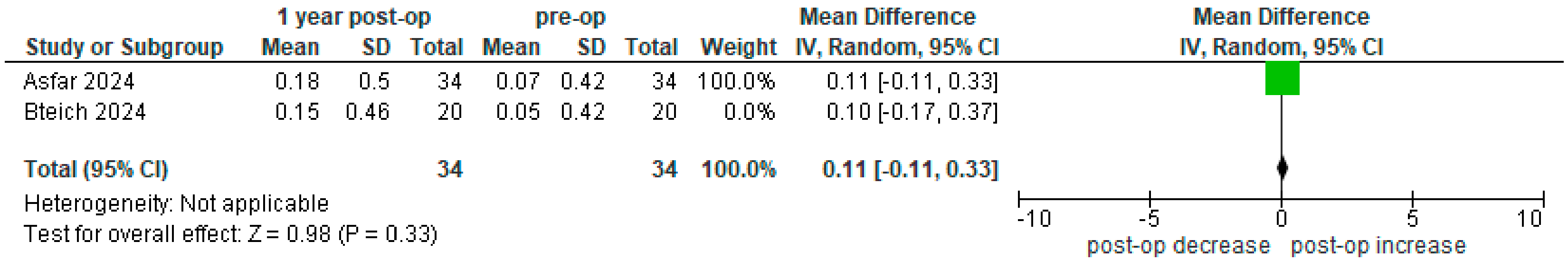

Figure S1.2.3.8B Difference in spherical aberration across 1 study[2] – FEMTOSECOND LASER

	1 year post-op			pre-op				Mean Difference
**Study or Subgroup**	**Mean**	**SD**	**Total**	**Mean**	**SD**	**Total**	**Weight**	**IV, Random, 95% CI**
Asfar 2024	0.18	0.5	34	0.07	0.42	34	0.0%	0.11 [-0.11, 0.33]
Bteich 2024	0.15	0.46	20	0.05	0.42	20	100.0%	0.10 [-0.17, 0.37]
**Total (95% CI)**			**20**			**20**	**100.0%**	**0.10 [-0.17, 0.37]**

Heterogeneity: Not applicable
Test for overall effect: Z = 0.72 (P = 0.47)

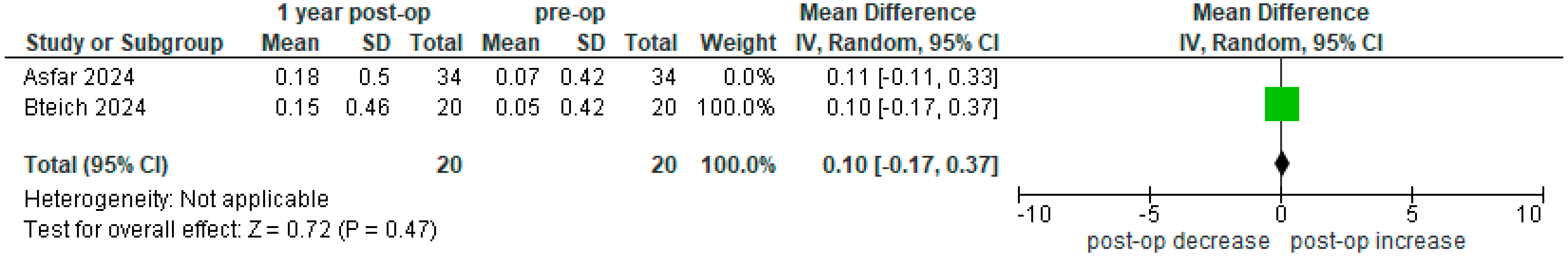

S1.2.3.9 Difference in vertical coma

Figure S1.2.3.9A Difference in vertical coma across 2 studies[15, 17] – TREPHINE

	1 year post-op			pre-op				Mean Difference
**Study or Subgroup**	**Mean**	**SD**	**Total**	**Mean**	**SD**	**Total**	**Weight**	**IV, Random, 95% CI**
Asfar 2024	0.38	0.65	34	1.49	1.02	34	50.5%	-1.11 [-1.52, -0.70]
Bteich 2024	0.35	0.97	20	1.44	1.09	20		Not estimable
Jacob 2023	-1.4	1.21	32	-2.09	0.96	32	49.5%	0.69 [0.15, 1.23]
**Total (95% CI)**			**66**			**66**	**100.0%**	**-0.22 [-1.98, 1.55]**

Heterogeneity: Tau² = 1.56; Chi² = 27.56, df = 1 (P < 0.00001); I² = 96%
Test for overall effect: Z = 0.24 (P = 0.81)

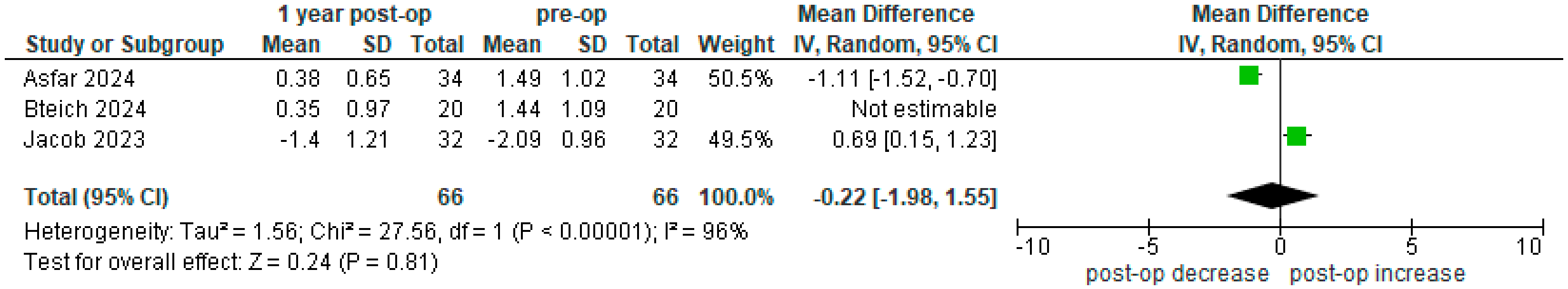

Figure S1.2.3.9B Difference in vertical coma across 1 study [2] – FEMTOSECOND LASER

	1 year post-op			pre-op				Mean Difference
Study or Subgroup	Mean	SD	Total	Mean	SD	Total	Weight	IV, Random, 95% CI
Asfar 2024	0.38	0.65	34	1.49	1.02	34	0.0%	-1.11 [-1.52, -0.70]
Bteich 2024	0.35	0.97	20	1.44	1.09	20	100.0%	-1.09 [-1.73, -0.45]
Jacob 2023	-1.4	1.21	32	-2.09	0.96	32	0.0%	0.69 [0.15, 1.23]
**Total (95% CI)**			**20**			**20**	**100.0%**	**-1.09 [-1.73, -0.45]**

Heterogeneity: Not applicable
Test for overall effect: Z = 3.34 (P = 0.0008)

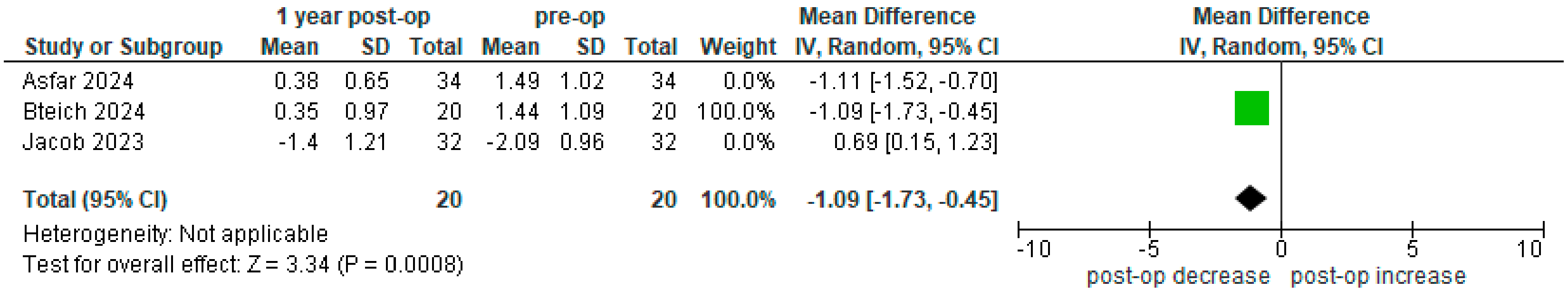

S1.2.3.10 Difference in horizontal coma across 0 studies

S1.2.3.10A Difference in horizontal coma across 0 studies – TREPHINE

S1.2.3.10B Difference in horizontal coma across 0 studies – FEMTOSECOND LASER

S1.2.3.11 Difference in trefoil

Figure S1.2.3.11A Difference in trefoil in one study [15] – TREPHINE

	1 year post-op			pre-op				Mean Difference
**Study or Subgroup**	**Mean**	**SD**	**Total**	**Mean**	**SD**	**Total**	**Weight**	**IV, Random, 95% CI**
Asfar 2024	-0.17	0.63	34	-0.37	0.59	34	100.0%	0.20 [-0.09, 0.49]
Bteich 2024	0.007	0.59	20	-0.27	0.4	20	0.0%	0.28 [-0.04, 0.59]
**Total (95% CI)**			**34**			**34**	**100.0%**	**0.20 [-0.09, 0.49]**

Heterogeneity: Not applicable
Test for overall effect: Z = 1.35 (P = 0.18)

Mean Difference
IV, Random, 95% CI

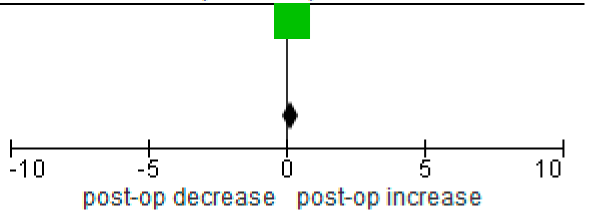

Figure S1.2.3.11B Difference in trefoil in one study [2] – FEMTOSECOND LASER

	1 year post-op			pre-op				Mean Difference
**Study or Subgroup**	**Mean**	**SD**	**Total**	**Mean**	**SD**	**Total**	**Weight**	**IV, Random, 95% CI**
Asfar 2024	-0.17	0.63	34	-0.37	0.59	34	0.0%	0.20 [-0.09, 0.49]
Bteich 2024	0.007	0.59	20	-0.27	0.4	20	100.0%	0.28 [-0.04, 0.59]
**Total (95% CI)**			**20**			**20**	**100.0%**	**0.28 [-0.04, 0.59]**

Heterogeneity: Not applicable
Test for overall effect: Z = 1.74 (P = 0.08)

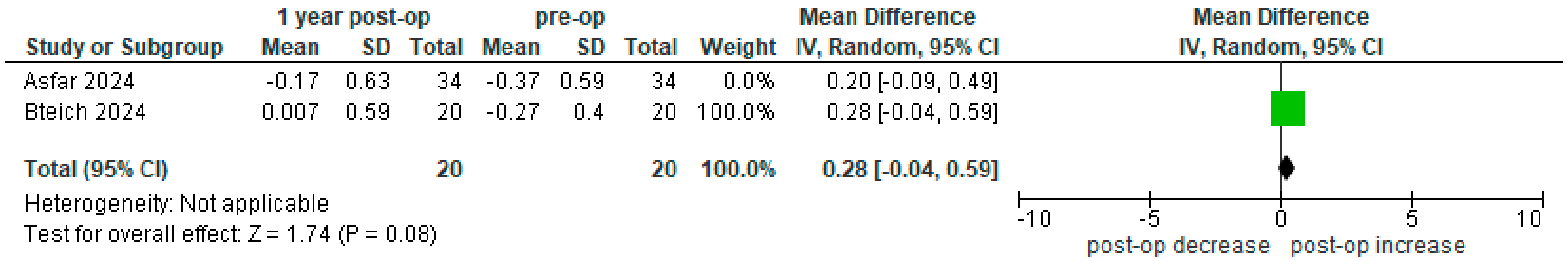

Figure S1.2.3.12A Difference in total RMS in one study [17] – TREPHINE

	1 year post-op			pre-op				Mean Difference
**Study or Subgroup**	**Mean**	**SD**	**Total**	**Mean**	**SD**	**Total**	**Weight**	**IV, Random, 95% CI**
Jacob 2023	2.59	0.8	32	2.85	0.78	32	100.0%	-0.26 [-0.65, 0.13]
**Total (95% CI)**			**32**			**32**	**100.0%**	**-0.26 [-0.65, 0.13]**

Heterogeneity: Not applicable
Test for overall effect: Z = 1.32 (P = 0.19)

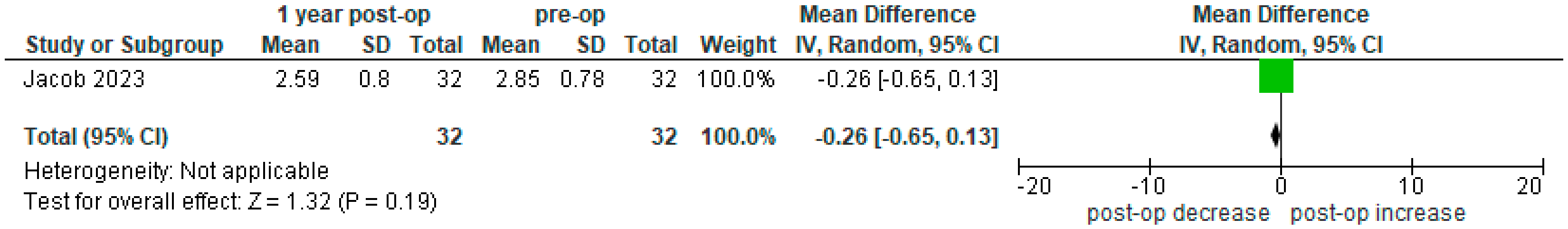

Figure S1.2.3.12B Difference in total RMS across 0 study – FEMTOSECOND LASER
No studies

SECTION S1.3 – SUBGROUP ANALYSIS CAIRS HYDRATION – HYDRATED VS DEHYDRATED

S1.3.1. 1 month postoperative versus preoperative

S1.3.1.1 Difference in uncorrected visual acuity

S1.3.1.1A. Difference in uncorrected visual acuity across 0 study – HYDRATED

Figure S1.3.1.1B. Difference in uncorrected visual acuity across 3 studies [1, 2, 5] – DEHYDRATED

	1 month post-op			pre-op				Mean Difference
Study or Subgroup	Mean	SD	Total	Mean	SD	Total	Weight	IV, Random, 95% CI
Bteich 2023	0.64	0.23	52	0.97	0.47	52	33.3%	-0.33 [-0.47, -0.19]
Bteich 2024	0.66	0.33	20	0.893	0.33	20	30.8%	-0.23 [-0.44, -0.03]
Haciagaoglu 2022	0.34	0.26	44	0.72	0.63	44	0.0%	-0.38 [-0.58, -0.18]
Keskin Perk 2025	0.4	0.34	62	0.96	0.5	62	0.0%	-0.56 [-0.71, -0.41]
Kirgiz 2024	0.432	0.03	23	1.096	0.05	23	36.0%	-0.66 [-0.69, -0.64]
Nacaroglu 2023	0.39	0.28	49	0.91	0.5	49	0.0%	-0.52 [-0.68, -0.36]
Yukecul 2024 (non-CXL)	0.26	0.22	37	0.77	0.76	37	0.0%	-0.51 [-0.76, -0.26]
Yukecul 2024 (prior CXL)	0.38	0.29	30	0.7	0.55	30	0.0%	-0.32 [-0.54, -0.10]
**Total (95% CI)**			**95**			**95**	**100.0%**	**-0.42 [-0.72, -0.12]**

Heterogeneity: $Tau^2 = 0.06; Chi^2 = 36.74, df = 2 (P < 0.00001); I^2 = 95\%$
Test for overall effect: Z = 2.78 (P = 0.006)

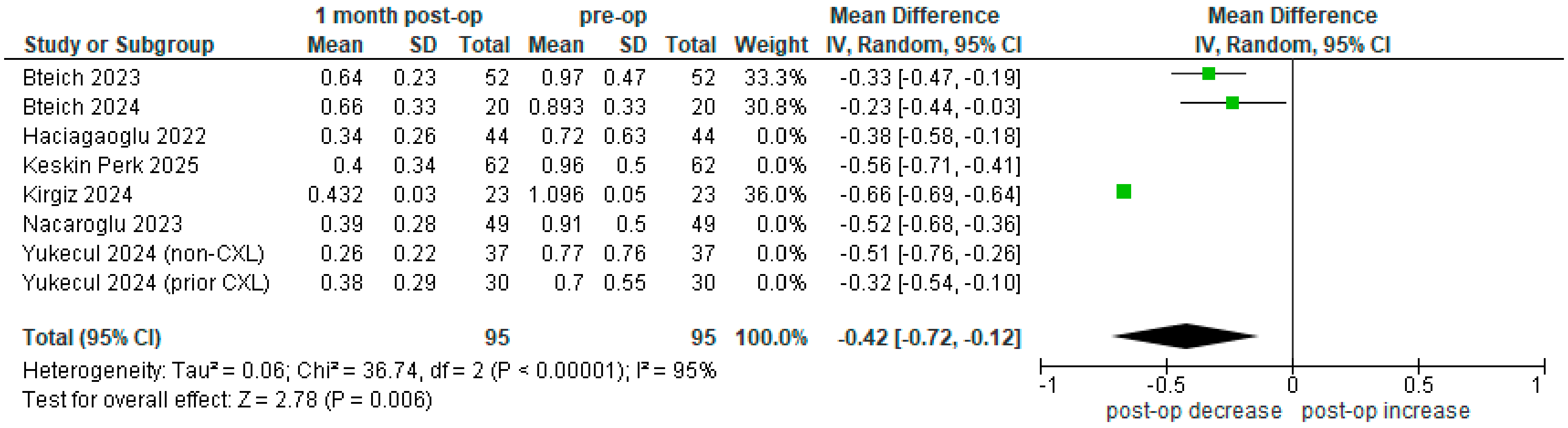

S1.3.1.2 Difference in best corrected visual acuity

S1.3.1.2A. Difference in best corrected visual acuity across 0 study – HYDRATED

Figure S1.3.1.2B. Difference in best corrected visual acuity across 4 studies [1, 2, 5, 8] – DEHYDRATED

	1 month post-op			pre-op				Mean Difference
**Study or Subgroup**	**Mean**	**SD**	**Total**	**Mean**	**SD**	**Total**	**Weight**	**IV, Random, 95% CI**
Bteich 2023	0.31	0.21	52	0.56	0.19	52	33.5%	-0.25 [-0.33, -0.17]
Bteich 2024	0.32	0.2	20	0.57	0.18	20	20.8%	-0.25 [-0.37, -0.13]
Haciagaoglu 2022	0.24	0.19	44	0.54	0.37	44	0.0%	-0.30 [-0.42, -0.18]
Keskin Perk 2025	0.28	0.22	62	0.72	0.47	62	0.0%	-0.44 [-0.57, -0.31]
Kirgiz 2024	0.237	0.06	23	0.602	0.3	23	19.2%	-0.36 [-0.49, -0.24]
Mechleb 2024 (Kmax inf 75D)	0.03	0.02	6	0.36	0.12	6	26.3%	-0.33 [-0.43, -0.23]
Mechleb 2024 (Kmax sup 75D)	1.03	0.88	4	2.45	1.1	4	0.2%	-1.42 [-2.80, -0.04]
Nacaroglu 2023	0.27	0.06	49	0.87	0.2	49	0.0%	-0.60 [-0.66, -0.54]
Yukecul 2024 (non-CXL)	0.19	0.15	37	0.51	0.29	37	0.0%	-0.32 [-0.43, -0.21]
Yukecul 2024 (prior CXL)	0.27	0.2	30	0.55	0.39	30	0.0%	-0.28 [-0.44, -0.12]
**Total (95% CI)**			**105**			**105**	**100.0%**	**-0.30 [-0.36, -0.23]**

Heterogeneity: Tau² = 0.00; Chi² = 6.08, df = 4 (P = 0.19); I² = 34%
Test for overall effect: Z = 8.77 (P < 0.00001)

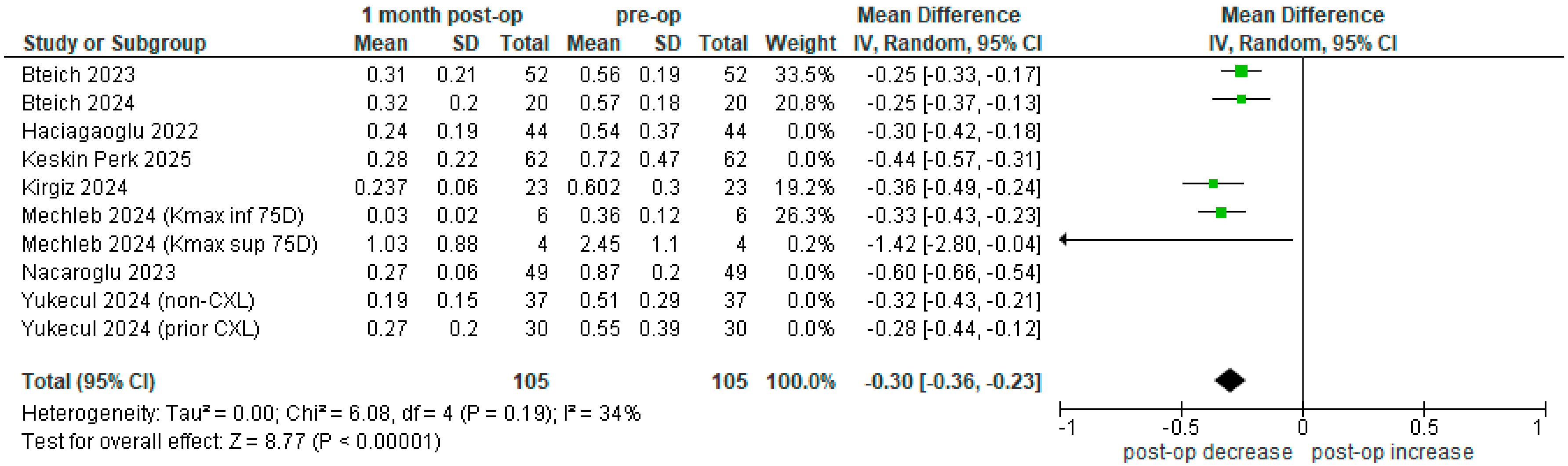

S1.3.1.3 Difference in pachymetry thinnest

Figure S1.3.1.3A. Difference in pachymetry thinnest across 0 study – HYDRATED

Figure S1.3.1.3B. Difference in pachymetry thinnest across 3 studies [1, 2, 5] – DEHYDRATED

	1 month post-op			pre-op				Mean Difference
**Study or Subgroup**	**Mean**	**SD**	**Total**	**Mean**	**SD**	**Total**	**Weight**	**IV, Random, 95% CI**
Bteich 2023	429.7	55.5	52	422.5	55.2	52	10.8%	7.20 [-14.08, 28.48]
Bteich 2024	443.8	41.2	20	442.8	38.1	20	8.2%	1.00 [-23.59, 25.59]
Haciagaoglu 2022	449.2	57.41	44	453.73	45.87	44	0.0%	-4.53 [-26.24, 17.18]
Keskin Perk 2025	446.32	40.26	62	449.68	49.48	62	0.0%	-3.36 [-19.24, 12.52]
Kirgiz 2024	431.33	11.78	23	439.33	10.43	23	81.0%	-8.00 [-14.43, -1.57]
Yukecul 2024 (non-CXL)	452	36	37	454	45	37	0.0%	-2.00 [-20.57, 16.57]
Yukecul 2024 (prior CXL)	449	42	30	437	38	30	0.0%	12.00 [-8.27, 32.27]
**Total (95% CI)**			**95**			**95**	**100.0%**	**-5.62 [-12.79, 1.56]**

Heterogeneity: Tau² = 5.78; Chi² = 2.15, df = 2 (P = 0.34); I² = 7%

Test for overall effect: Z = 1.53 (P = 0.12)

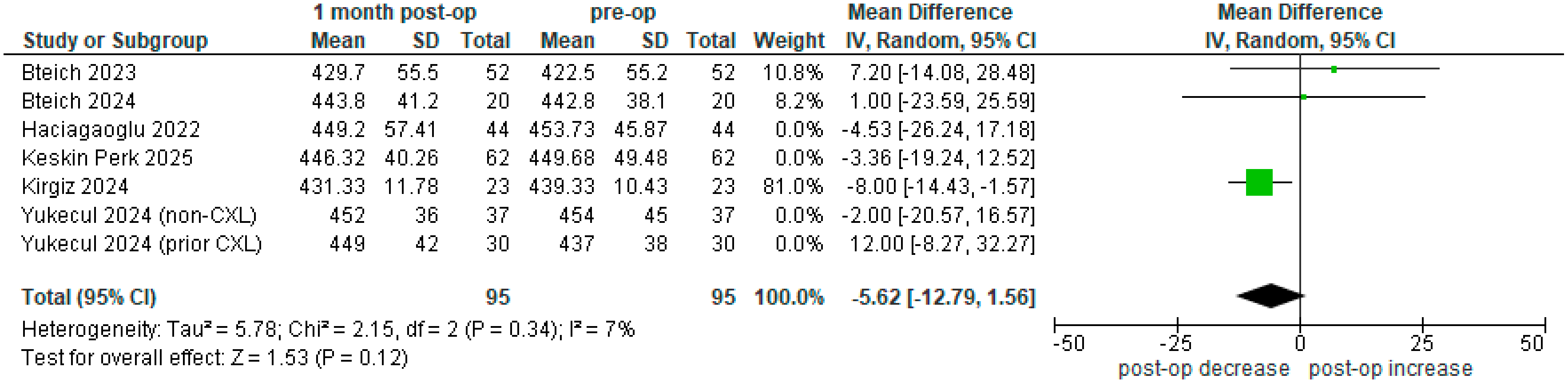

S1.3.1.4 Difference in pachymetry central point

Figure S1.3.1.4A Difference in pachymetry central point across 0 studies – HYDRATED

Figure S1.3.1.4B Difference in pachymetry central point across 0 studies – DEHYDRATED

S1.3.1.5 Difference in maximum keratometry

Figure S1.3.1.5A Difference in maximum keratometry across 0 studies - HYDRATED

Figure S1.3.1.5B Difference in maximum keratometry across 4 studies[1, 2, 5, 8] – DEHYDRATED

	1 month post-op			pre-op				Mean Difference
**Study or Subgroup**	**Mean**	**SD**	**Total**	**Mean**	**SD**	**Total**	**Weight**	**IV, Random, 95% CI**
Bteich 2023	54.17	8.22	52	58.09	7.92	52	24.8%	-3.92 [-7.02, -0.82]
Bteich 2024	53.3	6.22	20	57.21	7.53	20	21.7%	-3.91 [-8.19, 0.37]
Haciagaoglu 2022	55.46	6.97	44	57.56	7.06	44	0.0%	-2.10 [-5.03, 0.83]
Keskin Perk 2025	56.34	7.43	62	58.51	7.55	62	0.0%	-2.17 [-4.81, 0.47]
Kirgiz 2024	48.18	2.03	23	57.65	1.41	23	29.0%	-9.47 [-10.48, -8.46]
Mechleb 2024 (Kmax inf 75D)	51.93	5.79	6	54.43	4.09	6	18.0%	-2.50 [-8.17, 3.17]
Mechleb 2024 (Kmax sup 75D)	67.02	10.95	4	81.5	7.95	4	6.6%	-14.48 [-27.74, -1.22]
Nacaroglu 2023	56.75	7.72	49	59.2	7.66	49	0.0%	-2.45 [-5.50, 0.60]
Yukecul 2024 (non-CXL)	56.17	7.13	37	58.38	7.24	37	0.0%	-2.21 [-5.48, 1.06]
Yukecul 2024 (prior CXL)	55.28	6.26	30	57.22	6.68	30	0.0%	-1.94 [-5.22, 1.34]
**Total (95% CI)**			**105**			**105**	**100.0%**	**-5.96 [-9.82, -2.11]**

Heterogeneity: $Tau^2 = 13.11$; $Chi^2 = 21.39$, df = 4 (P = 0.0003); $I^2 = 81\%$
Test for overall effect: Z = 3.03 (P = 0.002)

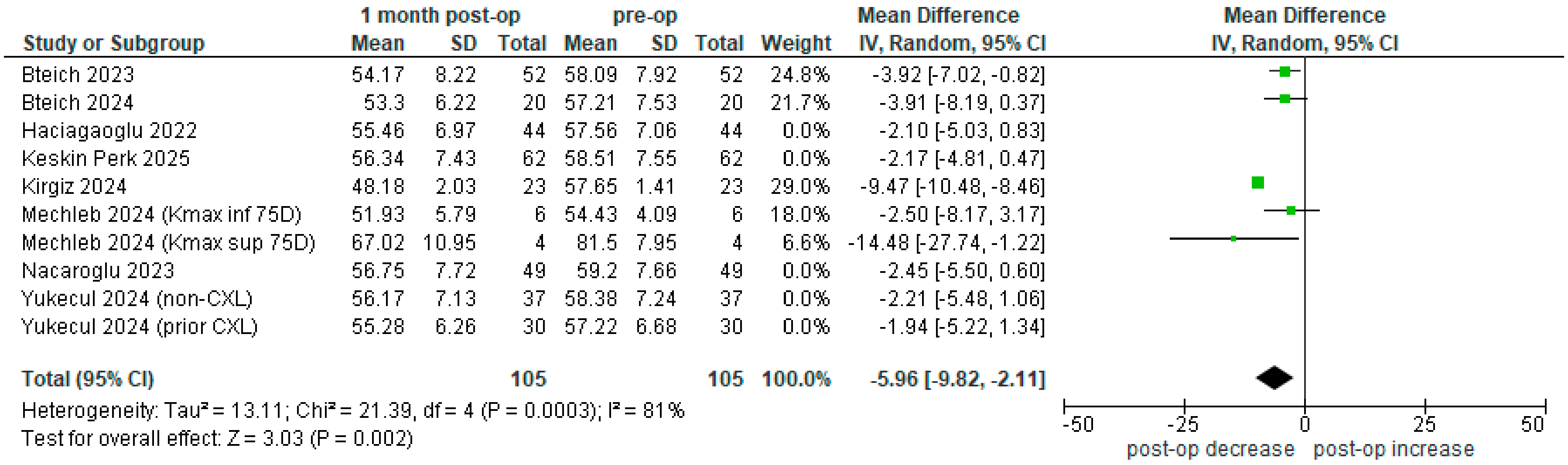

S1.3.1.6 Difference in mean simulated keratometry

Figure S1.3.1.6A Difference in mean simulated keratometry across 0 studies – HYDRATED

Figure S1.3.1.6B Difference in mean simulated keratometry across 4 studies [1, 2, 5, 8]– DEHYDRATED

	1 month post-op			pre-op				Mean Difference
**Study or Subgroup**	**Mean**	**SD**	**Total**	**Mean**	**SD**	**Total**	**Weight**	**IV, Random, 95% CI**
Bteich 2023	45.66	5.04	52	48.92	4.49	52	24.1%	-3.26 [-5.09, -1.43]
Bteich 2024	46.28	3.62	20	49.3	4.83	20	21.2%	-3.02 [-5.67, -0.37]
Haciagaoglu 2022	45.72	4.85	44	49.01	5.11	44	0.0%	-3.29 [-5.37, -1.21]
Keskin Perk 2025	46.22	4.98	62	49.17	4.97	62	0.0%	-2.95 [-4.70, -1.20]
Kirgiz 2024	42.91	1.32	23	49.86	1.16	23	27.0%	-6.95 [-7.67, -6.23]
Mechleb 2024 (Kmax inf 75D)	44.925	3.91	6	49.665	3.16	6	16.2%	-4.74 [-8.76, -0.72]
Mechleb 2024 (Kmax sup 75D)	55.325	5.36	4	68.01	2.215	4	11.5%	-12.69 [-18.37, -7.00]
Nacaroglu 2023	46.32	5.35	49	49.34	5.32	49	0.0%	-3.02 [-5.13, -0.91]
Yukecul 2024 (non-CXL)	44.52	4.8	37	49.11	4.66	37	0.0%	-4.59 [-6.75, -2.43]
Yukecul 2024 (prior CXL)	45.58	4.45	30	49.46	5.55	30	0.0%	-3.88 [-6.43, -1.33]
**Total (95% CI)**			**105**			**105**	**100.0%**	**-5.53 [-8.06, -3.00]**

Heterogeneity: Tau² = 6.05; Chi² = 25.03, df = 4 (P < 0.0001); I² = 84%
Test for overall effect: Z = 4.28 (P < 0.0001)

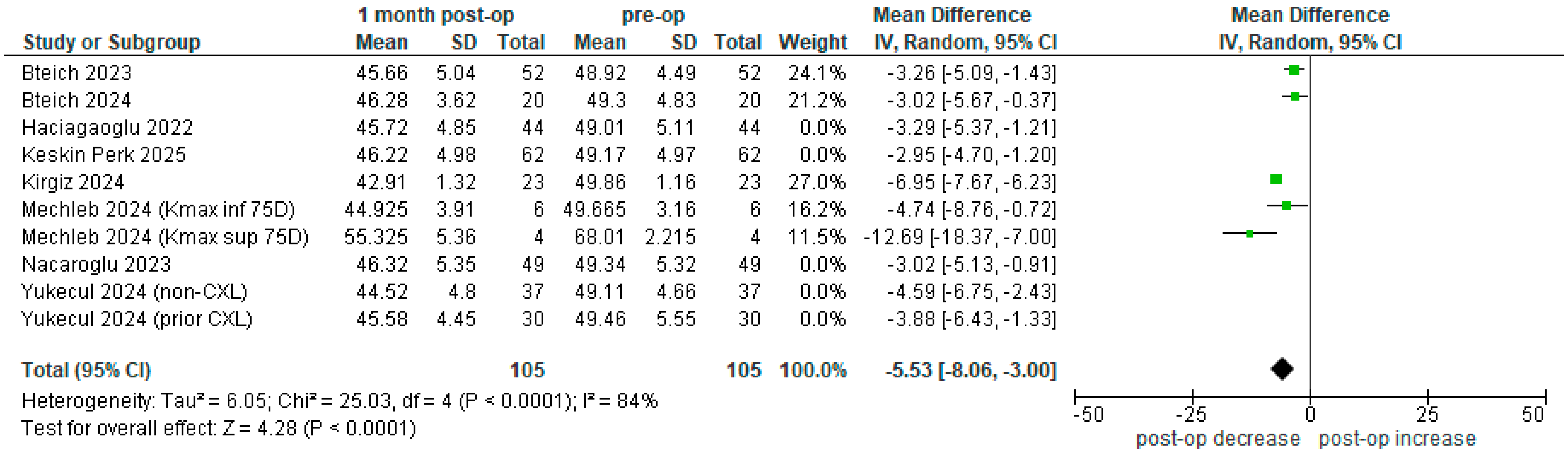

S1.3.1.7 Difference in total higher order aberrations

Figure S1.3.1.7A Difference in total higher order aberrations across 0 studies – HYDRATED

Figure S1.3.1.7B Difference in total higher order aberrations across 2 studies [1, 2] – DEHYDRATED

	1 month post-op			pre-op				Mean Difference
**Study or Subgroup**	**Mean**	**SD**	**Total**	**Mean**	**SD**	**Total**	**Weight**	**IV, Random, 95% CI**
Bteich 2023	1.74	0.85	52	2.2	1.13	52	61.9%	-0.46 [-0.84, -0.08]
Bteich 2024	1.53	0.59	20	1.92	0.95	20	38.1%	-0.39 [-0.88, 0.10]
**Total (95% CI)**			**72**			**72**	**100.0%**	**-0.43 [-0.74, -0.13]**

Heterogeneity: Tau² = 0.00; Chi² = 0.05, df = 1 (P = 0.83); I² = 0%
Test for overall effect: Z = 2.81 (P = 0.005)

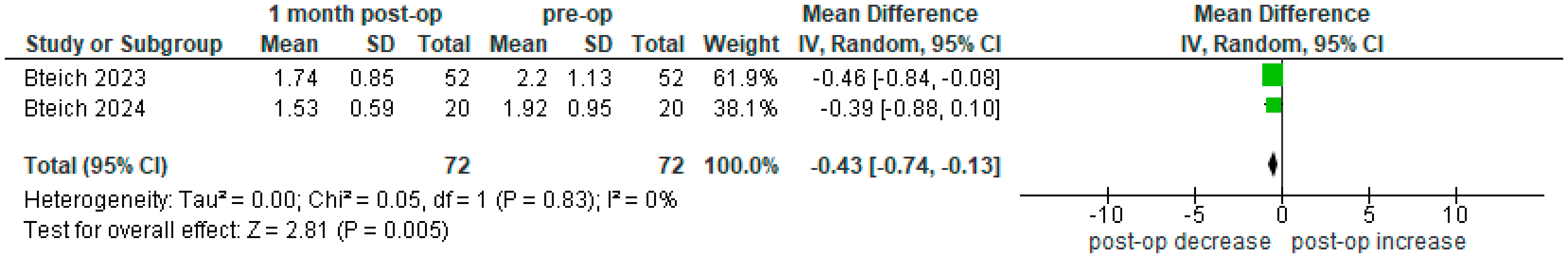

S1.3.1.8 Difference in spherical aberrations

S1.3.1.8A Difference in spherical aberrations – HYDRATED

Figure S1.3.1.8B Difference in spherical aberrations across 4 studies [1, 2, 5, 8]– DEHYDRATED

	1 month post-op			pre-op				Mean Difference
**Study or Subgroup**	**Mean**	**SD**	**Total**	**Mean**	**SD**	**Total**	**Weight**	**IV, Random, 95% CI**
Bteich 2023	0.51	0.59	52	0.15	0.45	52	28.7%	0.36 [0.16, 0.56]
Bteich 2024	0.31	0.39	20	0.05	0.42	20	25.6%	0.26 [0.01, 0.51]
Kirgiz 2024	0.8	0.1	23	0.38	0.07	23	36.3%	0.42 [0.37, 0.47]
Mechleb 2024 (Kmax inf 75D)	0.13	0.5	6	-0.94	0.75	6	7.9%	1.07 [0.35, 1.79]
Mechleb 2024 (Kmax sup 75D)	-1.35	0.69	4	-4.76	1.73	4	1.5%	3.41 [1.58, 5.24]
**Total (95% CI)**			**105**			**105**	**100.0%**	**0.46 [0.23, 0.69]**

Heterogeneity: Tau² = 0.04; Chi² = 15.30, df = 4 (P = 0.004); I² = 74%
Test for overall effect: Z = 3.92 (P < 0.0001)

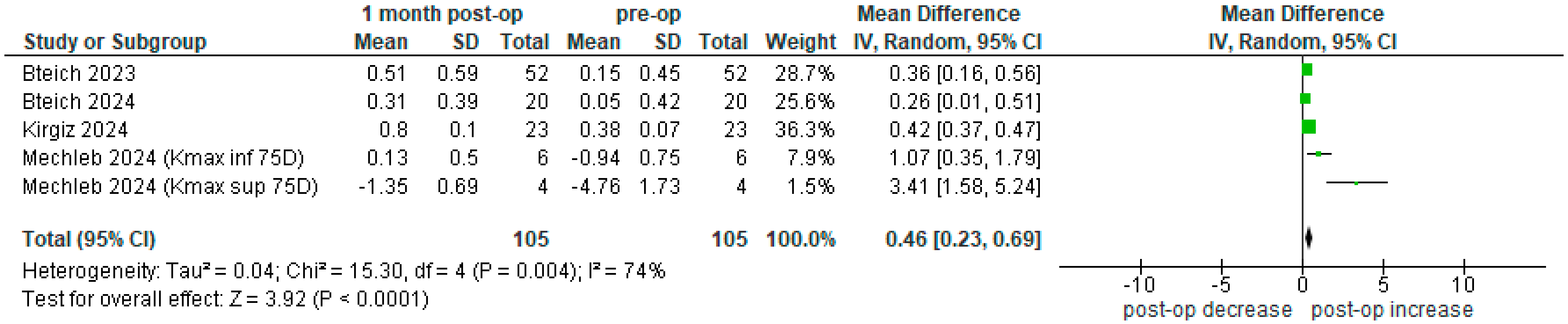

S1.3.1.9 Difference in vertical coma

Figure S1.3.1.9A Difference in vertical coma across 0 studies – HYDRATED

Figure S1.3.1.9B. Difference in vertical coma across 3 studies [1, 2, 8]– DEHYDRATED

	1 month post-op			pre-op				Mean Difference
**Study or Subgroup**	**Mean**	**SD**	**Total**	**Mean**	**SD**	**Total**	**Weight**	**IV, Random, 95% CI**
Bteich 2023	0.44	0.76	52	1.56	1.09	52	38.3%	-1.12 [-1.48, -0.76]
Bteich 2024	0.6	0.88	20	1.44	1.09	20	30.2%	-0.84 [-1.45, -0.23]
Mechleb 2024 (Kmax inf 75D)	1.16	1.19	6	2.03	0.52	6	18.9%	-0.87 [-1.91, 0.17]
Mechleb 2024 (Kmax sup 75D)	3.2	1.33	4	2.25	0.59	4	12.6%	0.95 [-0.48, 2.38]
**Total (95% CI)**			**82**			**82**	**100.0%**	**-0.73 [-1.32, -0.13]**

Heterogeneity: Tau² = 0.21; Chi² = 7.82, df = 3 (P = 0.05); I² = 62%

Test for overall effect: Z = 2.39 (P = 0.02)

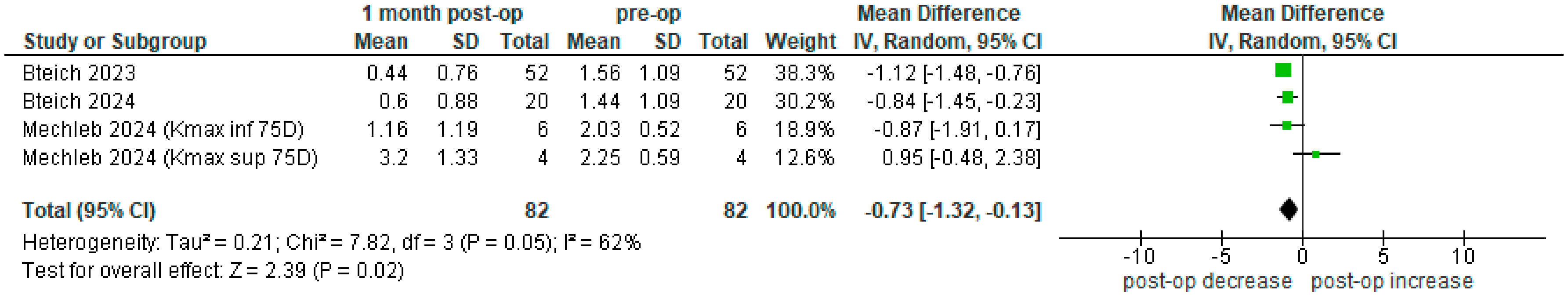

S1.3.1.10 Difference in trefoil

Figure S1.3.1.10A Difference in trefoil across 0 studies – HYDRATED

Figure S1.3.1.10B. Difference in trefoil across 3 studies [1, 2, 5] – DEHYDRATED

	1 month post-op			pre-op				Mean Difference
**Study or Subgroup**	**Mean**	**SD**	**Total**	**Mean**	**SD**	**Total**	**Weight**	**IV, Random, 95% CI**
Bteich 2023	-0.12	0.64	52	-0.39	0.52	52	20.3%	0.27 [0.05, 0.49]
Bteich 2024	0.088	0.59	20	-0.27	0.4	20	11.7%	0.36 [0.05, 0.67]
Kirgiz 2024	1.12	0.1	23	0.66	0.13	23	68.0%	0.46 [0.39, 0.53]
**Total (95% CI)**			**95**			**95**	**100.0%**	**0.41 [0.29, 0.52]**

Heterogeneity: Tau² = 0.00; Chi² = 2.82, df = 2 (P = 0.24); I² = 29%

Test for overall effect: Z = 6.99 (P < 0.00001)

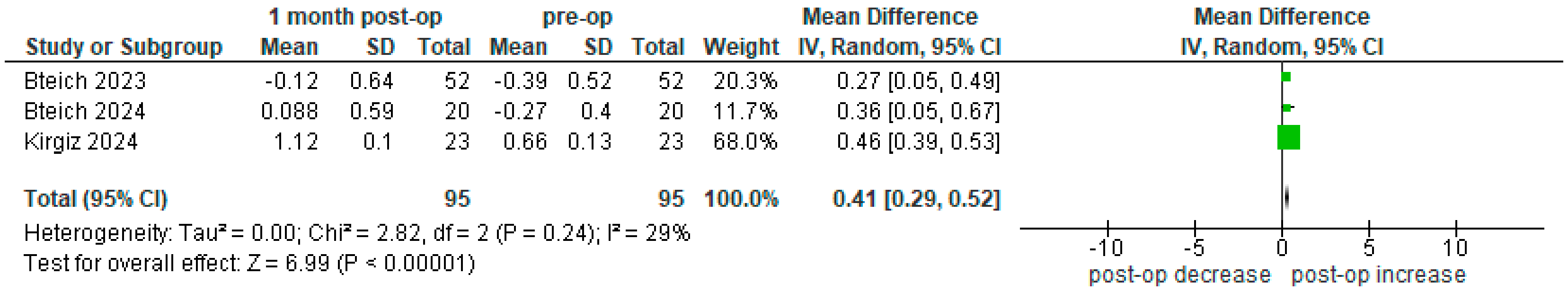

S3.1.1.11 Difference in total RMS

Figure S3.1.1.11A Difference in total RMS across 0 studies – HYDRATED

Figure S3.1.1.11B. Difference in total RMS across 3 studies [5, 8]– DEHYDRATED

	1 month post-op			pre-op				Mean Difference
**Study or Subgroup**	**Mean**	**SD**	**Total**	**Mean**	**SD**	**Total**	**Weight**	**IV, Random, 95% CI**
Kirgiz 2024	2.21	0.15	23	2.35	0.3	23	46.9%	-0.14 [-0.28, -0.00]
Mechleb 2024 (Kmax inf 75D)	8.45	5.87	6	14.1	4.04	6	31.6%	-5.65 [-11.35, 0.05]
Mechleb 2024 (Kmax sup 75D)	20.33	4.94	4	29.78	7.59	4	21.5%	-9.45 [-18.32, -0.58]
**Total (95% CI)**			**33**			**33**	**100.0%**	**-3.88 [-9.49, 1.72]**

Heterogeneity: Tau² = 17.41; Chi² = 7.81, df = 2 (P = 0.02); I² = 74%

Test for overall effect: Z = 1.36 (P = 0.17)

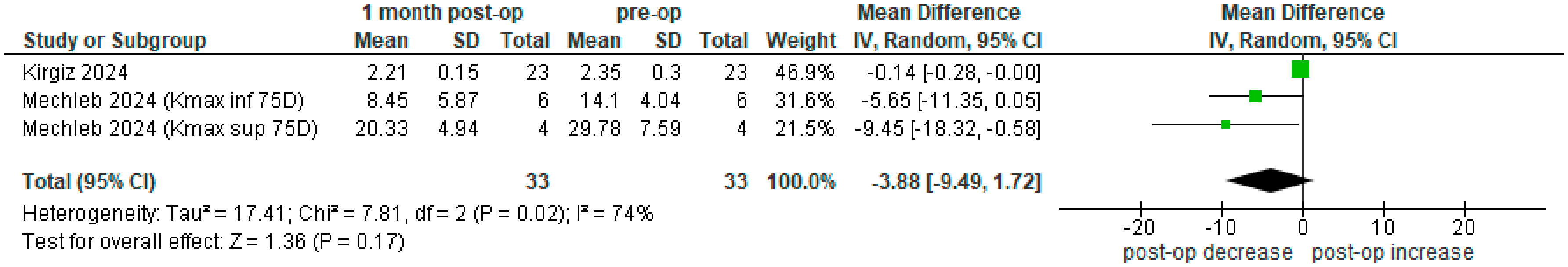

S1.3.2. 6 months postoperative versus preoperative

S1.3.2.1 Difference in uncorrected visual acuity

Figure S1.3.2.1A. Difference in uncorrected visual acuity across 1 study [11] – HYDRATED

	6 months post-op			pre-op				Mean Difference
**Study or Subgroup**	**Mean**	**SD**	**Total**	**Mean**	**SD**	**Total**	**Weight**	**IV, Random, 95% CI**
Mazzotta 2024	0.52	0	2	1	0	2		Not estimable
Hayashi 2025	0.68	0.54	5	1.39	0.28	5	0.0%	-0.71 [-1.24, -0.18]
Bteich 2023	0.68	0.23	4	0.93	0.31	4	0.0%	-0.25 [-0.63, 0.13]
Bteich 2023 ther refr	0.68	0.23	4	0.93	0.31	4	0.0%	-0.25 [-0.63, 0.13]
Yukecul 2024 (non-CXL)	0.31	0.21	37	0.77	0.76	37	0.0%	-0.46 [-0.71, -0.21]
Yukecul 2024 (prior CXL)	0.36	0.33	30	0.7	0.55	30	0.0%	-0.34 [-0.57, -0.11]
Haciagaoglu 2022	0.35	0.29	44	0.72	0.63	44	0.0%	-0.37 [-0.57, -0.17]
Bteich 2024	0.51	0.18	20	0.893	0.33	20	0.0%	-0.38 [-0.55, -0.22]
Nacaroglu 2023	0.4	0.24	49	0.91	0.5	49	0.0%	-0.51 [-0.67, -0.35]
Keskin Perk 2025	0.44	0.34	62	0.96	0.5	62	0.0%	-0.52 [-0.67, -0.37]
Kirgiz 2024	0.398	0.05	23	1.096	0.05	23	0.0%	-0.70 [-0.73, -0.67]
Colak 2025	0.48	0.21	4	1.2	0.5	4	100.0%	-0.72 [-1.25, -0.19]
**Total (95% CI)**			**4**			**4**	**100.0%**	**-0.72 [-1.25, -0.19]**

Heterogeneity: Not applicable
Test for overall effect: Z = 2.66 (P = 0.008)

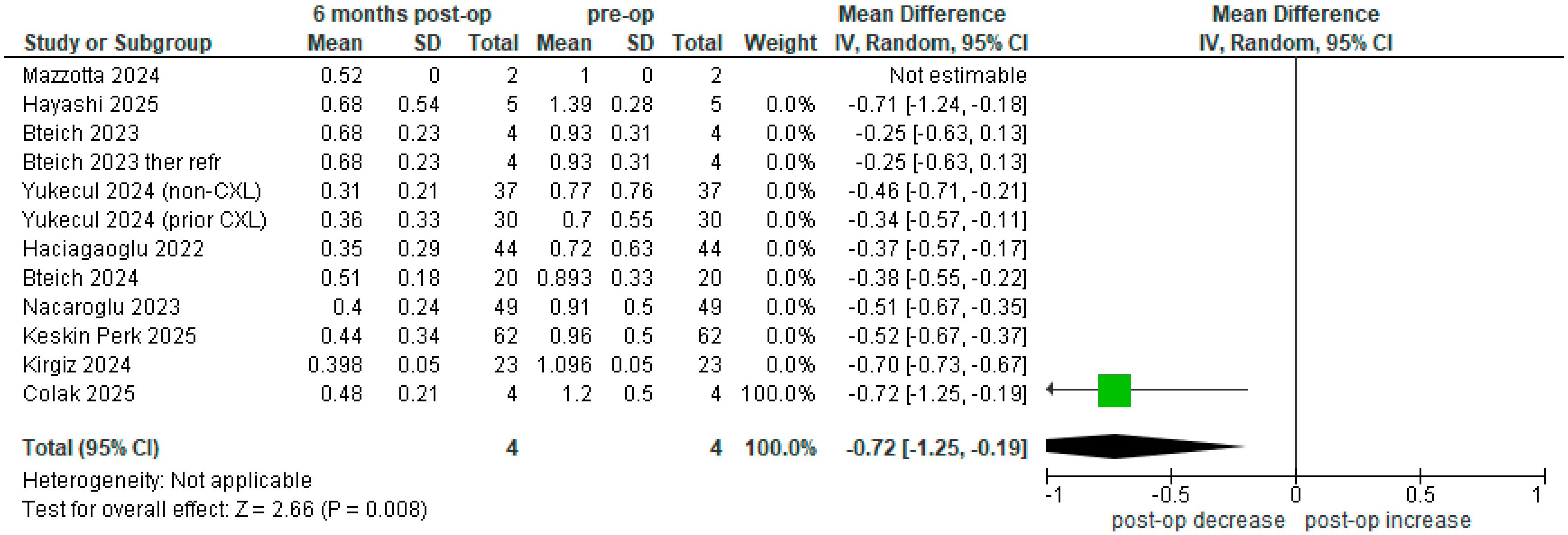

Figure S1.3.2.1B. Difference in uncorrected visual acuity across 4 studies [1, 2, 5, 12] – DEHYDRATED

	6 months post-op			pre-op				Mean Difference
**Study or Subgroup**	**Mean**	**SD**	**Total**	**Mean**	**SD**	**Total**	**Weight**	**IV, Random, 95% CI**
Mazzotta 2024	0.52	0	2	1	0	2		Not estimable
Hayashi 2025	0.68	0.54	5	1.39	0.28	5	0.0%	-0.71 [-1.24, -0.18]
Yukecul 2024 (prior CXL)	0.36	0.33	30	0.7	0.55	30	0.0%	-0.34 [-0.57, -0.11]
Haciagaoglu 2022	0.35	0.29	44	0.72	0.63	44	0.0%	-0.37 [-0.57, -0.17]
Bteich 2023	0.68	0.23	4	0.93	0.31	4	19.4%	-0.25 [-0.63, 0.13]
Bteich 2023 ther refr	0.68	0.23	4	0.93	0.31	4	19.4%	-0.25 [-0.63, 0.13]
Nacaroglu 2023	0.4	0.24	49	0.91	0.5	49	0.0%	-0.51 [-0.67, -0.35]
Bteich 2024	0.51	0.18	20	0.893	0.33	20	28.8%	-0.38 [-0.55, -0.22]
Kirgiz 2024	0.398	0.05	23	1.096	0.05	23	32.4%	-0.70 [-0.73, -0.67]
Keskin Perk 2025	0.44	0.34	62	0.96	0.5	62	0.0%	-0.52 [-0.67, -0.37]
Yukecul 2024 (non-CXL)	0.31	0.21	37	0.77	0.76	37	0.0%	-0.46 [-0.71, -0.21]
Colak 2025	0.48	0.21	4	1.2	0.5	4	0.0%	-0.72 [-1.25, -0.19]
**Total (95% CI)**			**51**			**51**	**100.0%**	**-0.43 [-0.70, -0.17]**

Heterogeneity: Tau² = 0.06; Chi² = 23.84, df = 3 (P < 0.0001); I² = 87%
Test for overall effect: Z = 3.24 (P = 0.001)

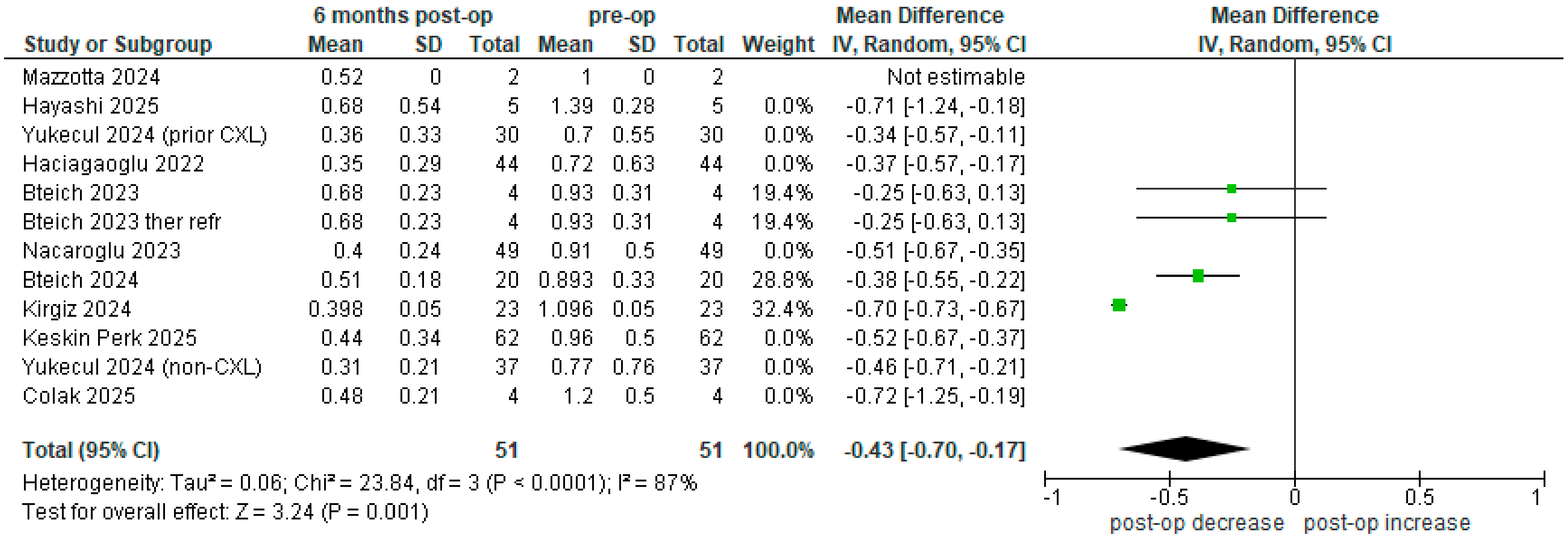

S1.3.2.2 Difference in best corrected visual acuity

Figure S1.3.2.2A Difference in best corrected visual acuity across 1 study [2] – HYDRATED

	6 months post-op			pre-op				Mean Difference
**Study or Subgroup**	**Mean**	**SD**	**Total**	**Mean**	**SD**	**Total**	**Weight**	**IV, Random, 95% CI**
Bteich 2023 ther refr	0.01	0.02	4	0.34	0.16	4	0.0%	-0.33 [-0.49, -0.17]
Bteich 2024	0.19	0.21	20	0.57	0.18	20	0.0%	-0.38 [-0.50, -0.26]
Colak 2025	0.4	0.14	4	0.53	0.13	4	100.0%	-0.13 [-0.32, 0.06]
Coscarelli 2024	0.301	0	3	1.06	0.22	3		Not estimable
Haciagaoglu 2022	0.25	0.22	44	0.54	0.37	44	0.0%	-0.29 [-0.42, -0.16]
Hayashi 2025	0.17	0.16	5	0.74	0.44	5	0.0%	-0.57 [-0.98, -0.16]
Keskin Perk 2025	0.25	0.24	62	0.72	0.47	62	0.0%	-0.47 [-0.60, -0.34]
Kirgiz 2024	0.167	0.05	23	0.602	0.3	23	0.0%	-0.43 [-0.56, -0.31]
Mechleb 2025	0.17	0.18	33	0.36	0.25	79	0.0%	-0.19 [-0.27, -0.11]
Nacaroglu 2023	0.31	0.09	49	0.87	0.2	49	0.0%	-0.56 [-0.62, -0.50]
Yukecul 2024 (non-CXL)	0.17	0.18	37	0.51	0.29	37	0.0%	-0.34 [-0.45, -0.23]
Yukecul 2024 (prior CXL)	0.27	0.23	30	0.55	0.39	30	0.0%	-0.28 [-0.44, -0.12]
**Total (95% CI)**			**4**			**4**	**100.0%**	**-0.13 [-0.32, 0.06]**

Heterogeneity: Not applicable
Test for overall effect: Z = 1.36 (P = 0.17)

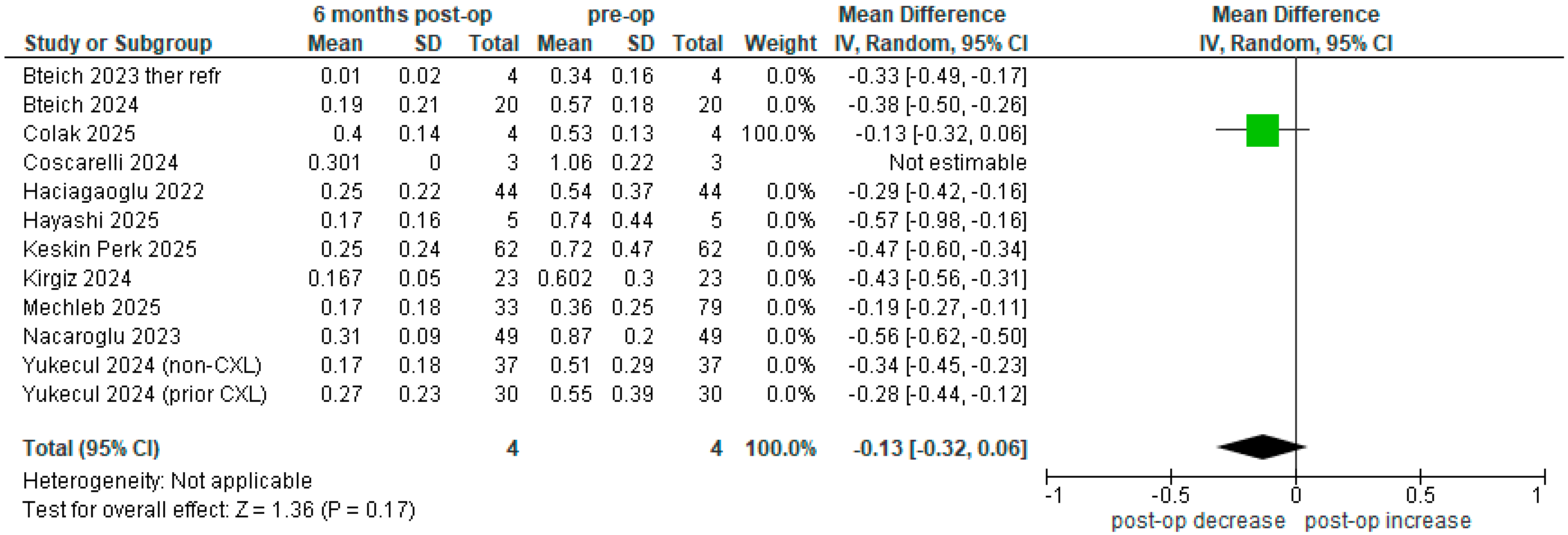

S1.3.2.2B Difference in best corrected visual acuity across 4 studies [1, 5, 12, 14]- DEHYDRATED

	6 months post-op			pre-op				Mean Difference
**Study or Subgroup**	**Mean**	**SD**	**Total**	**Mean**	**SD**	**Total**	**Weight**	**IV, Random, 95% CI**
Bteich 2023 ther refr	0.01	0.02	4	0.34	0.16	4	21.4%	-0.33 [-0.49, -0.17]
Bteich 2024	0.19	0.21	20	0.57	0.18	20	25.0%	-0.38 [-0.50, -0.26]
Colak 2025	0.4	0.14	4	0.53	0.13	4	0.0%	-0.13 [-0.32, 0.06]
Coscarelli 2024	0.301	0	3	1.06	0.22	3		Not estimable
Haciagaoglu 2022	0.25	0.22	44	0.54	0.37	44	0.0%	-0.29 [-0.42, -0.16]
Hayashi 2025	0.17	0.16	5	0.74	0.44	5	0.0%	-0.57 [-0.98, -0.16]
Keskin Perk 2025	0.25	0.24	62	0.72	0.47	62	0.0%	-0.47 [-0.60, -0.34]
Kirgiz 2024	0.167	0.05	23	0.602	0.3	23	24.7%	-0.43 [-0.56, -0.31]
Mechleb 2025	0.17	0.18	33	0.36	0.25	79	28.8%	-0.19 [-0.27, -0.11]
Nacaroglu 2023	0.31	0.09	49	0.87	0.2	49	0.0%	-0.56 [-0.62, -0.50]
Yukecul 2024 (non-CXL)	0.17	0.18	37	0.51	0.29	37	0.0%	-0.34 [-0.45, -0.23]
Yukecul 2024 (prior CXL)	0.27	0.23	30	0.55	0.39	30	0.0%	-0.28 [-0.44, -0.12]
**Total (95% CI)**			**83**			**129**	**100.0%**	**-0.33 [-0.45, -0.20]**

Heterogeneity: Tau² = 0.01; Chi² = 13.16, df = 3 (P = 0.004); I² = 77%
Test for overall effect: Z = 5.21 (P < 0.00001)

Mean Difference
IV, Random, 95% CI

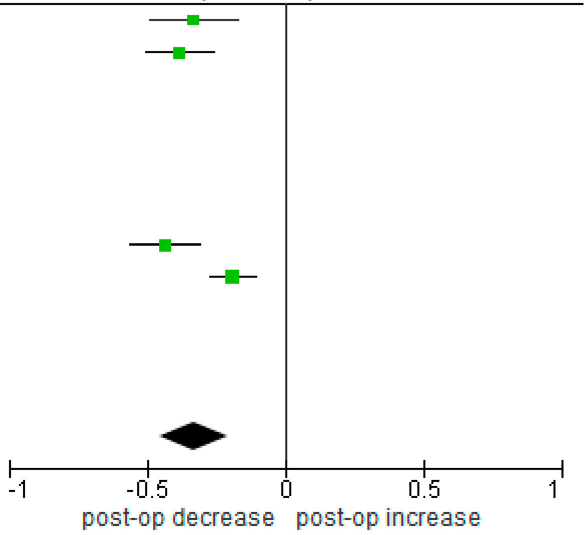

S1.3.2.3 Pachymetry thinnest

Figure S1.3.2.3A Pachymetry thinnest across 0 studies – HYDRATED

Figure S1.3.2.3B Pachymetry thinnest across 2 studies [2, 5]– DEHYDRATED

	6 months post-op			pre-op				Mean Difference
**Study or Subgroup**	**Mean**	**SD**	**Total**	**Mean**	**SD**	**Total**	**Weight**	**IV, Random, 95% CI**
Bteich 2024	447.5	51.5	20	442.8	38.1	20	8.1%	4.70 [-23.38, 32.78]
Haciagaoglu 2022	449.48	61.06	44	453.73	45.87	44	0.0%	-4.25 [-26.82, 18.32]
Keskin Perk 2025	445.7	47.35	62	449.68	49.48	62	0.0%	-3.98 [-21.03, 13.07]
Kirgiz 2024	445.5	17.5	23	439.33	10.43	23	91.9%	6.17 [-2.16, 14.50]
Mazzotta 2024	432	76.37	2	402.5	57.28	2	0.0%	29.50 [-102.80, 161.80]
Yukecul 2024 (non-CXL)	446	44	37	454	45	37	0.0%	-8.00 [-28.28, 12.28]
Yukecul 2024 (prior CXL)	452	41	30	437	38	30	0.0%	15.00 [-5.00, 35.00]
**Total (95% CI)**			**43**			**43**	**100.0%**	**6.05 [-1.93, 14.03]**

Heterogeneity: Tau² = 0.00; Chi² = 0.01, df = 1 (P = 0.92); I² = 0%

Test for overall effect: Z = 1.49 (P = 0.14)

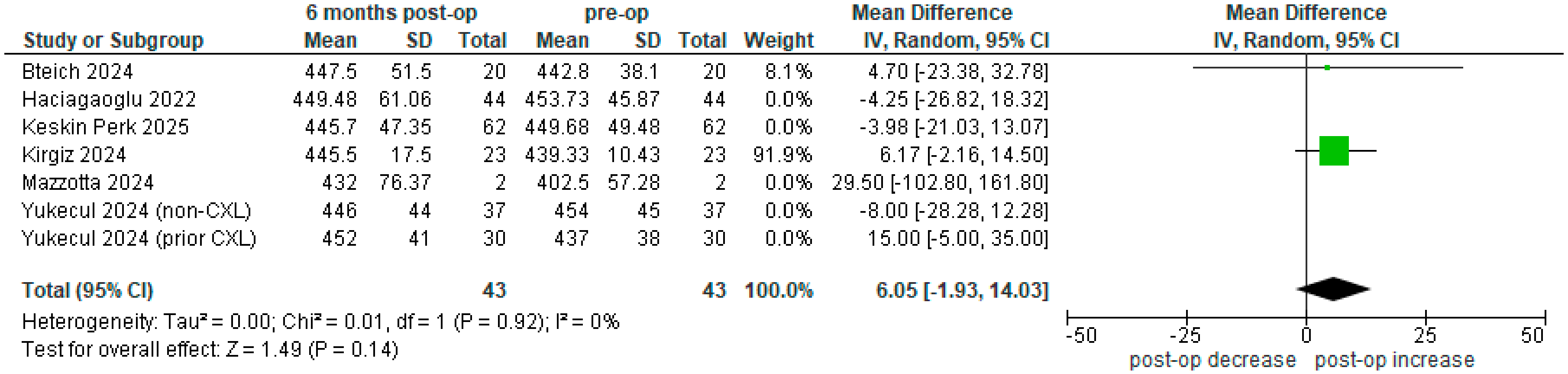

S1.3.2.4 Pachymetry central point

Figure S1.3.2.4A Pachymetry central point across 1 study [11] – HYDRATED

	6 months post-op			pre-op				Mean Difference
**Study or Subgroup**	**Mean**	**SD**	**Total**	**Mean**	**SD**	**Total**	**Weight**	**IV, Random, 95% CI**
Colak 2025	452.5	47.03	4	417.25	40.73	4	100.0%	35.25 [-25.72, 96.22]
Hayashi 2025	514.6	62.63	5	505.6	55.4	5	0.0%	9.00 [-64.29, 82.29]
Mechleb 2025	466	39	33	455	41	79	0.0%	11.00 [-5.09, 27.09]
Nacaroglu 2023	447.46	50.57	49	463.62	54.88	49	0.0%	-16.16 [-37.06, 4.74]
**Total (95% CI)**			**4**			**4**	**100.0%**	**35.25 [-25.72, 96.22]**

Heterogeneity: Not applicable

Test for overall effect: Z = 1.13 (P = 0.26)

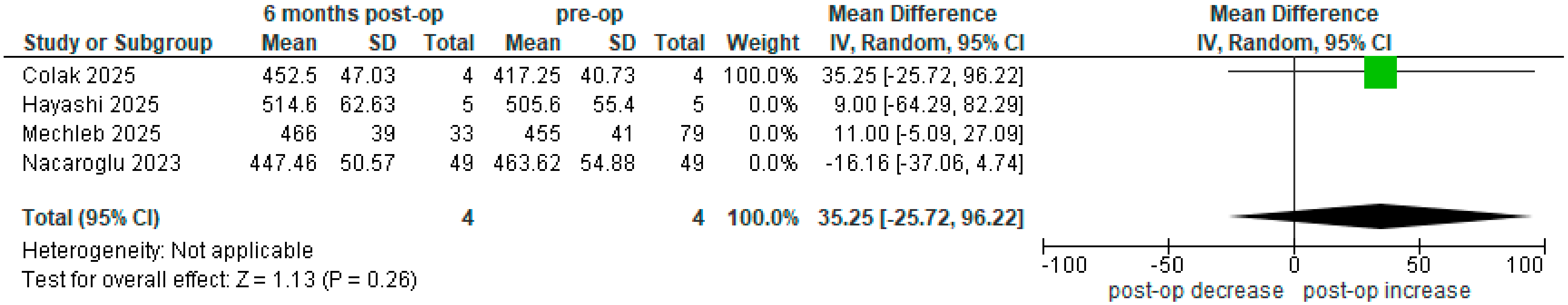

Figure S1.3.2.4B Pachymetry central point across 1 study [14]– DEHYDRATED

	6 months post-op			pre-op				Mean Difference
Study or Subgroup	Mean	SD	Total	Mean	SD	Total	Weight	IV, Random, 95% CI
Colak 2025	452.5	47.03	4	417.25	40.73	4	0.0%	35.25 [-25.72, 96.22]
Hayashi 2025	514.6	62.63	5	505.6	55.4	5	0.0%	9.00 [-64.29, 82.29]
Mechleb 2025	466	39	33	455	41	79	100.0%	11.00 [-5.09, 27.09]
Nacaroglu 2023	447.46	50.57	49	463.62	54.88	49	0.0%	-16.16 [-37.06, 4.74]
**Total (95% CI)**			**33**			**79**	**100.0%**	**11.00 [-5.09, 27.09]**

Heterogeneity: Not applicable
Test for overall effect: Z = 1.34 (P = 0.18)

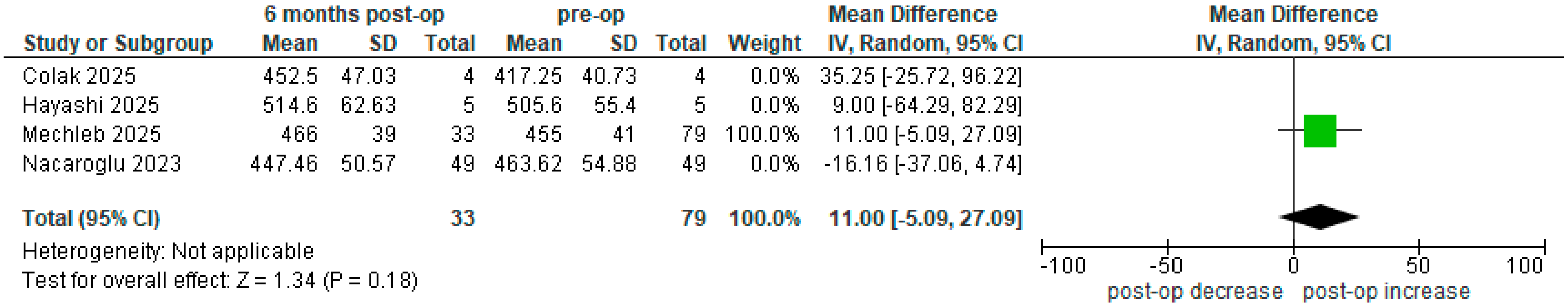

S1.3.2.5 Maximum keratometry

Figure S1.3.2.5A Maximum keratometry across 1 study [11] – HYDRATED

	6 months post-op			pre-op				Mean Difference
**Study or Subgroup**	**Mean**	**SD**	**Total**	**Mean**	**SD**	**Total**	**Weight**	**IV, Random, 95% CI**
Bteich 2023 ther refr	47.89	3.05	4	50.02	1.99	4	0.0%	-2.13 [-5.70, 1.44]
Bteich 2024	52.38	6.02	20	57.21	7.53	20	0.0%	-4.83 [-9.06, -0.60]
Colak 2025	55.88	3.64	4	56.55	3.31	4	100.0%	-0.67 [-5.49, 4.15]
Haciagaoglu 2022	55.4	6.73	44	57.56	7.06	44	0.0%	-2.16 [-5.04, 0.72]
Hayashi 2025	59.62	6.78	5	63.58	8.26	5	0.0%	-3.96 [-13.33, 5.41]
Keskin Perk 2025	55.27	6.17	62	58.51	7.55	62	0.0%	-3.24 [-5.67, -0.81]
Kirgiz 2024	46.96	1.88	23	57.65	1.41	23	0.0%	-10.69 [-11.65, -9.73]
Mazzotta 2024	50.81	4.53	2	56.21	8.78	2	0.0%	-5.40 [-19.09, 8.29]
Mechleb 2025	55.6	6.4	33	57.6	6.9	79	0.0%	-2.00 [-4.66, 0.66]
Nacaroglu 2023	55.83	6.96	49	59.2	7.66	49	0.0%	-3.37 [-6.27, -0.47]
Yukecul 2024 (non-CXL)	55.66	6.29	37	58.38	7.24	37	0.0%	-2.72 [-5.81, 0.37]
Yukecul 2024 (prior CXL)	54.11	6.65	30	57.22	6.68	30	0.0%	-3.11 [-6.48, 0.26]
**Total (95% CI)**			**4**			**4**	**100.0%**	**-0.67 [-5.49, 4.15]**

Heterogeneity: Not applicable
Test for overall effect: Z = 0.27 (P = 0.79)

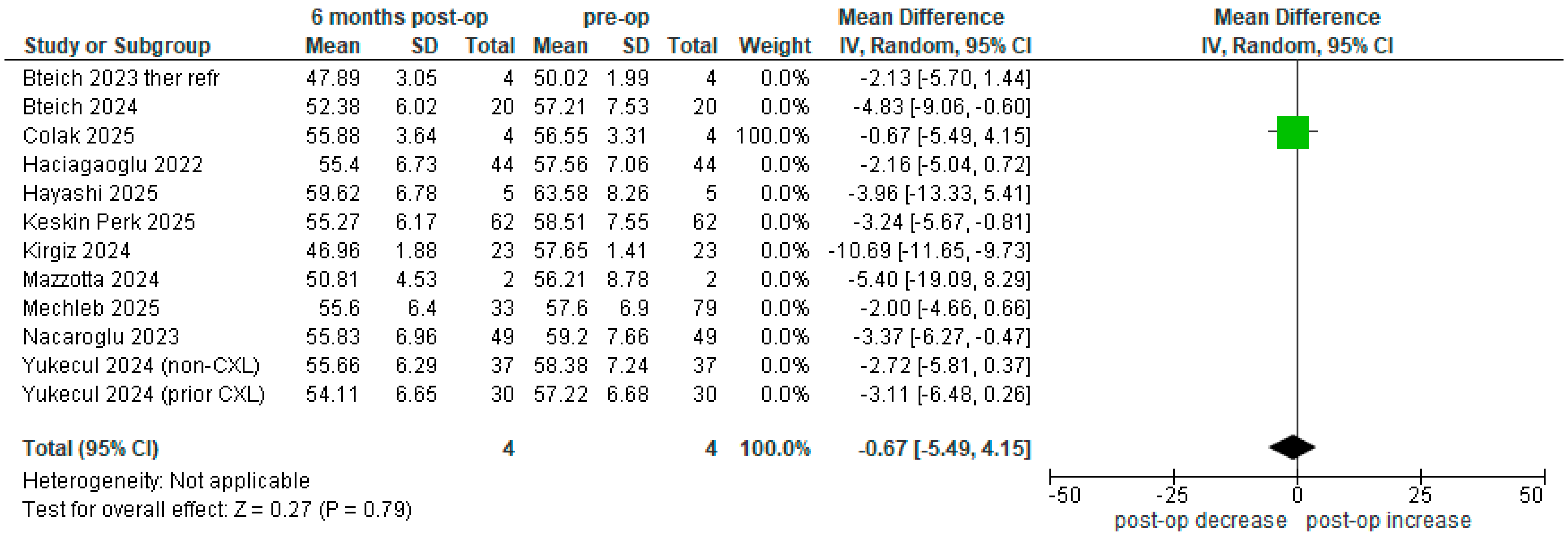

Figure S1.3.2.5B Maximum keratometry across 4 studies [2, 5, 12, 14]– DEHYDRATED

	6 months post-op			pre-op				Mean Difference
**Study or Subgroup**	**Mean**	**SD**	**Total**	**Mean**	**SD**	**Total**	**Weight**	**IV, Random, 95% CI**
Bteich 2023 ther refr	47.89	3.05	4	50.02	1.99	4	24.3%	-2.13 [-5.70, 1.44]
Bteich 2024	52.38	6.02	20	57.21	7.53	20	23.4%	-4.83 [-9.06, -0.60]
Colak 2025	55.88	3.64	4	56.55	3.31	4	0.0%	-0.67 [-5.49, 4.15]
Haciagaoglu 2022	55.4	6.73	44	57.56	7.06	44	0.0%	-2.16 [-5.04, 0.72]
Hayashi 2025	59.62	6.78	5	63.58	8.26	5	0.0%	-3.96 [-13.33, 5.41]
Keskin Perk 2025	55.27	6.17	62	58.51	7.55	62	0.0%	-3.24 [-5.67, -0.81]
Kirgiz 2024	46.96	1.88	23	57.65	1.41	23	26.8%	-10.69 [-11.65, -9.73]
Mazzotta 2024	50.81	4.53	2	56.21	8.78	2	0.0%	-5.40 [-19.09, 8.29]
Mechleb 2025	55.6	6.4	33	57.6	6.9	79	25.5%	-2.00 [-4.66, 0.66]
Nacaroglu 2023	55.83	6.96	49	59.2	7.66	49	0.0%	-3.37 [-6.27, -0.47]
Yukecul 2024 (non-CXL)	55.66	6.29	37	58.38	7.24	37	0.0%	-2.72 [-5.81, 0.37]
Yukecul 2024 (prior CXL)	54.11	6.65	30	57.22	6.68	30	0.0%	-3.11 [-6.48, 0.26]
**Total (95% CI)**			**80**			**126**	**100.0%**	**-5.03 [-10.55, 0.50]**

Heterogeneity: Tau² = 29.34; Chi² = 56.43, df = 3 (P < 0.00001); I² = 95%
Test for overall effect: Z = 1.78 (P = 0.07)

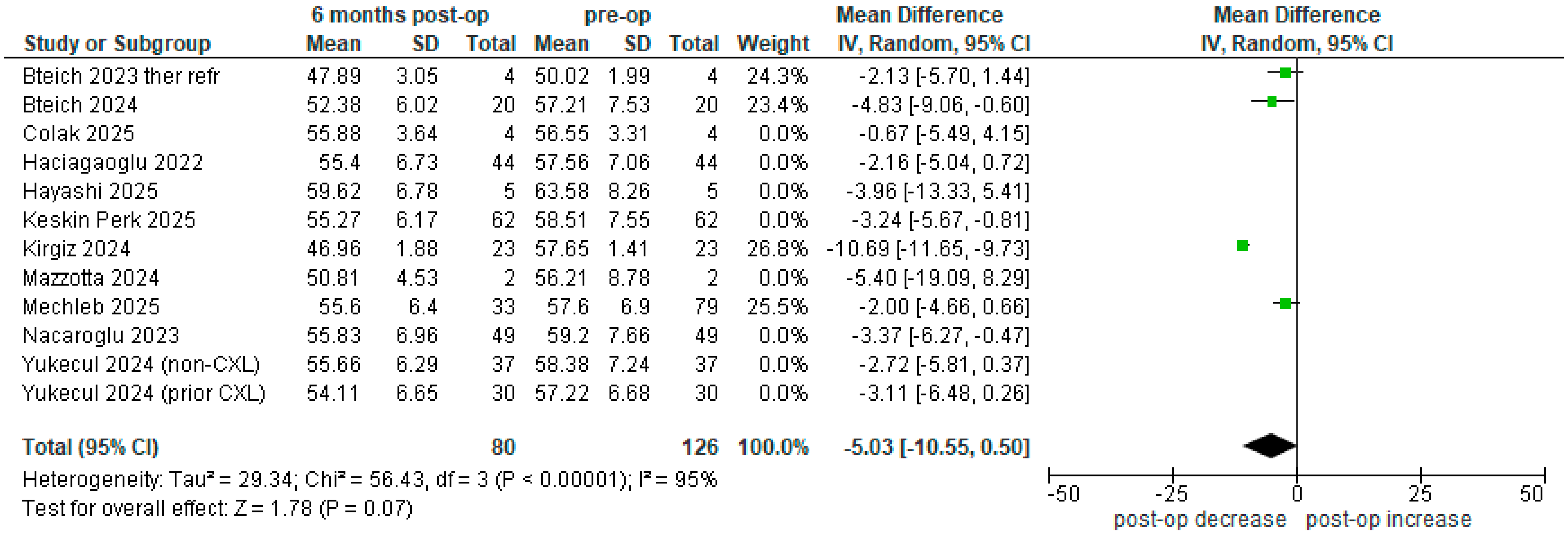

S1.3.2.6 Mean simulated keratometry

Figure S1.3.2.6A Mean simulated keratometry across 1 study [11] – HYDRATED

Study or Subgroup	6 months post-op Mean	SD	Total	pre-op Mean	SD	Total	Weight	Mean Difference IV, Random, 95% CI
Bteich 2023 ther refr	43.18	1.52	4	44.32	0.95	4	0.0%	-1.14 [-2.90, 0.62]
Bteich 2024	45.45	3.57	20	49.3	4.83	20	0.0%	-3.85 [-6.48, -1.22]
Colak 2025	45.88	3.27	4	49.05	3.44	4	100.0%	-3.17 [-7.82, 1.48]
Coscarelli 2024	44.28	0.87	3	55.78	2.84	3	0.0%	-11.50 [-14.86, -8.14]
Haciagaoglu 2022	45.71	4.8	44	49.01	5.11	44	0.0%	-3.30 [-5.37, -1.23]
Keskin Perk 2025	45.75	4.47	62	49.17	4.97	62	0.0%	-3.42 [-5.08, -1.76]
Kirgiz 2024	42.4	1.37	23	49.86	1.16	23	0.0%	-7.46 [-8.19, -6.73]
Mechleb 2025	45.3	5	33	49.2	6.5	79	0.0%	-3.90 [-6.13, -1.67]
Nacaroglu 2023	45.76	5.02	49	49.34	5.32	49	0.0%	-3.58 [-5.63, -1.53]
Yukecul 2024 (non-CXL)	44.45	4.2	37	49.11	4.66	37	0.0%	-4.66 [-6.68, -2.64]
Yukecul 2024 (prior CXL)	46.34	4.46	30	49.46	5.55	30	0.0%	-3.12 [-5.67, -0.57]
**Total (95% CI)**			**4**			**4**	**100.0%**	**-3.17 [-7.82, 1.48]**

Heterogeneity: Not applicable

Test for overall effect: Z = 1.34 (P = 0.18)

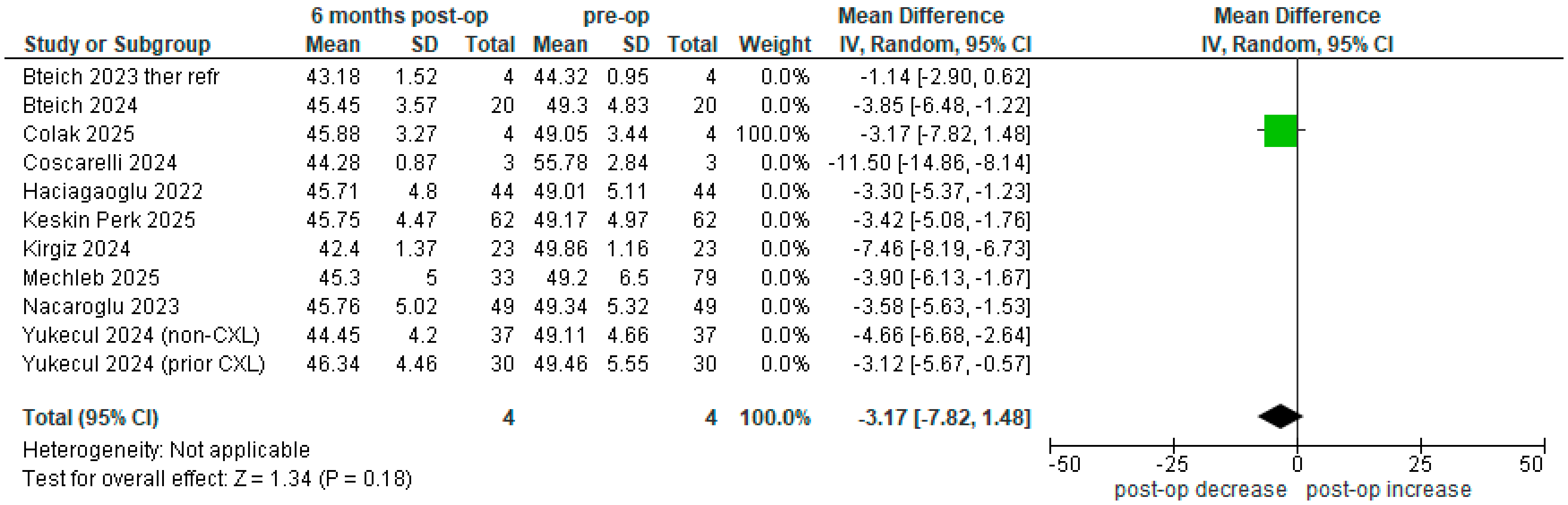

Figure S1.3.2.6B Mean simulated keratometry across 5 studies [2, 5, 12-14]– DEHYDRATED

	6 months post-op			pre-op				Mean Difference
**Study or Subgroup**	**Mean**	**SD**	**Total**	**Mean**	**SD**	**Total**	**Weight**	**IV, Random, 95% CI**
Bteich 2023 ther refr	43.18	1.52	4	44.32	0.95	4	20.8%	-1.14 [-2.90, 0.62]
Bteich 2024	45.45	3.57	20	49.3	4.83	20	19.3%	-3.85 [-6.48, -1.22]
Colak 2025	45.88	3.27	4	49.05	3.44	4	0.0%	-3.17 [-7.82, 1.48]
Coscarelli 2024	44.28	0.87	3	55.78	2.84	3	17.8%	-11.50 [-14.86, -8.14]
Haciagaoglu 2022	45.71	4.8	44	49.01	5.11	44	0.0%	-3.30 [-5.37, -1.23]
Keskin Perk 2025	45.75	4.47	62	49.17	4.97	62	0.0%	-3.42 [-5.08, -1.76]
Kirgiz 2024	42.4	1.37	23	49.86	1.16	23	22.0%	-7.46 [-8.19, -6.73]
Mechleb 2025	45.3	5	33	49.2	6.5	79	20.1%	-3.90 [-6.13, -1.67]
Nacaroglu 2023	45.76	5.02	49	49.34	5.32	49	0.0%	-3.58 [-5.63, -1.53]
Yukecul 2024 (non-CXL)	44.45	4.2	37	49.11	4.66	37	0.0%	-4.66 [-6.68, -2.64]
Yukecul 2024 (prior CXL)	46.34	4.46	30	49.46	5.55	30	0.0%	-3.12 [-5.67, -0.57]
**Total (95% CI)**			**83**			**129**	**100.0%**	**-5.45 [-8.63, -2.28]**

Heterogeneity: Tau² = 11.79; Chi² = 59.72, df = 4 (P < 0.00001); I² = 93%
Test for overall effect: Z = 3.37 (P = 0.0008)

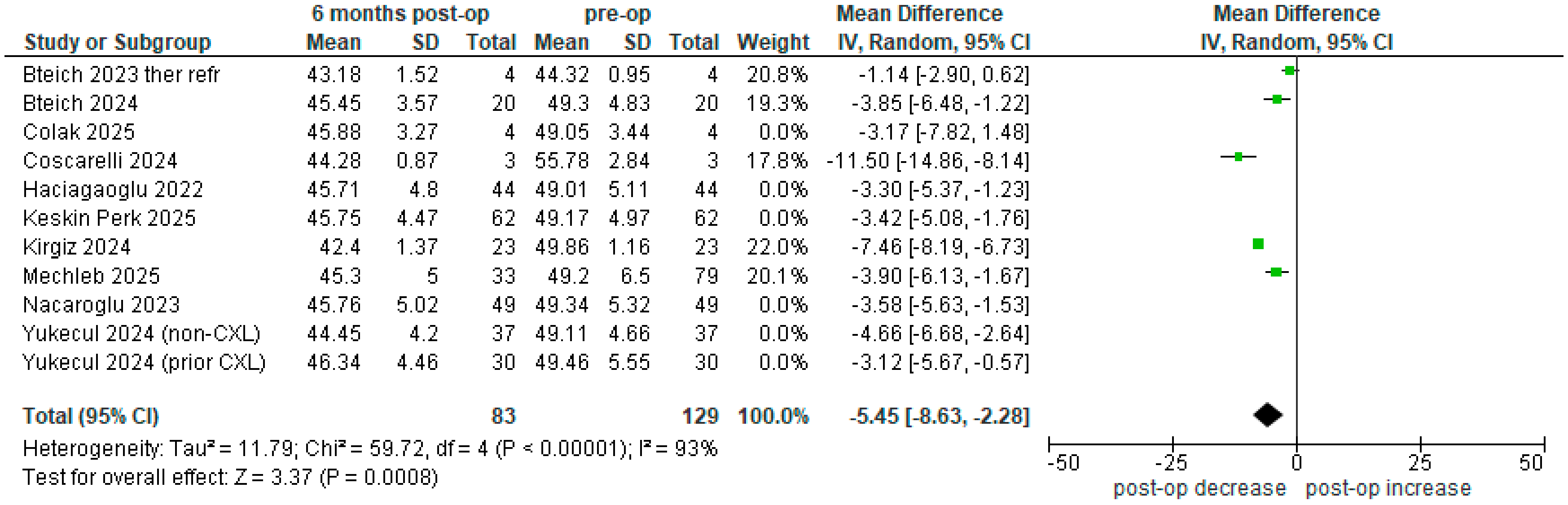

S1.3.2.7 Total higher order aberrations

Figure S1.3.2.7A Total higher order aberrations across 0 studies – HYDRATED

Figure S1.3.2.7B Total higher order aberrations across 2 studies [2, 14]– DEHYDRATED

	6 months post-op			pre-op				Mean Difference
**Study or Subgroup**	**Mean**	**SD**	**Total**	**Mean**	**SD**	**Total**	**Weight**	**IV, Random, 95% CI**
Bteich 2024	1.39	0.62	20	1.92	0.95	20	72.9%	-0.53 [-1.03, -0.03]
Hayashi 2025	4.24	1.61	5	5.59	1.55	5	0.0%	-1.35 [-3.31, 0.61]
Mechleb 2025	3.47	2.08	33	3.78	1.82	79	27.1%	-0.31 [-1.13, 0.51]
**Total (95% CI)**			**53**			**99**	**100.0%**	**-0.47 [-0.89, -0.05]**

Heterogeneity: Tau² = 0.00; Chi² = 0.20, df = 1 (P = 0.65); I² = 0%

Test for overall effect: Z = 2.17 (P = 0.03)

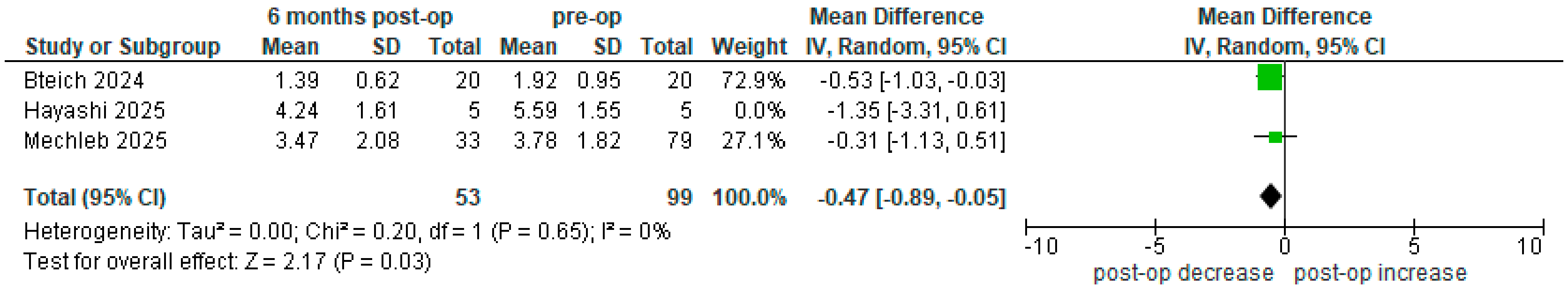

S1.3.2.8 Spherical aberration

S1.3.2.8A Spherical aberration across 0 studies – HYDRATED

Figure S1.3.2.8B Spherical aberration across 3 studies [2, 5, 12]– DEHYDRATED

	6 months post-op			pre-op				Mean Difference
**Study or Subgroup**	**Mean**	**SD**	**Total**	**Mean**	**SD**	**Total**	**Weight**	**IV, Random, 95% CI**
Bteich 2023 ther refr	-0.09	0.27	4	0.26	0.08	4	31.0%	-0.35 [-0.63, -0.07]
Bteich 2024	0.32	0.46	20	0.05	0.42	20	31.1%	0.27 [-0.00, 0.54]
Kirgiz 2024	0.65	0.1	23	0.38	0.07	23	37.8%	0.27 [0.22, 0.32]
**Total (95% CI)**			**47**			**47**	**100.0%**	**0.08 [-0.28, 0.43]**

Heterogeneity: Tau² = 0.09; Chi² = 18.80, df = 2 (P < 0.0001); I² = 89%

Test for overall effect: Z = 0.43 (P = 0.67)

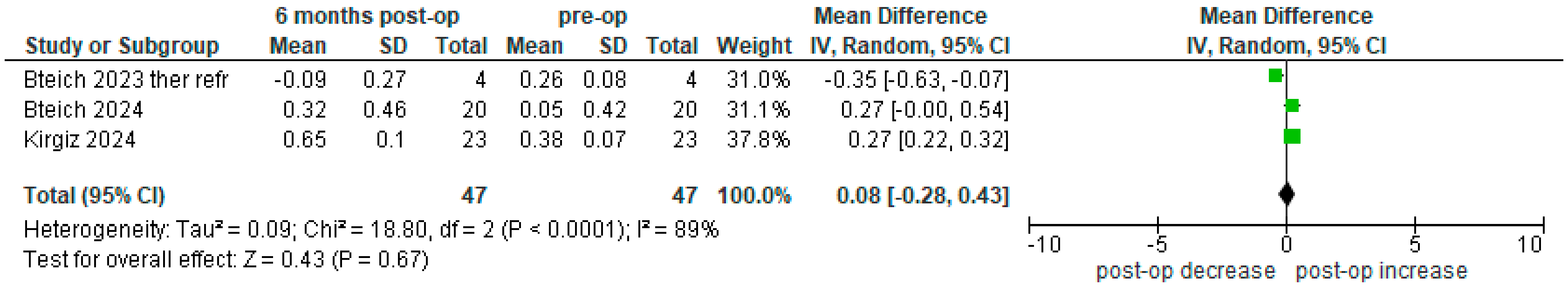

S1.3.2.9 Vertical coma

S1.3.2.9A Vertical coma across 0 studies – HYDRATED

Figure S1.3.2.9B Vertical coma across 2 studies [2, 12] – DEHYDRATED

	6 months post-op			pre-op				Mean Difference
**Study or Subgroup**	**Mean**	**SD**	**Total**	**Mean**	**SD**	**Total**	**Weight**	**IV, Random, 95% CI**
Bteich 2023 ther refr	0.21	0.19	4	1.05	0.21	4	80.6%	-0.84 [-1.12, -0.56]
Bteich 2024	0.53	0.69	20	1.44	1.09	20	19.4%	-0.91 [-1.48, -0.34]
**Total (95% CI)**			**24**			**24**	**100.0%**	**-0.85 [-1.10, -0.60]**

Heterogeneity: Tau² = 0.00; Chi² = 0.05, df = 1 (P = 0.83); I² = 0%

Test for overall effect: Z = 6.72 (P < 0.00001)

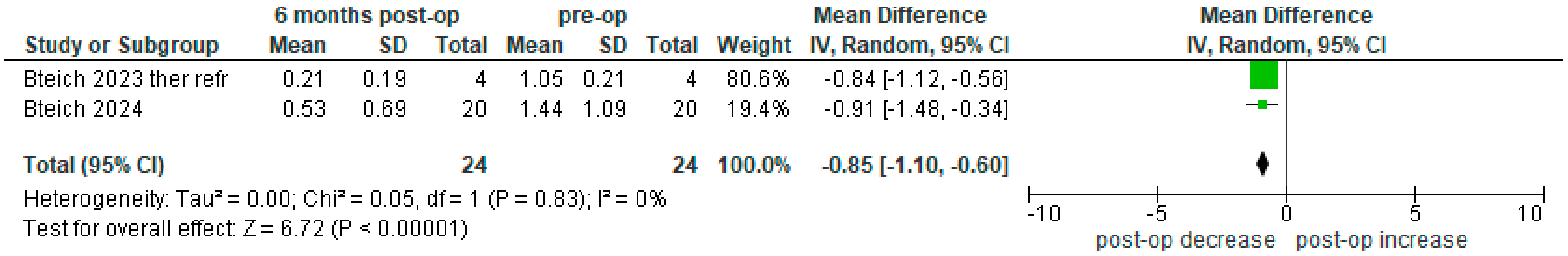

S1.3.2.10 Trefoil

S1.3.2.10A Trefoil across 0 studies – HYDRATED

Figure S1.3.2.10B Trefoil across 2 studies [2, 5] – DEHYDRATED

	6 months post-op			pre-op				Mean Difference
**Study or Subgroup**	**Mean**	**SD**	**Total**	**Mean**	**SD**	**Total**	**Weight**	**IV, Random, 95% CI**
Bteich 2024	0.1	0.6	20	-0.27	0.4	20	43.0%	0.37 [0.05, 0.69]
Kirgiz 2024	1.45	0.11	23	0.66	0.13	23	57.0%	0.79 [0.72, 0.86]
**Total (95% CI)**			**43**			**43**	**100.0%**	**0.61 [0.20, 1.02]**

Heterogeneity: Tau² = 0.07; Chi² = 6.47, df = 1 (P = 0.01); I² = 85%

Test for overall effect: Z = 2.93 (P = 0.003)

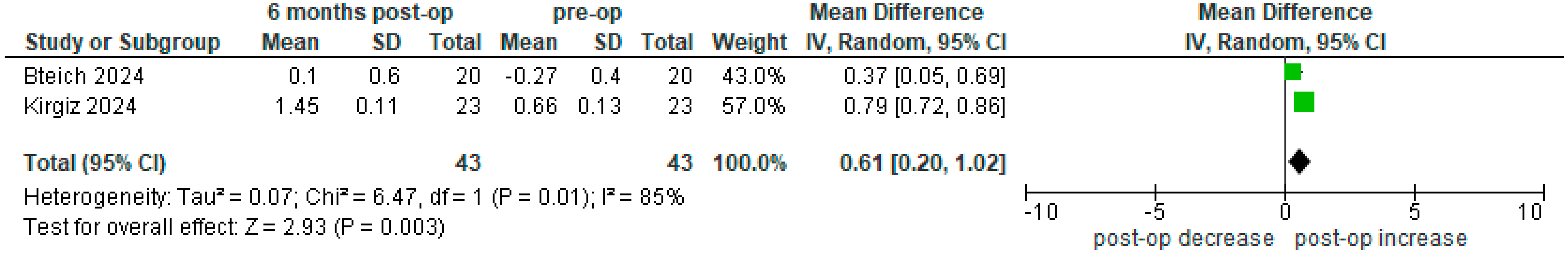

S1.3.2.11 Total RMS

S1.3.2.11A Total RMS across 0 studies – HYDRATED

Figure S1.3.2.11B Total RMS across 2 studies [5, 14] – DEHYDRATED

	6 months post-op			pre-op				Mean Difference
**Study or Subgroup**	**Mean**	**SD**	**Total**	**Mean**	**SD**	**Total**	**Weight**	**IV, Random, 95% CI**
Kirgiz 2024	2.44	0.15	23	2.35	0.3	23	56.1%	0.09 [-0.05, 0.23]
Mechleb 2025	10.76	6.24	33	14.37	6.33	79	43.9%	-3.61 [-6.16, -1.06]
**Total (95% CI)**			**56**			**102**	**100.0%**	**-1.53 [-5.13, 2.07]**

Heterogeneity: $Tau^2 = 6.00$; $Chi^2 = 8.09$, $df = 1$ ($P = 0.004$); $I^2 = 88\%$

Test for overall effect: $Z = 0.83$ ($P = 0.40$)

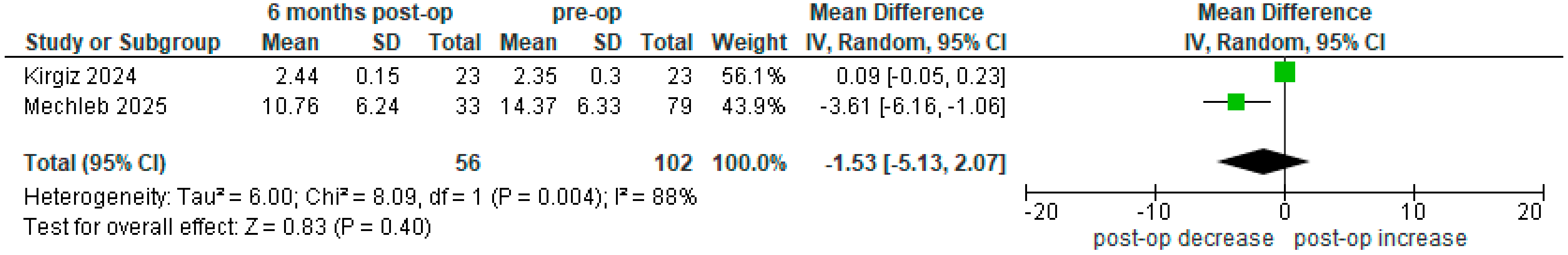

S1.3.3. 1 year postoperative versus preoperative

S1.3.3.1 Difference in uncorrected visual acuity

Figure S1.3.3.1A. Difference in uncorrected visual acuity across 2 studies [16, 17] – HYDRATED

	1 year post-op			pre-op				Mean Difference
**Study or Subgroup**	**Mean**	**SD**	**Total**	**Mean**	**SD**	**Total**	**Weight**	**IV, Random, 95% CI**
Asfar 2024	0.47	0.22	34	0.77	0.38	34	0.0%	-0.30 [-0.45, -0.15]
Bteich 2024	0.52	0.16	20	0.893	0.33	20	0.0%	-0.37 [-0.53, -0.21]
Jacob 2018	0.44	0.31	24	0.66	0.4	24	25.2%	-0.22 [-0.42, -0.02]
Jacob 2023	0.33	0.24	32	0.65	0.24	32	74.8%	-0.32 [-0.44, -0.20]
Keskin Perk 2025	0.44	0.35	50	0.96	0.5	62	0.0%	-0.52 [-0.68, -0.36]
Nacaroglu 2023	0.36	0.25	49	0.91	0.5	49	0.0%	-0.55 [-0.71, -0.39]
Yakut 2025	0.3	0.25	35	0.74	0.61	35	0.0%	-0.44 [-0.66, -0.22]
**Total (95% CI)**			**56**			**56**	**100.0%**	**-0.29 [-0.40, -0.19]**

Heterogeneity: Tau² = 0.00; Chi² = 0.70, df = 1 (P = 0.40); I² = 0%

Test for overall effect: Z = 5.68 (P < 0.00001)

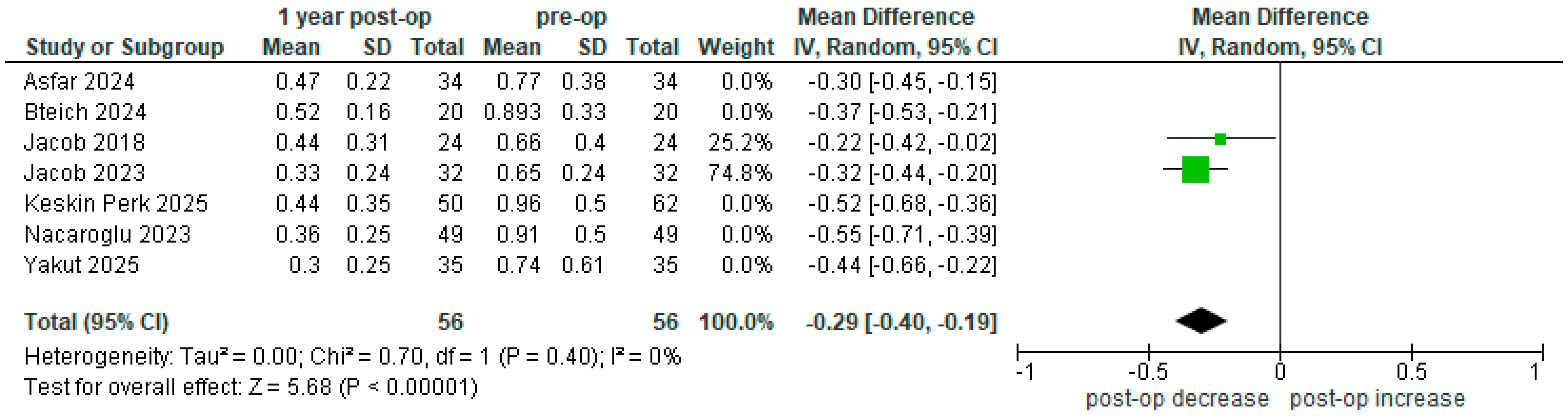

Figure S1.3.3.1B. Difference in uncorrected visual acuity across 2 studies [2, 15] – DEHYDRATED

	1 year post-op			pre-op				Mean Difference
**Study or Subgroup**	**Mean**	**SD**	**Total**	**Mean**	**SD**	**Total**	**Weight**	**IV, Random, 95% CI**
Asfar 2024	0.47	0.22	34	0.77	0.38	34	54.3%	-0.30 [-0.45, -0.15]
Bteich 2024	0.52	0.16	20	0.893	0.33	20	45.7%	-0.37 [-0.53, -0.21]
Jacob 2023	0.47	0.24	32	0.22	0.11	32	0.0%	0.25 [0.16, 0.34]
Keskin Perk 2025	0.44	0.35	50	0.96	0.5	62	0.0%	-0.52 [-0.68, -0.36]
Nacaroglu 2023	0.36	0.25	49	0.91	0.5	49	0.0%	-0.55 [-0.71, -0.39]
Yakut 2025	0.3	0.25	35	0.74	0.61	35	0.0%	-0.44 [-0.66, -0.22]
**Total (95% CI)**			**54**			**54**	**100.0%**	**-0.33 [-0.44, -0.22]**

Heterogeneity: $Tau^2 = 0.00$; $Chi^2 = 0.43$, df = 1 (P = 0.51); $I^2 = 0\%$
Test for overall effect: Z = 6.01 (P < 0.00001)

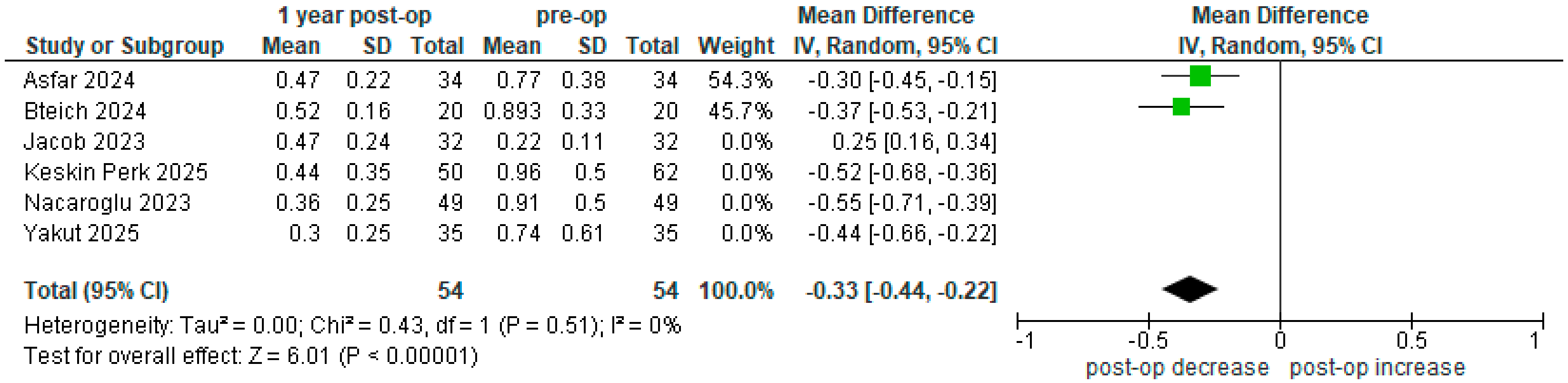

S1.3.3.2 Difference in corrected visual acuity

Figure S1.3.3.2A Difference in corrected visual acuity across 2 studies [16, 17] – HYDRATED

	1 year post-op			pre-op				Mean Difference
**Study or Subgroup**	**Mean**	**SD**	**Total**	**Mean**	**SD**	**Total**	**Weight**	**IV, Random, 95% CI**
Asfar 2024	0.16	0.18	34	0.52	0.23	34	0.0%	-0.36 [-0.46, -0.26]
Bteich 2024	0.15	0.18	20	0.57	0.18	20	0.0%	-0.42 [-0.53, -0.31]
Jacob 2018	0.1	0.11	24	0.23	0.16	24	35.0%	-0.13 [-0.21, -0.05]
Jacob 2023	0.05	0.06	32	0.12	0.11	32	65.0%	-0.07 [-0.11, -0.03]
Keskin Perk 2025	0.23	0.21	50	0.72	0.47	62	0.0%	-0.49 [-0.62, -0.36]
Nacaroglu 2023	0.36	0.15	49	0.87	0.2	49	0.0%	-0.51 [-0.58, -0.44]
Yakut 2025	0.15	0.15	35	0.54	0.32	35	0.0%	-0.39 [-0.51, -0.27]
**Total (95% CI)**			**56**			**56**	**100.0%**	**-0.09 [-0.15, -0.03]**

Heterogeneity: Tau² = 0.00; Chi² = 1.75, df = 1 (P = 0.19); I² = 43%
Test for overall effect: Z = 3.18 (P = 0.001)

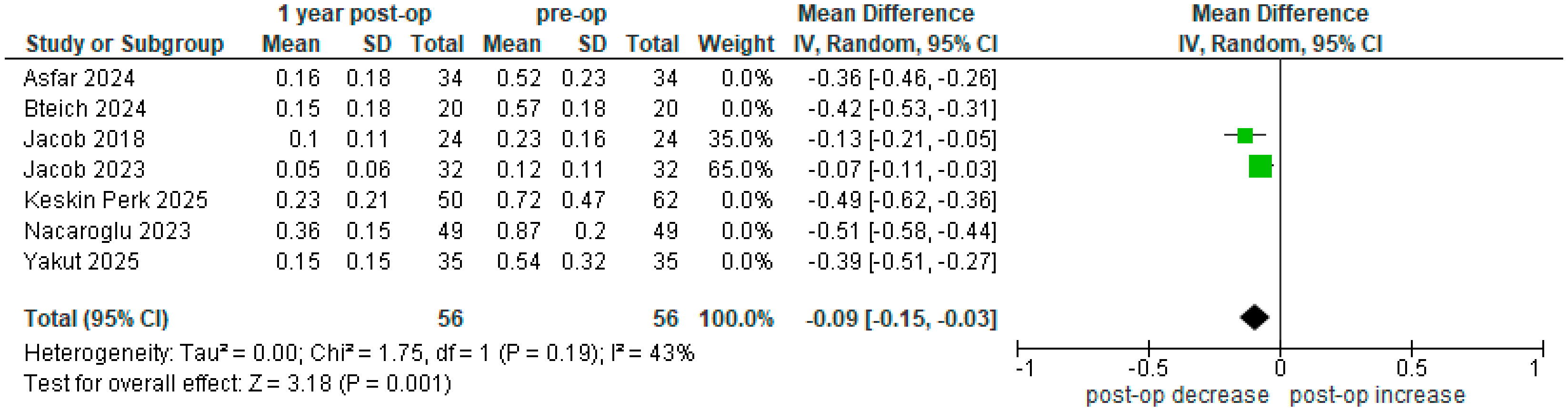

Figure S1.3.3.2B Difference in corrected visual acuity across 2 studies [2, 15]– DEHYDRATED

	1 year post-op			pre-op				Mean Difference
**Study or Subgroup**	**Mean**	**SD**	**Total**	**Mean**	**SD**	**Total**	**Weight**	**IV, Random, 95% CI**
Asfar 2024	0.16	0.18	34	0.52	0.23	34	56.4%	-0.36 [-0.46, -0.26]
Bteich 2024	0.15	0.18	20	0.57	0.18	20	43.6%	-0.42 [-0.53, -0.31]
Jacob 2023	0.89	0.17	32	0.76	0.19	32	0.0%	0.13 [0.04, 0.22]
Keskin Perk 2025	0.23	0.21	50	0.72	0.47	62	0.0%	-0.49 [-0.62, -0.36]
Nacaroglu 2023	0.36	0.15	49	0.87	0.2	49	0.0%	-0.51 [-0.58, -0.44]
Yakut 2025	0.15	0.15	35	0.54	0.32	35	0.0%	-0.39 [-0.51, -0.27]
**Total (95% CI)**			**54**			**54**	**100.0%**	**-0.39 [-0.46, -0.31]**

Heterogeneity: Tau² = 0.00; Chi² = 0.63, df = 1 (P = 0.43); I² = 0%
Test for overall effect: Z = 10.27 (P < 0.00001)

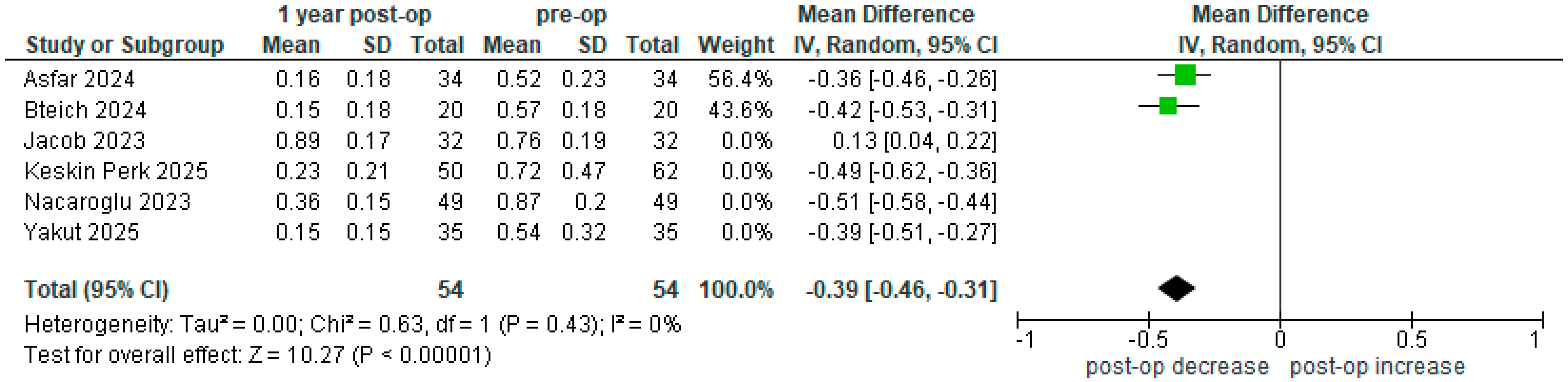

S1.3.3.3 Difference in pachymetry thinnest point

Figure S1.3.3.3A Difference in pachymetry thinnest point across 1 study [17] – HYDRATED

	1 year post-op			pre-op				Mean Difference
**Study or Subgroup**	**Mean**	**SD**	**Total**	**Mean**	**SD**	**Total**	**Weight**	**IV, Random, 95% CI**
Bteich 2024	442	51.8	20	442.8	38.1	20	0.0%	-0.80 [-28.98, 27.38]
Jacob 2023	455.94	45.74	32	449.88	35.99	32	100.0%	6.06 [-14.11, 26.23]
Keskin Perk 2025	444.38	50.15	50	449.68	49.48	62	0.0%	-5.30 [-23.87, 13.27]
**Total (95% CI)**			**32**			**32**	**100.0%**	**6.06 [-14.11, 26.23]**
Heterogeneity: Not applicable								
Test for overall effect: Z = 0.59 (P = 0.56)								

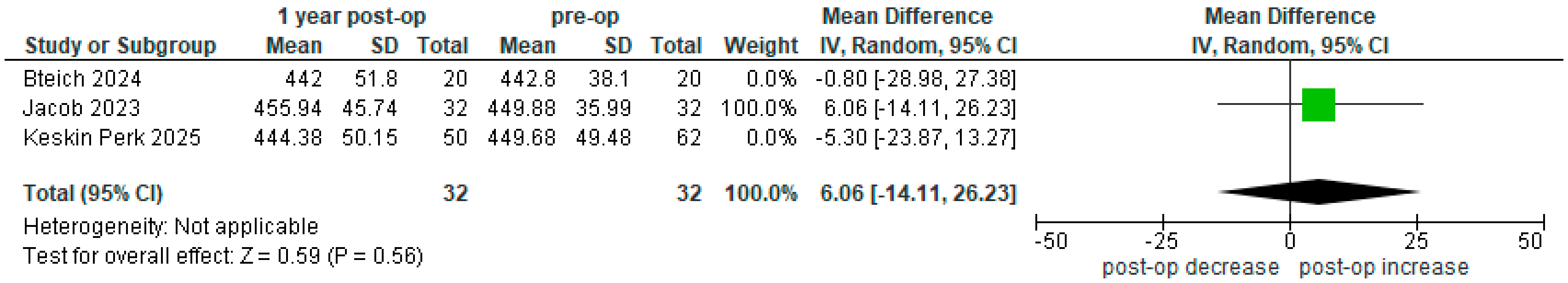

Figure S1.3.3.3B Difference in pachymetry thinnest point across 1 study [2] – DEHYDRATED

	1 year post-op			pre-op				Mean Difference
**Study or Subgroup**	**Mean**	**SD**	**Total**	**Mean**	**SD**	**Total**	**Weight**	**IV, Random, 95% CI**
Bteich 2024	442	51.8	20	442.8	38.1	20	100.0%	-0.80 [-28.98, 27.38]
Jacob 2023	455.94	45.74	32	449.88	35.99	32	0.0%	6.06 [-14.11, 26.23]
Keskin Perk 2025	444.38	50.15	50	449.68	49.48	62	0.0%	-5.30 [-23.87, 13.27]
**Total (95% CI)**			**20**			**20**	**100.0%**	**-0.80 [-28.98, 27.38]**

Heterogeneity: Not applicable
Test for overall effect: Z = 0.06 (P = 0.96)

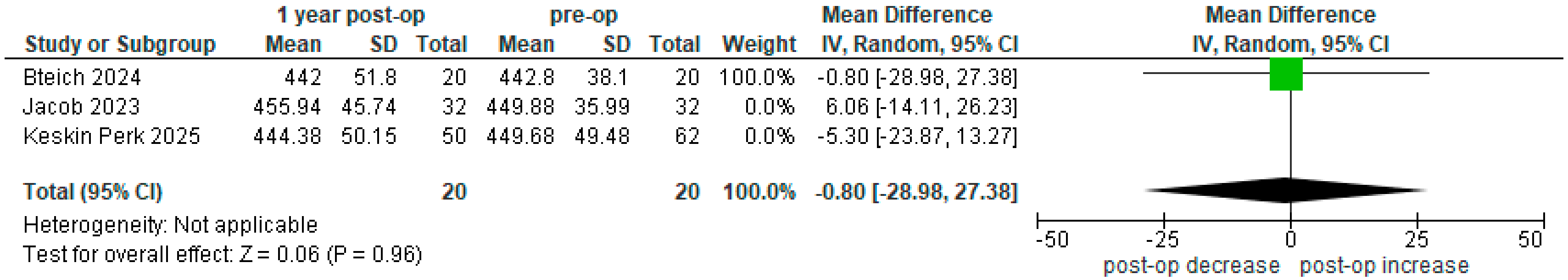

S1.3.3.4 Difference in pachymetry central point

Figure S1.3.3.4A Difference in pachymetry central point across 0 studies – HYDRATED

Figure S1.3.3.4B Difference in pachymetry central point across 0 studies – DEHYDRATED

S1.3.3.5 Difference in maximum keratometry

Figure S1.3.3.5A Difference in maximum keratometry in 1 study [17] – HYDRATED

Study or Subgroup	1 year post-op Mean	1 year post-op SD	1 year post-op Total	pre-op Mean	pre-op SD	pre-op Total	Weight	Mean Difference IV, Random, 95% CI
Asfar 2024	50.69	6.38	34	55.85	7.53	34	0.0%	-5.16 [-8.48, -1.84]
Bteich 2024	52.85	5.44	20	57.21	7.53	20	0.0%	-4.36 [-8.43, -0.29]
Jacob 2023	54.95	4.71	32	58.9	5.54	32	100.0%	-3.95 [-6.47, -1.43]
Keskin Perk 2025	55.17	5.87	50	58.51	7.55	62	0.0%	-3.34 [-5.83, -0.85]
Nacaroglu 2023	55.63	6.14	49	59.2	7.66	49	0.0%	-3.57 [-6.32, -0.82]
Yakut 2025	54.29	5.61	35	57	6.81	35	0.0%	-2.71 [-5.63, 0.21]
**Total (95% CI)**			**32**			**32**	**100.0%**	**-3.95 [-6.47, -1.43]**

Heterogeneity: Not applicable
Test for overall effect: Z = 3.07 (P = 0.002)

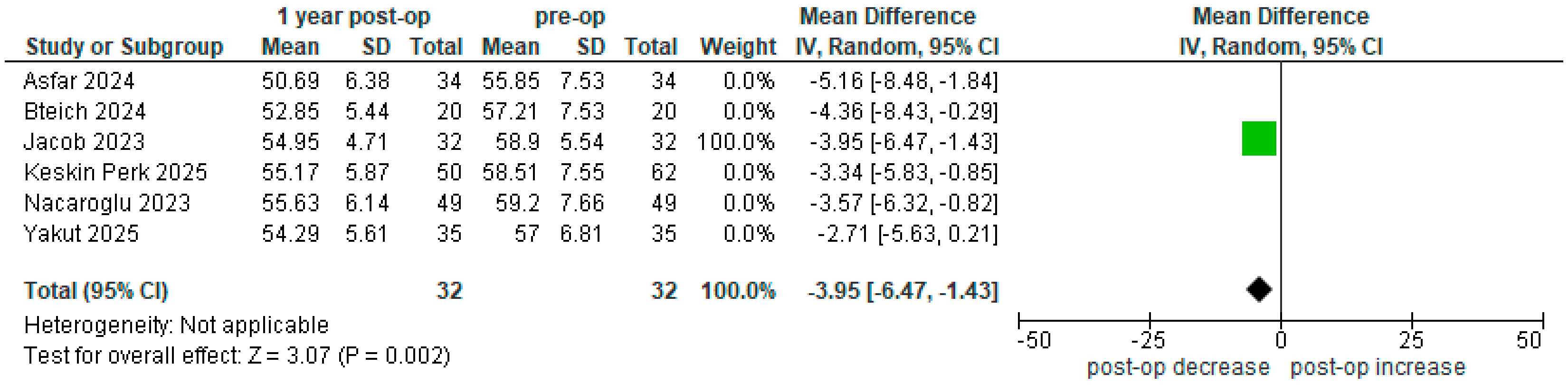

Figure S1.3.3.5B Difference in maximum keratometry across 2 studies [2, 15] – DEHYDRATED

	1 year post-op			pre-op				Mean Difference
**Study or Subgroup**	**Mean**	**SD**	**Total**	**Mean**	**SD**	**Total**	**Weight**	**IV, Random, 95% CI**
Asfar 2024	50.69	6.38	34	55.85	7.53	34	60.1%	-5.16 [-8.48, -1.84]
Bteich 2024	52.85	5.44	20	57.21	7.53	20	39.9%	-4.36 [-8.43, -0.29]
Jacob 2023	54.95	4.71	32	58.9	5.54	32	0.0%	-3.95 [-6.47, -1.43]
Keskin Perk 2025	55.17	5.87	50	58.51	7.55	62	0.0%	-3.34 [-5.83, -0.85]
Nacaroglu 2023	55.63	6.14	49	59.2	7.66	49	0.0%	-3.57 [-6.32, -0.82]
Yakut 2025	54.29	5.61	35	57	6.81	35	0.0%	-2.71 [-5.63, 0.21]
**Total (95% CI)**			**54**			**54**	**100.0%**	**-4.84 [-7.41, -2.27]**

Heterogeneity: Tau² = 0.00; Chi² = 0.09, df = 1 (P = 0.77); I² = 0%
Test for overall effect: Z = 3.69 (P = 0.0002)

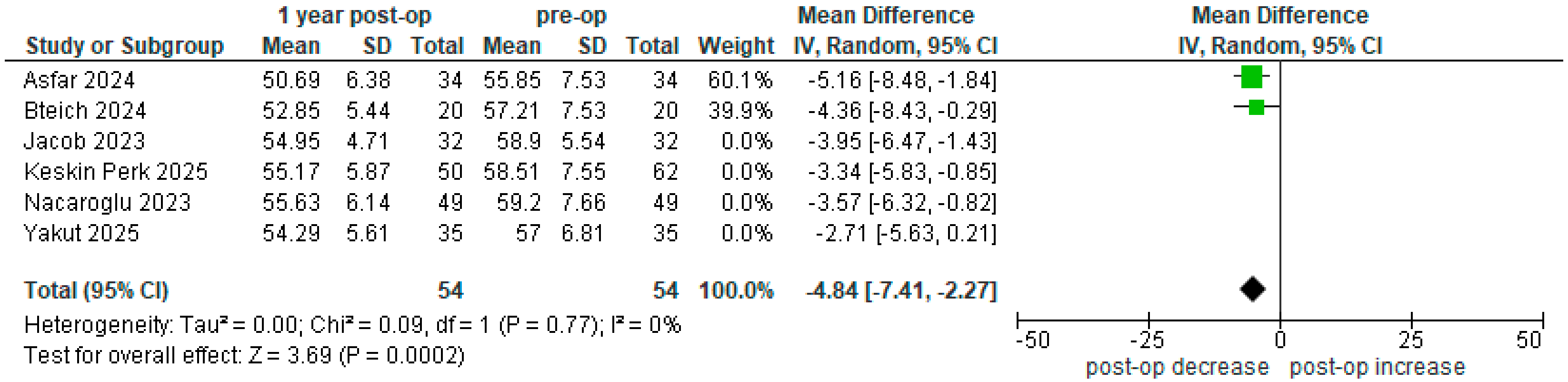

S1.3.3.6 Difference in mean simulated keratometry

Figure S1.3.3.6A Difference in mean simulated keratometry in 1 study [17] – HYDRATED

	1 year post-op			pre-op				Mean Difference
**Study or Subgroup**	**Mean**	**SD**	**Total**	**Mean**	**SD**	**Total**	**Weight**	**IV, Random, 95% CI**
Asfar 2024	44.25	3.29	34	47.52	3.82	34	0.0%	-3.27 [-4.96, -1.58]
Bteich 2024	45.23	2.83	20	49.3	4.83	20	0.0%	-4.07 [-6.52, -1.62]
Jacob 2023	45.63	2.91	32	49.07	3.06	32	100.0%	-3.44 [-4.90, -1.98]
Keskin Perk 2025	45.63	4.44	50	49.17	4.97	62	0.0%	-3.54 [-5.28, -1.80]
Nacaroglu 2023	45.39	4.92	49	49.34	5.32	49	0.0%	-3.95 [-5.98, -1.92]
Yakut 2025	44.5	4.42	35	48.26	4.78	35	0.0%	-3.76 [-5.92, -1.60]
**Total (95% CI)**			**32**			**32**	**100.0%**	**-3.44 [-4.90, -1.98]**

Heterogeneity: Not applicable
Test for overall effect: Z = 4.61 (P < 0.00001)

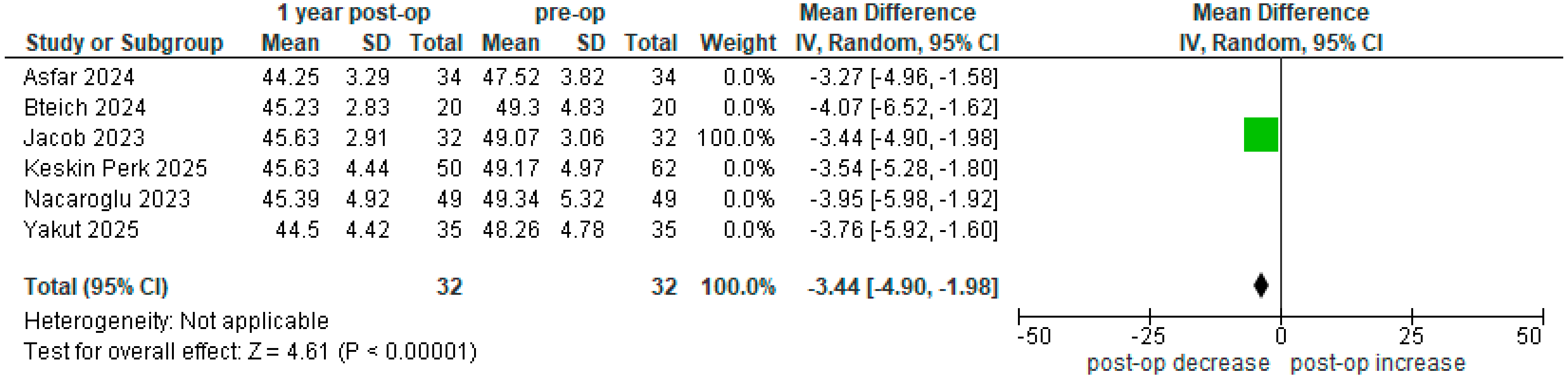

Figure S1.3.3.6B Difference in mean simulated keratometry across 2 studies [2, 15] – DEHYDRATED

	1 year post-op			pre-op				Mean Difference
**Study or Subgroup**	**Mean**	**SD**	**Total**	**Mean**	**SD**	**Total**	**Weight**	**IV, Random, 95% CI**
Asfar 2024	44.25	3.29	34	47.52	3.82	34	67.7%	-3.27 [-4.96, -1.58]
Bteich 2024	45.23	2.83	20	49.3	4.83	20	32.3%	-4.07 [-6.52, -1.62]
Jacob 2023	45.63	2.91	32	49.07	3.06	32	0.0%	-3.44 [-4.90, -1.98]
Keskin Perk 2025	45.63	4.44	50	49.17	4.97	62	0.0%	-3.54 [-5.28, -1.80]
Nacaroglu 2023	45.39	4.92	49	49.34	5.32	49	0.0%	-3.95 [-5.98, -1.92]
Yakut 2025	44.5	4.42	35	48.26	4.78	35	0.0%	-3.76 [-5.92, -1.60]
**Total (95% CI)**			**54**			**54**	**100.0%**	**-3.53 [-4.92, -2.13]**

Heterogeneity: $Tau^2 = 0.00$; $Chi^2 = 0.28$, df = 1 (P = 0.60); $I^2 = 0\%$
Test for overall effect: Z = 4.96 (P < 0.00001)

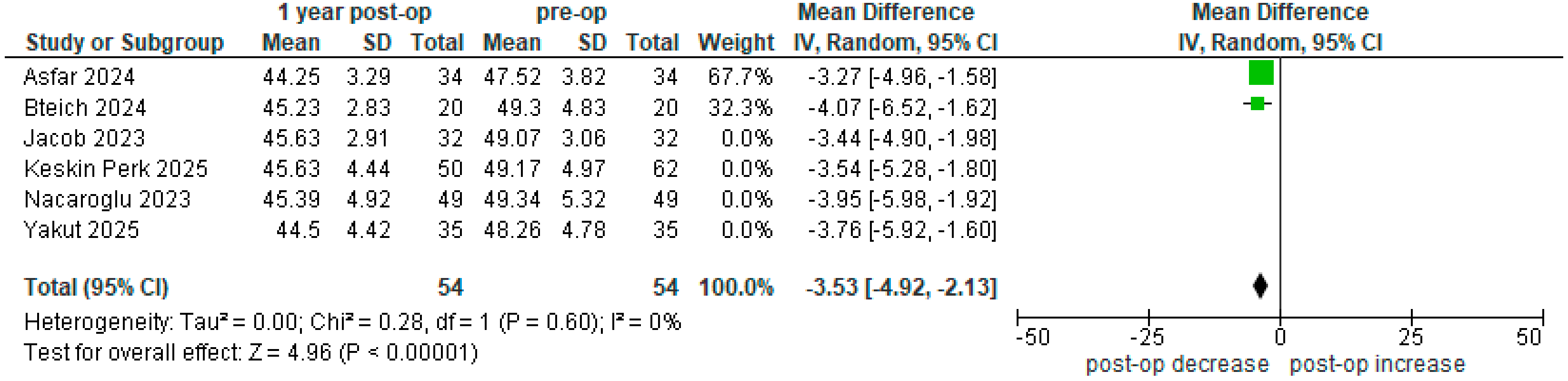

S1.3.3.7 Difference in total higher order aberrations

Figure S1.3.3.7A Difference in total higher order aberrations across 0 studies – HYDRATED

Figure S1.3.3.7B Difference in total higher order aberrations across 2 studies [2, 15]– DEHYDRATED

	1 year post-op			pre-op				Mean Difference
Study or Subgroup	Mean	SD	Total	Mean	SD	Total	Weight	IV, Random, 95% CI
Asfar 2024	1.54	0.93	34	2.07	1.05	34	53.4%	-0.53 [-1.00, -0.06]
Bteich 2024	1.36	0.65	20	1.92	0.95	20	46.6%	-0.56 [-1.06, -0.06]
**Total (95% CI)**			**54**			**54**	**100.0%**	**-0.54 [-0.89, -0.20]**

Heterogeneity: Tau² = 0.00; Chi² = 0.01, df = 1 (P = 0.93); I² = 0%
Test for overall effect: Z = 3.10 (P = 0.002)

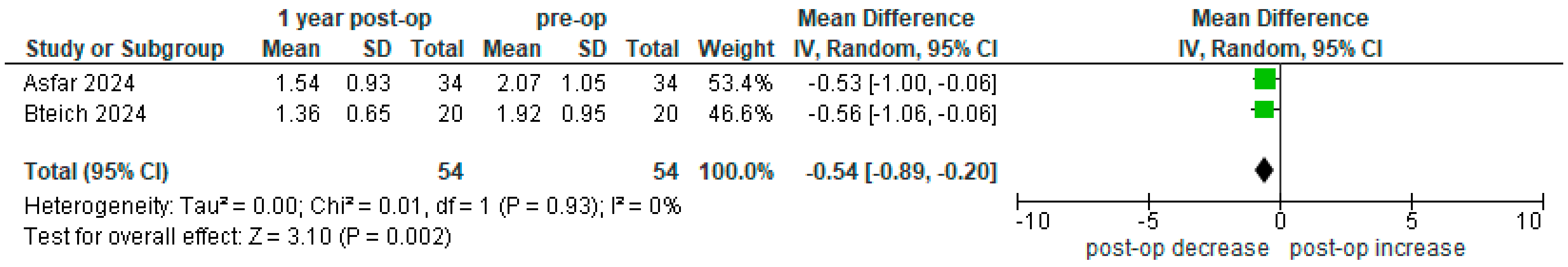

S1.3.3.8 Difference in spherical aberration

S1.3.3.8A Difference in spherical aberration across 0 studies – HYDRATED

Figure S1.3.3.8B Difference in spherical aberration across 2 studies [2, 15] – DEHYDRATED

	1 year post-op			pre-op				Mean Difference
**Study or Subgroup**	**Mean**	**SD**	**Total**	**Mean**	**SD**	**Total**	**Weight**	**IV, Random, 95% CI**
Asfar 2024	0.18	0.5	34	0.07	0.42	34	60.7%	0.11 [-0.11, 0.33]
Bteich 2024	0.15	0.46	20	0.05	0.42	20	39.3%	0.10 [-0.17, 0.37]
**Total (95% CI)**			**54**			**54**	**100.0%**	**0.11 [-0.06, 0.28]**

Heterogeneity: Tau² = 0.00; Chi² = 0.00, df = 1 (P = 0.96); I² = 0%
Test for overall effect: Z = 1.22 (P = 0.22)

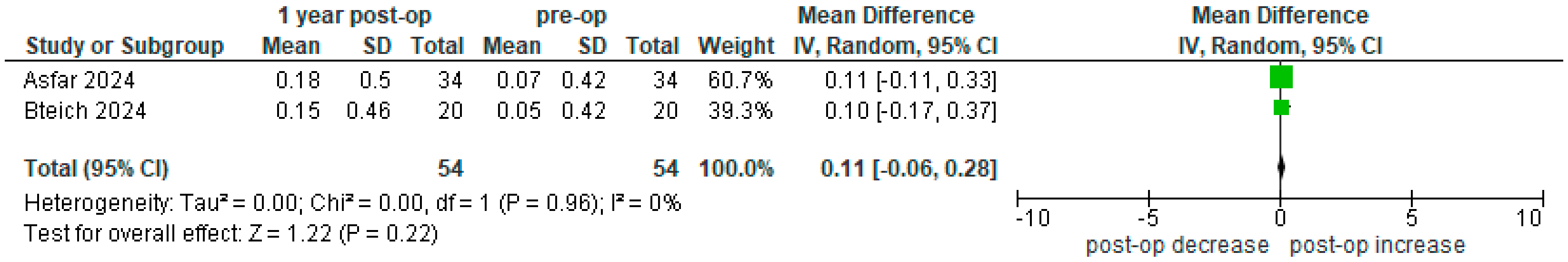

S1.3.3.9 Difference in vertical coma

Figure S1.3.3.9A Difference in vertical coma in 1 study [17] – HYDRATED

	1 year post-op			pre-op				Mean Difference
**Study or Subgroup**	**Mean**	**SD**	**Total**	**Mean**	**SD**	**Total**	**Weight**	**IV, Random, 95% CI**
Asfar 2024	0.38	0.65	34	1.49	1.02	34	0.0%	-1.11 [-1.52, -0.70]
Bteich 2024	0.35	0.97	20	1.44	1.09	20	0.0%	-1.09 [-1.73, -0.45]
Jacob 2023	-1.4	1.21	32	-2.09	0.96	32	100.0%	0.69 [0.15, 1.23]
**Total (95% CI)**			**32**			**32**	**100.0%**	**0.69 [0.15, 1.23]**
Heterogeneity: Not applicable								
Test for overall effect: Z = 2.53 (P = 0.01)								

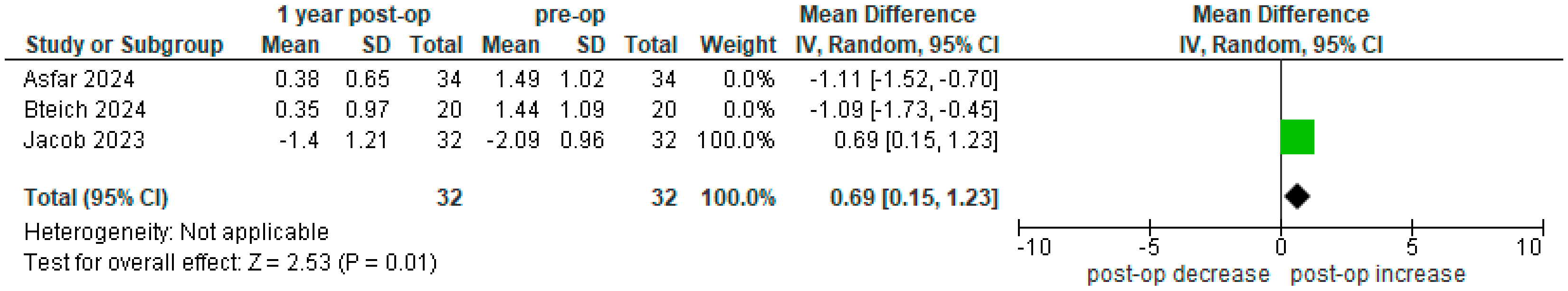

Figure S1.3.3.9B Difference in vertical coma across 2 studies [2, 15]– DEHYDRATED

	1 year post-op			pre-op				Mean Difference
**Study or Subgroup**	**Mean**	**SD**	**Total**	**Mean**	**SD**	**Total**	**Weight**	**IV, Random, 95% CI**
Asfar 2024	0.38	0.65	34	1.49	1.02	34	71.2%	-1.11 [-1.52, -0.70]
Bteich 2024	0.35	0.97	20	1.44	1.09	20	28.8%	-1.09 [-1.73, -0.45]
Jacob 2023	-1.4	1.21	32	-2.09	0.96	32	0.0%	0.69 [0.15, 1.23]
**Total (95% CI)**			**54**			**54**	**100.0%**	**-1.10 [-1.45, -0.76]**

Heterogeneity: Tau² = 0.00; Chi² = 0.00, df = 1 (P = 0.96); I² = 0%
Test for overall effect: Z = 6.31 (P < 0.00001)

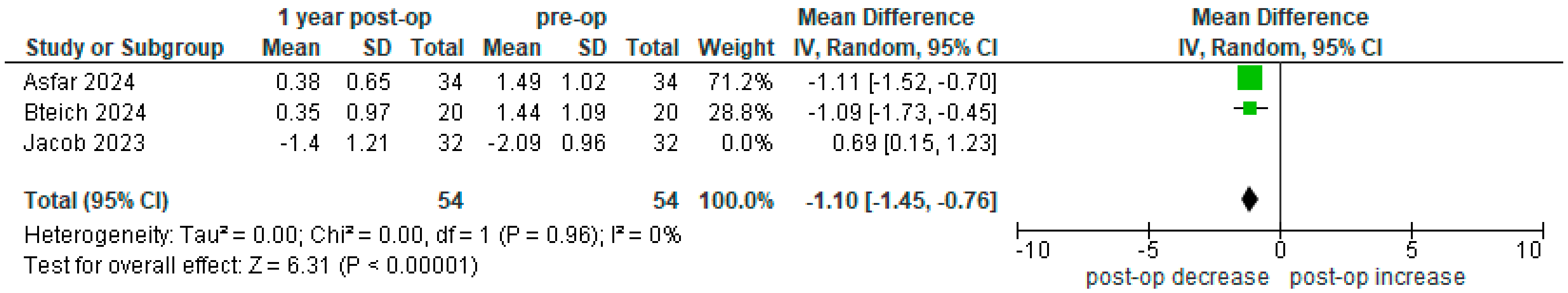

S1.3.3.10 Difference in trefoil

Figure S1.3.3.10A Difference in trefoil across 0 studies – HYDRATED

Figure S1.3.3.10B Difference in trefoil across 2 studies [2, 15]– DEHYDRATED

	1 year post-op			pre-op				Mean Difference
**Study or Subgroup**	**Mean**	**SD**	**Total**	**Mean**	**SD**	**Total**	**Weight**	**IV, Random, 95% CI**
Asfar 2024	-0.17	0.63	34	-0.37	0.59	34	53.7%	0.20 [-0.09, 0.49]
Bteich 2024	0.007	0.59	20	-0.27	0.4	20	46.3%	0.28 [-0.04, 0.59]
**Total (95% CI)**			**54**			**54**	**100.0%**	**0.24 [0.02, 0.45]**

Heterogeneity: Tau² = 0.00; Chi² = 0.13, df = 1 (P = 0.72); I² = 0%
Test for overall effect: Z = 2.17 (P = 0.03)

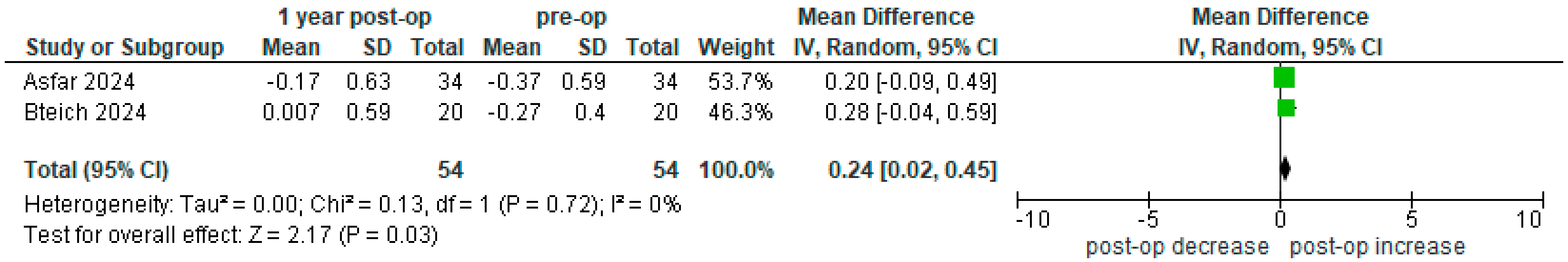

S1.3.3.11 Difference in total RMS

Figure S1.3.3.11A Difference in total RMS across 1 study [17] – HYDRATED

	1 year post-op			pre-op				Mean Difference	Mean Difference
**Study or Subgroup**	**Mean**	**SD**	**Total**	**Mean**	**SD**	**Total**	**Weight**	**IV, Random, 95% CI**	**IV, Random, 95% CI**
Jacob 2023	2.59	0.8	32	2.85	0.78	32	100.0%	-0.26 [-0.65, 0.13]	
**Total (95% CI)**			**32**			**32**	**100.0%**	**-0.26 [-0.65, 0.13]**	

Heterogeneity: Not applicable
Test for overall effect: Z = 1.32 (P = 0.19)

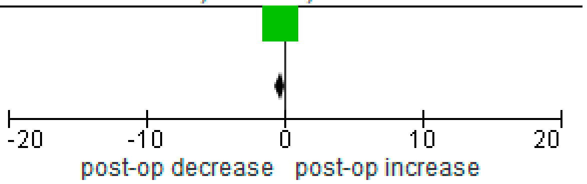

Figure S1.3.3.11B Difference in total RMS across 0 studies – DEHYDRATED

SECTION S1.4 – SUBGROUP ANALYSIS CAIRS PREPARATION – CXL VS NO CXL

S1.4.1. 1 month postoperative versus preoperative

S1.4.1.1. Difference in uncorrected visual acuity

Figure S1.4.1.1A. Difference in uncorrected visual acuity across 2 studies [6, 7] – CXL

	1 month post-op			pre-op				Mean Difference
Study or Subgroup	Mean	SD	Total	Mean	SD	Total	Weight	IV, Random, 95% CI
Bteich 2023	0.64	0.23	52	0.97	0.47	52	0.0%	-0.33 [-0.47, -0.19]
Bteich 2024	0.66	0.33	20	0.893	0.33	20	0.0%	-0.23 [-0.44, -0.03]
Haciagaoglu 2022	0.34	0.26	44	0.72	0.63	44	0.0%	-0.38 [-0.58, -0.18]
Keskin Perk 2025	0.4	0.34	62	0.96	0.5	62	0.0%	-0.56 [-0.71, -0.41]
Kirgiz 2024	0.432	0.03	23	1.096	0.05	23	0.0%	-0.66 [-0.69, -0.64]
Nacaroglu 2023	0.39	0.28	49	0.91	0.5	49	57.7%	-0.52 [-0.68, -0.36]
Yukecul 2024 (non-CXL)	0.26	0.22	37	0.77	0.76	37	0.0%	-0.51 [-0.76, -0.26]
Yukecul 2024 (prior CXL)	0.38	0.29	30	0.7	0.55	30	42.3%	-0.32 [-0.54, -0.10]
**Total (95% CI)**			**79**			**79**	**100.0%**	**-0.44 [-0.63, -0.24]**

Heterogeneity: Tau² = 0.01; Chi² = 2.04, df = 1 (P = 0.15); I² = 51%
Test for overall effect: Z = 4.41 (P < 0.0001)

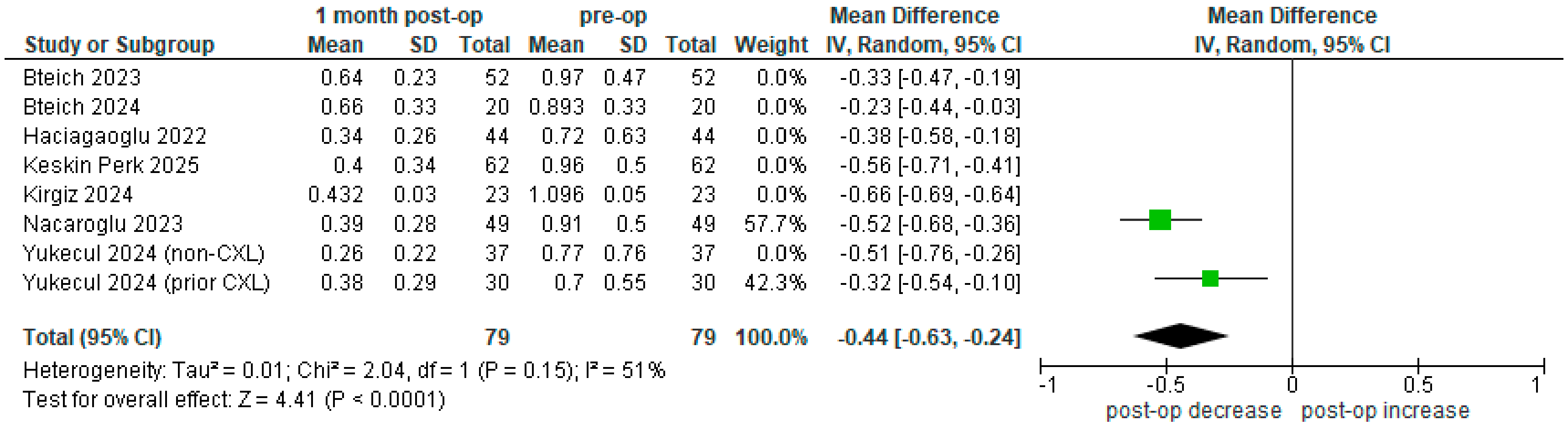

Figure S1.4.1.1B. Difference in uncorrected visual acuity across 6 studies [1-5, 7] – no CXL

	1 month post-op			pre-op				Mean Difference
**Study or Subgroup**	**Mean**	**SD**	**Total**	**Mean**	**SD**	**Total**	**Weight**	**IV, Random, 95% CI**
Bteich 2023	0.64	0.23	52	0.97	0.47	52	17.6%	-0.33 [-0.47, -0.19]
Bteich 2024	0.66	0.33	20	0.893	0.33	20	15.5%	-0.23 [-0.44, -0.03]
Haciagaoglu 2022	0.34	0.26	44	0.72	0.63	44	15.6%	-0.38 [-0.58, -0.18]
Keskin Perk 2025	0.4	0.34	62	0.96	0.5	62	17.4%	-0.56 [-0.71, -0.41]
Kirgiz 2024	0.432	0.03	23	1.096	0.05	23	20.2%	-0.66 [-0.69, -0.64]
Nacaroglu 2023	0.39	0.28	49	0.91	0.5	49	0.0%	-0.52 [-0.68, -0.36]
Yukecul 2024 (non-CXL)	0.26	0.22	37	0.77	0.76	37	13.7%	-0.51 [-0.76, -0.26]
Yukecul 2024 (prior CXL)	0.38	0.29	30	0.7	0.55	30	0.0%	-0.32 [-0.54, -0.10]
**Total (95% CI)**			**238**			**238**	**100.0%**	**-0.45 [-0.62, -0.29]**

Heterogeneity: Tau² = 0.04; Chi² = 45.79, df = 5 (P < 0.00001); I² = 89%
Test for overall effect: Z = 5.36 (P < 0.00001)

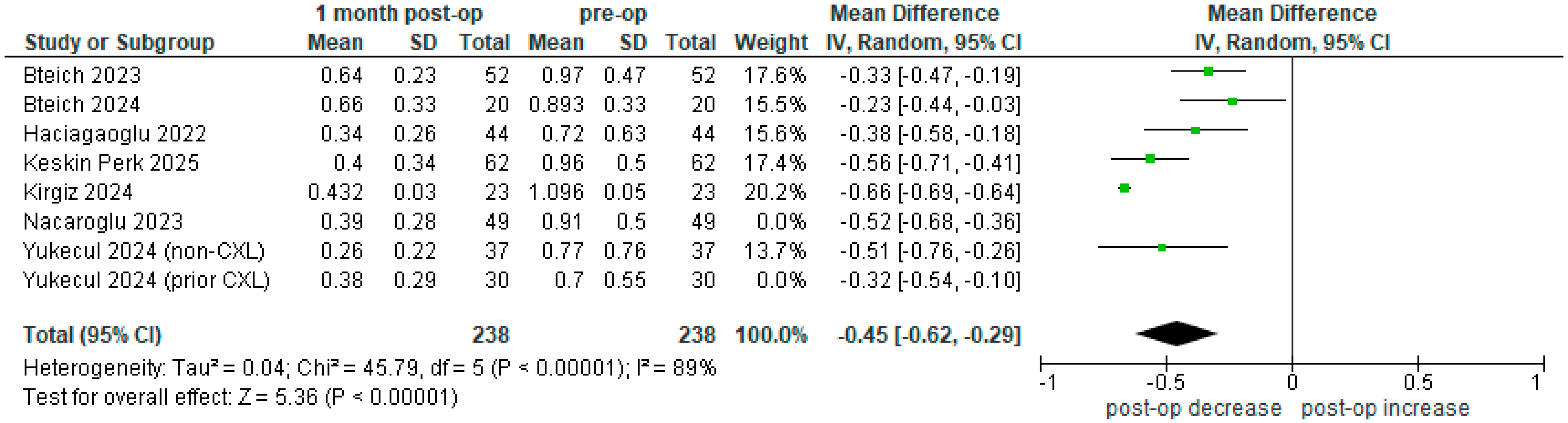

S1.4.1.2. Difference in best corrected visual acuity

Figure S1.4.1.2A. Difference in best corrected visual acuity across 2 studies [6, 7] – CXL

	1 month post-op			pre-op				Mean Difference
**Study or Subgroup**	**Mean**	**SD**	**Total**	**Mean**	**SD**	**Total**	**Weight**	**IV, Random, 95% CI**
Bteich 2023	0.31	0.21	52	0.56	0.19	52	0.0%	-0.25 [-0.33, -0.17]
Bteich 2024	0.32	0.2	20	0.57	0.18	20	0.0%	-0.25 [-0.37, -0.13]
Haciagaoglu 2022	0.24	0.19	44	0.54	0.37	44	0.0%	-0.30 [-0.42, -0.18]
Keskin Perk 2025	0.28	0.22	62	0.72	0.47	62	0.0%	-0.44 [-0.57, -0.31]
Kirgiz 2024	0.237	0.06	23	0.602	0.3	23	0.0%	-0.36 [-0.49, -0.24]
Mechleb 2024 (Kmax inf 75D)	0.03	0.02	6	0.36	0.12	6	0.0%	-0.33 [-0.43, -0.23]
Mechleb 2024 (Kmax sup 75D)	1.03	0.88	4	2.45	1.1	4	0.0%	-1.42 [-2.80, -0.04]
Nacaroglu 2023	0.27	0.06	49	0.87	0.2	49	52.7%	-0.60 [-0.66, -0.54]
Yukecul 2024 (non-CXL)	0.19	0.15	37	0.51	0.29	37	0.0%	-0.32 [-0.43, -0.21]
Yukecul 2024 (prior CXL)	0.27	0.2	30	0.55	0.39	30	47.3%	-0.28 [-0.44, -0.12]
**Total (95% CI)**			**79**			**79**	**100.0%**	**-0.45 [-0.76, -0.14]**

Heterogeneity: $Tau^2 = 0.05$; $Chi^2 = 14.04$, df = 1 (P = 0.0002); $I^2 = 93\%$
Test for overall effect: Z = 2.81 (P = 0.005)

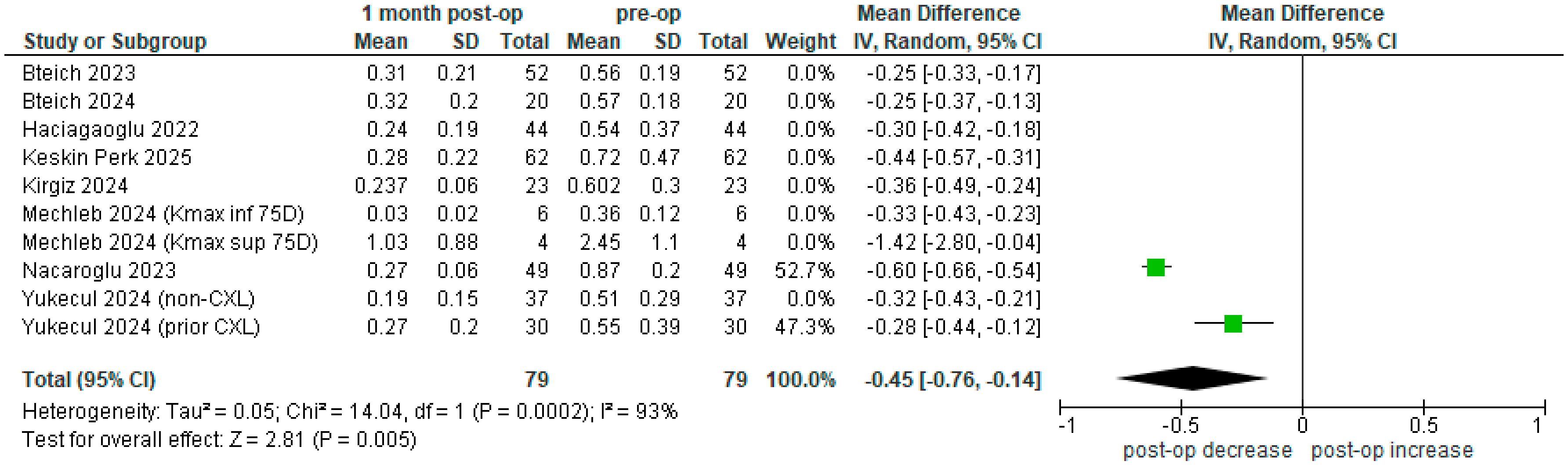

Figure S1.4.1.2B. Difference in best corrected visual acuity across 8 studies [1-5, 7, 8] – no CXL

	1 month post-op			pre-op				Mean Difference
**Study or Subgroup**	**Mean**	**SD**	**Total**	**Mean**	**SD**	**Total**	**Weight**	**IV, Random, 95% CI**
Bteich 2023	0.31	0.21	52	0.56	0.19	52	20.7%	-0.25 [-0.33, -0.17]
Bteich 2024	0.32	0.2	20	0.57	0.18	20	12.8%	-0.25 [-0.37, -0.13]
Haciagaoglu 2022	0.24	0.19	44	0.54	0.37	44	12.1%	-0.30 [-0.42, -0.18]
Keskin Perk 2025	0.28	0.22	62	0.72	0.47	62	11.3%	-0.44 [-0.57, -0.31]
Kirgiz 2024	0.237	0.06	23	0.602	0.3	23	11.8%	-0.36 [-0.49, -0.24]
Mechleb 2024 (Kmax inf 75D)	0.03	0.02	6	0.36	0.12	6	16.3%	-0.33 [-0.43, -0.23]
Mechleb 2024 (Kmax sup 75D)	1.03	0.88	4	2.45	1.1	4	0.1%	-1.42 [-2.80, -0.04]
Nacaroglu 2023	0.27	0.06	49	0.87	0.2	49	0.0%	-0.60 [-0.66, -0.54]
Yukecul 2024 (non-CXL)	0.19	0.15	37	0.51	0.29	37	14.8%	-0.32 [-0.43, -0.21]
Yukecul 2024 (prior CXL)	0.27	0.2	30	0.55	0.39	30	0.0%	-0.28 [-0.44, -0.12]
**Total (95% CI)**			**248**			**248**	**100.0%**	**-0.32 [-0.37, -0.26]**

Heterogeneity: Tau² = 0.00; Chi² = 10.67, df = 7 (P = 0.15); I² = 34%
Test for overall effect: Z = 11.97 (P < 0.00001)

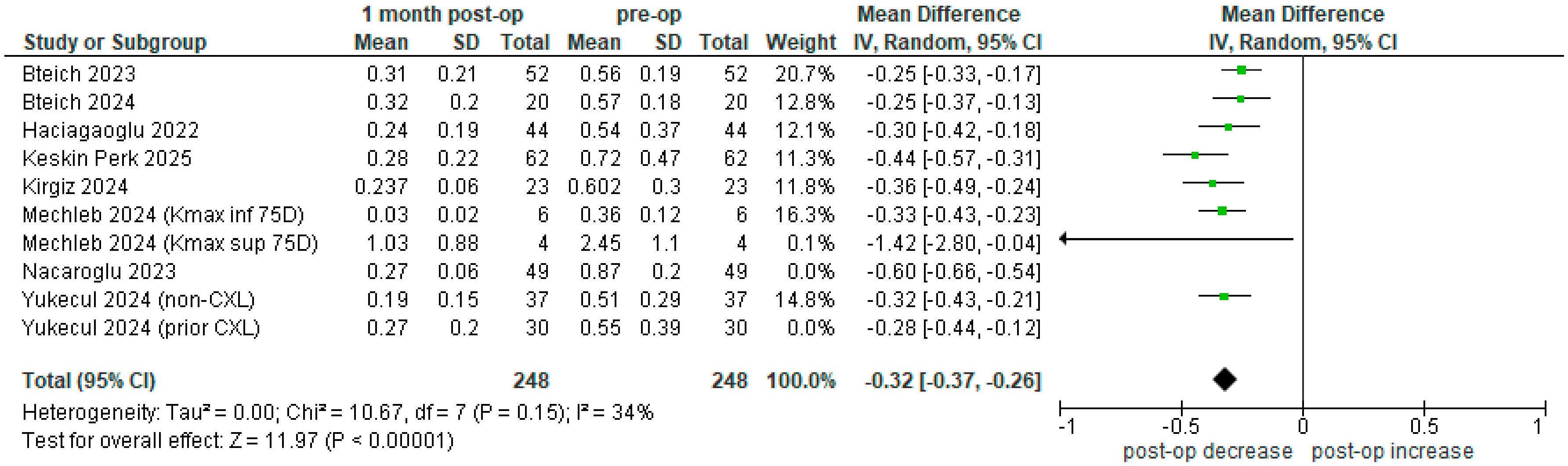

S1.4.1.3. Difference in pachymetry thinnest point

Figure S1.4.1.3A. Difference in pachymetry thinnest point in 1 study [7] – CXL

	1 month post-op			pre-op				Mean Difference
**Study or Subgroup**	**Mean**	**SD**	**Total**	**Mean**	**SD**	**Total**	**Weight**	**IV, Random, 95% CI**
Bteich 2023	429.7	55.5	52	422.5	55.2	52	0.0%	7.20 [-14.08, 28.48]
Bteich 2024	443.8	41.2	20	442.8	38.1	20	0.0%	1.00 [-23.59, 25.59]
Haciagaoglu 2022	449.2	57.41	44	453.73	45.87	44	0.0%	-4.53 [-26.24, 17.18]
Keskin Perk 2025	446.32	40.26	62	449.68	49.48	62	0.0%	-3.36 [-19.24, 12.52]
Kirgiz 2024	431.33	11.78	23	439.33	10.43	23	0.0%	-8.00 [-14.43, -1.57]
Yukecul 2024 (non-CXL)	452	36	37	454	45	37	0.0%	-2.00 [-20.57, 16.57]
Yukecul 2024 (prior CXL)	449	42	30	437	38	30	100.0%	12.00 [-8.27, 32.27]
**Total (95% CI)**			**30**			**30**	**100.0%**	**12.00 [-8.27, 32.27]**
Heterogeneity: Not applicable								
Test for overall effect: Z = 1.16 (P = 0.25)								

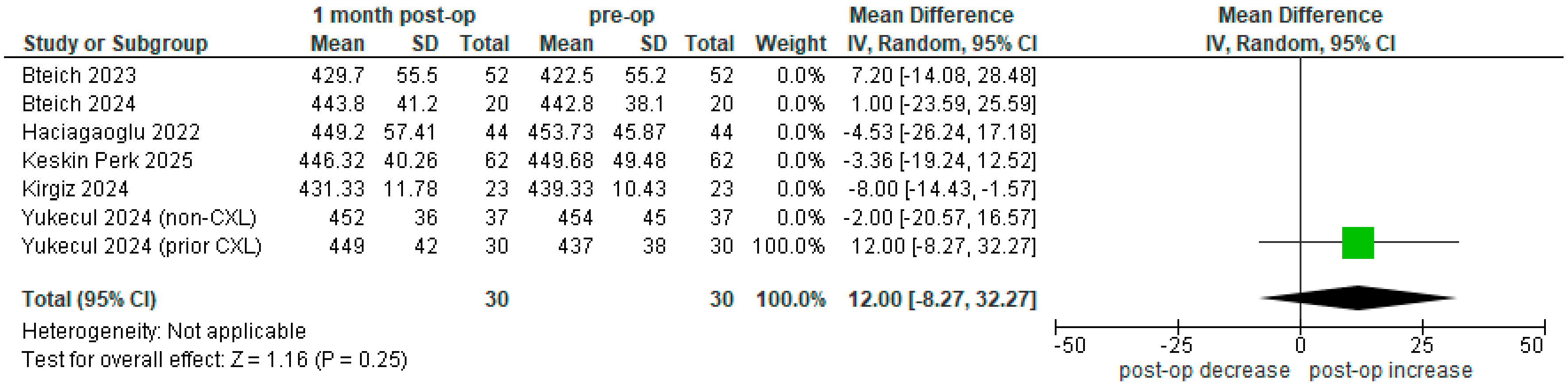

Figure S1.4.1.3B. Difference in pachymetry thinnest point across 6 studies [1-5, 7] – no CXL

Study or Subgroup	1 month post-op Mean	SD	Total	pre-op Mean	SD	Total	Weight	Mean Difference IV, Random, 95% CI
Bteich 2023	429.7	55.5	52	422.5	55.2	52	6.0%	7.20 [-14.08, 28.48]
Bteich 2024	443.8	41.2	20	442.8	38.1	20	4.5%	1.00 [-23.59, 25.59]
Haciagaoglu 2022	449.2	57.41	44	453.73	45.87	44	5.7%	-4.53 [-26.24, 17.18]
Keskin Perk 2025	446.32	40.26	62	449.68	49.48	62	10.7%	-3.36 [-19.24, 12.52]
Kirgiz 2024	431.33	11.78	23	439.33	10.43	23	65.3%	-8.00 [-14.43, -1.57]
Yukecul 2024 (non-CXL)	452	36	37	454	45	37	7.8%	-2.00 [-20.57, 16.57]
Yukecul 2024 (prior CXL)	449	42	30	437	38	30	0.0%	12.00 [-8.27, 32.27]
**Total (95% CI)**			**238**			**238**	**100.0%**	**-5.53 [-10.72, -0.33]**

Heterogeneity: $Tau^2 = 0.00$; $Chi^2 = 2.43$, $df = 5$ ($P = 0.79$); $I^2 = 0\%$
Test for overall effect: $Z = 2.08$ ($P = 0.04$)

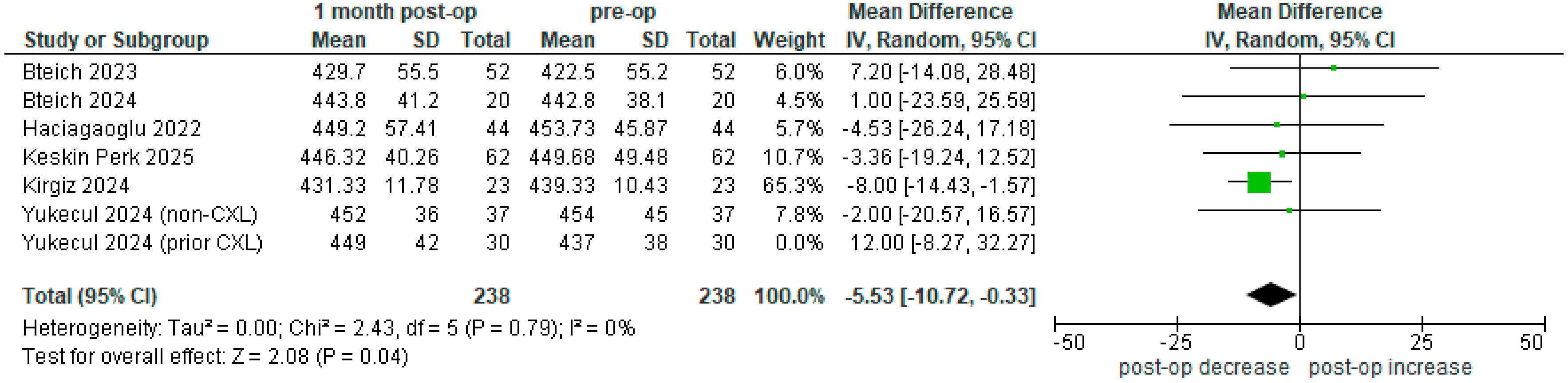

S1.4.1.4. Difference in pachymetry central point

Figure S1.4.1.4A. Difference in pachymetry central point in 1 study [6] – CXL

	1 month post-op			pre-op				Mean Difference
**Study or Subgroup**	**Mean**	**SD**	**Total**	**Mean**	**SD**	**Total**	**Weight**	**IV, Random, 95% CI**
Nacaroglu 2023	449.69	36.72	49	463.62	54.88	49	100.0%	-13.93 [-32.42, 4.56]
**Total (95% CI)**			**49**			**49**	**100.0%**	**-13.93 [-32.42, 4.56]**

Heterogeneity: Not applicable
Test for overall effect: Z = 1.48 (P = 0.14)

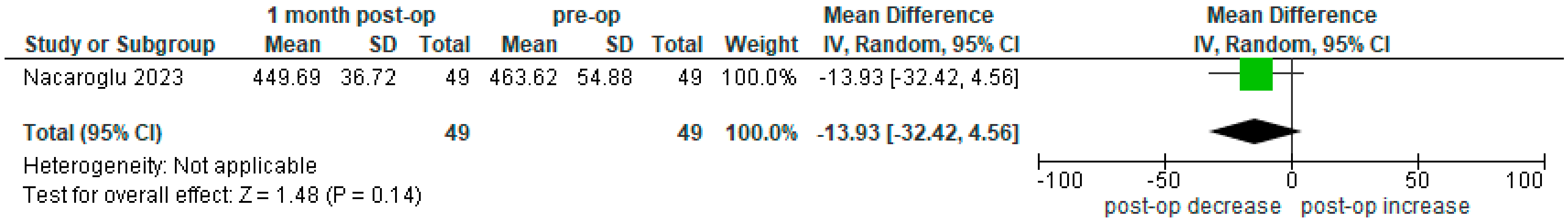

D.1.4B. Difference in pachymetry central point across 0 studies – no CXL

S1.4.1.5. Difference in maximum keratometry

Figure S1.4.1.5A. Difference in maximum keratometry across 2 studies [6, 7] – CXL

Study or Subgroup	1 month post-op Mean	SD	Total	pre-op Mean	SD	Total	Weight	Mean Difference IV, Random, 95% CI
Bteich 2023	54.17	8.22	52	58.09	7.92	52	0.0%	-3.92 [-7.02, -0.82]
Bteich 2024	53.3	6.22	20	57.21	7.53	20	0.0%	-3.91 [-8.19, 0.37]
Haciagaoglu 2022	55.46	6.97	44	57.56	7.06	44	0.0%	-2.10 [-5.03, 0.83]
Keskin Perk 2025	56.34	7.43	62	58.51	7.55	62	0.0%	-2.17 [-4.81, 0.47]
Kirgiz 2024	48.18	2.03	23	57.65	1.41	23	0.0%	-9.47 [-10.48, -8.46]
Mechleb 2024 (Kmax inf 75D)	51.93	5.79	6	54.43	4.09	6	0.0%	-2.50 [-8.17, 3.17]
Mechleb 2024 (Kmax sup 75D)	67.02	10.95	4	81.5	7.95	4	0.0%	-14.48 [-27.74, -1.22]
Nacaroglu 2023	56.75	7.72	49	59.2	7.66	49	53.6%	-2.45 [-5.50, 0.60]
Yukecul 2024 (non-CXL)	56.17	7.13	37	58.38	7.24	37	0.0%	-2.21 [-5.48, 1.06]
Yukecul 2024 (prior CXL)	55.28	6.26	30	57.22	6.68	30	46.4%	-1.94 [-5.22, 1.34]
**Total (95% CI)**			**79**			**79**	**100.0%**	**-2.21 [-4.44, 0.02]**

Heterogeneity: $Tau^2 = 0.00$; $Chi^2 = 0.05$, df = 1 (P = 0.82); $I^2 = 0\%$

Test for overall effect: Z = 1.95 (P = 0.05)

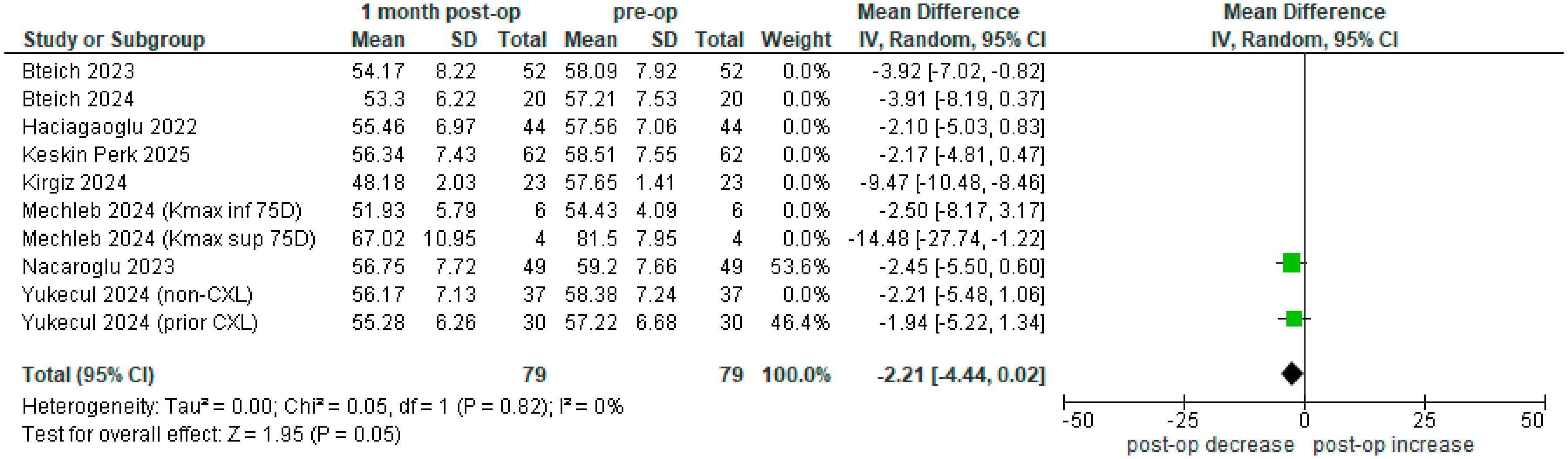

Figure S1.4.1.5B. Difference in maximum keratometry across 7 studies [1-5, 7, 8] – no CXL

	1 month post-op			pre-op				Mean Difference
**Study or Subgroup**	**Mean**	**SD**	**Total**	**Mean**	**SD**	**Total**	**Weight**	**IV, Random, 95% CI**
Bteich 2023	54.17	8.22	52	58.09	7.92	52	14.0%	-3.92 [-7.02, -0.82]
Bteich 2024	53.3	6.22	20	57.21	7.53	20	12.6%	-3.91 [-8.19, 0.37]
Haciagaoglu 2022	55.46	6.97	44	57.56	7.06	44	14.2%	-2.10 [-5.03, 0.83]
Keskin Perk 2025	56.34	7.43	62	58.51	7.55	62	14.5%	-2.17 [-4.81, 0.47]
Kirgiz 2024	48.18	2.03	23	57.65	1.41	23	15.8%	-9.47 [-10.48, -8.46]
Mechleb 2024 (Kmax inf 75D)	51.93	5.79	6	54.43	4.09	6	10.8%	-2.50 [-8.17, 3.17]
Mechleb 2024 (Kmax sup 75D)	67.02	10.95	4	81.5	7.95	4	4.4%	-14.48 [-27.74, -1.22]
Nacaroglu 2023	56.75	7.72	49	59.2	7.66	49	0.0%	-2.45 [-5.50, 0.60]
Yukecul 2024 (non-CXL)	56.17	7.13	37	58.38	7.24	37	13.8%	-2.21 [-5.48, 1.06]
Yukecul 2024 (prior CXL)	55.28	6.26	30	57.22	6.68	30	0.0%	-1.94 [-5.22, 1.34]
**Total (95% CI)**			**248**			**248**	**100.0%**	**-4.36 [-7.62, -1.10]**

Heterogeneity: $Tau^2 = 17.26$; $Chi^2 = 64.21$, df = 7 ($P < 0.00001$); $I^2 = 89\%$
Test for overall effect: Z = 2.62 (P = 0.009)

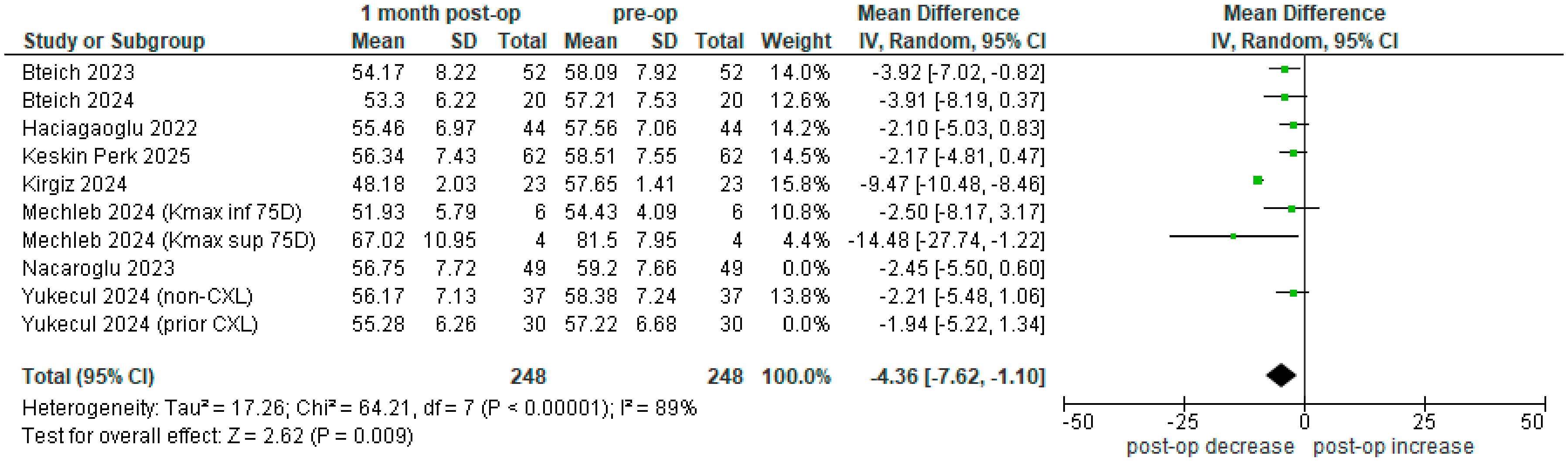

S1.4.1.6. Difference in mean simulated keratometry

Figure S1.4.1.6A. Difference in mean simulated keratometry across 2 studies [6, 7] – CXL

Study or Subgroup	1 month post-op Mean	SD	Total	pre-op Mean	SD	Total	Weight	Mean Difference IV, Random, 95% CI
Bteich 2023	45.66	5.04	52	48.92	4.49	52	0.0%	-3.26 [-5.09, -1.43]
Bteich 2024	46.28	3.62	20	49.3	4.83	20	0.0%	-3.02 [-5.67, -0.37]
Haciagaoglu 2022	45.72	4.85	44	49.01	5.11	44	0.0%	-3.29 [-5.37, -1.21]
Keskin Perk 2025	46.22	4.98	62	49.17	4.97	62	0.0%	-2.95 [-4.70, -1.20]
Kirgiz 2024	42.91	1.32	23	49.86	1.16	23	0.0%	-6.95 [-7.67, -6.23]
Mechleb 2024 (Kmax inf 75D)	44.925	3.91	6	49.665	3.16	6	0.0%	-4.74 [-8.76, -0.72]
Mechleb 2024 (Kmax sup 75D)	55.325	5.36	4	68.01	2.215	4	0.0%	-12.69 [-18.37, -7.00]
Nacaroglu 2023	46.32	5.35	49	49.34	5.32	49	59.2%	-3.02 [-5.13, -0.91]
Yukecul 2024 (non-CXL)	44.52	4.8	37	49.11	4.66	37	0.0%	-4.59 [-6.75, -2.43]
Yukecul 2024 (prior CXL)	45.58	4.45	30	49.46	5.55	30	40.8%	-3.88 [-6.43, -1.33]
**Total (95% CI)**			**79**			**79**	**100.0%**	**-3.37 [-5.00, -1.75]**

Heterogeneity: Tau² = 0.00; Chi² = 0.26, df = 1 (P = 0.61); I² = 0%
Test for overall effect: Z = 4.06 (P < 0.0001)

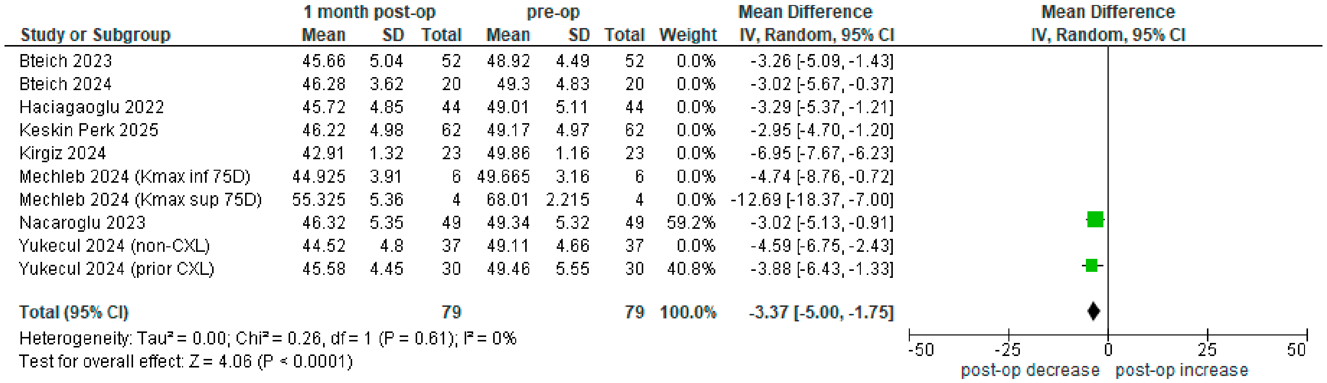

Figure S1.4.1.6B. Difference in mean simulated keratometry across 7 studies [1-5, 7, 8] – no CXL

	1 month post-op			pre-op				Mean Difference
Study or Subgroup	Mean	SD	Total	Mean	SD	Total	Weight	IV, Random, 95% CI
Bteich 2023	45.66	5.04	52	48.92	4.49	52	14.3%	-3.26 [-5.09, -1.43]
Bteich 2024	46.28	3.62	20	49.3	4.83	20	12.3%	-3.02 [-5.67, -0.37]
Haciagaoglu 2022	45.72	4.85	44	49.01	5.11	44	13.7%	-3.29 [-5.37, -1.21]
Keskin Perk 2025	46.22	4.98	62	49.17	4.97	62	14.5%	-2.95 [-4.70, -1.20]
Kirgiz 2024	42.91	1.32	23	49.86	1.16	23	16.4%	-6.95 [-7.67, -6.23]
Mechleb 2024 (Kmax inf 75D)	44.925	3.91	6	49.665	3.16	6	9.0%	-4.74 [-8.76, -0.72]
Mechleb 2024 (Kmax sup 75D)	55.325	5.36	4	68.01	2.215	4	6.2%	-12.69 [-18.37, -7.00]
Nacaroglu 2023	46.32	5.35	49	49.34	5.32	49	0.0%	-3.02 [-5.13, -0.91]
Yukecul 2024 (non-CXL)	44.52	4.8	37	49.11	4.66	37	13.5%	-4.59 [-6.75, -2.43]
Yukecul 2024 (prior CXL)	45.58	4.45	30	49.46	5.55	30	0.0%	-3.88 [-6.43, -1.33]
**Total (95% CI)**			**248**			**248**	**100.0%**	**-4.69 [-6.47, -2.92]**

Heterogeneity: $Tau^2 = 4.85$; $Chi^2 = 43.98$, df = 7 ($P < 0.00001$); $I^2 = 84\%$
Test for overall effect: Z = 5.19 ($P < 0.00001$)

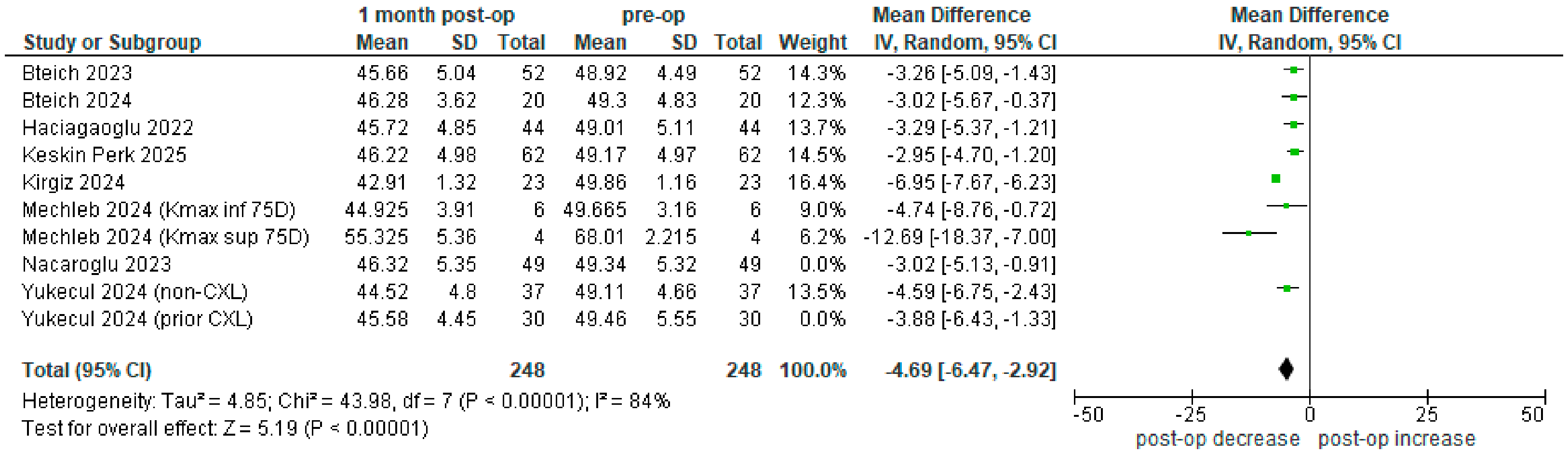

S1.4.1.7. Difference in total higher order aberrations

Figure S1.4.1.7A. Difference in total higher order aberrations across 0 studies – CXL

Figure S1.4.1.7B. Difference in total higher order aberrations across 2 studies [1, 2] – no CXL

	1 month post-op			pre-op				Mean Difference
**Study or Subgroup**	**Mean**	**SD**	**Total**	**Mean**	**SD**	**Total**	**Weight**	**IV, Random, 95% CI**
Bteich 2023	1.74	0.85	52	2.2	1.13	52	61.9%	-0.46 [-0.84, -0.08]
Bteich 2024	1.53	0.59	20	1.92	0.95	20	38.1%	-0.39 [-0.88, 0.10]
**Total (95% CI)**			**72**			**72**	**100.0%**	**-0.43 [-0.74, -0.13]**

Heterogeneity: Tau² = 0.00; Chi² = 0.05, df = 1 (P = 0.83); I² = 0%
Test for overall effect: Z = 2.81 (P = 0.005)

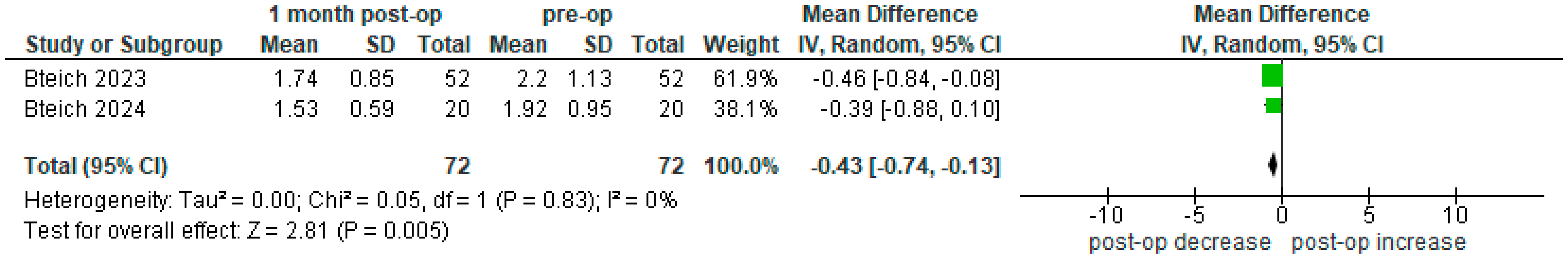

S1.4.1.8. Difference in spherical aberration

Figure S1.4.1.8A. Difference in spherical aberration across 0 studies – CXL

Figure S1.4.1.8B. Difference in spherical aberration across 4 studies [1, 2, 5, 8] – no CXL

	1 month post-op			pre-op				Mean Difference
**Study or Subgroup**	**Mean**	**SD**	**Total**	**Mean**	**SD**	**Total**	**Weight**	**IV, Random, 95% CI**
Bteich 2023	0.51	0.59	52	0.15	0.45	52	28.7%	0.36 [0.16, 0.56]
Bteich 2024	0.31	0.39	20	0.05	0.42	20	25.6%	0.26 [0.01, 0.51]
Kirgiz 2024	0.8	0.1	23	0.38	0.07	23	36.3%	0.42 [0.37, 0.47]
Mechleb 2024 (Kmax inf 75D)	0.13	0.5	6	-0.94	0.75	6	7.9%	1.07 [0.35, 1.79]
Mechleb 2024 (Kmax sup 75D)	-1.35	0.69	4	-4.76	1.73	4	1.5%	3.41 [1.58, 5.24]
**Total (95% CI)**			**105**			**105**	**100.0%**	**0.46 [0.23, 0.69]**

Heterogeneity: $Tau^2 = 0.04$; $Chi^2 = 15.30$, df = 4 (P = 0.004); $I^2 = 74\%$
Test for overall effect: Z = 3.92 (P < 0.0001)

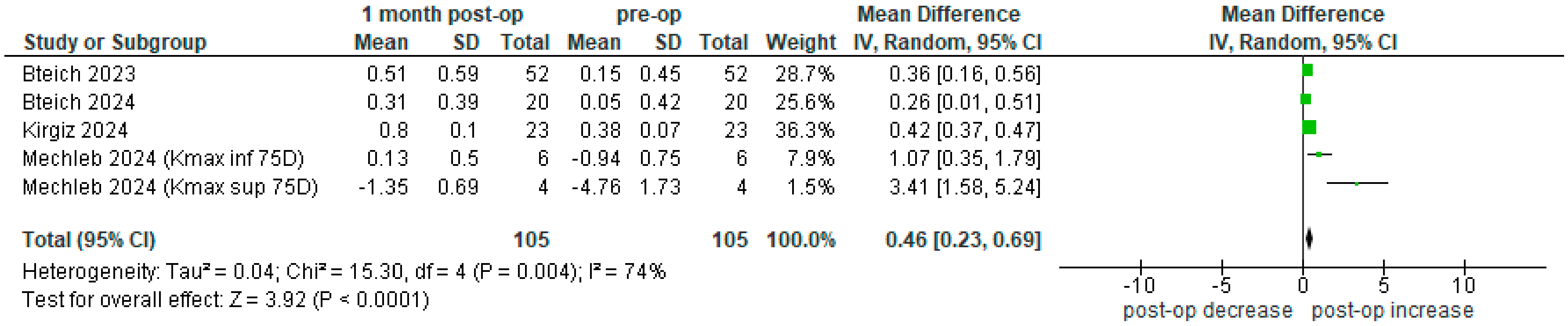

S1.4.1.9. Difference in vertical coma

Figure S1.4.1.9A. Difference in vertical coma across 0 studies – CXL

Figure S1.4.1.9B. Difference in vertical coma across 3 studies [1, 2, 8] – no CXL

	1 month post-op			pre-op				Mean Difference
**Study or Subgroup**	**Mean**	**SD**	**Total**	**Mean**	**SD**	**Total**	**Weight**	**IV, Random, 95% CI**
Bteich 2023	0.44	0.76	52	1.56	1.09	52	38.3%	-1.12 [-1.48, -0.76]
Bteich 2024	0.6	0.88	20	1.44	1.09	20	30.2%	-0.84 [-1.45, -0.23]
Mechleb 2024 (Kmax inf 75D)	1.16	1.19	6	2.03	0.52	6	18.9%	-0.87 [-1.91, 0.17]
Mechleb 2024 (Kmax sup 75D)	3.2	1.33	4	2.25	0.59	4	12.6%	0.95 [-0.48, 2.38]
**Total (95% CI)**			**82**			**82**	**100.0%**	**-0.73 [-1.32, -0.13]**

Heterogeneity: $Tau^2 = 0.21$; $Chi^2 = 7.82$, df = 3 (P = 0.05); $I^2 = 62\%$

Test for overall effect: Z = 2.39 (P = 0.02)

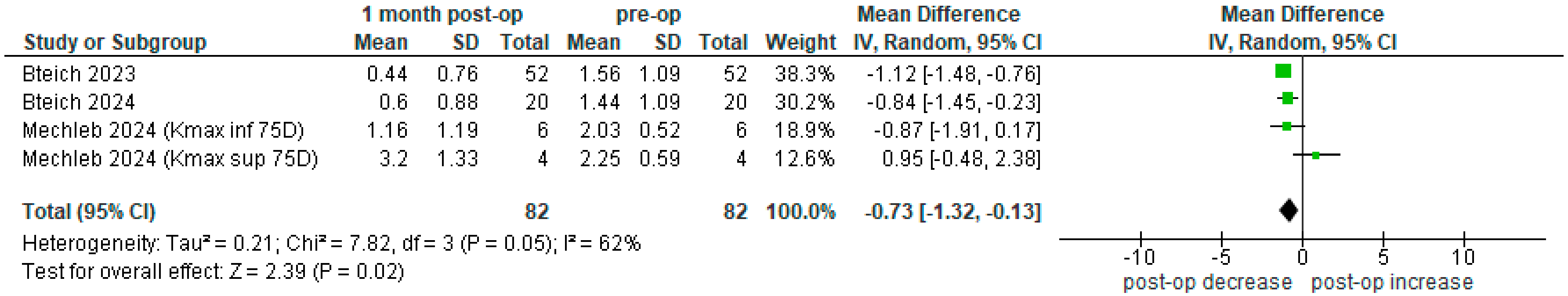

S1.4.1.10. Difference in horizontal coma

Figure S1.4.1.10A. Difference in horizontal coma across 0 studies – CXL

Figure S1.4.1.10B. Difference in horizontal coma across 0 studies – no CXL

S1.4.1.11. Difference in trefoil

Figure S1.4.1.11A. Difference in trefoil across 0 studies – CXL

Figure S1.4.1.11B. Difference in trefoil across 3 studies [1, 2, 5]– no CXL

	1 month post-op			pre-op				Mean Difference
**Study or Subgroup**	**Mean**	**SD**	**Total**	**Mean**	**SD**	**Total**	**Weight**	**IV, Random, 95% CI**
Bteich 2023	-0.12	0.64	52	-0.39	0.52	52	20.3%	0.27 [0.05, 0.49]
Bteich 2024	0.088	0.59	20	-0.27	0.4	20	11.7%	0.36 [0.05, 0.67]
Kirgiz 2024	1.12	0.1	23	0.66	0.13	23	68.0%	0.46 [0.39, 0.53]
**Total (95% CI)**			**95**			**95**	**100.0%**	**0.41 [0.29, 0.52]**

Heterogeneity: Tau² = 0.00; Chi² = 2.82, df = 2 (P = 0.24); I² = 29%
Test for overall effect: Z = 6.99 (P < 0.00001)

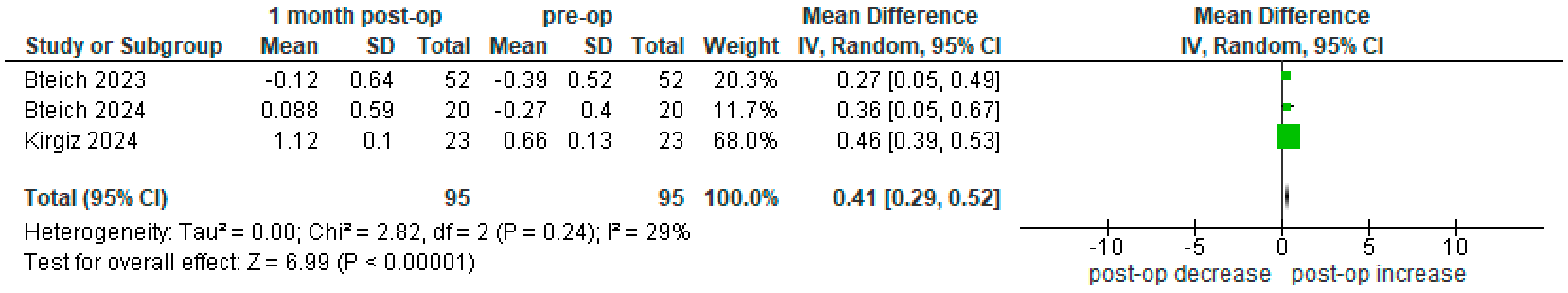

S1.4.1.12. Difference in total RMS

Figure S1.4.1.12A. Difference in total RMS across 0 studies – CXL

Figure S1.4.1.12B. Difference in total RMS across 2 studies [5, 8] – no CXL

	1 month post-op			pre-op				Mean Difference
**Study or Subgroup**	**Mean**	**SD**	**Total**	**Mean**	**SD**	**Total**	**Weight**	**IV, Random, 95% CI**
Kirgiz 2024	2.21	0.15	23	2.35	0.3	23	46.9%	-0.14 [-0.28, -0.00]
Mechleb 2024 (Kmax inf 75D)	8.45	5.87	6	14.1	4.04	6	31.6%	-5.65 [-11.35, 0.05]
Mechleb 2024 (Kmax sup 75D)	20.33	4.94	4	29.78	7.59	4	21.5%	-9.45 [-18.32, -0.58]
**Total (95% CI)**			**33**			**33**	**100.0%**	**-3.88 [-9.49, 1.72]**

Heterogeneity: Tau² = 17.41; Chi² = 7.81, df = 2 (P = 0.02); I² = 74%
Test for overall effect: Z = 1.36 (P = 0.17)

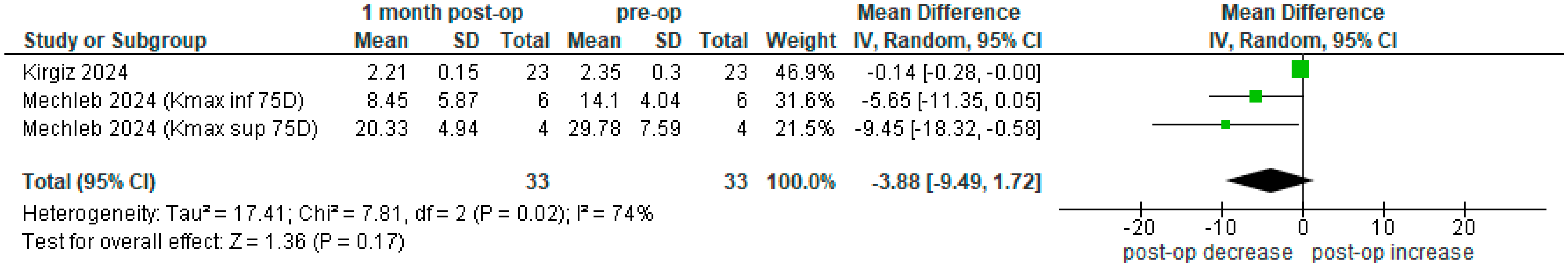

S1.4.2. 6 months postoperative versus preoperative

Figure S1.4.2.1A. Difference in uncorrected visual acuity across 2 studies [6, 7]- CXL

	6 months post-op			pre-op				Mean Difference
**Study or Subgroup**	**Mean**	**SD**	**Total**	**Mean**	**SD**	**Total**	**Weight**	**IV, Random, 95% CI**
Mazzotta 2024	0.52	0	2	1	0	2		Not estimable
Hayashi 2025	0.68	0.54	5	1.39	0.28	5	0.0%	-0.71 [-1.24, -0.18]
Colak 2025	0.48	0.21	4	1.2	0.5	4	0.0%	-0.72 [-1.25, -0.19]
Bteich 2023	0.68	0.23	4	0.93	0.31	4	0.0%	-0.25 [-0.63, 0.13]
Bteich 2023 ther refr	0.68	0.23	4	0.93	0.31	4	0.0%	-0.25 [-0.63, 0.13]
Yukecul 2024 (non-CXL)	0.31	0.21	37	0.77	0.76	37	0.0%	-0.46 [-0.71, -0.21]
Haciagaoglu 2022	0.35	0.29	44	0.72	0.63	44	0.0%	-0.37 [-0.57, -0.17]
Bteich 2024	0.51	0.18	20	0.893	0.33	20	0.0%	-0.38 [-0.55, -0.22]
Yukecul 2024 (prior CXL)	0.36	0.33	30	0.7	0.55	30	37.1%	-0.34 [-0.57, -0.11]
Keskin Perk 2025	0.44	0.34	62	0.96	0.5	62	0.0%	-0.52 [-0.67, -0.37]
Nacaroglu 2023	0.4	0.24	49	0.91	0.5	49	62.9%	-0.51 [-0.67, -0.35]
Kirgiz 2024	0.398	0.05	23	1.096	0.05	23	0.0%	-0.70 [-0.73, -0.67]
**Total (95% CI)**			**81**			**81**	**100.0%**	**-0.45 [-0.61, -0.29]**

Heterogeneity: $Tau^2 = 0.00$; $Chi^2 = 1.45$, $df = 1$ ($P = 0.23$); $I^2 = 31\%$
Test for overall effect: $Z = 5.44$ ($P < 0.00001$)

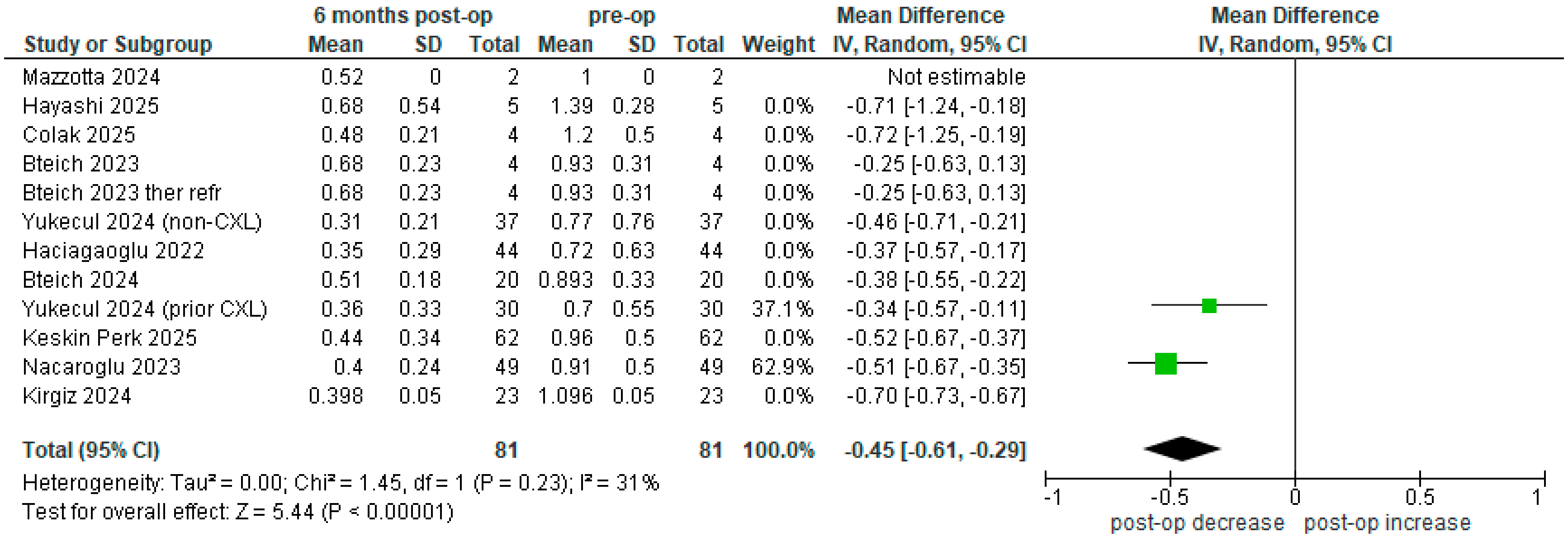

Figure S1.4.2.1B. Difference in uncorrected visual acuity across 9 studies [1-5, 7, 10-12]– no CXL

	6 months post-op			pre-op				Mean Difference
**Study or Subgroup**	**Mean**	**SD**	**Total**	**Mean**	**SD**	**Total**	**Weight**	**IV, Random, 95% CI**
Mazzotta 2024	0.52	0	2	1	0	2		Not estimable
Hayashi 2025	0.68	0.54	5	1.39	0.28	5	5.3%	-0.71 [-1.24, -0.18]
Colak 2025	0.48	0.21	4	1.2	0.5	4	5.4%	-0.72 [-1.25, -0.19]
Bteich 2023	0.68	0.23	4	0.93	0.31	4	8.2%	-0.25 [-0.63, 0.13]
Bteich 2023 ther refr	0.68	0.23	4	0.93	0.31	4	8.2%	-0.25 [-0.63, 0.13]
Yukecul 2024 (non-CXL)	0.31	0.21	37	0.77	0.76	37	11.7%	-0.46 [-0.71, -0.21]
Haciagaoglu 2022	0.35	0.29	44	0.72	0.63	44	13.4%	-0.37 [-0.57, -0.17]
Bteich 2024	0.51	0.18	20	0.893	0.33	20	14.7%	-0.38 [-0.55, -0.22]
Keskin Perk 2025	0.44	0.34	62	0.96	0.5	62	15.2%	-0.52 [-0.67, -0.37]
Kirgiz 2024	0.398	0.05	23	1.096	0.05	23	17.9%	-0.70 [-0.73, -0.67]
Nacaroglu 2023	0.4	0.24	49	0.91	0.5	49	0.0%	-0.51 [-0.67, -0.35]
Yukecul 2024 (prior CXL)	0.36	0.33	30	0.7	0.55	30	0.0%	-0.34 [-0.57, -0.11]
**Total (95% CI)**			**203**			**203**	**100.0%**	**-0.48 [-0.63, -0.33]**

Heterogeneity: Tau² = 0.03; Chi² = 39.46, df = 8 (P < 0.00001); I² = 80%
Test for overall effect: Z = 6.43 (P < 0.00001)

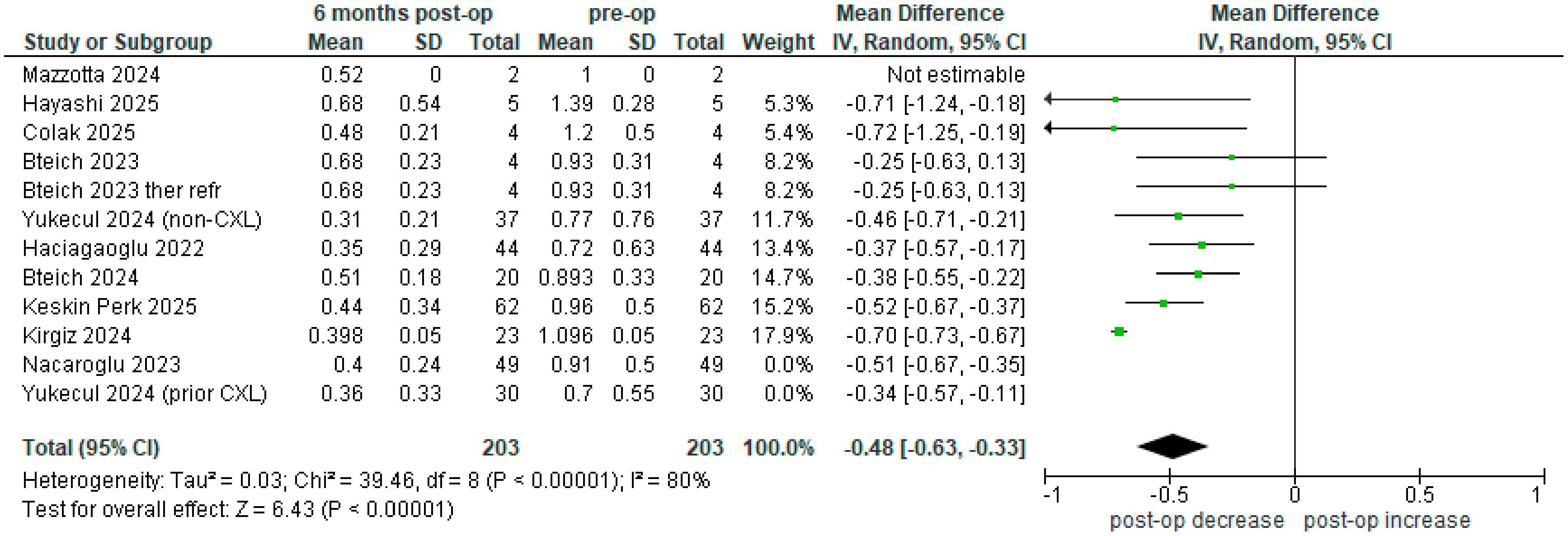

S1.4.2.2 Difference in best corrected visual acuity across 2 studies

Figure S1.4.2.2A. Difference in best corrected visual acuity across 2 studies [6, 7]- CXL

	6 months post-op			pre-op				Mean Difference
**Study or Subgroup**	**Mean**	**SD**	**Total**	**Mean**	**SD**	**Total**	**Weight**	**IV, Random, 95% CI**
Bteich 2023 ther refr	0.01	0.02	4	0.34	0.16	4	0.0%	-0.33 [-0.49, -0.17]
Bteich 2024	0.19	0.21	20	0.57	0.18	20	0.0%	-0.38 [-0.50, -0.26]
Colak 2025	0.4	0.14	4	0.53	0.13	4	0.0%	-0.13 [-0.32, 0.06]
Coscarelli 2024	0.301	0	3	1.06	0.22	3		Not estimable
Haciagaoglu 2022	0.25	0.22	44	0.54	0.37	44	0.0%	-0.29 [-0.42, -0.16]
Hayashi 2025	0.17	0.16	5	0.74	0.44	5	0.0%	-0.57 [-0.98, -0.16]
Keskin Perk 2025	0.25	0.24	62	0.72	0.47	62	0.0%	-0.47 [-0.60, -0.34]
Kirgiz 2024	0.167	0.05	23	0.602	0.3	23	0.0%	-0.43 [-0.56, -0.31]
Mechleb 2025	0.17	0.18	33	0.36	0.25	79	0.0%	-0.19 [-0.27, -0.11]
Nacaroglu 2023	0.31	0.09	49	0.87	0.2	49	53.7%	-0.56 [-0.62, -0.50]
Yukecul 2024 (non-CXL)	0.17	0.18	37	0.51	0.29	37	0.0%	-0.34 [-0.45, -0.23]
Yukecul 2024 (prior CXL)	0.27	0.23	30	0.55	0.39	30	46.3%	-0.28 [-0.44, -0.12]
**Total (95% CI)**			**79**			**79**	**100.0%**	**-0.43 [-0.70, -0.16]**

Heterogeneity: $Tau^2 = 0.04$; $Chi^2 = 10.03$, df = 1 (P = 0.002); $I^2 = 90\%$
Test for overall effect: Z = 3.08 (P = 0.002)

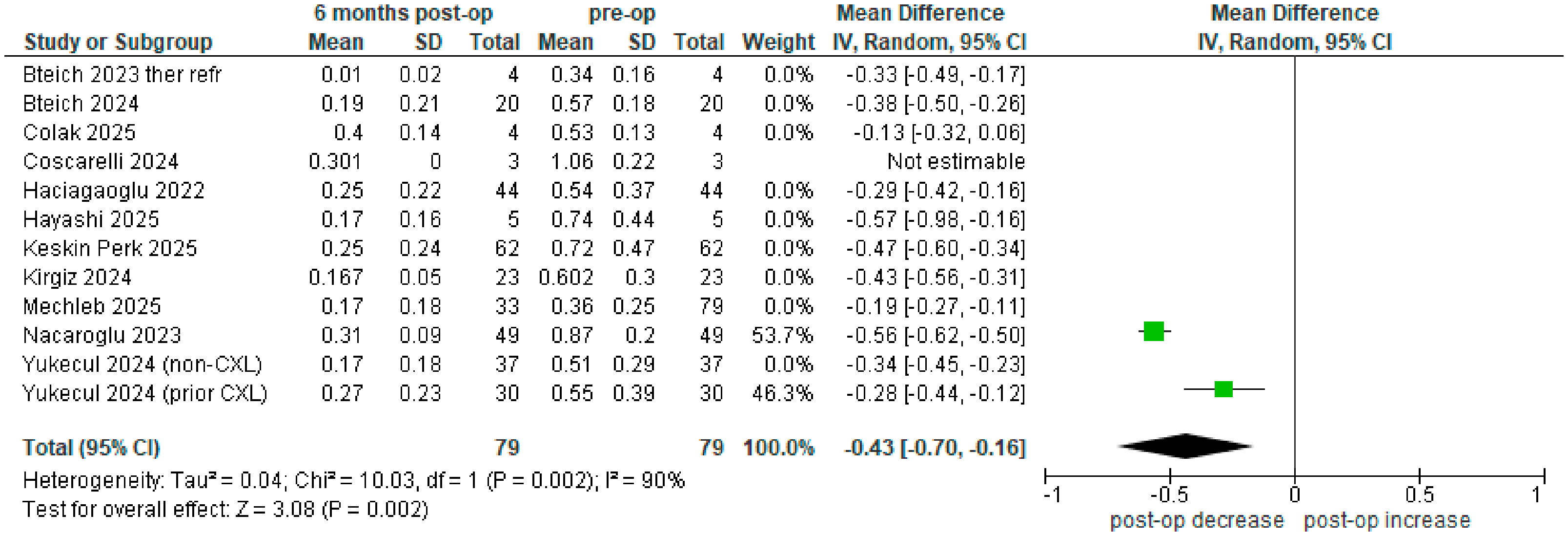

Figure S1.4.2.2B. Difference in best corrected visual acuity across 9 studies [2-5, 7, 10-12, 14]– no CXL

	6 months post-op			pre-op				Mean Difference
**Study or Subgroup**	**Mean**	**SD**	**Total**	**Mean**	**SD**	**Total**	**Weight**	**IV, Random, 95% CI**
Bteich 2023 ther refr	0.01	0.02	4	0.34	0.16	4	10.4%	-0.33 [-0.49, -0.17]
Bteich 2024	0.19	0.21	20	0.57	0.18	20	12.6%	-0.38 [-0.50, -0.26]
Colak 2025	0.4	0.14	4	0.53	0.13	4	8.9%	-0.13 [-0.32, 0.06]
Coscarelli 2024	0.301	0	3	1.06	0.22	3		Not estimable
Haciagaoglu 2022	0.25	0.22	44	0.54	0.37	44	12.2%	-0.29 [-0.42, -0.16]
Hayashi 2025	0.17	0.16	5	0.74	0.44	5	3.1%	-0.57 [-0.98, -0.16]
Keskin Perk 2025	0.25	0.24	62	0.72	0.47	62	12.0%	-0.47 [-0.60, -0.34]
Kirgiz 2024	0.167	0.05	23	0.602	0.3	23	12.4%	-0.43 [-0.56, -0.31]
Mechleb 2025	0.17	0.18	33	0.36	0.25	79	15.0%	-0.19 [-0.27, -0.11]
Nacaroglu 2023	0.31	0.09	49	0.87	0.2	49	0.0%	-0.56 [-0.62, -0.50]
Yukecul 2024 (non-CXL)	0.17	0.18	37	0.51	0.29	37	13.3%	-0.34 [-0.45, -0.23]
Yukecul 2024 (prior CXL)	0.27	0.23	30	0.55	0.39	30	0.0%	-0.28 [-0.44, -0.12]
**Total (95% CI)**			**235**			**281**	**100.0%**	**-0.33 [-0.41, -0.25]**

Heterogeneity: Tau² = 0.01; Chi² = 24.46, df = 8 (P = 0.002); I² = 67%
Test for overall effect: Z = 8.21 (P < 0.00001)

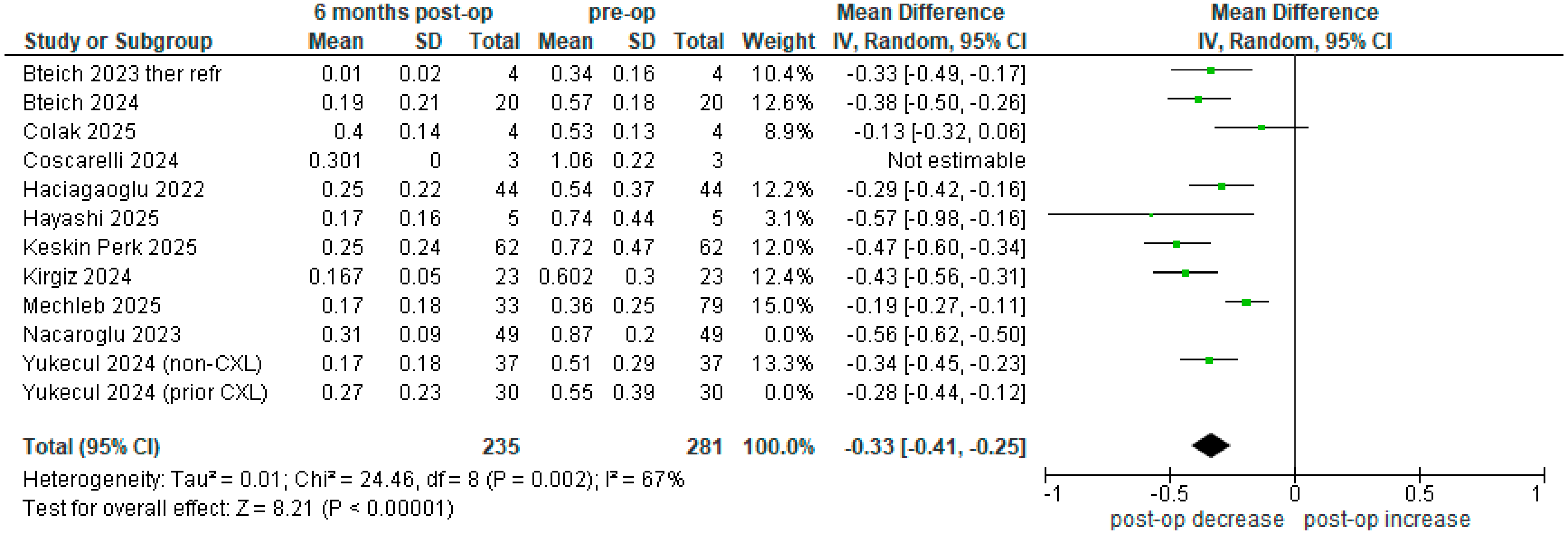

S1.4.2.3 Difference in pachymetry thinnest

Figure S1.4.2.3A. Difference in pachymetry thinnest across 2 studies [7, 9]– CXL

	6 months post-op			pre-op				Mean Difference
**Study or Subgroup**	**Mean**	**SD**	**Total**	**Mean**	**SD**	**Total**	**Weight**	**IV, Random, 95% CI**
Bteich 2024	447.5	51.5	20	442.8	38.1	20	0.0%	4.70 [-23.38, 32.78]
Haciagaoglu 2022	449.48	61.06	44	453.73	45.87	44	0.0%	-4.25 [-26.82, 18.32]
Keskin Perk 2025	445.7	47.35	62	449.68	49.48	62	0.0%	-3.98 [-21.03, 13.07]
Kirgiz 2024	445.5	17.5	23	439.33	10.43	23	0.0%	6.17 [-2.16, 14.50]
Mazzotta 2024	432	76.37	2	402.5	57.28	2	2.2%	29.50 [-102.80, 161.80]
Yukecul 2024 (non-CXL)	446	44	37	454	45	37	0.0%	-8.00 [-28.28, 12.28]
Yukecul 2024 (prior CXL)	452	41	30	437	38	30	97.8%	15.00 [-5.00, 35.00]
**Total (95% CI)**			**32**			**32**	**100.0%**	**15.32 [-4.45, 35.10]**

Heterogeneity: Tau² = 0.00; Chi² = 0.05, df = 1 (P = 0.83); I² = 0%

Test for overall effect: Z = 1.52 (P = 0.13)

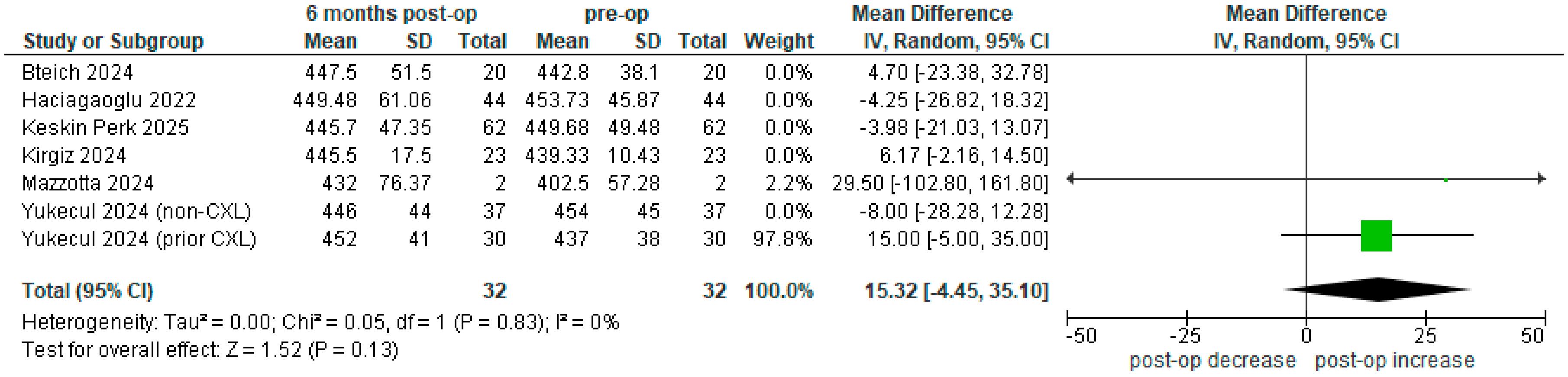

Figure S1.4.2.3B. Difference in pachymetry thinnest across 5 studies [2-5, 7]– no CXL

	6 months post-op			pre-op				Mean Difference
**Study or Subgroup**	**Mean**	**SD**	**Total**	**Mean**	**SD**	**Total**	**Weight**	**IV, Random, 95% CI**
Bteich 2024	447.5	51.5	20	442.8	38.1	20	5.4%	4.70 [-23.38, 32.78]
Haciagaoglu 2022	449.48	61.06	44	453.73	45.87	44	8.3%	-4.25 [-26.82, 18.32]
Keskin Perk 2025	445.7	47.35	62	449.68	49.48	62	14.6%	-3.98 [-21.03, 13.07]
Kirgiz 2024	445.5	17.5	23	439.33	10.43	23	61.3%	6.17 [-2.16, 14.50]
Mazzotta 2024	432	76.37	2	402.5	57.28	2	0.0%	29.50 [-102.80, 161.80]
Yukecul 2024 (non-CXL)	446	44	37	454	45	37	10.3%	-8.00 [-28.28, 12.28]
Yukecul 2024 (prior CXL)	452	41	30	437	38	30	0.0%	15.00 [-5.00, 35.00]
**Total (95% CI)**			**186**			**186**	**100.0%**	**2.27 [-4.25, 8.79]**

Heterogeneity: Tau² = 0.00; Chi² = 2.69, df = 4 (P = 0.61); I² = 0%
Test for overall effect: Z = 0.68 (P = 0.49)

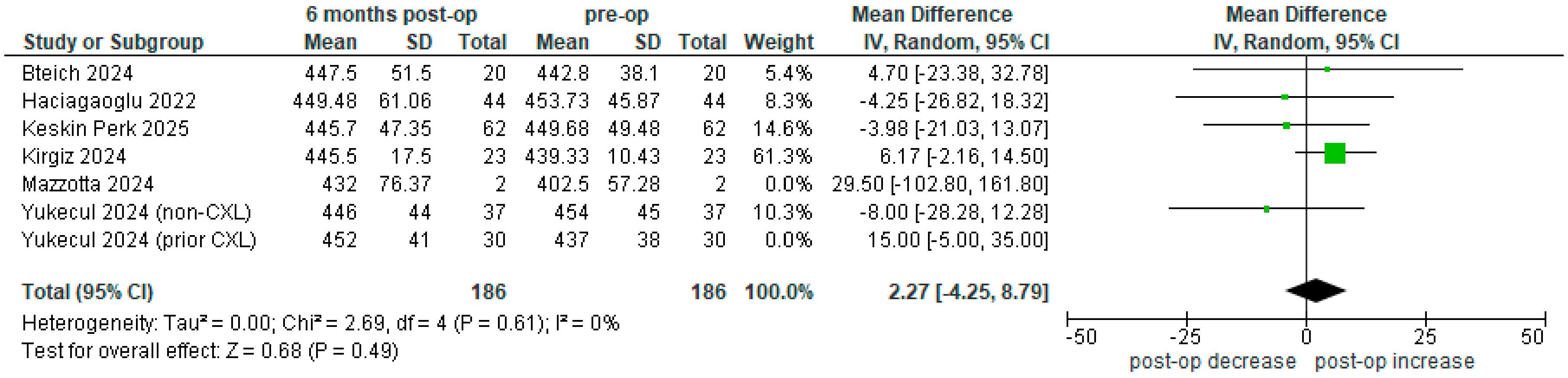

S1.4.2.4 Difference in pachymetry central point

Figure S1.4.2.4A. Difference in pachymetry central point in 1 study [6] – CXL

	6 months post-op			pre-op				Mean Difference
**Study or Subgroup**	**Mean**	**SD**	**Total**	**Mean**	**SD**	**Total**	**Weight**	**IV, Random, 95% CI**
Colak 2025	452.5	47.03	4	417.25	40.73	4	0.0%	35.25 [-25.72, 96.22]
Hayashi 2025	514.6	62.63	5	505.6	55.4	5	0.0%	9.00 [-64.29, 82.29]
Mechleb 2025	466	39	33	455	41	79	0.0%	11.00 [-5.09, 27.09]
Nacaroglu 2023	447.46	50.57	49	463.62	54.88	49	100.0%	-16.16 [-37.06, 4.74]
**Total (95% CI)**			**49**			**49**	**100.0%**	**-16.16 [-37.06, 4.74]**
Heterogeneity: Not applicable								
Test for overall effect: Z = 1.52 (P = 0.13)								

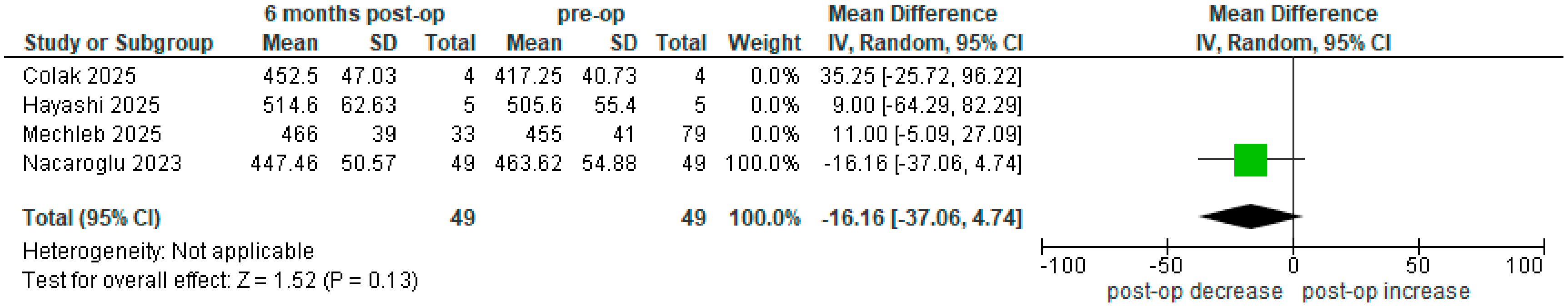

Figure S1.4.2.4B. Difference in pachymetry central point across 3 studies [10, 11, 14]– no CXL

	6 months post-op			pre-op				Mean Difference
**Study or Subgroup**	**Mean**	**SD**	**Total**	**Mean**	**SD**	**Total**	**Weight**	**IV, Random, 95% CI**
Colak 2025	452.5	47.03	4	417.25	40.73	4	6.2%	35.25 [-25.72, 96.22]
Hayashi 2025	514.6	62.63	5	505.6	55.4	5	4.3%	9.00 [-64.29, 82.29]
Mechleb 2025	466	39	33	455	41	79	89.5%	11.00 [-5.09, 27.09]
Nacaroglu 2023	447.46	50.57	49	463.62	54.88	49	0.0%	-16.16 [-37.06, 4.74]
**Total (95% CI)**			**42**			**88**	**100.0%**	**12.42 [-2.79, 27.64]**

Heterogeneity: Tau² = 0.00; Chi² = 0.58, df = 2 (P = 0.75); I² = 0%
Test for overall effect: Z = 1.60 (P = 0.11)

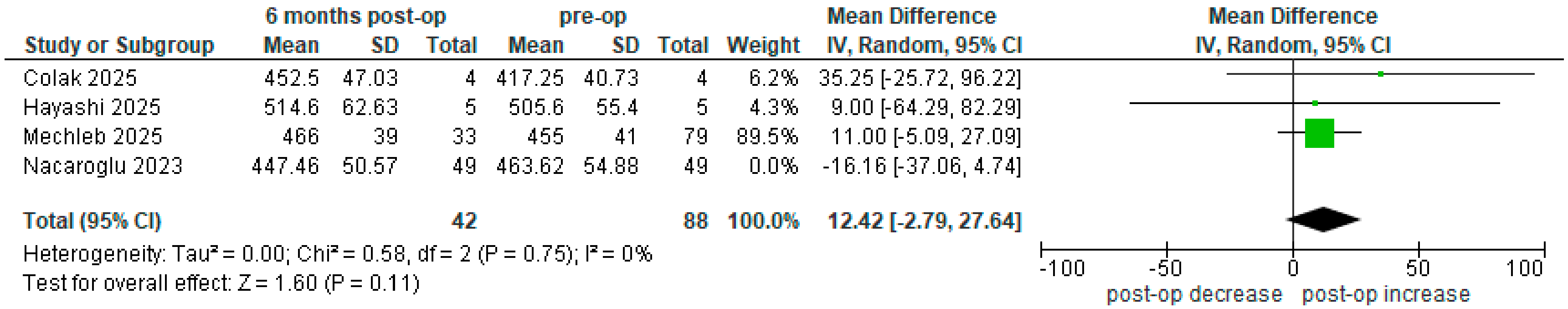

S1.4.2.5 Difference in maximum keratometry

Figure S1.4.2.5A Difference in maximum keratometry across 3[6, 7, 9] studies – CXL

	6 months post-op			pre-op				Mean Difference
**Study or Subgroup**	**Mean**	**SD**	**Total**	**Mean**	**SD**	**Total**	**Weight**	**IV, Random, 95% CI**
Bteich 2023 ther refr	47.89	3.05	4	50.02	1.99	4	0.0%	-2.13 [-5.70, 1.44]
Bteich 2024	52.38	6.02	20	57.21	7.53	20	0.0%	-4.83 [-9.06, -0.60]
Colak 2025	55.88	3.64	4	56.55	3.31	4	0.0%	-0.67 [-5.49, 4.15]
Haciagaoglu 2022	55.4	6.73	44	57.56	7.06	44	0.0%	-2.16 [-5.04, 0.72]
Hayashi 2025	59.62	6.78	5	63.58	8.26	5	0.0%	-3.96 [-13.33, 5.41]
Keskin Perk 2025	55.27	6.17	62	58.51	7.55	62	0.0%	-3.24 [-5.67, -0.81]
Kirgiz 2024	46.96	1.88	23	57.65	1.41	23	0.0%	-10.69 [-11.65, -9.73]
Mazzotta 2024	50.81	4.53	2	56.21	8.78	2	2.5%	-5.40 [-19.09, 8.29]
Mechleb 2025	55.6	6.4	33	57.6	6.9	79	0.0%	-2.00 [-4.66, 0.66]
Nacaroglu 2023	55.83	6.96	49	59.2	7.66	49	56.1%	-3.37 [-6.27, -0.47]
Yukecul 2024 (non-CXL)	55.66	6.29	37	58.38	7.24	37	0.0%	-2.72 [-5.81, 0.37]
Yukecul 2024 (prior CXL)	54.11	6.65	30	57.22	6.68	30	41.4%	-3.11 [-6.48, 0.26]
**Total (95% CI)**			**81**			**81**	**100.0%**	**-3.31 [-5.48, -1.14]**

Heterogeneity: $Tau^2 = 0.00$; $Chi^2 = 0.10$, $df = 2$ ($P = 0.95$); $I^2 = 0\%$
Test for overall effect: $Z = 2.99$ ($P = 0.003$)

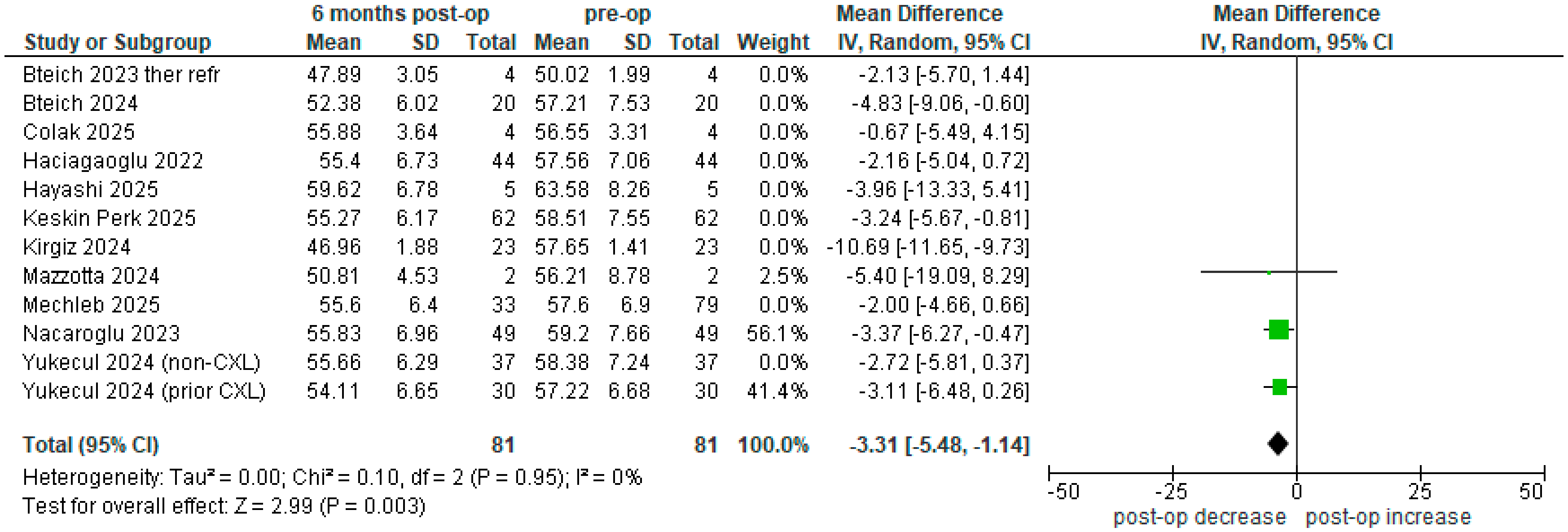

Figure S1.4.2.5B Difference in maximum keratometry across 9 studies [2-5, 7, 10-12, 14]– no CXL

	6 months post-op			pre-op				Mean Difference
**Study or Subgroup**	**Mean**	**SD**	**Total**	**Mean**	**SD**	**Total**	**Weight**	**IV, Random, 95% CI**
Bteich 2023 ther refr	47.89	3.05	4	50.02	1.99	4	11.4%	-2.13 [-5.70, 1.44]
Bteich 2024	52.38	6.02	20	57.21	7.53	20	10.9%	-4.83 [-9.06, -0.60]
Colak 2025	55.88	3.64	4	56.55	3.31	4	10.4%	-0.67 [-5.49, 4.15]
Haciagaoglu 2022	55.4	6.73	44	57.56	7.06	44	11.9%	-2.16 [-5.04, 0.72]
Hayashi 2025	59.62	6.78	5	63.58	8.26	5	6.7%	-3.96 [-13.33, 5.41]
Keskin Perk 2025	55.27	6.17	62	58.51	7.55	62	12.2%	-3.24 [-5.67, -0.81]
Kirgiz 2024	46.96	1.88	23	57.65	1.41	23	12.8%	-10.69 [-11.65, -9.73]
Mazzotta 2024	50.81	4.53	2	56.21	8.78	2	0.0%	-5.40 [-19.09, 8.29]
Mechleb 2025	55.6	6.4	33	57.6	6.9	79	12.0%	-2.00 [-4.66, 0.66]
Nacaroglu 2023	55.83	6.96	49	59.2	7.66	49	0.0%	-3.37 [-6.27, -0.47]
Yukecul 2024 (non-CXL)	55.66	6.29	37	58.38	7.24	37	11.8%	-2.72 [-5.81, 0.37]
Yukecul 2024 (prior CXL)	54.11	6.65	30	57.22	6.68	30	0.0%	-3.11 [-6.48, 0.26]
**Total (95% CI)**			**232**			**278**	**100.0%**	**-3.68 [-7.16, -0.21]**

Heterogeneity: Tau² = 24.22; Chi² = 111.47, df = 8 (P < 0.00001); I² = 93%
Test for overall effect: Z = 2.08 (P = 0.04)

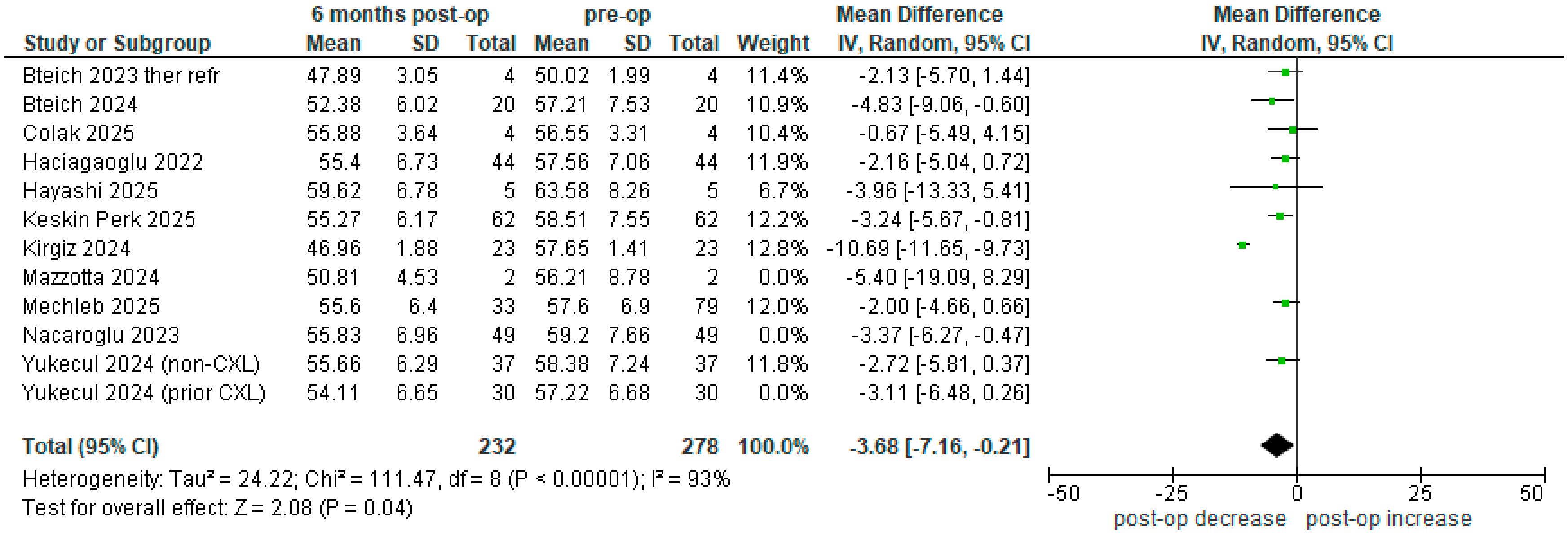

S1.4.2.6 Difference in mean simulated keratometry

Figure S1.4.2.6A Difference in mean simulated keratometry across 2 studies [6, 7]– CXL

	6 months post-op			pre-op				Mean Difference
**Study or Subgroup**	**Mean**	**SD**	**Total**	**Mean**	**SD**	**Total**	**Weight**	**IV, Random, 95% CI**
Bteich 2023 ther refr	43.18	1.52	4	44.32	0.95	4	0.0%	-1.14 [-2.90, 0.62]
Bteich 2024	45.45	3.57	20	49.3	4.83	20	0.0%	-3.85 [-6.48, -1.22]
Colak 2025	45.88	3.27	4	49.05	3.44	4	0.0%	-3.17 [-7.82, 1.48]
Coscarelli 2024	44.28	0.87	3	55.78	2.84	3	0.0%	-11.50 [-14.86, -8.14]
Haciagaoglu 2022	45.71	4.8	44	49.01	5.11	44	0.0%	-3.30 [-5.37, -1.23]
Keskin Perk 2025	45.75	4.47	62	49.17	4.97	62	0.0%	-3.42 [-5.08, -1.76]
Kirgiz 2024	42.4	1.37	23	49.86	1.16	23	0.0%	-7.46 [-8.19, -6.73]
Mechleb 2025	45.3	5	33	49.2	6.5	79	0.0%	-3.90 [-6.13, -1.67]
Nacaroglu 2023	45.76	5.02	49	49.34	5.32	49	60.7%	-3.58 [-5.63, -1.53]
Yukecul 2024 (non-CXL)	44.45	4.2	37	49.11	4.66	37	0.0%	-4.66 [-6.68, -2.64]
Yukecul 2024 (prior CXL)	46.34	4.46	30	49.46	5.55	30	39.3%	-3.12 [-5.67, -0.57]
**Total (95% CI)**			**79**			**79**	**100.0%**	**-3.40 [-5.00, -1.80]**

Heterogeneity: $Tau^2 = 0.00$; $Chi^2 = 0.08$, df = 1 (P = 0.78); $I^2 = 0\%$

Test for overall effect: Z = 4.17 (P < 0.0001)

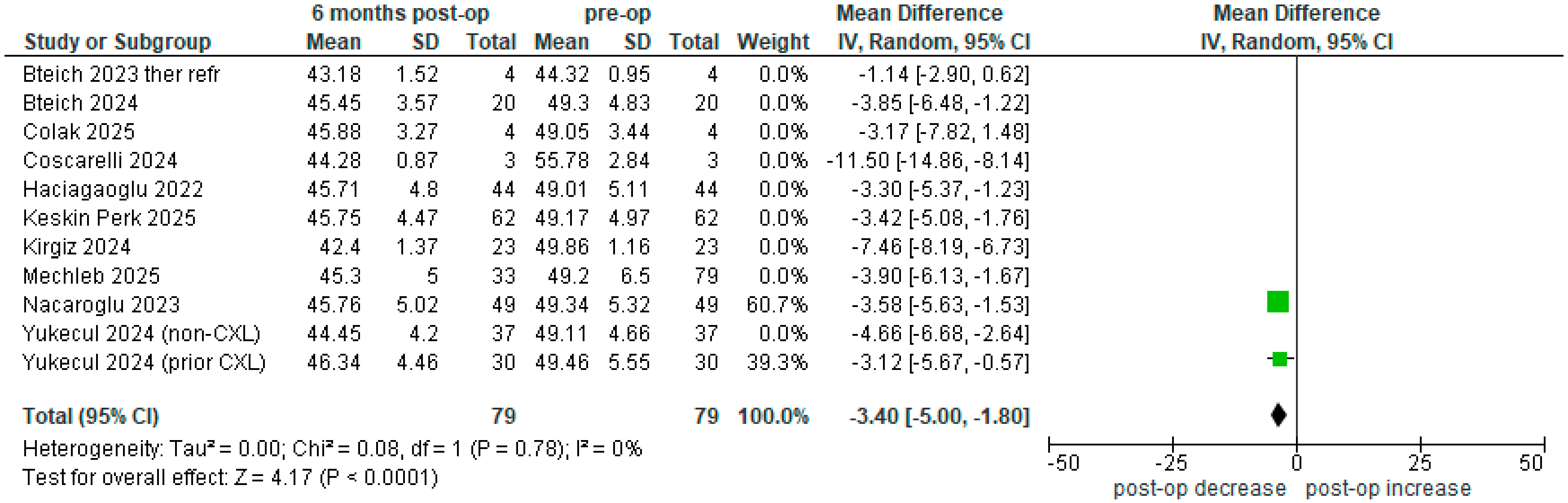

Figure S1.4.2.6B Difference in mean simulated keratometry across 9 studies [2-5, 7, 11-14]– no CXL

	6 months post-op			pre-op				Mean Difference
**Study or Subgroup**	**Mean**	**SD**	**Total**	**Mean**	**SD**	**Total**	**Weight**	**IV, Random, 95% CI**
Bteich 2023 ther refr	43.18	1.52	4	44.32	0.95	4	12.0%	-1.14 [-2.90, 0.62]
Bteich 2024	45.45	3.57	20	49.3	4.83	20	10.8%	-3.85 [-6.48, -1.22]
Colak 2025	45.88	3.27	4	49.05	3.44	4	7.7%	-3.17 [-7.82, 1.48]
Coscarelli 2024	44.28	0.87	3	55.78	2.84	3	9.6%	-11.50 [-14.86, -8.14]
Haciagaoglu 2022	45.71	4.8	44	49.01	5.11	44	11.6%	-3.30 [-5.37, -1.23]
Keskin Perk 2025	45.75	4.47	62	49.17	4.97	62	12.2%	-3.42 [-5.08, -1.76]
Kirgiz 2024	42.4	1.37	23	49.86	1.16	23	13.1%	-7.46 [-8.19, -6.73]
Mechleb 2025	45.3	5	33	49.2	6.5	79	11.4%	-3.90 [-6.13, -1.67]
Nacaroglu 2023	45.76	5.02	49	49.34	5.32	49	0.0%	-3.58 [-5.63, -1.53]
Yukecul 2024 (non-CXL)	44.45	4.2	37	49.11	4.66	37	11.7%	-4.66 [-6.68, -2.64]
Yukecul 2024 (prior CXL)	46.34	4.46	30	49.46	5.55	30	0.0%	-3.12 [-5.67, -0.57]
**Total (95% CI)**			**230**			**276**	**100.0%**	**-4.66 [-6.64, -2.69]**

Heterogeneity: Tau² = 7.65; Chi² = 78.36, df = 8 (P < 0.00001); I² = 90%
Test for overall effect: Z = 4.62 (P < 0.00001)

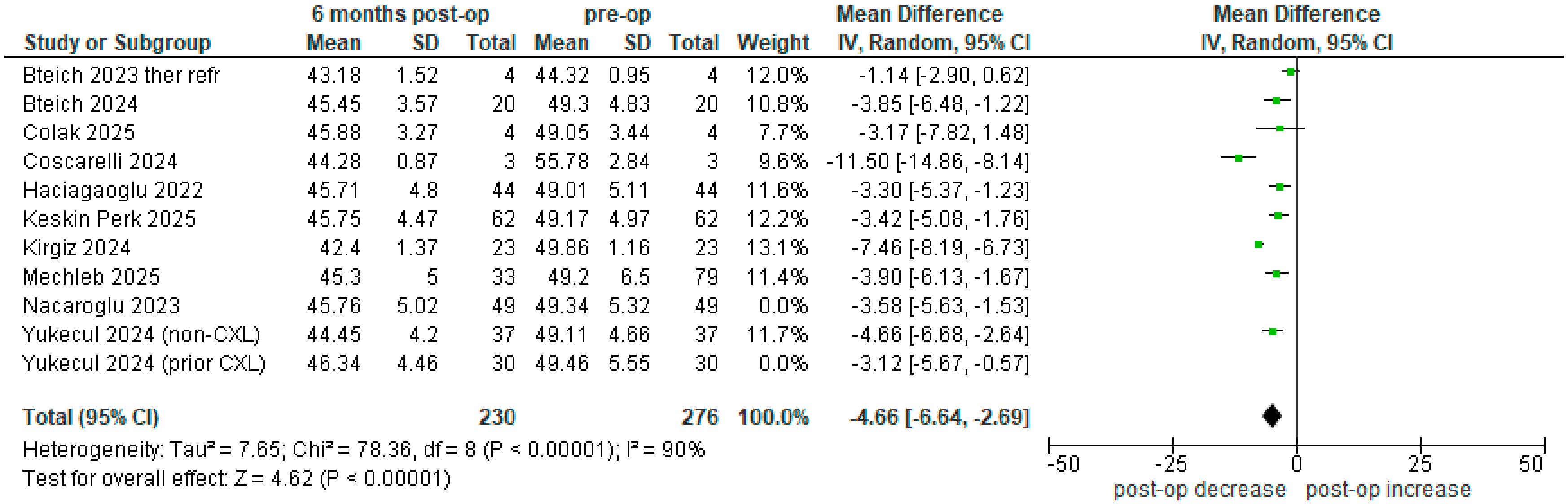

S1.4.2.7 Difference in total higher order aberrations

Figure S1.4.2.7A Difference in total higher order aberrations across 0 studies – CXL

Figure S1.4.2.7B Difference in total higher order aberrations across 3 studies [2, 10, 14]– no CXL

	6 months post-op			pre-op				Mean Difference
**Study or Subgroup**	**Mean**	**SD**	**Total**	**Mean**	**SD**	**Total**	**Weight**	**IV, Random, 95% CI**
Bteich 2024	1.39	0.62	20	1.92	0.95	20	69.6%	-0.53 [-1.03, -0.03]
Hayashi 2025	4.24	1.61	5	5.59	1.55	5	4.5%	-1.35 [-3.31, 0.61]
Mechleb 2025	3.47	2.08	33	3.78	1.82	79	25.9%	-0.31 [-1.13, 0.51]
**Total (95% CI)**			**58**			**104**	**100.0%**	**-0.51 [-0.92, -0.09]**

Heterogeneity: Tau² = 0.00; Chi² = 0.94, df = 2 (P = 0.62); I² = 0%
Test for overall effect: Z = 2.41 (P = 0.02)

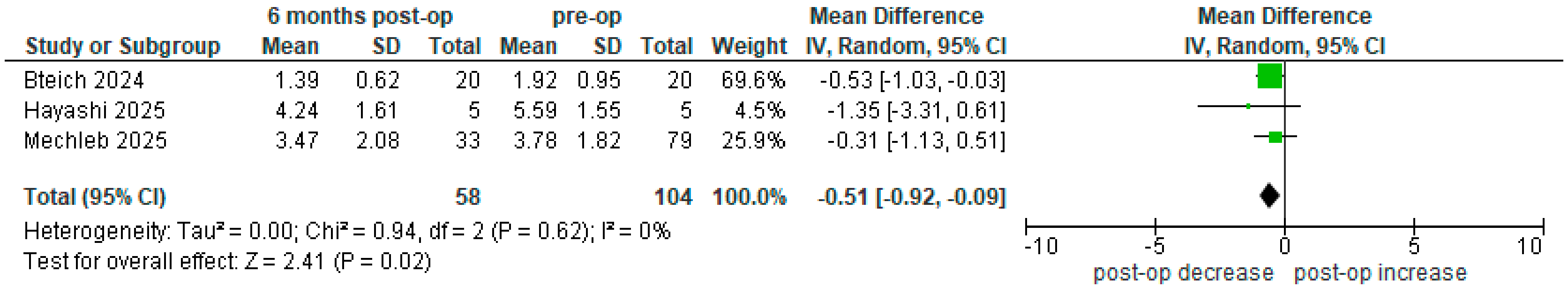

S1.4.2.8 Difference in spherical aberration

Figure S1.4.2.8A Difference in spherical aberration across 0 studies – CXL

Figure S1.4.2.8B Difference in spherical aberration across 3 studies [2, 5, 12]– no CXL

	6 months post-op			pre-op				Mean Difference
Study or Subgroup	Mean	SD	Total	Mean	SD	Total	Weight	IV, Random, 95% CI
Bteich 2023 ther refr	-0.09	0.27	4	0.26	0.08	4	31.0%	-0.35 [-0.63, -0.07]
Bteich 2024	0.32	0.46	20	0.05	0.42	20	31.1%	0.27 [-0.00, 0.54]
Kirgiz 2024	0.65	0.1	23	0.38	0.07	23	37.8%	0.27 [0.22, 0.32]
**Total (95% CI)**			**47**			**47**	**100.0%**	**0.08 [-0.28, 0.43]**

Heterogeneity: $Tau^2 = 0.09$; $Chi^2 = 18.80$, df = 2 (P < 0.0001); $I^2 = 89\%$
Test for overall effect: Z = 0.43 (P = 0.67)

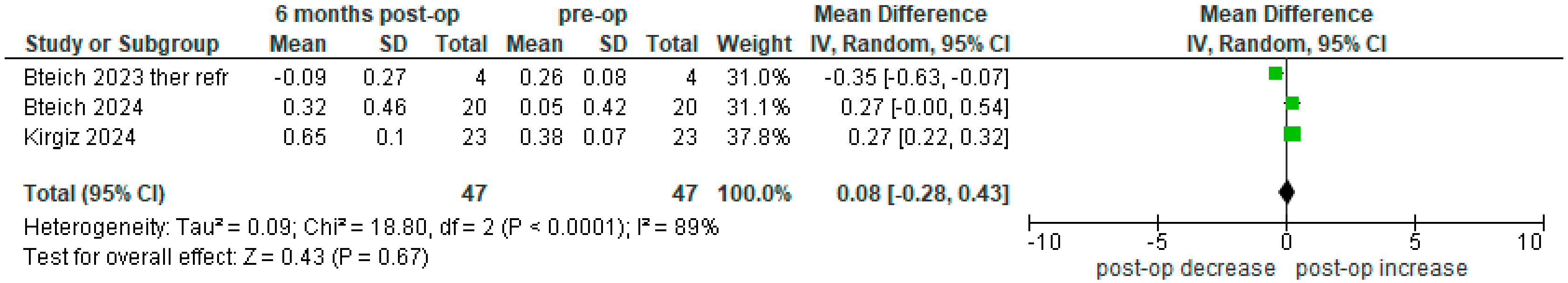

S1.4.2.9 Difference in vertical coma

Figure S1.4.2.9A Difference in vertical coma across 0 studies – CXL

Figure S1.4.2.9B Difference in vertical coma across 2 studies [2, 12]– no CXL

	6 months post-op			pre-op				Mean Difference
**Study or Subgroup**	**Mean**	**SD**	**Total**	**Mean**	**SD**	**Total**	**Weight**	**IV, Random, 95% CI**
Bteich 2023 ther refr	0.21	0.19	4	1.05	0.21	4	80.6%	-0.84 [-1.12, -0.56]
Bteich 2024	0.53	0.69	20	1.44	1.09	20	19.4%	-0.91 [-1.48, -0.34]
**Total (95% CI)**			**24**			**24**	**100.0%**	**-0.85 [-1.10, -0.60]**

Heterogeneity: Tau² = 0.00; Chi² = 0.05, df = 1 (P = 0.83); I² = 0%
Test for overall effect: Z = 6.72 (P < 0.00001)

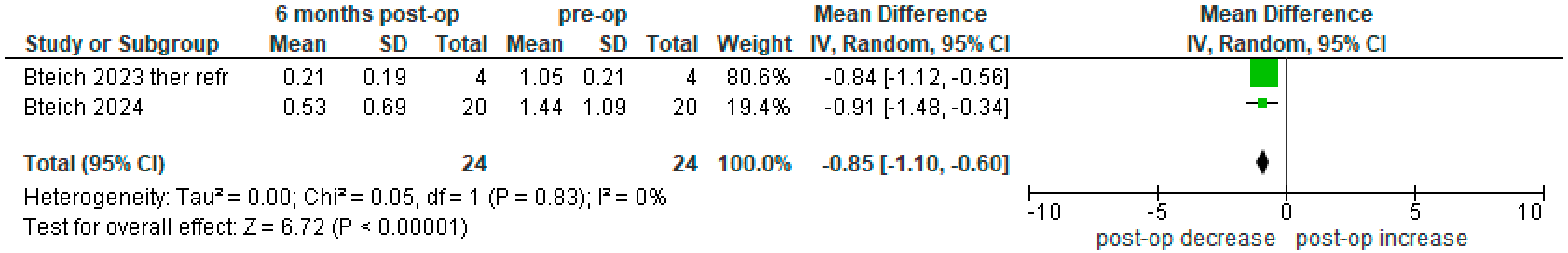

S1.4.2.10 Difference in trefoil

Figure S1.4.2.10A Difference in vertical coma across 0 studies – CXL

Figure S1.4.2.10B Difference in vertical coma across 2 studies [2, 5]– no CXL

	6 months post-op			pre-op				Mean Difference
**Study or Subgroup**	**Mean**	**SD**	**Total**	**Mean**	**SD**	**Total**	**Weight**	**IV, Random, 95% CI**
Bteich 2024	0.1	0.6	20	-0.27	0.4	20	43.0%	0.37 [0.05, 0.69]
Kirgiz 2024	1.45	0.11	23	0.66	0.13	23	57.0%	0.79 [0.72, 0.86]
**Total (95% CI)**			**43**			**43**	**100.0%**	**0.61 [0.20, 1.02]**

Heterogeneity: $Tau^2 = 0.07$; $Chi^2 = 6.47$, df = 1 (P = 0.01); $I^2 = 85\%$
Test for overall effect: Z = 2.93 (P = 0.003)

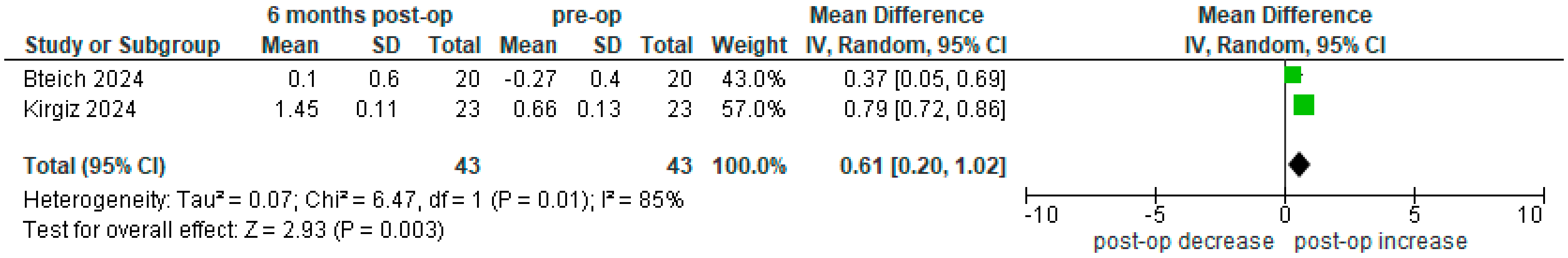

S1.4.2.11 Difference in trefoil

Figure S1.4.2.11A Difference in trefoil across 0 studies – CXL

Figure S1.4.2.11B Difference in trefoil across 2 studies [2, 5]– no CXL

	6 months post-op			pre-op				Mean Difference
**Study or Subgroup**	**Mean**	**SD**	**Total**	**Mean**	**SD**	**Total**	**Weight**	**IV, Random, 95% CI**
Bteich 2024	0.1	0.6	20	-0.27	0.4	20	43.0%	0.37 [0.05, 0.69]
Kirgiz 2024	1.45	0.11	23	0.66	0.13	23	57.0%	0.79 [0.72, 0.86]
**Total (95% CI)**			**43**			**43**	**100.0%**	**0.61 [0.20, 1.02]**

Heterogeneity: Tau² = 0.07; Chi² = 6.47, df = 1 (P = 0.01); I² = 85%
Test for overall effect: Z = 2.93 (P = 0.003)

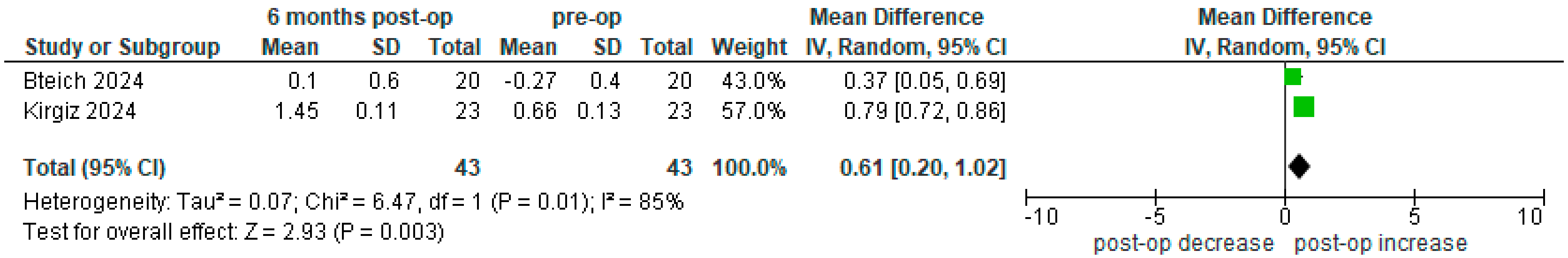

S1.4.2.12 Difference in total RMS

Figure S1.4.2.12A Difference in total RMS across 0 studies – CXL

Figure S1.4.2.12B Difference in total RMS across 2 studies [5, 14]– no CXL

	6 months post-op			pre-op				Mean Difference
**Study or Subgroup**	**Mean**	**SD**	**Total**	**Mean**	**SD**	**Total**	**Weight**	**IV, Random, 95% CI**
Kirgiz 2024	2.44	0.15	23	2.35	0.3	23	56.1%	0.09 [-0.05, 0.23]
Mechleb 2025	10.76	6.24	33	14.37	6.33	79	43.9%	-3.61 [-6.16, -1.06]
**Total (95% CI)**			**56**			**102**	**100.0%**	**-1.53 [-5.13, 2.07]**

Heterogeneity: Tau² = 6.00; Chi² = 8.09, df = 1 (P = 0.004); I² = 88%
Test for overall effect: Z = 0.83 (P = 0.40)

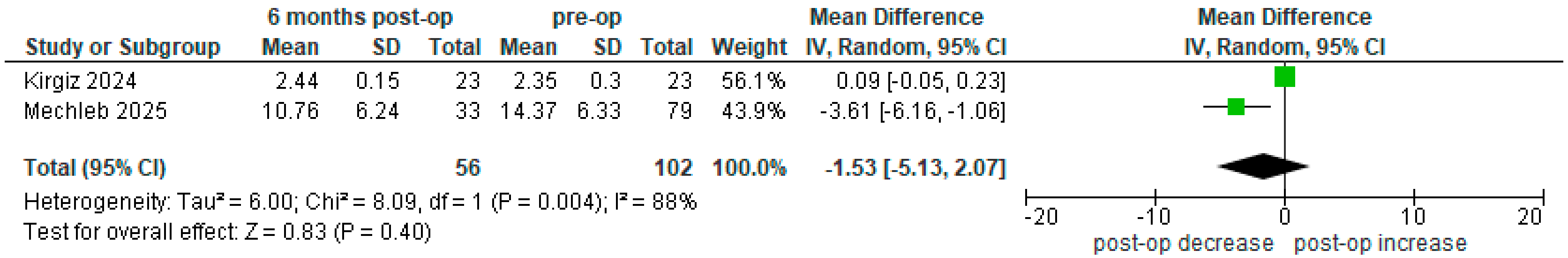

S1.4.3. 1 year postoperative versus preoperative

S1.4.3.1. Difference in uncorrected visual acuity

Figure S1.4.3.1A. Difference in uncorrected visual acuity across 3 studies [6, 16, 17] – CXL

	1 year post-op			pre-op				Mean Difference
**Study or Subgroup**	**Mean**	**SD**	**Total**	**Mean**	**SD**	**Total**	**Weight**	**IV, Random, 95% CI**
Asfar 2024	0.47	0.22	34	0.77	0.38	34	0.0%	-0.30 [-0.45, -0.15]
Bteich 2024	0.52	0.16	20	0.893	0.33	20	0.0%	-0.37 [-0.53, -0.21]
Jacob 2018	0.44	0.31	24	0.66	0.4	24	28.6%	-0.22 [-0.42, -0.02]
Jacob 2023	0.33	0.24	32	0.65	0.24	32	37.8%	-0.32 [-0.44, -0.20]
Keskin Perk 2025	0.44	0.35	50	0.96	0.5	62	0.0%	-0.52 [-0.68, -0.36]
Nacaroglu 2023	0.36	0.25	49	0.91	0.5	49	33.6%	-0.55 [-0.71, -0.39]
Yakut 2025	0.3	0.25	35	0.74	0.61	35	0.0%	-0.44 [-0.66, -0.22]
**Total (95% CI)**			**105**			**105**	**100.0%**	**-0.37 [-0.55, -0.19]**

Heterogeneity: $Tau^2 = 0.02$; $Chi^2 = 7.88$, $df = 2$ ($P = 0.02$); $I^2 = 75\%$
Test for overall effect: $Z = 4.04$ ($P < 0.0001$)

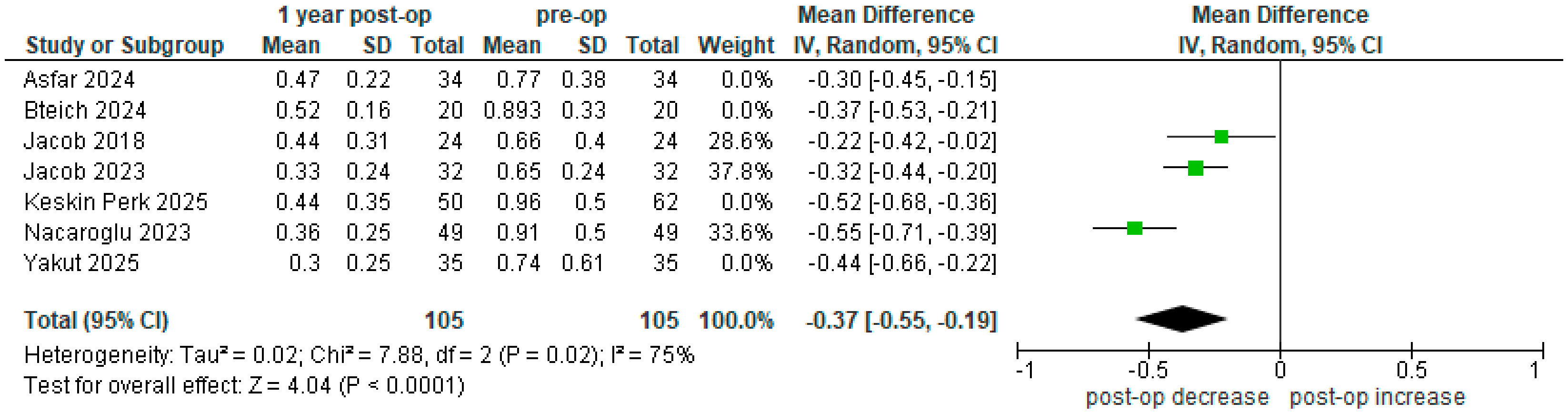

Figure S1.4.3.1B. Difference in uncorrected visual acuity across 4 studies [2, 4, 15, 18] – no CXL

	1 year post-op			pre-op				Mean Difference
**Study or Subgroup**	**Mean**	**SD**	**Total**	**Mean**	**SD**	**Total**	**Weight**	**IV, Random, 95% CI**
Asfar 2024	0.47	0.22	34	0.77	0.38	34	29.7%	-0.30 [-0.45, -0.15]
Bteich 2024	0.52	0.16	20	0.893	0.33	20	26.5%	-0.37 [-0.53, -0.21]
Jacob 2023	0.47	0.24	32	0.22	0.11	32	0.0%	0.25 [0.16, 0.34]
Keskin Perk 2025	0.44	0.35	50	0.96	0.5	62	27.1%	-0.52 [-0.68, -0.36]
Nacaroglu 2023	0.36	0.25	49	0.91	0.5	49	0.0%	-0.55 [-0.71, -0.39]
Yakut 2025	0.3	0.25	35	0.74	0.61	35	16.7%	-0.44 [-0.66, -0.22]
**Total (95% CI)**			**139**			**151**	**100.0%**	**-0.40 [-0.50, -0.30]**

Heterogeneity: $Tau^2 = 0.00$; $Chi^2 = 4.22$, $df = 3$ ($P = 0.24$); $I^2 = 29\%$

Test for overall effect: $Z = 7.94$ ($P < 0.00001$)

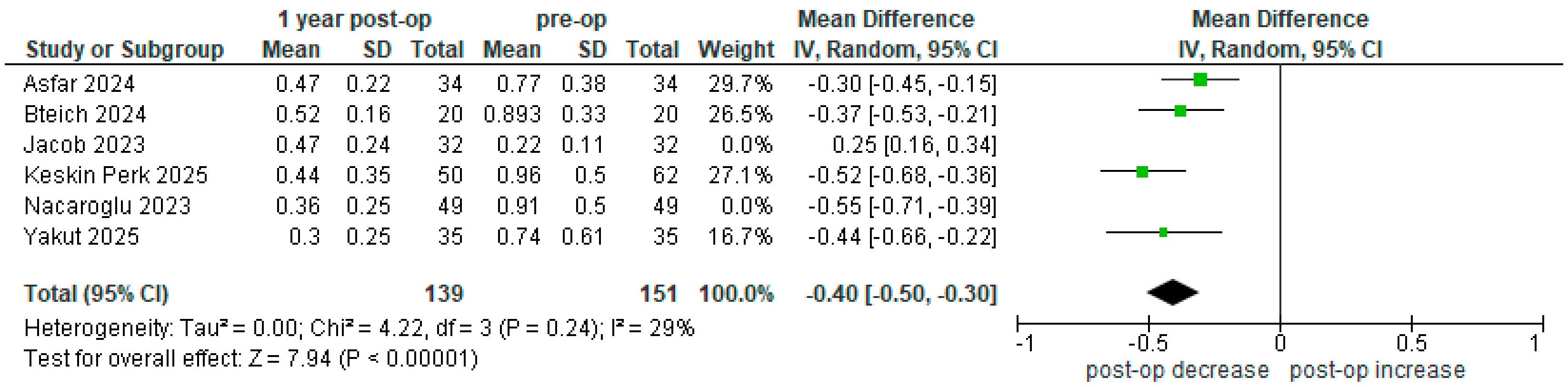

S1.4.3.2. Difference in best corrected visual acuity

Figure S1.4.3.2A. Difference in best corrected visual acuity across 3 studies [6, 16, 17] – CXL

	1 year post-op			pre-op				Mean Difference
**Study or Subgroup**	**Mean**	**SD**	**Total**	**Mean**	**SD**	**Total**	**Weight**	**IV, Random, 95% CI**
Asfar 2024	0.16	0.18	34	0.52	0.23	34	0.0%	-0.36 [-0.46, -0.26]
Bteich 2024	0.15	0.18	20	0.57	0.18	20	0.0%	-0.42 [-0.53, -0.31]
Jacob 2018	0.1	0.11	24	0.23	0.16	24	33.1%	-0.13 [-0.21, -0.05]
Jacob 2023	0.05	0.06	32	0.12	0.11	32	33.7%	-0.07 [-0.11, -0.03]
Keskin Perk 2025	0.23	0.21	50	0.72	0.47	62	0.0%	-0.49 [-0.62, -0.36]
Nacaroglu 2023	0.36	0.15	49	0.87	0.2	49	33.2%	-0.51 [-0.58, -0.44]
Yakut 2025	0.15	0.15	35	0.54	0.32	35	0.0%	-0.39 [-0.51, -0.27]
**Total (95% CI)**			**105**			**105**	**100.0%**	**-0.24 [-0.51, 0.03]**

Heterogeneity: Tau² = 0.06; Chi² = 111.63, df = 2 (P < 0.00001); I² = 98%
Test for overall effect: Z = 1.71 (P = 0.09)

Mean Difference
IV, Random, 95% CI

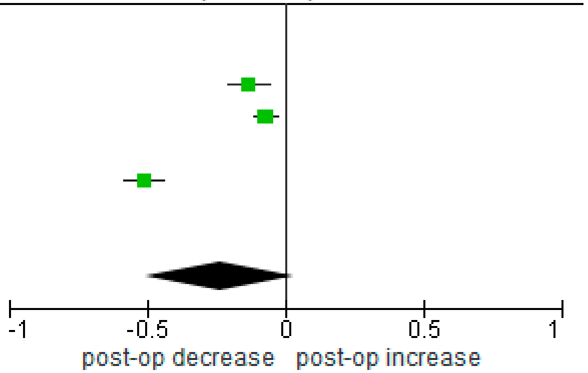

Figure S1.4.3.2B. Difference in best corrected visual acuity across 4 studies [2, 4, 15, 18] – no CXL

	1 year post-op			pre-op				Mean Difference
**Study or Subgroup**	**Mean**	**SD**	**Total**	**Mean**	**SD**	**Total**	**Weight**	**IV, Random, 95% CI**
Asfar 2024	0.16	0.18	34	0.52	0.23	34	32.9%	-0.36 [-0.46, -0.26]
Bteich 2024	0.15	0.18	20	0.57	0.18	20	25.5%	-0.42 [-0.53, -0.31]
Jacob 2023	0.89	0.17	32	0.76	0.19	32	0.0%	0.13 [0.04, 0.22]
Keskin Perk 2025	0.23	0.21	50	0.72	0.47	62	18.6%	-0.49 [-0.62, -0.36]
Nacaroglu 2023	0.36	0.15	49	0.87	0.2	49	0.0%	-0.51 [-0.58, -0.44]
Yakut 2025	0.15	0.15	35	0.54	0.32	35	23.1%	-0.39 [-0.51, -0.27]
**Total (95% CI)**			**139**			**151**	**100.0%**	**-0.41 [-0.46, -0.35]**

Heterogeneity: Tau² = 0.00; Chi² = 2.56, df = 3 (P = 0.46); I² = 0%
Test for overall effect: Z = 14.15 (P < 0.00001)

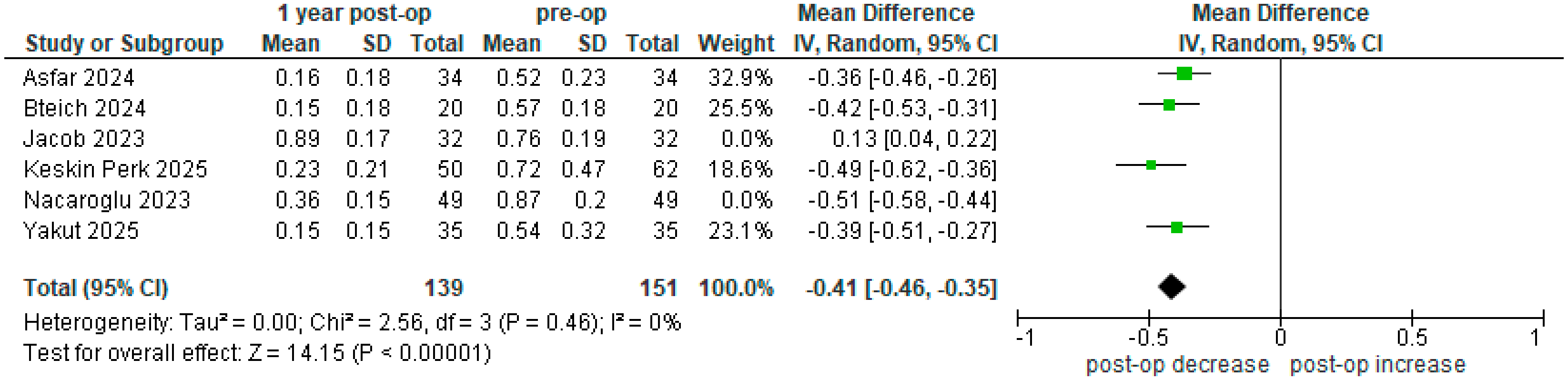

S1.4.3.3. Difference in pachymetry thinnest point

Figure S1.4.3.3A. Difference in pachymetry thinnest point in one study [17] – CXL

	1 year post-op			pre-op				Mean Difference
**Study or Subgroup**	**Mean**	**SD**	**Total**	**Mean**	**SD**	**Total**	**Weight**	**IV, Random, 95% CI**
Bteich 2024	442	51.8	20	442.8	38.1	20	0.0%	-0.80 [-28.98, 27.38]
Jacob 2023	455.94	45.74	32	449.88	35.99	32	100.0%	6.06 [-14.11, 26.23]
Keskin Perk 2025	444.38	50.15	50	449.68	49.48	62	0.0%	-5.30 [-23.87, 13.27]
**Total (95% CI)**			**32**			**32**	**100.0%**	**6.06 [-14.11, 26.23]**

Heterogeneity: Not applicable
Test for overall effect: Z = 0.59 (P = 0.56)

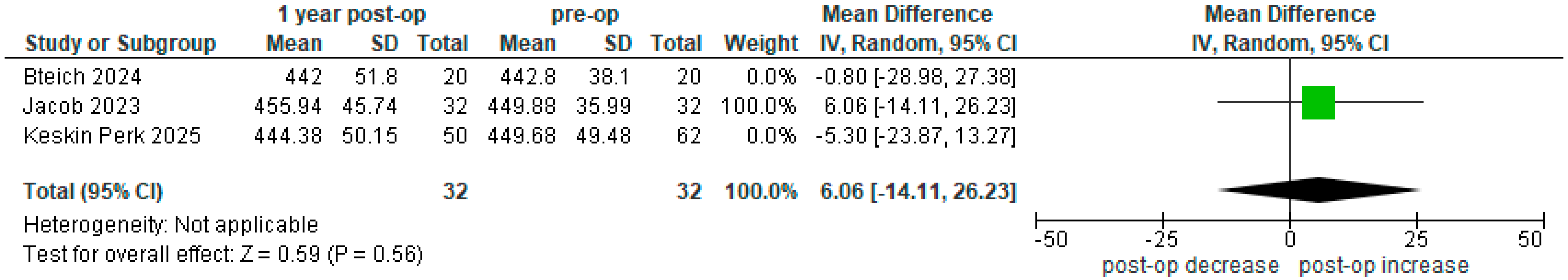

Figure S1.4.3.3B. Difference in pachymetry thinnest point across 2 studies [2, 4] – no CXL

	1 year post-op			pre-op				Mean Difference
**Study or Subgroup**	**Mean**	**SD**	**Total**	**Mean**	**SD**	**Total**	**Weight**	**IV, Random, 95% CI**
Bteich 2024	442	51.8	20	442.8	38.1	20	30.3%	-0.80 [-28.98, 27.38]
Jacob 2023	455.94	45.74	32	449.88	35.99	32	0.0%	6.06 [-14.11, 26.23]
Keskin Perk 2025	444.38	50.15	50	449.68	49.48	62	69.7%	-5.30 [-23.87, 13.27]
**Total (95% CI)**			**70**			**82**	**100.0%**	**-3.94 [-19.44, 11.57]**

Heterogeneity: Tau² = 0.00; Chi² = 0.07, df = 1 (P = 0.79); I² = 0%
Test for overall effect: Z = 0.50 (P = 0.62)

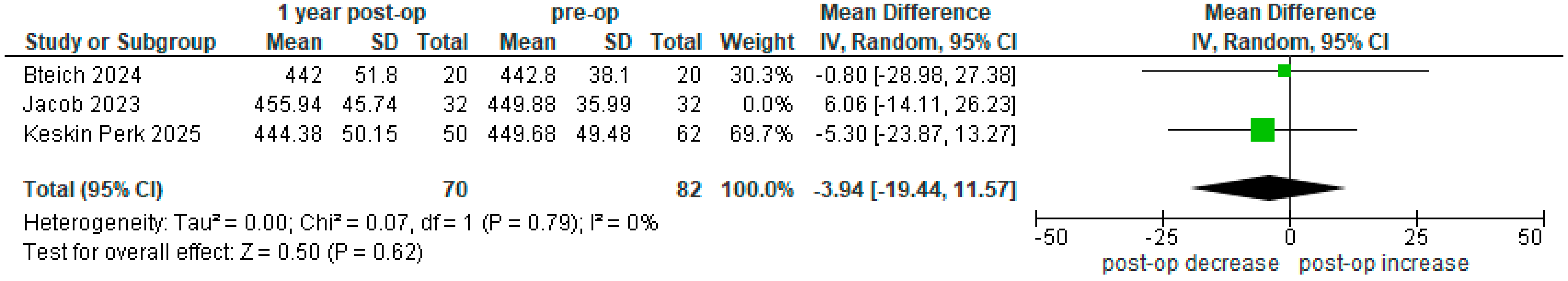

S1.4.3.4. Difference in pachymetry central point

Figure S1.4.3.4A. Difference in pachymetry central point in 1 study [6] – CXL

	1 year post-op			pre-op				Mean Difference
**Study or Subgroup**	**Mean**	**SD**	**Total**	**Mean**	**SD**	**Total**	**Weight**	**IV, Random, 95% CI**
Nacaroglu 2023	447.31	56.35	49	463.62	54.88	49	100.0%	-16.31 [-38.33, 5.71]
Yakut 2025	452	50.75	35	453.91	47.65	35	0.0%	-1.91 [-24.97, 21.15]
**Total (95% CI)**			**49**			**49**	**100.0%**	**-16.31 [-38.33, 5.71]**
Heterogeneity: Not applicable								
Test for overall effect: Z = 1.45 (P = 0.15)								

Mean Difference
IV, Random, 95% CI

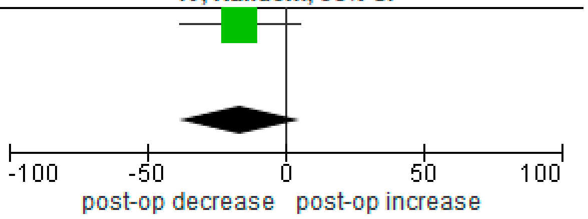

Figure S1.4.3.4B. Difference in pachymetry central point in 1 study [18] – No CXL

	1 year post-op			pre-op				Mean Difference
**Study or Subgroup**	**Mean**	**SD**	**Total**	**Mean**	**SD**	**Total**	**Weight**	**IV, Random, 95% CI**
Nacaroglu 2023	447.31	56.35	49	463.62	54.88	49	0.0%	-16.31 [-38.33, 5.71]
Yakut 2025	452	50.75	35	453.91	47.65	35	100.0%	-1.91 [-24.97, 21.15]
**Total (95% CI)**			**35**			**35**	**100.0%**	**-1.91 [-24.97, 21.15]**
Heterogeneity: Not applicable								
Test for overall effect: Z = 0.16 (P = 0.87)								

Mean Difference
IV, Random, 95% CI

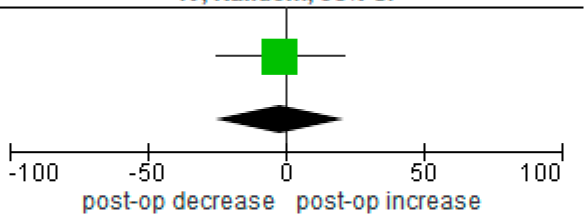

S1.4.3.5. Difference in maximum keratometry

Figure S1.4.3.5A. Difference in maximum keratometry across 2 studies [6, 17] – CXL

	1 year post-op			pre-op				Mean Difference
**Study or Subgroup**	**Mean**	**SD**	**Total**	**Mean**	**SD**	**Total**	**Weight**	**IV, Random, 95% CI**
Asfar 2024	50.69	6.38	34	55.85	7.53	34	0.0%	-5.16 [-8.48, -1.84]
Bteich 2024	52.85	5.44	20	57.21	7.53	20	0.0%	-4.36 [-8.43, -0.29]
Jacob 2023	54.95	4.71	32	58.9	5.54	32	54.3%	-3.95 [-6.47, -1.43]
Keskin Perk 2025	55.17	5.87	50	58.51	7.55	62	0.0%	-3.34 [-5.83, -0.85]
Nacaroglu 2023	55.63	6.14	49	59.2	7.66	49	45.7%	-3.57 [-6.32, -0.82]
Yakut 2025	54.29	5.61	35	57	6.81	35	0.0%	-2.71 [-5.63, 0.21]
**Total (95% CI)**			**81**			**81**	**100.0%**	**-3.78 [-5.63, -1.92]**

Heterogeneity: Tau² = 0.00; Chi² = 0.04, df = 1 (P = 0.84); I² = 0%
Test for overall effect: Z = 3.99 (P < 0.0001)

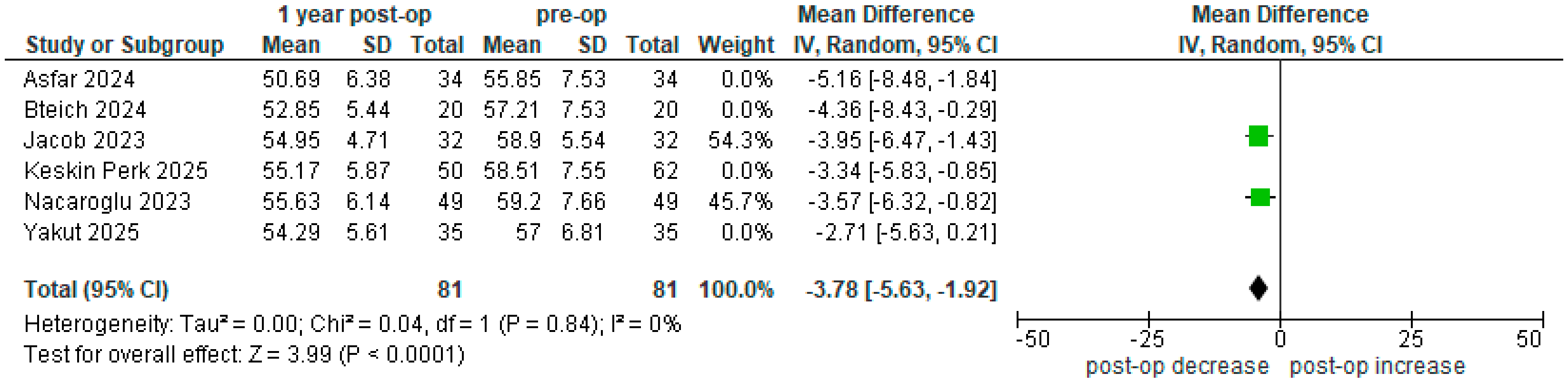

Figure S1.4.3.5B. Difference in maximum keratometry across 4 studies [2, 4, 15, 18] – no CXL

	1 year post-op			pre-op				Mean Difference
**Study or Subgroup**	**Mean**	**SD**	**Total**	**Mean**	**SD**	**Total**	**Weight**	**IV, Random, 95% CI**
Asfar 2024	50.69	6.38	34	55.85	7.53	34	21.1%	-5.16 [-8.48, -1.84]
Bteich 2024	52.85	5.44	20	57.21	7.53	20	14.0%	-4.36 [-8.43, -0.29]
Jacob 2023	54.95	4.71	32	58.9	5.54	32	0.0%	-3.95 [-6.47, -1.43]
Keskin Perk 2025	55.17	5.87	50	58.51	7.55	62	37.6%	-3.34 [-5.83, -0.85]
Nacaroglu 2023	55.63	6.14	49	59.2	7.66	49	0.0%	-3.57 [-6.32, -0.82]
Yakut 2025	54.29	5.61	35	57	6.81	35	27.2%	-2.71 [-5.63, 0.21]
**Total (95% CI)**			**139**			**151**	**100.0%**	**-3.70 [-5.22, -2.17]**

Heterogeneity: Tau² = 0.00; Chi² = 1.37, df = 3 (P = 0.71); I² = 0%
Test for overall effect: Z = 4.75 (P < 0.00001)

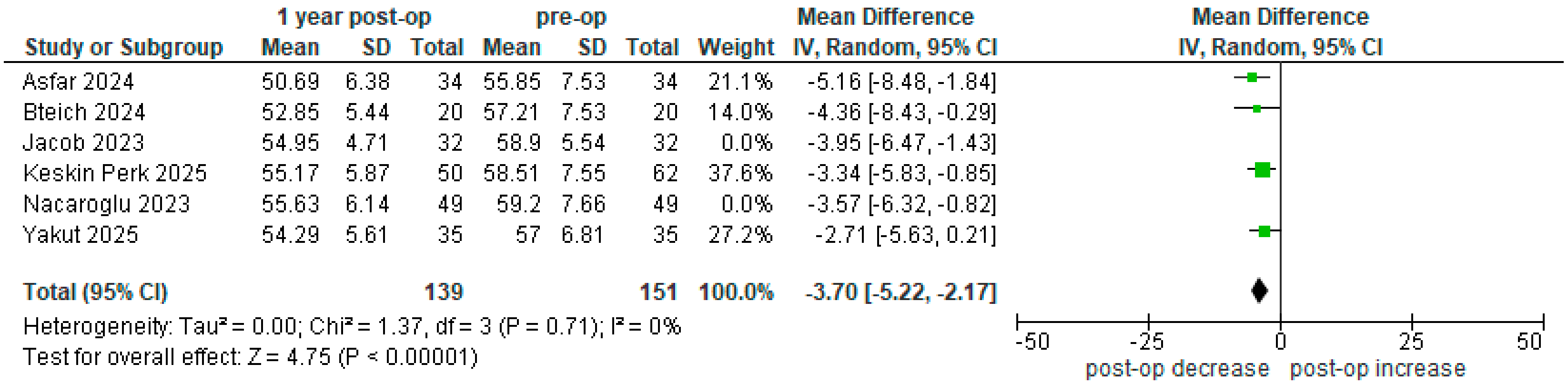

S1.4.3.6. Difference in mean simulated keratometry

Figure S1.4.3.6A. Difference in mean simulated keratometry across 2 studies [6, 17] - CXL

	1 year post-op			pre-op				Mean Difference
**Study or Subgroup**	**Mean**	**SD**	**Total**	**Mean**	**SD**	**Total**	**Weight**	**IV, Random, 95% CI**
Asfar 2024	44.25	3.29	34	47.52	3.82	34	0.0%	-3.27 [-4.96, -1.58]
Bteich 2024	45.23	2.83	20	49.3	4.83	20	0.0%	-4.07 [-6.52, -1.62]
Jacob 2023	45.63	2.91	32	49.07	3.06	32	65.8%	-3.44 [-4.90, -1.98]
Keskin Perk 2025	45.63	4.44	50	49.17	4.97	62	0.0%	-3.54 [-5.28, -1.80]
Nacaroglu 2023	45.39	4.92	49	49.34	5.32	49	34.2%	-3.95 [-5.98, -1.92]
Yakut 2025	44.5	4.42	35	48.26	4.78	35	0.0%	-3.76 [-5.92, -1.60]
**Total (95% CI)**			**81**			**81**	**100.0%**	**-3.61 [-4.80, -2.43]**

Heterogeneity: Tau² = 0.00; Chi² = 0.16, df = 1 (P = 0.69); I² = 0%
Test for overall effect: Z = 5.97 (P < 0.00001)

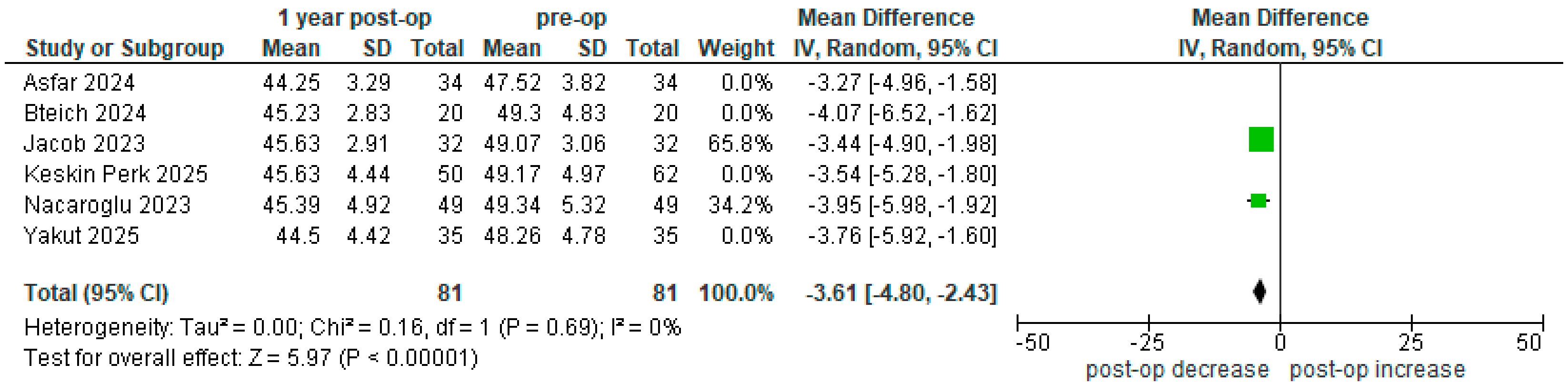

Figure S1.4.3.6B. Difference in mean simulated keratometry across 4 studies [2, 4, 15, 18] – no CXL

	1 year post-op			pre-op				Mean Difference
**Study or Subgroup**	**Mean**	**SD**	**Total**	**Mean**	**SD**	**Total**	**Weight**	**IV, Random, 95% CI**
Asfar 2024	44.25	3.29	34	47.52	3.82	34	32.9%	-3.27 [-4.96, -1.58]
Bteich 2024	45.23	2.83	20	49.3	4.83	20	15.7%	-4.07 [-6.52, -1.62]
Jacob 2023	45.63	2.91	32	49.07	3.06	32	0.0%	-3.44 [-4.90, -1.98]
Keskin Perk 2025	45.63	4.44	50	49.17	4.97	62	31.0%	-3.54 [-5.28, -1.80]
Nacaroglu 2023	45.39	4.92	49	49.34	5.32	49	0.0%	-3.95 [-5.98, -1.92]
Yakut 2025	44.5	4.42	35	48.26	4.78	35	20.3%	-3.76 [-5.92, -1.60]
**Total (95% CI)**			**139**			**151**	**100.0%**	**-3.58 [-4.55, -2.61]**

Heterogeneity: Tau² = 0.00; Chi² = 0.31, df = 3 (P = 0.96); I² = 0%
Test for overall effect: Z = 7.21 (P < 0.00001)

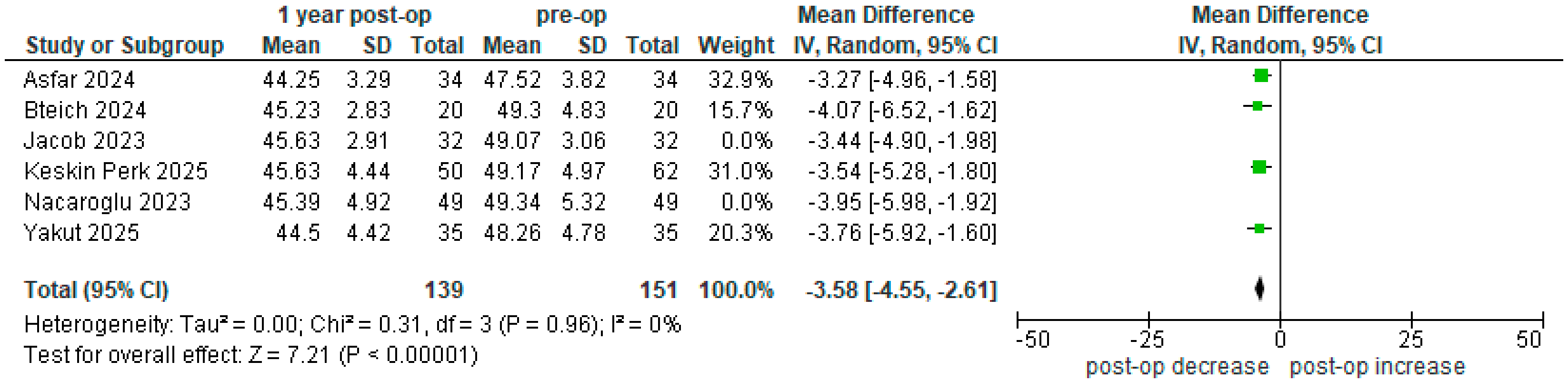

S1.4.3.7. Difference in total higher order aberrations

Figure S1.4.3.7A. Difference in total higher order aberrations across 0 studies – CXL

Figure S1.4.3.7B. Difference in total higher order aberrations across 2 studies [2, 15] – no CXL

	1 year post-op			pre-op				Mean Difference
**Study or Subgroup**	**Mean**	**SD**	**Total**	**Mean**	**SD**	**Total**	**Weight**	**IV, Random, 95% CI**
Asfar 2024	1.54	0.93	34	2.07	1.05	34	53.4%	-0.53 [-1.00, -0.06]
Bteich 2024	1.36	0.65	20	1.92	0.95	20	46.6%	-0.56 [-1.06, -0.06]
**Total (95% CI)**			**54**			**54**	**100.0%**	**-0.54 [-0.89, -0.20]**

Heterogeneity: Tau² = 0.00; Chi² = 0.01, df = 1 (P = 0.93); I² = 0%
Test for overall effect: Z = 3.10 (P = 0.002)

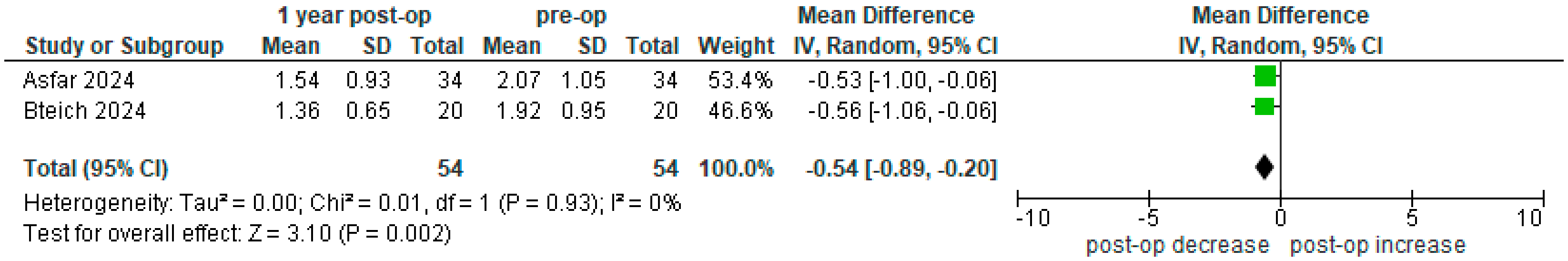

S1.4.3.8. Difference in spherical aberration

S1.4.3.8A. Difference in spherical aberration across 0 studies – CXL

Figure S1.4.3.8B. Difference in spherical aberration across 2 studies [2, 15]– no CXL

	1 year post-op			pre-op				Mean Difference
Study or Subgroup	Mean	SD	Total	Mean	SD	Total	Weight	IV, Random, 95% CI
Asfar 2024	0.18	0.5	34	0.07	0.42	34	60.7%	0.11 [-0.11, 0.33]
Bteich 2024	0.15	0.46	20	0.05	0.42	20	39.3%	0.10 [-0.17, 0.37]
**Total (95% CI)**			**54**			**54**	**100.0%**	**0.11 [-0.06, 0.28]**

Heterogeneity: Tau² = 0.00; Chi² = 0.00, df = 1 (P = 0.96); I² = 0%
Test for overall effect: Z = 1.22 (P = 0.22)

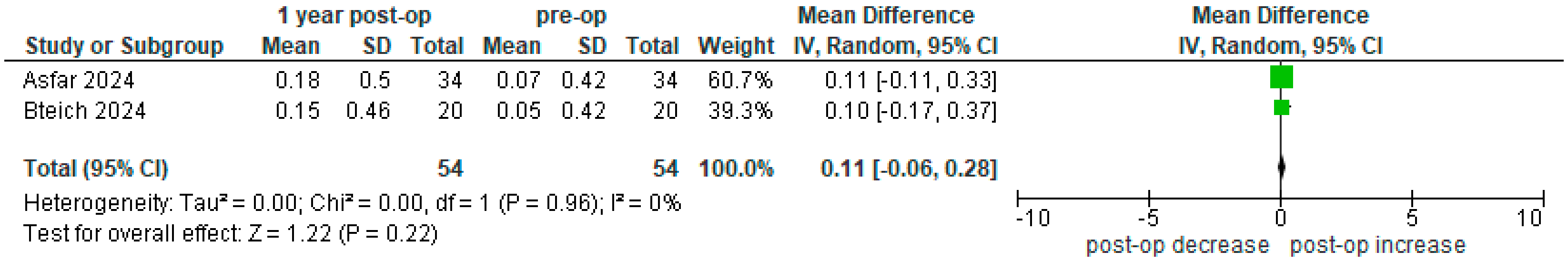

S1.4.3.9. Difference in vertical coma

Figure S1.4.3.9A. Difference in vertical coma in one study [17]– CXL

	1 year post-op			pre-op				Mean Difference
**Study or Subgroup**	**Mean**	**SD**	**Total**	**Mean**	**SD**	**Total**	**Weight**	**IV, Random, 95% CI**
Asfar 2024	0.38	0.65	34	1.49	1.02	34	0.0%	-1.11 [-1.52, -0.70]
Bteich 2024	0.35	0.97	20	1.44	1.09	20	0.0%	-1.09 [-1.73, -0.45]
Jacob 2023	-1.4	1.21	32	-2.09	0.96	32	100.0%	0.69 [0.15, 1.23]
**Total (95% CI)**			**32**			**32**	**100.0%**	**0.69 [0.15, 1.23]**

Heterogeneity: Not applicable
Test for overall effect: Z = 2.53 (P = 0.01)

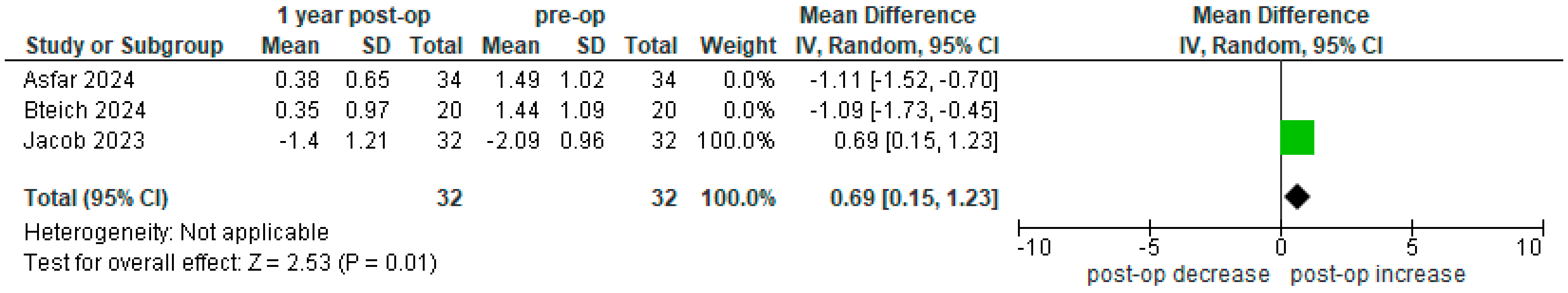

Figure S1.4.3.9B. Difference in vertical coma across 2 studies [2, 15] – no CXL

	1 year post-op			pre-op				Mean Difference
**Study or Subgroup**	**Mean**	**SD**	**Total**	**Mean**	**SD**	**Total**	**Weight**	**IV, Random, 95% CI**
Asfar 2024	0.38	0.65	34	1.49	1.02	34	71.2%	-1.11 [-1.52, -0.70]
Bteich 2024	0.35	0.97	20	1.44	1.09	20	28.8%	-1.09 [-1.73, -0.45]
Jacob 2023	-1.4	1.21	32	-2.09	0.96	32	0.0%	0.69 [0.15, 1.23]
**Total (95% CI)**			**54**			**54**	**100.0%**	**-1.10 [-1.45, -0.76]**

Heterogeneity: Tau² = 0.00; Chi² = 0.00, df = 1 (P = 0.96); I² = 0%
Test for overall effect: Z = 6.31 (P < 0.00001)

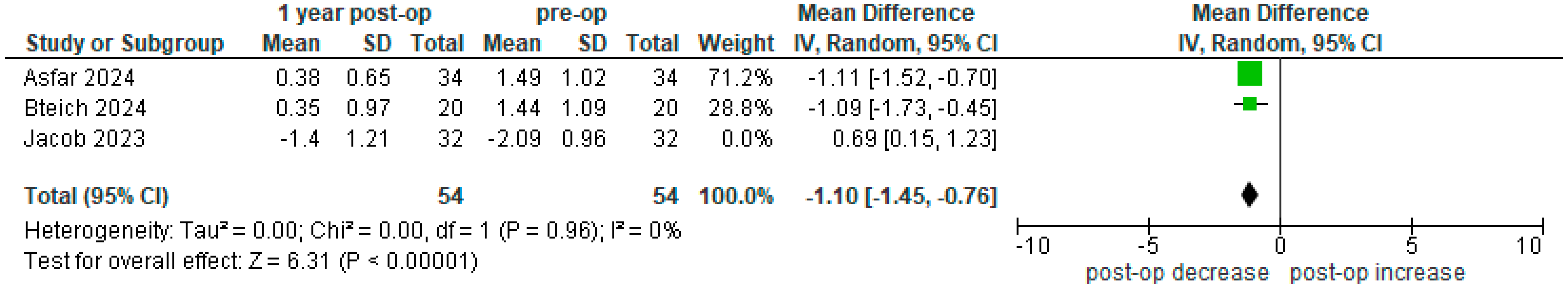

S1.4.3.10. Difference in trefoil

S1.4.3.10A. Difference in trefoil across 0 studies – CXL

Figure S1.4.3.10B. Difference in trefoil across 2 studies [2, 15] – no CXL

	1 year post-op			pre-op				Mean Difference
**Study or Subgroup**	**Mean**	**SD**	**Total**	**Mean**	**SD**	**Total**	**Weight**	**IV, Random, 95% CI**
Asfar 2024	-0.17	0.63	34	-0.37	0.59	34	53.7%	0.20 [-0.09, 0.49]
Bteich 2024	0.007	0.59	20	-0.27	0.4	20	46.3%	0.28 [-0.04, 0.59]
**Total (95% CI)**			**54**			**54**	**100.0%**	**0.24 [0.02, 0.45]**

Heterogeneity: Tau² = 0.00; Chi² = 0.13, df = 1 (P = 0.72); I² = 0%
Test for overall effect: Z = 2.17 (P = 0.03)

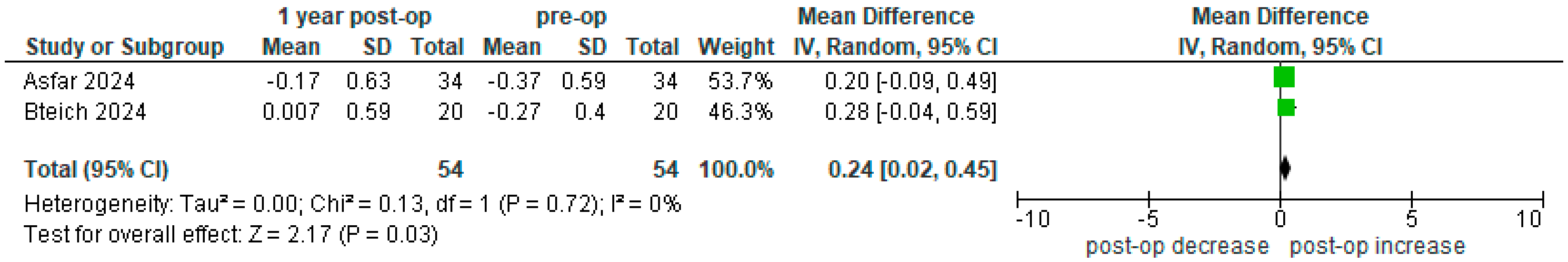

S1.4.3.11. Difference in total RMS

Figure S1.4.3.11A. Difference in total RMS in 1 study [17] – CXL

	1 year post-op			pre-op				Mean Difference
**Study or Subgroup**	**Mean**	**SD**	**Total**	**Mean**	**SD**	**Total**	**Weight**	**IV, Random, 95% CI**
Jacob 2023	2.59	0.8	32	2.85	0.78	32	100.0%	-0.26 [-0.65, 0.13]
**Total (95% CI)**			**32**			**32**	**100.0%**	**-0.26 [-0.65, 0.13]**
Heterogeneity: Not applicable								
Test for overall effect: Z = 1.32 (P = 0.19)								

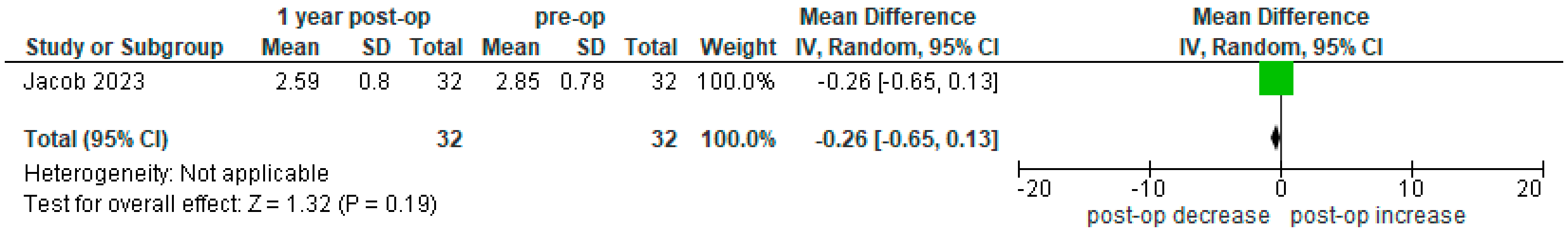

Figure S1.4.3.11B. Difference in total RMS across 0 studies – no CXL

S1.4.4. Welch's t test

Table S1.4.4.1. Segment preparation: trephined blade vs femtosecond laser

	1 month	6 months	1 year
Trephine BCVA (logMAR)	-0,37*	-0,39*	-0,32*
Laser BCVA (logMAR)	-0,3*	-0,29*	-0,42*
t test p-value	0,426	0,256	0,334
Trephine Maximum keratometry (D)	-3,56*	-3,96*	-3,67*
Laser Maximum keratometry (D)	-4,34*	-2,66*	-4,36*
t test p-value	0,768	0,56	0,739

Table S1.4.4.2. Segment preparation: non-dehydrated vs dehydrated

	1 month	6 months	1 year
Non-dehydrated BCVA (logMAR)	-	-0,13	-0,09*
Dehydrated BCVA (logMAR)	-	-0,33*	-0,39*
t test p-value		0,038*	9E-09**
Fresh Maximum keratometry (D)	-	-0,67	-3,95*
Non-dehydrated Maximum keratometry (D)	-	-5,03*	-4,84*
t test p-value		0,176	0,618

Table S1.4.4.3. Post-operative corneal cross-linking vs no cross-linking

	1 month	6 months	1 year
No cross-linking BCVA (logMAR)	-0,32*	-0,33*	-0,41*
Cross-linking BCVA (logMAR)	-0,45*	-0,43*	-0,24
t test p-value	0,414	0,482	0,224
No cross-linking Maximum keratometry (D)	-4,36*	-3,68*	-3,7*
Cross-linking Maximum keratometry (D)	-2,21	-3,31*	-3,78*
t test p-value	0,283	0,858	0,947

Section S2 – Characteristics of included studies

Asfar 2024

Methods	Type of study: Interventional study<br>Study design: Retrospective cohort study
Participants	Country: Lebanon<br>Number of individuals with keratoconus: 31<br>Number of eyes: 34<br>Subgroups: 34<br>Inclusion criteria: individuals with a need for reduced spectacle correction and an intolerance to contact lenses who had unsatisfactory visuals results with spectacles and were reluctant to have or intolerant to rigid gas-permeable lenses, with no history of corneal cross-linking or customized photorefractive keratectomy<br>Exclusion criteria: the presence of corneal scarring, a history of prior corneal surgeries; systemic autoimmune ailments, a history of herpetic keratitis; and patients with a follow-up period of fewer than 3 months
Interventions	CAIRS with trephine and without concomitant corneal crosslinking<br><br>Donor tissue:<br>- levy: trimming<br>- cutout: 500- or 750-μm double-bladed Jacob's trephine<br>- dehydration<br>Surgical technique:<br>- incision: femtosecond laser<br>- implantation depth: 250 to 300 um<br>- tunnel parameters: .8-mm inner diameter and 7.6-mm outer diameter, resulting in a tunnel width of 900 μm<br>- cross-linking: no<br>Postoperative medication: not specified
Outcomes	UDVA (logMAR), CDVA (logMAR), cylinder (D), MRSE (D), Kmax (D), Kmean (D), keratometric astigmatism K2-K1, vertical coma, horizontal coma, spherical aberration; trefoil [3, -3], trefoil [3, 3], total coma, total HOA, largest stromal elevation, thinnest epithelium over the cone; mean epithelial thickness in 1 mm central to the ring measured preoperatively, 3 months postoperatively and last follow-up

Quality assessment

**Item**	**Authors' judgement**
Consecutive patients?	Unclear
Reasons for inclusion reported?	Yes
Reasons for exclusion reported?	Yes
Were point estimates and measures of variability presented for the outcome measures?	Yes
Was calculation of statistical power reported?	Yes

Risk of bias

**Item**	**Authors' judgement**
Data collector blinded?	No
Outcome assessor blinded?	No
Free of selective reporting?	Unclear
Free of other bias?	No

Bteich 2023 Therapeutic Refractive Surgery

Methods	Type of study: Interventional study<br>Study design: Retrospective case series
Participants	Country: Lebanon<br>Number of individuals with keratoconus: 2<br>Number of eyes: 4<br>Subgroups: -<br>Inclusion criteria: irregular keratoconus<br>Exclusion criteria: a history of intraocular surgeries or autoimmune/connective tissue diseases
Interventions	CAIRS with femtosecond laser and without concomitant corneal crosslinking<br><br>Donor tissue:<br>- levy: trimming<br>- cutout: double bladed trephine blade<br>- soaking: dyeing / sterilization

	- dehydration under 35% to 45% average humidity conditions<br>Surgical technique:<br>- incision: femtosecond laser 260 to 275 um<br>- implantation depth<br>- tunnel parameters: inner diameter 6 mm, outer diameter 7.8 mm<br>- cross-linking<br>Postoperative medication: moxifloxacin 4x/day for 1 week, prednisolone acetate 4x/day for 1 week then tapered gradually over 1 month, and sodium hyaluronate eye drops as needed
Outcomes	UDVA (logMAR), CDVA (logMAR), manifest sphere (D), manifest cylinder (D) x axis, Kmean (D), Kmax (D), spherical aberration, coma, aberration astigmatism measured preoperatively, and at 1, 3 and 6 months postoperatively

Quality assessment

**Item**	**Authors' judgement**
Consecutive patients?	Unclear
Reasons for inclusion reported?	Yes
Reasons for exclusion reported?	Unclear
Were point estimates and measures of variability presented for the outcome measures?	Yes
Was calculation of statistical power reported?	No

Risk of bias

**Item**	**Authors' judgement**
Data collector blinded?	No
Outcome assessor blinded?	No
Free of selective reporting?	No, only the preoperative measurements and 6 months postoperatively are presented, but not the measurements at 1 and 3 months postoperatively
Free of other bias?	No, small sample size

Bteich 2023

Methods	Type of study: Interventional study<br>Study design: Retrospective case series
Participants	Country: Lebanon<br>Number of individuals with keratoconus / corneal ectasia after laser vision correction: 39<br>Number of treated eyes: 52 eyes (47 eyes with keratoconus and 5 eyes with corneal ectasia after laser vision correction)<br>Subgroups: -<br>Inclusion criteria: patients with keratoconus or corneal ectasia after laser vision correction who had unsatisfactory visual results with spectacles and were reluctant to have or intolerant to rigid gas-permeable lenses<br>Exclusion criteria: corneal opacity, previous intraocular surgeries; viral keratitis; autoimmune and connective tissue diseases, follow-up period of < 3 months
Interventions	CAIRS with femtosecond laser and without concomitant corneal crosslinking<br><br>Donor tissue:<br>- levy: trimming: 500 or 750 µm ring of stromal tissue, which has been dissected into two equal semicircular segments<br>- cutout: laser<br>- soaking: dyeing / sterilization<br>- dehydration / rehydration: dehydration for 45 min in a room with 35% or 45% humidity<br>Surgical technique:<br>- incision: laser<br>- implantation depth: ranging from 250 to 300 µm. For patients with ectasia after laser vision correction, the flap depth was determined on OCT at a diameter of 6 to 8 mm<br>- tunnel parameters:<br>*depth: the target tunnel depth was at least deeper than the deepest point of the flap interface plus three times the standard deviation of the laser tunnel depth (7 µm), i.e., 23, with an additional safety margin of 50 µm, ensuring a low chance of intersection with the flap<br>*position: 5/6 mm inner diameter and 6.8/7.8 mm outer diameter, 900 µm tunnel width<br>- cross-linking<br>Postoperative medication: moxifloxacin 4x/day for 1 week, pred forte 4x/day for 1week then tapered over 1 month, and sodium hyaluronate eye drops as needed for lubrication
Outcomes	UDVA (logMAR), CDVA (logMAR), cylinder (D), MRSE (D), Jackson cross cylinder power at axis 90° and 180°, Jackson cross cylinder power at axis 45° and 135°, blur strength assessed at 1 week, 1 month and 3 months postoperatively

Quality assessment

**Item**	**Authors' judgement**
Consecutive patients?	Unclear
Reasons for inclusion reported?	Yes
Reasons for exclusion reported?	Yes
Were point estimates and measures of variability presented for the outcome measures?	Yes
Was calculation of statistical power reported?	No

Risk of bias

**Item**	**Authors' judgement**
Data collector blinded?	No
Outcome assessor blinded?	No
Free of selective reporting?	Unclear
Free of other bias?	No, small number of patients

Bteich 2025

Methods	Type of study: Interventional study<br>Study design: Retrospective case series
Participants	Country: Lebanon<br>Number of individuals with keratoconus: 15<br>Number of eyes: 20<br>Subgroups: -<br>Inclusion criteria: stable keratoconus preoperatively, patients with inadequate visual results with spectacles or were intolerant to rigid-gas permeable lenses<br>Exclusion criteria: corneal opacity, previous intraocular surgeries, viral keratitis; autoimmune and connective tissue diseases, follow-up period < 3 months
Interventions	Femto-CAIRS without concomitant corneal crosslinking<br><br>Donor tissue:<br>- levy: circular trimming: outer diameter of a segment can vary up to 9 mm while the internal diameter would vary up to 8 mm for segments of 500 mm size and up to 7.5 mm for the 750 mm segments<br>- cutout: multiple / laser / punch + size<br>- soaking: dyeing / sterilization<br>- dehydration / rehydration: dehydration<br>Surgical technique:<br>- incision: blade / laser: femtosecond laser<br>- implantation depth:<br>- tunnel parameters:<br>*depth: range from 250 to 300 mm and 700 to 900 mm width<br>*position: the optical zone was 6.0 mm<br>- cross-linking: no<br>Postoperative medication: fourth-generation fluoroquinolones 4 times daily for 1 week, prednisolone acetate 4 times daily for 1 week and then tapered over 1 month, and sodium hyaluronate eye drops as needed for lubrication
Outcomes	UDVA (logMAR), CDVA (logMAR), cylinder (D), MRSE (D) assessed preoperatively, 1 week, 1 month, 3 months, 6 months, and 12 months postoperatively

Quality assessment

**Item**	**Authors' judgement**
Consecutive patients?	Unclear
Reasons for inclusion reported?	Yes

Reasons for exclusion reported?	Yes
Were point estimates and measures of variability presented for the outcome measures?	Yes
Was calculation of statistical power reported?	No

Risk of bias

**Item**	**Authors' judgement**
Data collector blinded?	No
Outcome assessor blinded?	No
Free of selective reporting?	Unclear
Free of other bias?	No

Colak 2025

Methods	Type of study: Interventional study<br>Study design: Retrospective case series
Participants	Country: Turkey<br>Number of individuals with keratoconus: 5<br>Number of eyes: 6<br>Subgroups: -<br>Inclusion criteria: central cones (nipple shaped or bowtie shaped), intolerance to contact lenses, clear central cornea, no previous history of ocular surgery; minimal corneal thickness of at least 350 µm<br>Exclusion criteria: -
Interventions	CAIRS by KeraNatural without concomitant corneal crosslinking<br><br>Donor tissue:<br>- levy: KeraNatural<br>- cutout: each segment was manually cut and shaped intraoperativelyto align with the specific topographic characteristics of the patient's cornea<br>- soaking: sterile balanced salt solution<br>- hydration<br>Surgical technique:<br>- incision: femtosecond laser (iFS)<br>- implantation depth: 200 to 250 mm<br>- tunnel parameters: inner and outer diameters were 4 and 7.5 µm<br>- cross-linking: no<br>Postoperative medication: -
Outcomes	UDVA (logMAR), CDVA (LogMAR), manifest sphere (D), manifest astigmatism (D), K1 (D), K2 (D), Kmean (D), Kmax (D), central corneal thickness (µm) measured preoperatively and postoperatively

Quality assessment

**Item**	**Authors' judgement**
Consecutive patients?	Unclear
Reasons for inclusion reported?	Yes
Reasons for exclusion reported?	Yes
Were point estimates and measures of variability presented for the outcome measures?	No

Was calculation of statistical power reported?	No

Risk of bias

**Item**	**Authors' judgement**
Data collector blinded?	No
Outcome assessor blinded?	No
Free of selective reporting?	Unclear
Free of other bias?	No

Coscarelli 2024

Methods	Type of study: Interventional study<br>Study design: Single-arm prospective cohort study
Participants	Country: Brazil<br>Number of individuals with keratoconus: 3<br>Number of eyes: 3<br>Subgroups: -<br>Inclusion criteria: (1) keratoconus diagnosis and (2) central clear cornea, with no scar/striae<br>Exclusion criteria: (1) previous corneal or intraocular surgical procedures, (2) local or systemic infections present at the time of the surgery, (3) corneal scars, and (4) any other vision-limiting disorders besides keratoconus
Interventions	Donor tissue:<br>- levy: double-bladed trephine<br>- cutout: donut-shaped 5.4/8 mm<br>- soaking: dyeing / sterilization<br>- dehydration / rehydration: left outside the storage preservation media for 2 hours to ensure dehydration and stiffening of the tissue<br>Surgical technique:<br>- incision: blade / laser: laser<br>- implantation depth -<br>- tunnel parameters: depth set at 50% of the thinnest corneal thickness point on the desired location<br>- cross-linking: no<br>Postoperative medication: moxifloxacin 0.5% with dexamethasone 0.1% 4 times a day for 7 days and then tapered for 60 days, artificial eye drops were used 2 to 4 times daily for at least 30 days
Outcomes	UCVA, BSCVA, K1, K2, refraction

Quality assessment

**Item**	**Authors' judgement**
Consecutive patients?	Unclear
Reasons for inclusion reported?	Yes
Reasons for exclusion reported?	Yes
Were point estimates and measures of variability presented for the outcome measures?	No, but the outcomes of each case are reported separately
Was calculation of statistical power reported?	No

Risk of bias

**Item**	**Authors' judgement**
Data collector blinded?	No
Outcome assessor blinded?	No
Free of selective reporting?	Unclear
Free of other bias?	No, small number of patients

Haciagaoglu 2022

Methods	Type of study: Interventional study<br>Study design: Retrospective case series
Participants	Country: Turkey<br>Number of individuals with keratoconus: 32<br>Number of eyes: 44<br>Subgroups: -<br>Inclusion criteria: age ≥ 20 years, contact lens intolerance, a clear cornea, and minimal corneal thickness of at least 350 um at the thinnest location<br>Exclusion criteria: a history of corneal/intraocular surgery, central or paracentral scarring, previous viral keratitis, connective tissue diseases, and pregnancy or lactation during the study
Interventions	Donor tissue:<br>- levy: the inner and outer diameters of the tunnel were 4 mm and 7.5 mm, respectively<br>- cutout: femtosecond laser<br>- soaking: -<br>- dehydration / rehydration: not specified<br>Surgical technique:<br>- incision: blade / laser: laser<br>- implantation depth -<br>- tunnel parameters: depth 200 um<br>- cross-linking: no<br>Postoperative medication: topical moxifloxacin 0.5% four times a day for one week and preservative-free artificial tears were administered five times a day for four weeks postoperatively. Topical dexamethasone 0.1% was used five times daily for four weeks, then tapered and discontinued at an average of 6 weeks
Outcomes	UDVA, CDVA, spherical equivalent, flat keratometry, steep keratometry, mean keratometry, maximum keratometry, pachymetry of thinnest point

Quality assessment

**Item**	**Authors' judgement**
Consecutive patients?	Unclear
Reasons for inclusion reported?	Yes
Reasons for exclusion reported?	Yes

Were point estimates and measures of variability presented for the outcome measures?	Yes
Was calculation of statistical power reported?	No

Risk of bias

**Item**	**Authors' judgement**
Data collector blinded?	No
Outcome assessor blinded?	No
Free of selective reporting?	Unclear
Free of other bias?	No

Hayashi 2025

Methods	Type of study: Interventional study<br>Study design: Prospective case series
Participants	Country: Japan<br>Number of individuals with keratoconus: 5<br>Number of eyes: 5<br>Subgroups: -<br>Inclusion criteria: keratoconus treated with manual CAIRS<br>Exclusion criteria: not specified
Interventions	Donor tissue:<br>- levy: punched<br>- cutout: Jacobs CAIRS trephine<br>- soaking: 0.1% Brilliant Blue G dye<br>- hydration / dehydration NR<br>Surgical technique:<br>- incision: Micro Feather Knife<br>- implantation depth: 200 µm<br>- tunnel parameters: inner diameter of 5.0 mm and a width of about 3 mm<br>- cross-linking: no<br>Postoperative medication: gatifloxacin hydrate 0.3% ophthalmic solution (Gatiflo; Senju Pharmaceutical Co. Ltd., Osaka, Japan) 4 times daily for 2 weeks, betamethasone (Sanbetason; Santen, Osaka, Japan) 4 times daily for 2 weeks, and 2% rebamipide ophthalmic solution (Mucosta; Otsuka, Tokyo, Japan) 4 times daily for 4 weeks, then tapered or stopped. Finally, a weak steroid (0.1% fluorometholone [Senjyu Pharmaceutical, Osaka, Japan]) was administered twice per day for 1 month after surgery

Outcomes	BCVA (logMAR), UDCA (logMAR), refraction (D), cyl (D), SE (D), Kmax (D), anterior Ks (D), anterior Kf (D), posterior Ks (D), posterior Kf (D), CCT (μm), total HOAs (μm)

Quality assessment

**Item**	**Authors' judgement**
Consecutive patients?	Unclear
Reasons for inclusion reported?	Yes
Reasons for exclusion reported?	No
Were point estimates and measures of variability presented for the outcome measures?	Yes
Was calculation of statistical power reported?	Yes

Risk of bias

**Item**	**Authors' judgement**
Data collector blinded?	No
Outcome assessor blinded?	No
Free of selective reporting?	Unclear
Free of other bias?	No

Jacob 2018

Methods	Type of study: Interventional study<br>Study design: Prospective case series
Participants	Country: India<br>Number of individuals with keratoconus: 29<br>Number of eyes: 24<br>Subgroups: CAIRS followed by accelerated corneal cross-linking (A-CXL), either conventional or contact lens-assisted CXL (A-CACXL)<br>Inclusion criteria: Amsler-Krumeich stages 1-4 showing progression and with sufficient minimum corneal thickness to allow A-CXL or A-CACXL (progression defined as an increase in simulated

	maximum keratometry or steepest keratometry values of greater than 0.75D in the preceding 6 months.<br>Exclusion criteria: patients > 35 years and those with severe allergies, autoimmune and immunodeficiency syndromes, previous viral keratitis, corneas steeper than 68D, central or paracentral scarring; corneas too thin (< 320 um minimum corneal thickness) to allow A-CAXL, and history of prior corneal/intraocular surgery.
Interventions	Donor tissue:<br>- levy: trimming: circular tissue<br>- cutout: multiple / laser / punch + size: double bladed trephine (outer diameter of 7.5 mm, inner diameter of 6.7 mm), bisected into two equal halves<br>- soaking: dyeing / sterilization<br>- dehydration / rehydration: dehydrated<br>Surgical technique:<br>- incision: blade / laser: femtosecond laser<br>- tunnel parameters:<br>*depth: depth was programmed to be at 50% of the minimum pachymetry in the 7 mm optical zone<br>*position: inner diameter of 6.5 mm and outer diameter of 8 mm<br>- cross-linking (CXL) or conventional or contact lens-assisted CXL (A-CACXL)<br>Postoperative medication: topical ofloxacin with dexamethasone combination eye drops 6x/day for 2 weeks and then tapered to stop over the next 3 weeks. A therapeutic bandage contact lens was used until complete epithelial healing. Tear supplements and UV protective glasses for 6 months after surgery.
Outcomes	UDVA, CDVA, spherical equivalent, refractive astigmatism, topographic astigmatism, maximum keratometry, steepest keratometry, anterior best fit sphere, posterior best fit sphere; mean power 3-mm zone; mean power 5-mm zone, AS-OCT pachymetry preoperatively and at last visit

Quality assessment

**Item**	**Authors' judgement**
Consecutive patients?	Unclear
Reasons for inclusion reported?	Yes
Reasons for exclusion reported?	Yes
Were point estimates and measures of variability presented for the outcome measures?	Yes
Was calculation of statistical power reported?	No

Risk of bias

**Item**	**Authors' judgement**
Data collector blinded?	No
Outcome assessor blinded?	No
Free of selective reporting?	Unclear
Free of other bias?	No

Jacob 2023

Methods	Type of study: Interventional study<br>Study design: Prospective case series
Participants	Country: India<br>Number of individuals with keratoconus: 29<br>Number of eyes: 32<br>Subgroups: -<br>Inclusion criteria: Amsler-Krumeich stages 1-4 with pericentral or paracentral decentered cones that showed gradation of keratometry values, with one side being steeper than the other<br>Exclusion criteria: severe allergies, autoimmune and immunodeficiency syndromes, previous viral keratitis, central or paracentral scaring, corneal thickness < 320 µm, history of prior corneal/intraocular surgery
Interventions	Donor tissue:<br>- levy: trimming: circular tissue<br>- cutout: multiple / laser / punch + size: double bladed trephine (outer diameter of 7.5/8.75 mm, inner diameter of 6.5/8 mm)<br>- soaking: dyeing / sterilization<br>- dehydration / rehydration: dehydrated<br>Surgical technique:<br>- incision: blade / laser: femtosecond laser<br>- implantation depth: 50% depth of the minimum pachymetry in the zone of implantation up to a maximum depth of 280 µm<br>- tunnel parameters:<br>*depth: depth was programmed to be at 50% of the minimum stromal thickness<br>*position: inner diameter of 4.6 mm and tunnel width of approximately 1.5 mm<br>- cross-linking<br>Postoperative medication: dexamethasone eyedrops 6x/day for 2 weeks; tapered and stopped over the next 2 weeks. Antibiotic eye drops were applied for the initial 2 weeks.
Outcomes	UDVA, SCDVA, sphere, cylinder, spherical equivalent, K1 front, K2 front, Km front, astigmatism front, Q-value front, Kmax, thinnest pachymetry, RMS HOA, vertical coma, horizontal coma, AS-OCT segment width at 1 month and at last visit, AS-OCT segment height at 1 month and at last visit

Quality assessment

**Item**	**Authors' judgement**
Consecutive patients?	Unclear

Reasons for inclusion reported?	Yes
Reasons for exclusion reported?	Yes
Were point estimates and measures of variability presented for the outcome measures?	Yes
Was calculation of statistical power reported?	No

Risk of bias

**Item**	**Authors' judgement**
Data collector blinded?	No
Outcome assessor blinded?	No
Free of selective reporting?	Unclear
Free of other bias?	No

Keskin Perk 2025

Methods	Type of study: Interventional study<br>Study design: Prospective case series
Participants	Country: Turkey<br>Number of individuals with keratoconus: 49<br>Number of eyes: 62<br>Subgroups: -<br>Inclusion criteria: age > 18 years old, progressive keratoconus; Amsler-Krumeich stages 1-3, CDVA < 0.3 logMAR and contact lens intolerance, corneal thickness > 350 µm, absence of additional ocular diseases other than keratoconus<br>Exclusion criteria: central or paracentral scaring, history of viral keratitis, severe dry eye, history of prior corneal/intraocular surgery, autoimmune and connective tissue diseases and pregnant or lactating patients, patients who had previously undergone crosslinking treatment or who had a double-ring segment implanted
Interventions	Donor tissue:<br>- levy: trimming: prepared CAIRSs by KeraNatural<br>- cutout: multiple / laser / punch + size: arc length of approximately 160°<br>- soaking: dyeing / sterilization<br>- dehydration / rehydration<br>Surgical technique:<br>- incision: blade / laser<br>- implantation depth: the tunnel depth ranged from 200 to 250 µm (approximately 35-40% depth)<br>- tunnel parameters:<br>*depth<br>*position: inner diameter of 4 mm and an outer diameter of 7.5 mm<br>- cross-linking<br>Postoperative medication:
Outcomes	UDVA (logMAR), CDVA (logMAR), SE (D), SR (D), CR (D), K1 (D), K2 (D), Kmean (D), Kmax (D), Pachymetry (um)

Quality assessment

**Item**	**Authors' judgement**
Consecutive patients?	Unclear
Reasons for inclusion reported?	Yes
Reasons for exclusion reported?	Yes

Were point estimates and measures of variability presented for the outcome measures?	Yes
Was calculation of statistical power reported?	No

Risk of bias

**Item**	**Authors' judgement**
Data collector blinded?	No
Outcome assessor blinded?	No
Free of selective reporting?	Unclear
Free of other bias?	No

Kirgiz 2024

Methods	Type of study: Interventional study<br>Study design: Retrospective case series
Participants	Country: Turkey<br>Number of individuals with keratoconus: 23<br>Number of eyes with keratoconus: 23<br>Subgroups: -<br>Inclusion criteria: age > 18 years old; keratoconus, unable to achieve a satisfactory level of visual acuity with either spectacles or contact lenses, corneal thickness > 350 um, contact lens intolerance, no progression of keratoconus for at least 1 year of follow-up; and at least 1 year of follow-up after corneal cross-linking (if performed), CAIRSs implantation without previous CXL was performed in patients over 35 years of age with no progression of keratoconus during at least 2 years of follow-up<br>Exclusion criteria: history of prior corneal/intraocular surgery; corneal scarring, previous hydrops; active severe allergy or allergic conjunctivitis, breastfeeding or pregnancy; at least 12 months after CXL
Interventions	Donor tissue:<br>- levy: punch<br>- cutout: 2 mm wide 360 degrees ring-shaped, then split with a 45 blade to create two CAIRSs<br>- soaking: -<br>- dehydration / rehydration: dehydration for 20 min<br>Surgical technique:<br>- incision: Visumax 500 kHz femtosecond laser<br>- implantation depth: 200 µm<br>- tunnel parameters:<br>*depth<br>*position: inner diameter to 4.50 mm and outer diameter to 7.75 mm<br>- cross-linking<br>Postoperative medication: moxifloxacin drops 4x/day for 1 week, prednisolone acetate drops 4x/day, switch to loteprednol etabonate drops 2 weeks later, and frequency of topical steroid use was discontinued after 1 month
Outcomes	UCVA (Snellen), BCVA (Snellen), SE (D), K1 (D), K2 (D), Kmean (D), Kmax (D), CT (u), ECD (cells/mm2)

Quality assessment

Item	Authors' judgement
Consecutive patients?	Unclear

Reasons for inclusion reported?	Yes
Reasons for exclusion reported?	Yes
Were point estimates and measures of variability presented for the outcome measures?	Yes
Was calculation of statistical power reported?	No

Risk of bias

**Item**	**Authors' judgement**
Data collector blinded?	No
Outcome assessor blinded?	No
Free of selective reporting?	Unclear
Free of other bias?	No

Mazzota 2024

Methods	Type of study: Interventional study<br>Study design: Prospective case series
Participants	Country: Italy<br>Number of individuals with keratoconus: xxx<br>Number of eyes: 2/2<br>Subgroups: -<br>Inclusion criteria: -<br>Exclusion criteria: -
Interventions	Donor tissue:<br>- levy: all-femtosecond laser-cut procedure<br>- cutout: one segment<br>- soaking: -<br>- hydration / dehydration dehydration<br>Surgical technique:<br>- incision: IntraLase™ femtosecond laser<br>- implantation depth: 382 µm<br>- tunnel parameters: 6 mm optical zone, 2 mm size incision and 2 access cuts for the tunnels<br>- cross-linking: yes, preoperative cross-linking<br>Postoperative medication: mydriatic drops, netilmicin dexamethasone eyedrops, therapeutic soft con-tactlens
Outcomes	UCVA, Kmax, cornea aberrations, thinnest point; coma

Quality assessment

**Item**	**Authors' judgement**
Consecutive patients?	Yes
Reasons for inclusion reported?	No
Reasons for exclusion reported?	No
Were point estimates and measures of variability presented for the outcome measures?	No
Was calculation of statistical power reported?	No

Risk of bias

**Item**	**Authors' judgement**
Data collector blinded?	No
Outcome assessor blinded?	No
Free of selective reporting?	Unclear
Free of other bias?	No

Mechleb 2024

Methods	Type of study: Interventional study<br>Study design: Prospective case series
Participants	Country: France<br>Number of individuals with keratoconus: 10<br>Number of eyes with keratoconus: 10<br>Subgroups:<br>- group A: maximal keratometry Kmax < 75D<br>- group B Kmax > 75D<br>Inclusion criteria: patients diagnosed with keratoconus, with no restrictions on the stage of the condition<br>Exclusion criteria: -
Interventions	Donor tissue:<br>- levy: trimming<br>- cutout: mean arc length 137±28.7 degrees<br>- soaking: -<br>- dehydration / rehydration: dehydrated<br>Surgical technique:<br>- incision: blade / laser<br>- implantation depth: 250 µm<br>- tunnel parameters:<br>- cross-linking: no<br>Postoperative medication: topical ciprofloxacin 0.3% and tobramycin 0.3% 4x/day for 1 week and topical dexamethasone 0.1% 4x/day for 1 week, which was tapered off over a period of 1 month
Outcomes	CDVA (logMAR), SE (diopters), sphere (diopters), cylinder (diopters), refractive flat axis (degree), Kmax (diopters), K1 flat (D), K2 steep (D), K1 axis (degree), y-value steepest point (mm), total RMS (um), HOA RMS (um), comatic 90 degrees (um), spherical aberration (um)

Quality assessment

**Item**	**Authors' judgement**
Consecutive patients?	Unclear
Reasons for inclusion reported?	Yes
Reasons for exclusion reported?	No

Were point estimates and measures of variability presented for the outcome measures?	Yes
Was calculation of statistical power reported?	No

Risk of bias

**Item**	**Authors' judgement**
Data collector blinded?	No
Outcome assessor blinded?	No
Free of selective reporting?	Unclear
Free of other bias?	No

Mechleb 2025

Methods	Type of study: Interventional study<br>Study design: Retrospective case series
Participants	Country: France<br>Number of individuals with keratoconus: 71<br>Number of eyes: 79<br>Subgroups: -<br>Inclusion criteria: individuals aged 18 years and above with a confirmed diagnosis of keratoconus, patients seeking visual improvement and/or experiencing intolerance to rigid gas-permeable contact lenses, and those with keratoconus classified as grade 1 to 4 according to the Amsler–Krumeich classification, provided their condition was stable<br>Exclusion criteria: patients with severe and progressing keratoconus [defined as an annual increase of 1.50 dipoter (D) or more], those with corneal opacity, a history of hydrops or previous ocular surgery, and individuals with active systemic or ocular diseases
Interventions	Donor tissue:<br>- levy: femtosecond (FS) laser set to anterior lamellar keratoplasty mode<br>- cutout: multiple concentric cuts on donor corneal grafts at a consistent depth of 400 µm to create overlapping annuli, CAIRS width was determined using Bteich nomogram, with adjustments for advanced keratoconus, and he arc length of the CAIRS primarily depended on subjective refraction<br>- soaking: -<br>- hydration / dehydration: dehydration<br>Surgical technique:<br>- incision: femtosecond laser<br>- implantation depth: 250 µm<br>- tunnel parameters: 5-mm optical zone<br>- cross-linking: no cross-linking<br>Postoperative medication: topical antibiotics (cipro-floxacin 0.3% and tobramycin 0.3%) and steroids (dexamethasone 0.1%), administered 4 times daily for a week and tapered over a month
Outcomes	UDVA (logMAR), CDVA (logMAR), SE (D), sphere (D), cylinder (D), refractive flat axis (degree), Kmax (D), Km (D), K1 (flat) (D), K2 (steep) (D), K1 axis (degrees), CCT (µm), y-value steepest point (mm), total RMS (µm), HOA RMS (µm), coma 90 degrees (µm), spherical aberration (µm)

Quality assessment

**Item**	**Authors' judgement**
Consecutive patients?	Unclear
Reasons for inclusion reported?	Yes

Reasons for exclusion reported?	Yes
Were point estimates and measures of variability presented for the outcome measures?	Yes
Was calculation of statistical power reported?	No

Risk of bias

**Item**	**Authors' judgement**
Data collector blinded?	No
Outcome assessor blinded?	No
Free of selective reporting?	Unclear
Free of other bias?	No

Nacaraglu 2023

Methods	Type of study: Interventional study<br>Study design: Retrospective case series
Participants	Country: Turkey<br>Number of individuals with keratoconus: 49<br>Number of eyes with keratoconus: 65<br>Subgroups: -<br>Inclusion criteria: age > 18 years old, keratoconus, CDVA < 0.3 logMAR and contact lens intolerance or who did not prefer to use contacts<br>Exclusion criteria: history of previous corneal / intraocular surgery, post-LASIK corneal ectasia, central or paracentral scaring; history of viral keratitis, central corneal thickness < 400 um
Interventions	Donor tissue:<br>- levy: trimming: prepared CAIRSs by KeraNatural<br>- cutout: multiple / laser / punch + size: arc length of approximately 160°<br>- soaking: dyeing / sterilization<br>- dehydration / rehydration<br>Surgical technique:<br>- incision: blade / laser<br>- implantation depth: depth of 35% of the minimum pachymetry in a 7 mm central optical zone<br>- tunnel parameters:<br>*depth<br>*position: inner diameter of 4 mm and an outer diameter of 7.5 mm<br>- cross-linking<br>Postoperative medication:
Outcomes	UCVA (logMAR), CDVA (logMAR), SE (D), K1 (D), K2 (D), Kmax (D), K average (D), CCT (um), AME (um), PME (um), BFS anterior (R), BFS posterior (R), anterior elevation, posterior elevation

Quality assessment

**Item**	**Authors' judgement**
Consecutive patients?	Yes
Reasons for inclusion reported?	Yes
Reasons for exclusion reported?	Yes

Were point estimates and measures of variability presented for the outcome measures?	Yes
Was calculation of statistical power reported?	Yes

Risk of bias

**Item**	**Authors' judgement**
Data collector blinded?	No
Outcome assessor blinded?	No
Free of selective reporting?	Yes
Free of other bias?	No

Yakut 2025

Methods	Type of study: Interventional study<br>Study design: Prospective case series
Participants	Country: Turkey<br>Number of individuals with keratoconus: 27<br>Number of eyes: 35<br>Subgroups: -<br>Inclusion criteria: keratoconus patients with a total corneal thickness of at least 350 μm at the thinnest point, a clear cornea, asymmetrical non-central cones, and documented contact lens intolerance history<br>Exclusion criteria: a history of corneal or intraocular surgery or viral keratitis, the presence of glaucoma or any retinal disease, connective tissue or systemic autoimmune diseases, and pregnancy or lactation during the study period
Interventions	Donor tissue:<br>- levy: KeraNatural<br>- cutout: -<br>- soaking: -<br>- hydration / dehydration: NR<br>Surgical technique:<br>- incision: femtosecond laser (iFS® 150kH, Intralase, Abbott Medical Optics Inc, CA, USA)<br>- implantation depth: 250 μm<br>- tunnel parameters: inner and outer diameters of the tunnel were 4 mm and 7.5 mm<br>- cross-linking: no<br>Postoperative medication: topical moxifloxacin 0.5% (Vigamox®, Alcon Laboratories, Fort Worth, TX) administered four times a day for 1 week along with 0.1% dexamethasone (Maxidex®, Alcon Laboratories, Belgium) and preservative-free artificial tears administered five times a day for 4 weeks
Outcomes	UDVA (Snellen), CDVA (Snellen), Sphere (D), Cylinder (D), SE (D), K1 (D), K2 (D), Kmean (D), Kmax (D), CCT (μm)

Quality assessment

**Item**	**Authors' judgement**
Consecutive patients?	Unclear
Reasons for inclusion reported?	Yes
Reasons for exclusion reported?	Yes

Were point estimates and measures of variability presented for the outcome measures?	Yes
Was calculation of statistical power reported?	No

Risk of bias

**Item**	**Authors' judgement**
Data collector blinded?	No
Outcome assessor blinded?	No
Free of selective reporting?	Unclear
Free of other bias?	No

Yukecul 2024

Methods	Type of study: Interventional study<br>Study design: Retrospective case series
Participants	Country: Turkey<br>Number of individuals with keratoconus: 47<br>Number of eyes with keratoconus: 67 (30 eyes in the CXL group and 37 eyes in the non-CXL group)<br>Subgroups: CXL and Non-CXL group<br>Inclusion criteria: age > 20 years old, contact lens intolerance, corneal thickness > 400 µm at the implantation area, asymmetric non-central cones, contact lens intolerance<br>Exclusion criteria: history of a previous corneal/intraocular surgery; central or paracentral scaring; history of viral keratitis, glaucoma or any retinal disease, autoimmune and connective tissue diseases and pregnant or lactating
Interventions	Donor tissue:<br>- levy: trimming: prepared CAIRSs by KeraNatural<br>- cutout: multiple / laser / punch + size: arc length of approximately 160°<br>- soaking: dyeing / sterilization<br>- dehydration / rehydration<br>Surgical technique:<br>- incision: blade / laser<br>- implantation depth: depth of 35% of the minimum pachymetry in a 7 mm central optical zone<br>- tunnel parameters:<br>*depth: 200 um<br>*position: inner diameter of 4 mm and an outer diameter of 7.5 mm<br>- cross-linking<br>Postoperative medication: topical moxifloxacin 0.5% 4x/day for 1 week and dexamethasone 0.1%
Outcomes	SE (D), sphere (D), cylinder (D), UDVA (decimal), CDVA (decimal), K1 (D), K2 (D), Kmean (D), Kmax (D), pachymetry

Quality assessment

**Item**	**Authors' judgement**
Consecutive patients?	Unclear
Reasons for inclusion reported?	Yes
Reasons for exclusion reported?	Yes

Were point estimates and measures of variability presented for the outcome measures?	Yes
Was calculation of statistical power reported?	No

Risk of bias

**Item**	**Authors' judgement**
Data collector blinded?	No
Outcome assessor blinded?	No
Free of selective reporting?	Unclear
Free of other bias?	No

Section S3– Search strategy

Search strategy for Medline (OVID)

01. "Keratoconus" [Mesh]

02. keratocon*

03. 1 or 2

04. (corneal allogenic intrastromal ring segment*)

05. CAIRS

06. 4 or 5

07. 3 and 6

Search strategy for Embase

01. keratoconus.sh.
02. keratocon$.af.
03. 1 or 2
04. corneal allogenic intrastromal ring segment.sh.
05. CAIRS.af.
06. 4 or 5
07. 3 and 6

Section S4 – PRISMA 2020 checklist

Section and Topic	Item #	Checklist item	Location where item is reported
**TITLE**			
Title	1	Identify the report as a systematic review.	1
**ABSTRACT**			
Abstract	2	See the PRISMA 2020 for Abstracts checklist.	1
**INTRODUCTION**			
Rationale	3	Describe the rationale for the review in the context of existing knowledge.	1-2
Objectives	4	Provide an explicit statement of the objective(s) or question(s) the review addresses.	2
**METHODS**			
Eligibility criteria	5	Specify the inclusion and exclusion criteria for the review and how studies were grouped for the syntheses.	2-3
Information sources	6	Specify all databases, registers, websites, organisations, reference lists and other sources searched or consulted to identify studies. Specify the date when each source was last searched or consulted.	2
Search strategy	7	Present the full search strategies for all databases, registers and websites, including any filters and limits used.	2
Selection process	8	Specify the methods used to decide whether a study met the inclusion criteria of the review, including how many reviewers screened each record and each report retrieved, whether they worked independently, and if applicable, details of automation tools used in the process.	Section S3
Data collection process	9	Specify the methods used to collect data from reports, including how many reviewers collected data from each report, whether they worked independently, any processes for obtaining or confirming data from study investigators, and if applicable, details of automation tools used in the process.	3
Data items	10a	List and define all outcomes for which data were sought. Specify whether all results that were compatible with each outcome domain in each study were sought (e.g. for all measures, time points, analyses), and if not, the methods used to decide which results to collect.	3

Section and Topic	Item #	Checklist item	Location where item is reported
	10b	List and define all other variables for which data were sought (e.g. participant and intervention characteristics, funding sources). Describe any assumptions made about any missing or unclear information.	3
Study risk of bias assessment	11	Specify the methods used to assess risk of bias in the included studies, including details of the tool(s) used, how many reviewers assessed each study and whether they worked independently, and if applicable, details of automation tools used in the process.	3
Effect measures	12	Specify for each outcome the effect measure(s) (e.g. risk ratio, mean difference) used in the synthesis or presentation of results.	3
Synthesis methods	13a	Describe the processes used to decide which studies were eligible for each synthesis (e.g. tabulating the study intervention characteristics and comparing against the planned groups for each synthesis (item #5)).	3
	13b	Describe any methods required to prepare the data for presentation or synthesis, such as handling of missing summary statistics, or data con-versions.	3
	13c	Describe any methods used to tabulate or visually display results of individual studies and syntheses.	3
	13d	Describe any methods used to synthesize results and provide a rationale for the choice(s). If meta-analysis was performed, describe the model(s), method(s) to identify the presence and extent of statistical heterogeneity, and software package(s) used.	3
	13e	Describe any methods used to explore possible causes of heterogeneity among study results (e.g. subgroup analysis, meta-regression).	3
	13f	Describe any sensitivity analyses conducted to assess robustness of the synthesized results.	-
Reporting bias assessment	14	Describe any methods used to assess risk of bias due to missing results in a synthesis (arising from reporting biases).	3
Certainty assessment	15	Describe any methods used to assess certainty (or confidence) in the body of evidence for an outcome.	3
**RESULTS**			

Section and Topic	Item #	Checklist item	Location where item is reported
Study selection	16a	Describe the results of the search and selection process, from the number of records identified in the search to the number of studies included in the review, ideally using a flow diagram.	5-7 and Figure 3
	16b	Cite studies that might appear to meet the inclusion criteria, but which were excluded, and explain why they were excluded.	Figure 3
Study characteristics	17	Cite each included study and present its characteristics.	Section S2 and Table 2
Risk of bias in studies	18	Present assessments of risk of bias for each included study.	Section S2
Results of individual studies	19	For all outcomes, present, for each study: (a) summary statistics for each group (where appropriate) and (b) an effect estimate and its precision (e.g. confidence/credible interval), ideally using structured tables or plots.	Section S1
Results of syntheses	20a	For each synthesis, briefly summarise the characteristics and risk of bias among contributing studies.	5-7
	20b	Present results of all statistical syntheses conducted. If meta-analysis was done, present for each the summary estimate and its precision (e.g. confidence/credible interval) and measures of statistical heterogeneity. If comparing groups, describe the direction of the effect.	Section S1and Table 4-7
	20c	Present results of all investigations of possible causes of heterogeneity among study results.	Section S1
	20d	Present results of all sensitivity analyses conducted to assess the robustness of the synthesized results.	-
Reporting biases	21	Present assessments of risk of bias due to missing results (arising from reporting biases) for each synthesis assessed.	Section S2
Certainty of evidence	22	Present assessments of certainty (or confidence) in the body of evidence for each outcome assessed.	Section S1and Table 4-7
**DISCUSSION**			
Discussion	23a	Provide a general interpretation of the results in the context of other evidence.	15-17
	23b	Discuss any limitations of the evidence included in the review.	16-17
	23c	Discuss any limitations of the review processes used.	16-17

Section and Topic	Item #	Checklist item	Location where item is reported
	23d	Discuss implications of the results for practice, policy, and future research.	15-17
**OTHER INFORMATION**			
Registration and protocol	24a	Provide registration information for the review, including register name and registration number, or state that the review was not registered.	2
	24b	Indicate where the review protocol can be accessed, or state that a protocol was not prepared.	2
	24c	Describe and explain any amendments to information provided at registration or in the protocol.	-
Support	25	Describe sources of financial or non-financial support for the review, and the role of the funders or sponsors in the review.	17
Competing interests	26	Declare any competing interests of review authors.	17
Availability of data, code and other materials	27	Report which of the following are publicly available and where they can be found: template data collection forms; data extracted from included studies; data used for all analyses; analytic code; any other materials used in the review.	Section S1

From: Page MJ, McKenzie JE, Bossuyt PM, Boutron I, Hoffmann TC, Mulrow CD, et al. The PRISMA 2020 statement: an updated guideline for reporting systematic reviews. BMJ 2021;372:n71. doi: 10.1136/bmj.n71

For more information, visit: http://www.prisma-statement.org/

References

1. Bteich, Y., et al., *Corneal Allogenic Intrastromal Ring Segments (CAIRS) for Corneal Ectasia: A Comprehensive Segmental Tomography Evaluation.* J Refract Surg, 2023. **39**(11): p. 767-776.
2. Bteich, Y., et al., *Femtosecond Laser-Assisted Graft Preparation and Implantation of Corneal Allogeneic Intrastromal Ring Segments for Corneal Ectasia: 1-Year Results.* Cornea, 2025. **44**(3): p. 360-367.
3. Haciagaoglu, S., et al., *Allograft corneal ring segment for keratoconus management: Istanbul nomogram clinical results.* Eur J Ophthalmol, 2022: p. 11206721221142995.
4. Keskin Perk, F.F.N., et al., *Long-Term Results of Sterile Corneal Allograft Ring Segments Implantation in Keratoconus Treatment.* Cornea, 2025. **44**(4): p. 475-482.
5. Kirgiz, A., et al., *Clinical outcomes of femtosecond laser-assisted corneal allogenic intrastromal ring segment (CAIRS) in the treatment of keratoconus.* Clin Exp Ophthalmol, 2024. **52**(7): p. 713-723.
6. Nacaroglu, S.A., et al., *Efficacy and safety of intracorneal allogenic ring segment implantation in keratoconus: 1-year results.* Eye (Lond), 2023. **37**(18): p. 3807-3812.
7. Yucekul, B., et al., *Effect of Corneal Allogenic Intrastromal Ring Segment (CAIRS) Implantation Surgery in Patients With Keratoconus According to Prior Corneal Cross-linking Status.* J Refract Surg, 2024. **40**(6): p. e392-e397.
8. Mechleb, N., *A Technique of Multiple Corneal Allogeneic Ring Segments Prepared from a Single Corneal Graft: A Case Series.* 2024.
9. Mazzotta, C., et al., *Crosslinked All-Femtosecond Laser-Cut Corneal Allogenic Intracorneal Ring Segments (AFXL CAIRSs): Pilot Ex Vivo Study and Report of First Two Cases Performed in Italy.* J Clin Med, 2024. **13**(19).
10. Hayashi, T., et al., *A Manual Technique for Corneal Allogeneic Intrastromal Ring Segments Without a Femtosecond Laser.* Cornea, 2025. **44**(8): p. 1053-1057.
11. Colak, D., et al., *Implantation of Opposing Dual Corneal Allogeneic Intrastromal Ring Segments With Topography-Guidance: A Novel Method for Improved Corneal Reshaping.* Cornea, 2025.
12. Bteich, Y., et al., *Asymmetric All-Femtosecond Laser-Cut Corneal Allogenic Intrastromal Ring Segments.* J Refract Surg, 2023. **39**(12): p. 856-862.
13. Coscarelli, S., S.P. Coscarelli, and L. Torquetti, *Donut-shaped Corneal Allogeneic Intrastromal Segment as an Alternative to Deep Anterior Lamellar Keratoplasty in Advanced Keratoconus.* Cornea, 2024. **43**(5): p. 658-663.
14. Mechleb, N., et al., *Six-Month Results of Multiple Femtosecond Laser-Assisted Corneal Allogeneic Ring Segments Implantation: A Case Series.* Cornea, 2025.
15. Asfar, K.E., *Corneal Allogenic Intrastromal Ring Segments (CAIRS) Versus Synthetic Segments: A Single Segment Comparative Analysis Using Propensity Score Matching.* 2024.
16. Jacob, S., et al., *Corneal Allogenic Intrastromal Ring Segments (CAIRS) Combined With Corneal Cross-linking for Keratoconus.* J Refract Surg, 2018. **34**(5): p. 296-303.
17. Jacob, S., et al., *Customized corneal allogenic intrastromal ring segments (CAIRS) for keratoconus with decentered asymmetric cone.* Indian J Ophthalmol, 2023. **71**(12): p. 3723-3729.
18. Yakut, B., et al., *Postoperative corneal remodeling after corneal allograft intrastromal ring segment (CAIRS) implantation for keratoconus: An OCT study.* Indian J Ophthalmol, 2025. **73**(3): p. 382-388.
19. Mechleb, N. A Technique of Multiple Corneal Allogeneic Ring Segments Prepared from a Single Corneal Graft: A Case Series. *Cornea* **2024**, *43*, 1441–1447.
